# Structure and Conformational Mobility of OLED-Relevant 1,3,5-Triazine Derivatives

**DOI:** 10.3390/molecules28031248

**Published:** 2023-01-27

**Authors:** Georgi M. Dobrikov, Yana Nikolova, Ivaylo Slavchev, Miroslav Dangalov, Vera Deneva, Liudmil Antonov, Nikolay G. Vassilev

**Affiliations:** 1 Institute of Organic Chemistry with Centre of Phytochemistry, Bulgarian Academy of Sciences, Acad. G. Bonchev Str., Bl. 9, 1113 Sofia, Bulgaria; 2 Institute of Electronics, Bulgarian Academy of Sciences, 72 Tsarigradsko Chaussee Blvd., 1784 Sofia, Bulgaria

**Keywords:** 1,3,5-Triazine, propeller, dynamic NMR spectroscopy, DFT calculation

## Abstract

A series of OLED-relevant compounds, consisting of 1,3,5-triazine core linked to various aromatic arms by amino group, has been synthesized and characterized. The studied compounds exist in solution as a mixture of two conformers, a symmetric propeller and asymmetric conformer, in which one of the aromatic arms is rotated around the C-N bond. At temperatures below −40 °C, the VT NMR spectra in DMF-d7 are in a slow exchange regime, and the signals of two conformers can be elucidated. At temperatures above 100 °C, the VT NMR spectra in DMSO-d6 are in a fast exchange regime, and the averaged spectra can be measured. The ratio of symmetric and asymmetric conformers in DMF-d7 varies from 14:86 to 50:50 depending on the substituents. The rotational barriers of symmetric and asymmetric conformers in DMF-d7 were measured for all compounds and are in the interval from 11.7 to 14.7 kcal/mol. The ground-state energy landscapes of the studied compounds, obtained by DFT calculations, show good agreement with the experimental rotational barriers. The DFT calculations reveal that the observed chemical exchange occurs by the rotation around the C(1,3,5-triazine)-N bond. Although some of the compounds are potentially tautomeric, the measured absorption and emission spectra do not indicate proton transfer neither in the ground nor in the excited state.

## 1. Introduction

The design of new OLEDs is strongly dependent on the appropriate HOMO and LUMO energy levels. In this regard, a wide spectrum of different classes of organic compounds (exclusively aromatic) is tested experimentally. Although most of the OLEDs used in practice are polymers [1], significant efforts are also being made to develop monomeric analogues. A recent trend in their design is to replace the linear structure with a star-shaped one [2,3]. A significant change in the properties of the material can be achieved by varying the “arms” and the core of the star-shaped architecture [4,5]. Until now, fluorene derivatives have mainly been reported as compounds, where the star-shaped structures are suitable for such applications [6,7]. Other studied star-shaped structures of particular interest are 1,3,5-triazines, as they possess desired optoelectronic and photovoltaic properties [8,9,10]. Moreover, a combination of 1,3,5-triazines and carbazole moieties in single molecules significantly improves their performance [11,12,13,14,15]. Different aspects of carbazole properties and their use in light-emitting applications have recently been reviewed [16]. In the study of *Zassowski* et al. [17], it was shown that star-shaped compounds of this type demonstrate even better optoelectronic properties than their linear analogues. Numerous studies have focused on devices based on polymers with an incorporated carbazole building block [18,19,20,21]. Carbazole derivatives are an effective host material for phosphorescent additives in PhOLEDs [15,22,23,24,25,26,27,28], an active component of fluorescent OLEDs with thermally activated delayed fluorescence (TADF) [14,29,30,31,32,33,34,35,36,37,38,39,40,41,42,43] or molecules with a hybridized local and charge state of transfer (HLCT) [44,45,46,47,48,49,50]. Computer-aided design is also currently being used to suggest more stable and more efficient organic diodes [51]. It emphasizes machine learning-based approaches and in silico pre-screening. Simulations are performed to predict the device properties depending on the chemical composition prior to synthesis.

In the current work, we present a detailed study of the conformational mobility of tri-substituted 1,3,5-triazines presented by *Zassowski* et al. [17] and of several new 1,3,5-triazine compounds with various substituents with potential OLED applicability. The new 1,3,5-triazine compounds include aromatic substituents with hydroxyl groups capable of forming intramolecular hydrogen bonds, which could stabilize the planar structure of the compounds, modulate their properties, and possibly stimulate the proton transfer.

## 2. Results and Discussion

### 2.1. Synthesis

**Scheme 1:**
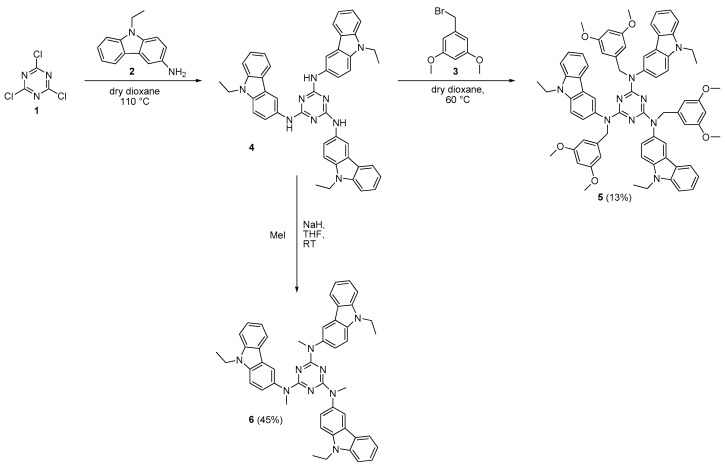
The synthesis of compounds **4**–**6**.

**Scheme 2:**
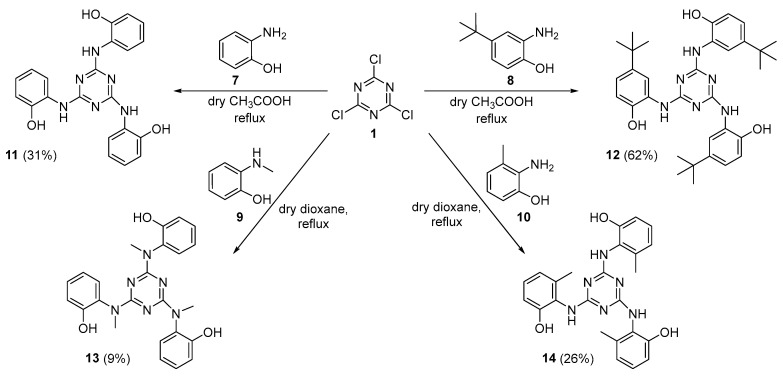
The synthesis of compounds **11**–**14**.

The compounds investigated in this article are synthesized according to the procedures presented in Scheme 1 and Scheme 2. Our synthetic approach was based on the preparation of a small series of sterically hindered 1,3,5-triazines, bearing three equal N-bridged substituents. All compounds (except **4** and **6** [17]) are new and were obtained in high purity. Compound **4** (Scheme 1) was prepared as a result of a reaction between cyanuric chloride (**1**) and 9-ethyl-9H-carbazol-3-amine (**2**) in refluxing anhydrous dioxane. Alkylation of **4** with 3,5-dimethoxybenzylbromide (**3**) in dry dioxane led to target product **5**. Compound **6** was prepared in a moderate yield at room temperature through reaction of **4** with methyliodide in the presence of NaH. According to Scheme 2, reactions of **1** with aminophenols **7**–**10** lead to corresponding products **11**–**14** in low to moderate yields. Due to the different solubility of **7**–**10**, an efficient general procedure for synthesis of **11**–**14** is not applicable [52]. Therefore, each reaction was performed in different solvents and conditions. Compounds **11** and **12** were prepared through refluxing anhydrous acetic acid, while compounds **13** and **14** were prepared through refluxing anhydrous dioxane. Low yields of compounds **5, 11, 13** and **14** were observed after purification by recrystallizations.

### 2.2. Potential Tautomerism

**Scheme 3:**
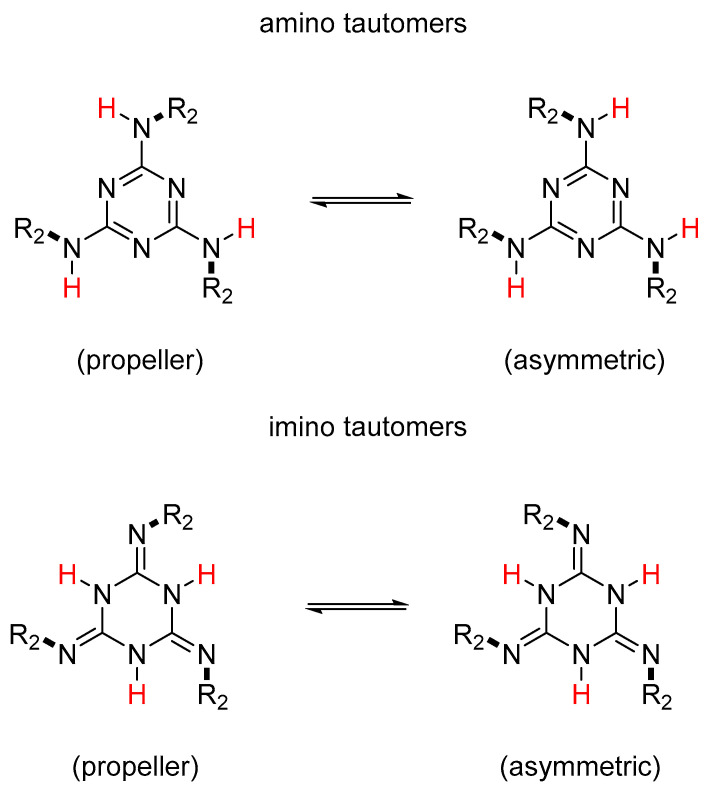
Border cases of the possible tautomers and corresponding isomers.

Some of the studied 1,3,5-triazine compounds (**4**, **11**, **12** and **14**) may exist in amino and imino tautomeric forms (Scheme 3), each of them exhibiting different dynamic isomerization. The first one is proton transfer by itself. The second one is *E/Z* isomerization between the imino tautomers, stabilized by conjugation with the aromatic substituents. The third one is conformational isomerism of amino tautomers due to restricted rotation about the amino triazine single bond. The overall situation supposes that up to nineteen possible isomers could be expected as a combination of all possible processes. In compounds **5**, **6** and **13**, the amino–imino tautomerism is not possible, while in **11**, **12** and **14**, an enol-keto proton transfer from the OH group in the phenyl ring to the triazine nitrogen cannot be additionally excluded.

Simple considerations based on the aromaticity of the triamino derivatives lost in the imino tautomers [53] and molecular mechanics calculations did not conclusively confirm the preference of the amino or imino form. Definitive proof was given by the dynamic NMR investigations and X-ray structural determination of 2-chloro-4,6-bis(pyrazolylamino)-1,3,5-triazine [54], which, due to its low symmetry, exists in three different conformations of the amino tautomer.

In the current case, an a priori exclusion of some tautomers is not evident, but some logical consideration can help to distinguish the processes. Bearing in mind that both (amino and imino) tautomers have different conjugation systems and different polarity, they can easily be detected by their UV-Vis absorption spectra, which should be solvent-dependent [55]. The amino–imino proton exchange proceeds through a four-membered ring, which leads, without solvent assistance, to an increase of the transition state energy [56] and makes the process possible to be detected by NMR spectroscopy. Recently, the transition state through the four-membered ring in 1,3,5-triazine derivatives has been estimated by DFT calculation to be about 50 kcal/mol [57]. In addition, compounds **5** and **6**, where tautomerism is not possible, can be used as model compounds of **4**, while **13** is a reference compound for the series **11**–**14**. The ground-state enol–keto proton transfer in the series **11**–**14** is also not likely due to a variety of reasons: the formed tautomer, originating from migration of the OH proton to the triazine nitrogen atom, is zwitterionic in nature; it is not stabilized by re-arrangement of the electronic density and proceeds through hydrogen bonding in the seven-membered ring, characterized by substantial nonplanarity. If observed in the ground state, it should be characterized by a red shift in the absorption spectra in polar solvents [58], while the relatively low barrier of proton exchange makes it not detectable by the NMR. The process is much more likely in the excited state and, if it occurs, could feature large Stokes shifts in the measured emission [59].

**Figure 1:**
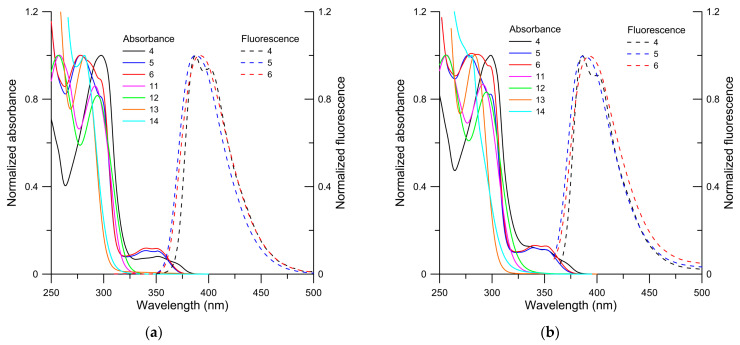
Absorption and emission spectra of studied compounds in: (**a**) acetonitrile; (**b**) dichloromethane.

The absorption and emission spectra of the studied compounds are shown in Figure 1. The spectral data in acetonitrile are compared in Table 1 with the TD-DFT predicted absorption of the amine tautomers. Several conclusions can be made considering them. Considering series **4**–**6**, the shapes of all three compounds are very similar and there are no solvent-dependent spectral changes going from dichloromethane to acetonitrile. This excludes the existence of tautomeric equilibrium at room temperature. The predicted absorbance of the amino tautomers very nicely corresponds, both in terms of position and intensity, to the experimentally measured ones. The observed weak emission (the maximal quantum yield is 5% in **6**), attributed to the existence of amine nitrogen atoms, is also similar in shape. The values of the quantum yields are similar to those measured and reported in [17] for similar compounds. The largest Stokes shift is below 2500 cm^−1^, which excludes excited state proton transfer.

In the case of **11**–**14**, as expected, the spectra of the studied compounds are similar in shape and, again, not solvent-dependent. This fact, along with the absence of emissions, clearly indicates that no proton transfer from the OH to the triazole nitrogen occurs. The steric hindrance in **13** and **14**, hindering the O-H…N hydrogen bonding and the overall conjugation, leads to a slight blue shift in the long-wavelength absorption maxima. Our DFT calculations predict that the symmetric imino conformer is less stable than the symmetric amino conformer (Scheme 3) by ca. 20 kcal/mol.

### 2.3. Dynamic NMR Spectroscopy, Conformational Analysis and Molecular Geometry

**Figure 2:**
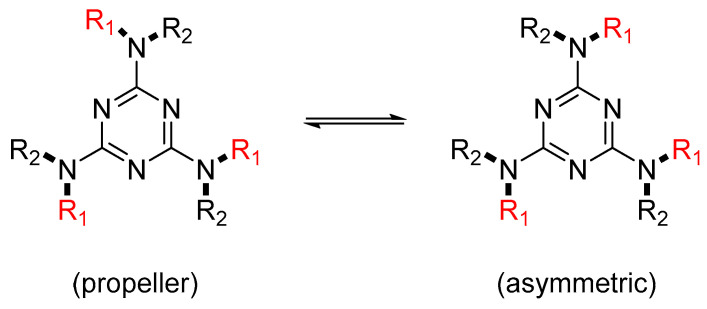
Possible conformers for the 1,3,5-triazine derivatives **4**, **5**, **6**, **11**, **12**, **13** and **14**. Substituents R_1_ and R_2_ are presented in Table 2.

The structures of the newly synthesized compounds were confirmed by 1D and 2D NMR spectra. Conformational exchanges occurred in solution at rates that were intermediate on the NMR time scale. The possible conformers (propeller and asymmetric) of the studied compounds are presented in Figure 2.

The population of symmetric and asymmetric conformers of the studied compounds in DMF-d7 vary from 14%/86% in compound **6** to 50%/50% in compound **4** (Table 2). These ratios are measured from ^1^H NMR spectra at the lowest measured temperature (223 or 233 K).

The measured rotational barriers between the conformers, presented in Table 3 together with the theoretically predicted values, show that there is a good correspondence between the theoretical and experimental data. The differences almost fall within the expected deviation of theoretical values at this level of theory.

### 2.4. NMR Structure Determination

The ^1^H NMR spectra of compound **11** at 293 K showed broad signals for the OH, NH, H-3 and H-4 protons (Figure S10). The NMR spectra were recorded in CDCl_3_, DMSO-d6 and DMF-d7 solutions at different temperature intervals. At 223 K in DMF-d7, the signals were split, and four signals were observed for every proton. One signal of high intensity and three similar signals of medium intensity were observed. This pattern could be assigned to two isomeric compounds, one symmetrical conformer and one asymmetrical conformer (Figure S10). A similar behavior was observed in the ^13^C NMR spectra. The NMR spectra of compound **11** at 293 K in DMSO and DMF solutions showed broad signals that coalesce to give signals for a single compound at 353 K in DMSO. Similarly, for compounds **4**–**6** and **12**–**14** at 223 K (DMF), two isomeric compounds could be detected: one symmetric and one asymmetrical conformer. Upon heating the samples to 343–403 K, the signals coalesced to show the presence of one single molecule in DMSO (^1^H and ^13^C spectra in the supporting information).

Assignment of the NMR signals to the two conformers was performed at a low temperature in DMF-d7 solutions. At a high temperature in DMSO-d6, due to rapid exchange of the NMR signals, they were also assigned.

Table 2 shows the ratio of isomers determined by ^1^H-NMR spectroscopy.

### 2.5. Dynamic NMR Studies of Compounds ***4**–**6*** and ***11**–**14***

Using dynamic NMR spectroscopy (DNMR), we studied the restricted rotation of triazines **4**–**6** and **11**–**14** (Scheme 1). The most frequently used methods for deriving the exchange rate constants are line shape analysis and magnetization transfer [60,61]. Their application becomes inefficient, difficult and even impossible when the number of exchangeable forms increases or the difference in chemical shifts decreases. In such multi-site systems or when the dispersion of the signals is small, the use of 2D exchange spectroscopy (2D EXSY) is preferred. In 2D exchange spectroscopy, interference between processes is avoided and the rate constants for chemical exchange of each process can be estimated from the corresponding 2D matrix of integral intensities using the relationship between the integral intensities, mixing time and the rate constants [62]. Further, 2D EXSY has been applied to resolve numerous complex kinetic processes in many fields, including organometallic chemistry, metallotropy, fluxional behavior and conformational studies [63,64,65,66,67,68,69]. In addition, 1D EXSY techniques have also been introduced and successfully applied [70,71]. We applied the 2D Rotating-frame Overhauser Enhancement SpectroscopY (2D ROESY) [72] to study the chemical exchange because in ROESY experiments, the signals generated by chemical exchange differ from NOE signals [73], unlike in the NOESY experiment, where these effects may produce magnetization in the same direction. The ROESY experiment is affected by some possible complications [72], such as COSY-type crosspeaks between *J*-coupled spins, TOCSY transfers within J-coupled spin systems, offset dependencies of spin-lock fields, and zero-quantum (ZQ) artifacts, which may influence the quantitative applications of crossrelaxation. In order to avoid these artifacts, a T-ROESY [74] NMR experiment was developed. The offset dependencies of the spinlock field have to be considered for quantitative applications [75]. Thiele et al. [76] published the ‘EASY ROESY’ experiment, which makes the offset dependency of cross-peak volumes negligible and reduces TOCSY transfers. An efficient ZQ suppression scheme [77] was recently incorporated in NOESY experiments. Recently, Pure Shift NOESY was introduced [78], and gradient-selected pure-shift EASY-ROESY techniques have become quantitative [79]. In our case, the exchange is between spin systems located in different substituents. Therefore, we cannot expect TOCSY or COSY artefacts that will affect the quantitative measurements of exchange rates.

The assignment of exchanging conformers in VT NMR spectra of complex systems is difficult without the application of an integrated approach, which combines methods of dynamic NMR study and DFT calculation. Dynamic NMR study provides information about the exchanging sites and the corresponding rate constants. The DFT calculations can help in conformer assignment by predicting their populations and/or NMR chemical shifts. The comparison of experimental and DFT calculated barriers can reveal the exchange mechanism. This protocol, including the combination of dynamic NMR and DFT calculation, was recently successfully applied for studying the structure and exchange mechanism of palladium complexes [65,66,69], ortho-diphenylphosphinobenzenecarboxamide ligands [67], galantamine derivatives [68], and atropisomers of 2,20-diaryl-1,10-binaphthalenes containing three stereogenic axes [80].

The experimentally determined rotational barriers of the studied compounds in DMF-d7 are within the interval from 11.7 to 14.7 kcal/mol (Table 3). It is known that arylamino-1,3,5-triazines show hindered rotation about the partially double exocyclic C–N bond, due to the n–πconjugation of the amine lone pair with the triazine ring [81]. The substituents that enhance the double character of the C–N bond increase the barrier to hindered rotation. On the other hand, the steric effects of substituents have to be taken into account. An additional factor that will influence the rotational barriers is the solvent and the possible interactions with the solvent.

It has been reported that the rotational barrier in three melamines (*N*,*N*-dimethylamino-bis(*N*-tert-butylamino)-s-triazine, tris(*N*-tert-butylamino)-s-triazine and tris(*N*-methylamino)-s-triazine) strongly depends on the solvent and increases in the order: CDCl_3_ (12.7–13.8 kcal/mol), CD_2_Cl_2_ (13.2–15.4 kcal/mol), Acetone-*d6* (13.6–15.0 kcal/mol) and DMF-*d7* (14.2–15.9 kcal/mol) [82].

The rotational barriers in our compounds fall in the range of reported values for arylamino1,3,5-triazines (59–77 kJ mol^−1^) (14–18 kcal/mol) [81], for 2-chloro-4,6-bis(pyrazolylamino)-1,3,5-triazines (50–76 kJ mol^−1^) (12–18 kcal/mol) [54], and for *N*^2^,*N*^4^,*N*^6^-tris(1*H*-pyrazolyl)-1,3,5-triazine-2,4,6-triamines (49–79 kJ mol^−1^) (12–19 kcal/mol) [83]. The rotational barriers of 2,4-diamino-1,3,5-triazine derivatives were estimated by coalescent methods and are in the range of 53–63 kJ mol^−1^ (13–15 kcal/mol) [84].

In order to better understand the role of the substituents, the solvent and the possible intramolecular hydrogen bonding on the rotational barrier height, we perform DFT calculations in the studied compounds.

### 2.6. DFT Calculations

**Figure 3:**
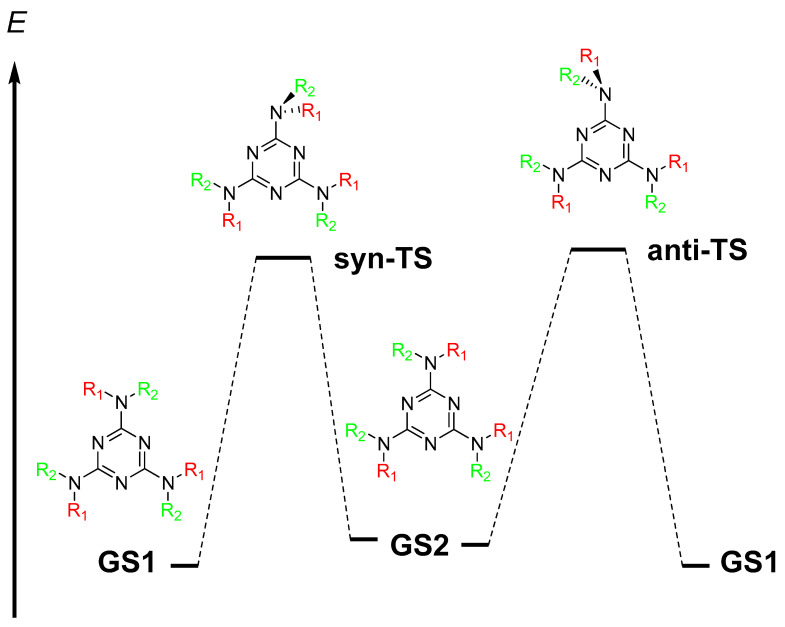
Hindered rotation in studied 1,3,5-triazines (GS1 is the symmetric conformer, GS2 is the asymmetric conformer, and syn-TS and anti-TS are the transition states).

In order to understand the trends in the populations of conformers and in the rotational barriers, we carried out a DFT conformational search in the ground state, which reveals the stable structures (GS) and transition state (TS) of all studied compounds (Figure 3).

Dynamic NMR study using 2D EXSY spectra provides information about the structure of exchanging conformers (one symmetric and one asymmetric conformer) and about the exchanging routes. DFT calculations of GS structures provide information about the thermodynamic stability of possible conformers. In most cases of exchange between complex structures or many conformers, the calculation of populations or prediction of chemical shifts helps in the conformer’s assignment. This is redundant here, as it is clear from the experiment who is who; moreover, even the application of broad base sets and modern DFT methods does not predict the experimental populations accurately enough (Table 2). The comparison of experimental and DFT calculated barriers can reveal the exchange mechanism. This integrated approach, which combines dynamic NMR spectroscopy and computational chemistry, has recently been successfully applied to study the structure and mechanism of exchange [63,64,65,66,67,68,69].

We begin the discussion with the results of the DFT calculations of the GS structures of our simplest compound. There are at least six possible GS conformations. GS1 is the most stable, and it represents the structure of the symmetric conformer. GS2 is obtained by rotation around the C_triazine_-N bond, and this structure is the asymmetric conformer. Further study of this structure led to the GS2_3H structure with three hydrogen bonds (Figure S24). This structure is predicted to be preferable by SMD(DMF)//M062X/TZVP calculations. Both structures (GS2 and GS2_3H) are consistent with the measured ^15^N NMR spectra of **1** at 243 K (Figures S22 and S23). Unfortunately, no correlation was observed in 1H,^15^N-HSQC NMR spectra between hydroxyl hydrogens and 1,3,5-triazine nitrogens. The GS3 is obtained by rotation around the C_Ph_-N bond.

The TS structures are two, syn and anti TS, with respect to the orientation of oxygen atoms. In syn TS, the oxygens are on the same side, while in anti TS, one of the oxygen molecules is on the other side of the 1,3,5-triazine plane (Figure S25). In a similar manner, there are two TS structures regarding rotation around the N-Ph bond: syn and anti TS (Figure S26). The calculated theoretical thermodynamic parameters of restricted rotation around the 1,3,5-triazine-N bond are in good agreement with the experimental values (Table S23 and Table 3). The calculated theoretical thermodynamic parameters of restricted rotation around the N-Ph bond correspond to free rotation around a single bond (Table S24).

The same protocol was applied for all studied compounds: calculation of two syn and anti GS structures and two syn and anti TS structures. The results from the calculations of rotation around the triazine-N bond are given in Table 3. The obtained theoretical thermodynamic parameters of restricted rotation around the triazine-N bond reproduce the experimental values well. The observed differences can be reduced by the inclusion of specific solvent interaction in DMF.

## 3. Materials and Methods

### 3.1. General

Reagents were of commercial grade and used without further purification. Tetrahydrofurane (THF) and dioxane were distilled over sodium/benzophenone. Commercial anhydrous acetic acid was used for the reactions. Thin-layer chromatography (TLC) was performed on aluminum sheets pre-coated with Merck Kieselgel 60 F_254_ 0.25 mm (Merck, Darmstadt, Germany). Flash column chromatography was carried out using Silica Gel 60 230–400 mesh (Fluka, Buchs, Switzerland). Melting points were determined in a capillary tube on the SRS MPA100 OptiMelt (Sunnyvale, CA, USA) automated melting point system (uncorrected). The NMR spectra were recorded on a Bruker Avance II+ 600 (600.13 for ^1^H NMR and 150.92 MHz for ^13^C NMR) spectrometer with TMS as the internal standard for chemical shifts (δ, ppm). ^1^H and ^13^C NMR data are reported as follows: chemical shift, multiplicity (s = singlet, d = doublet, t = triplet, q = quartet, br = broad, and m = multiplet), coupling constants *J* (Hz), integration, and identification. The assignment of the ^1^H and ^13^C NMR spectra was made on the basis of DEPT, COSY, HSQC, HMBC and NOESY experiments. Mass spectra (MS) were recorded on a Shimadzu Liquid Chromatograph Mass Spectrometer LCMS-2020. Direct MS-regime was applied; each compound was dissolved in MeOH with the following: concentration 1 mg/mL; injection volume 3–5 μL; mobile phase—1% formic acid in MeOH; and MS detector with electrospray ionization (ESI). MS spectra are reported as fragmentation in m/z with relative intensities (%). IR spectra were recorded using a Bruker Tensor 27 FTIR spectrometer. 

### 3.2. Synthesis

The synthesis and structures of the studied compounds in this article are shown in Scheme 1 and Scheme 2. Compounds **4** and **11**–**14** were obtained by nucleophilic aromatic substitution. Compounds **5** and **6** were obtained by substitution of the amine proton with methyl iodide or **3** in the presence of sodium hydride. Synthesized compounds are characterized by good solubility in the common organic solvents and can be purified using column chromatography with subsequent precipitation/crystallization.

***N,N’,N’’*-tris(9-ethyl-9H-carbazol-3-yl)-1,3,5-triazine-2,4,6-triamine** (**4**). In 40 mL dry dioxane, 0.784 g (4.25 mmol) cyanuric chloride (**1**) was dissolved. Then, 2.707 g (12.75 mmol) of K_3_PO_4_ and 2.681 g (12.75 mmol) of 3-amino-9-ethylcarbazole (**2**) were added, and the formed suspension was stirred under argon at 110 °C for 67 h. The reaction mixture was cooled to r.t. and 100 mL water was added. The formed precipitate was filtered and washed consequently with water, isopropanol and acetone. Finally, the product was dried in vacuo to give 2.069 g (69%) of pure **4** as white off powder. This led to decomp. >305 °C without melting. IR (KBr), *υ*/cm^−1^: 3400, 3376 (*υ*_NH_), 3050, 3020 (*υc_H_(Ar)*) and 1505 (*υ*_C=N_). MS: (ESI^+^ in MeOH with 1% HCOOH) 707 (M+1, 100%).

***N,N’,N’’*-tris(3,5-dimethoxybenzyl)-*N,N’,N’’*-tris(9-ethyl-9H-carbazol-3-yl)-1,3,5-triazine-2,4,6-triamine** (**5**). In 30 mL dry dioxane, we suspended 0.250 g (0.29 mmol) of **4** and 0.140 g (3.54 mmol) of NaH (65% in mineral oil). This suspension was stirred under argon for 1 h at 60 °C. Then, 0.655 g (2.83 mmol) of **3** was added and the reaction mixture was stirred under argon for 18 h at this temperature. After cooling, the mixture was evaporated to dryness and dissolved in DCM. The organic phase was washed with saturated aq. NaCl, dried over MgSO_4_ and evaporated to dryness. TLC—DCM:MTBE = 10:1. The crude product was purified by column chromatography: 60 g silica, phase 1 DCM for impurities; phase 2 DCM:MTBE = 10:1 for the product. After this column, the product was not pure enough. Next, purification by column chromatography was performed: 60 g silica, phase 1 DCM:acetone = 400:1 for impurities; phase 2 DCM:acetone = 200:1 for the product. Finally, the product was washed with 10 mL hot PE and dried in vacuo to give 0.105 g (26%) of pure **5** as a light beige powder. M.p. 129–130°C. IR (KBr), *υ*/cm^−1^: 3050, 3021 (*υc_H_(Ar)*) and 1539 (*υ*_C=N_). MS: (ESI^+^) 1157 (M+1, 100%).

***N,N’,N’’*-tris(9-ethyl-9H-carbazol-3-yl)-*N,N’,N’’*-trimethyl-1,3,5-triazine-2,4,6-triamine** (**6**). In 40 mL dry THF, we suspended 0.500 g (0.71 mmol) of **4** and 0.140 g (3.54 mmol) of NaH (65% in mineral oil). The mixture was stirred under argon for 1 h at r.t. Then, 0.18 mL (2.83 mmol) methyl iodide was added, and stirring was continued for another 48 h. The solvent was evaporated in vacuo and residue was dissolved in water and extracted with ethyl acetate. The organic phase was washed with water and dried over anhydrous Na_2_SO_4_. TLC –PE:EtOAc = 5:1. The crude product was purified by column chromatography (70 g silica gel, phase PE:EtOAc = 6:1). After column chromatography, the product was washed with hot PE and dried in vacuo to give 0.24 g (45%) of pure **6** as a light yellow powder. M.p. 153–154 °C. IR (KBr), *υ*/cm^−1^: 3050, 3025 (*υc_H_(Ar)*) and 1538 (*υ*_C=N_). MS: (ESI^+^) 749 (M+1, 100%).

**2,2’,2’’-(1,3,5-triazine-2,4,6-triyltriimino)triphenol** (**11**). In 40 mL of glacial acetic acid, 1.50 g (8.13 mmol) cyanuric chloride (**1**) and 3.28 g (30.10 mmol) 2-aminophenol (**7**) were dissolved. The mixture was refluxed at 130 °C for 21 h (with an anhydrous CaCl_2_-tube over reflux condenser). After cooling, the mixture was poured into excess ice-cooled aqueous ammonia (pH ~ 9–10), and CHCl_3_ was added. Both liquid phases were filtered together through a shot filter, and the crude product was collected in the filter. The product was purified as follows: boiling with 25 mL CHCl_3_ and cooling. After filtering, the product was consequently washed with 5 mL cold CHCl_3_ and 2 mL cold MTBE. The obtained solid was dried in vacuo to give 1.00 g (31%) of pure **11** as a light pink powder. M.p. 236–237 °C. IR (KBr), *υ*/cm^−1^: 3409, 3396, 3388 (*υ*_NH_), 3280 (broad, *υ*_OH_), 3050 (*υc_H_(Ar)*) and 1510 (*υ*_C=N_). MS: (ESI^+^) 403 (M+1, 100%); (ESI^−^) 401 (M−1, 100%), 310 (M−C_6_H_4_OH, 37%).

**2,2’,2’’-(1,3,5-triazine-2,4,6-triyltriimino)tris(5-tert-butylphenol)** (**12**). In 40 mL glacial acetic acid, 1.00 g (5.42 mmol) cyanuric chloride (**1**) and 3.05 g (18.44 mmol) 2-amino-4-(tert-butyl)phenol (**8**) were dissolved. The mixture was refluxed at 130 °C for 3 h (with an anhydrous CaCl_2_-tube over reflux condenser). After cooling, the mixture was poured into excess ice-cooled aqueous ammonia (pH ~ 9–10). The crude product was extracted with CHCl_3_ (3x80 mL) and evaporated to dryness. TLC—DCM:*i*-PrOH = 100:1 and DCM:Et_2_O = 20:1. The product was first filtered through a short column (40 g silica, phase DCM:i-PrOH = 100:1) in order to remove part of impurities. After evaporation to dryness, a second purification by column chromatography was performed—75 g silica, phase 1 DCM:Et_2_O = 50:1 for impurities; phase 2 DCM:Et_2_O = 10:1 for the product. After column chromatography, the product was washed with hot heptane and dried in vacuo to give 1.937 g (62%) of pure **12** as white powder. Then, decomp. >175 °C without melting occurred. IR (KBr), *υ*/cm^−1^: 3383, 3282, 3189 (*υ*_NH_), 3280 (broad, *υ*_OH_), 3063 (*υc_H_(Ar)*) and 1507 (*υ*_C=N_). MS: (ESI^+^) 571 (M+1, 100%); (ESI^−^) 569 (M−1, 100%).

**2,2’,2’’-[1,3,5-triazine-2,4,6-triyltris(methylimino)]triphenol** (**13**). In 50 mL dry dioxane, 0.50 g (2.71 mmol) cyanuric chloride (**1**) and 1.035 g (8.41 mmol) 2-(methylamino)phenol (**9**) were dissolved 0. The mixture was gently refluxed at 105 °C for 18 h (with an anhydrous CaCl_2_-tube over reflux condenser). After cooling, the mixture was evaporated to dryness and dissolved in DCM. The organic phase was washed with 25% aq. ammonia and water, dried over MgSO_4_ and evaporated to dryness. TLC—DCM:i-PrOH = 100:1. The crude product was purified by column chromatography: 60 g silica, phase 1 DCM for impurities; phase 2 DCM:i-PrOH = 200:1 for the product. After column chromatography, the formed solid was recrystallized from 6 mL EtOH and finally washed with 10 mL hot MTBE. The product was dried in vacuo to give 0.106 g (9%) of pure **13** as a beige powder. M.p. 209–210 °C. IR (KBr), *υ*/cm^−1^: 3247 (broad, *υ*_OH_), 3069 (*υc_H_(Ar)*) and 1540 (*υ*_C=N_). MS: (ESI^+^) 445 (M+1, 100%); (ESI^−^) 443 (M−1, 100%).

**2,2’,2’’-(1,3,5-triazine-2,4,6-triyltriimino)tris(3-methylphenol)** (**14**). In 50 mL dry dioxane, 0.50 g (2.71 mmol) cyanuric chloride (**1**) and 1.035 g (8.41 mmol) 2-amino-3-methylphenol (**10**) were dissolved. The mixture was gently refluxed at 105 °C for 18 h (with anhydrous CaCl_2_-tube over reflux condenser). After cooling, 2 mL Et_3_N was added and evaporated to dryness. The crude product was dissolved in 100 mL CHCl_3_ and washed with saturated aq. NaCl and water, then dried over anhydrous Na_2_SO_4_. TLC—DCM:MTBE = 10:1. After evaporation to dryness, the crude product was purified by column chromatography: 40 g silica, phase 1 CHCl_3_ for impurities; phase 2 CHCl_3_:*i*-PrOH = 20:1 for the product. After column chromatography, the formed solid needed several crystallizations: 1) recrystallization from 40 mL *i*-PrOH; recrystallization from 20 mL acetone (in both cases the product is in the filtrate, not in the solid over the filter!). The collected filtrates from recrystallizations were evaporated to dryness and the formed solid was washed consequently with 50 mL hot MTBE and 50 mL hot PE. The product was dried in vacuo to give 0.309 g (26%) of pure **14** as a pale yellow powder. M.p. 286–287 °C. IR (KBr), *υ*/cm^−1^: 3359 (*υ*_NH_), 3274 (broad, *υ*_OH_), 3085 (*υc_H_(Ar)*) and 1504 (*υ*_C=N_). MS: (ESI^+^) 445 (M+1, 100%); (ESI^−^) 443 (M−1, 100%).

### 3.3. UV-Vis and Fluorescence Spectra

Spectral measurements were performed on a Jasco V-570 UV-Vis-NIR spectrophotometer (JASCO Corporation 2967-5, Ishikawa-cho, Hachioji, Tokyo, Japan), equipped with a thermostatic cell holder (using Huber MPC-K6 thermostat (Peter Huber Kaltemaschinenbau GmbH, D-77656 Offenburg, Germany) with precision 1 °C) and Jasco FP-6600 spectrofluorimeter (JASCO Corporation 2967-5, Ishikawa-cho, Hachioji, Tokyo, Japan) in spectral-grade solvents at 25 °C.

### 3.4. Computational Details

All calculations were performed by means of quantum chemical calculations at the density functional theory (DFT) level using Gaussian09 Rev. D.01 program package [85].

The geometries of all compounds have been fully optimized, and the corresponding transition states were localized using the B3LYP [86] functional with the 6–31+G(d,p) basis set [87] or using the M06-2X [88,89] functional with the TZVP [90] basis set. Solvent effect was included implicitly to the optimizations via the SMD [91] model with the built-in parameters for solvents (DMF). The nature of all critical points was confirmed by means of vibrational analysis.

The Δ*H*, Δ*S* and Δ*G* values were calculated at T = 298.15 K at the same level of theory including zero-point energy in the particular solvent environment (represented by relative permittivity) and vibrational, rotational and translational thermal energy corrections.

For prediction of the UV-Vis spectral data, the M06-2X [88,89] functional with the TZVP [90] basis set was used for the structure optimizations in the ground state. Solvent effect was included implicitly to the optimizations via the SMD [91] model with the built-in parameters for the solvent (CH_3_CN). In the excited state, CAM-B3LYP [92] with the same basis set was applied [93]. The TD-DFT method [94,95] was used for singlet excited-state optimizations, again without restrictions, applying tight optimization criteria and an ultrafine grid in the computation of two-electron integrals and their derivatives. Bearing in mind that M06-2X systematically underestimates the absorption band positions [96], the UV-Vis spectral data were predicted by the B3LYP [97] functional using the M06-2X optimized ground-state geometries.

### 3.5. Dynamic NMR Studies

^1^H and ^13^C spectra were recorded on a Bruker Avance II+ 600 spectrometer (Bruker BioSpin GmbH, Ettlingen, Germany) using BBO probe at 600.13 for ^1^H NMR and 150.92 MHz for ^13^C NMR with TMS as the internal standard for chemical shifts (*δ*, ppm). The spectra were recorded in steps of 5 K between 223 and 323 K (0.05M in 600 µL DMF-d7). Temperature calibration was performed with the builtin B-VT 3000 unit (it was checked and calibrated with methanol and ethylene glycol reference samples). ^1^H NMR spectra were acquired using a spectral width of 10 kHz, an acquisition time of 3.4 s, and 32 scans, zerofilled to 64k datapoints (0.15 Hz per point) and processed without apodization.

^1^H EXSY spectra (noesygpphzs Bruker pulse program) were recorded on a BBO probe typically in steps of 5 K between 223 and 258 K. The spectra were acquired using a spectral width of 4.2 kHz, 2048 × 256 complex time domain datapoints, mixing times in the range of 0.02 to 1.5 s and 2 scans in about 45 min. The spectra were zerofilled to 4096 × 4096 datapoints and processed with a shifted square sine bell apodization in both dimensions. The measurement details and processing of studied compounds can be found in the Supporting Information. Exchange rates were calculated from diagonal and crosspeak integrals using EXSYCalc (MestreLab Research S.L., Santiago de Compostela, Spain).

**Error analysis**: Usually, the presented errors in the activation parameters are the statistical errors based on scattering of the data points around the Eyring straight line only. The errors in this analysis are due to inaccuracies in both the calculated rate constants, ***k***, and the measured temperatures, ***T***, and are computed according to the error propagation equations of Binsch [97] and Heinzer and Oth [98]. The absolute error in temperature is assumed to be no more than ±0.5 K. The relative errors in ***k*** are estimated to be no more than ±10% at all temperatures according to the precision of the volume integration of peaks. The errors analysis was performed using a self-made computer program using the cited equations.

## 4. Conclusions

Seven OLED-relevant compounds (two known and five new) consisting of a 1,3,5-triazine core linked to various aromatic arms by amino group were synthesized and characterized. Conformational studies with respect to hindered rotation around the C(1,3,5-triazine)–N bond and C(Ph)–N bond were performed. The conformations of the studied compounds were elucidated on the basis of NMR and DFT studies. In the solution, two conformers were revealed and proved to originate from restricted rotation around the C(1,3,5-triazine)-N bond. The absorption and emission spectra of the studied compounds, as well as the HOMO–LUMO energy gap, limited the selection of some of these compounds for OLED applications. Further studies will show the applicability of these compounds in novel multilayer devices.

## Figures and Tables

**Table 1:** Spectroscopic properties of the studied derivatives determined for acetonitrile solutions.

Compound	λ^abs^_max_ [nm]	λ^em^_max_ [nm]	Δ ^a^ [eV]	φ_eff_^b^ [%]	λ_max_ [nm] (*f*) ^c^	Eg [eV] ^d^
**4**	300 338 353 358	386	0.19	0.33	344 (0.03)	4.09
**5**	280 300 338 353	386	0.25	0.90	330 (0.04)	4.32
**6**	280 300 338 353	391	0.28	5.75	334 (0.04)	4.37
**11**	290	-	-	-	280 (0.54)	4.97
**12**	295	-	-	-	285 (0.49)	4.91
**13**	282	-	-	-	267 (0.55)	5.32
**14**	282	-	-	-	254 (0.33)	5.08

^a^ Minimum Stokes shift. ^b^ Quantum efficiency in the solution. ^c^ B3LYP/TZVP//M06-2X/TZVP calculated UV-Vis parameters of the symmetric conformer. ^d^ HOMO-LUMO energy gap calculated by DFT.

**Table 2:** Populations of propeller and asymmetric conformers in DMF-d7 of studied compounds.

Comp.	R_1_	R_2_	Propeller, %, (^a^, ^b^)	Asymmetric, %, (^a^, ^b^)	(T, K)
**6**	CH_3_	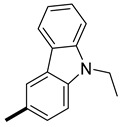	14 (0, 50)	86 (100, 50)	223
**5**	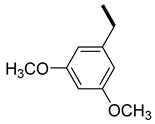	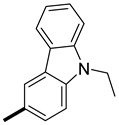	17 (100, 94)	83 (0, 6)	223
**13**	CH_3_	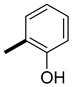	37 (26, 88)	63 (74, 12)	233
**11**	H	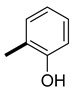	42 (21, 96)	58 (79, 4)	223
**12**	H	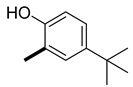	46 (18, 81)	54 (82, 19)	233
**14**	H	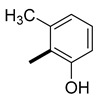	48 (11, 99)	52 (89, 1)	233
**4**	H	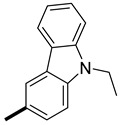	50 (1, 96)	50 (99, 4)	223

^a^ Calculated at SMD(DMF)//M062X/TZVP level of theory. ^b^ Calculated at SMD(DMF)//B3LYP/6-31+G(d,p) level of theory.

**Table 3:** Rotational barriers of propeller and asymmetric conformers in DMF-d7 of studied compounds.

Comp.	R_1_	R_2_	Experimental Rotational Barrier, ΔG^≠^(298K)	Theoretical Rotational Barrier, ΔG^≠^(298K) ^a^ (^b^)
**4**	H	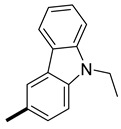	13.6 ± 0.1 (AS) ^c^ 13.9 ± 0.1 (SA)	14.5 (11.7) 12.5 (13.2)
**5**	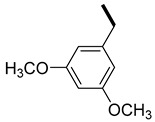	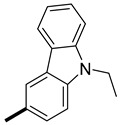	14.7 ± 0.1 (AS) 14.3 ± 0.1 (SA)	17.8 (12.9) 11.4 (14.1)
**6**	CH_3_	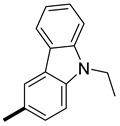	12.5 ± 0.1 (AS) 11.7 ± 0.1 (SA)	9.3 (11.1) 12.6 (11.1)
**11**	H	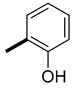	12.7 ± 0.1 (AS) 12.4 ± 0.1 (SA)	14.8 (14.4) 14.4 (15.9)
**12**	H	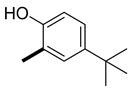	13.0 ± 0.1 (AS) 13.0 ± 0.1 (SA)	14.6 (15.0) 14.2 (14.7)
**13**	CH_3_	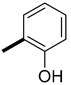	13.8 ± 0.1 (AS) 14.1 ± 0.1 (SA)	14.7 (13.9) 14.2 (13.5)
**14**	H	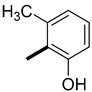	14.2 ± 0.1 (AS) 13.9 ± 0.1 (SA)	14.4 (15.7) 13.5 (16.9)

^a^ Calculated at SMD(DMF)//M062X/TZVP level of theory. ^b^ Calculated at SMD(DMF)//B3LYP/6-31+G(d,p) level of theory. ^c^ AS – asymmetric to symmetric process; SA – symmetric to asymmetric process.

## Data Availability

The data are available within the article and its Supplementary Materials.

## Supplementary Materials

The following supporting information can be downloaded at: https://www.mdpi.com/article/10.3390/molecules28031248/s1, Analytical data for synthesized compounds; ^1^H and ^13^C NMR spectra of the studies compounds; Dynamic NMR spectra (Figures S1–S21: VT ^1^H NMR spectra, ^1^H ROESY spectra and Eyring plots of rate constants; Tables S1–S21: Rate constants and thermodynamic parameters of exchange process); Figure S22: ^1^H,^15^N-HSQC spectrum of compound **11** in DMF-d7 at 243 K; Figure S23: ^1^H,^15^N-HMBC spectrum of compound **11** in DMF-d7 at 243 K; DFT calculations (Figures S24–S26: DFT calculated GS and TS structures, Tables S22–S24: Theoretical thermodynamic parameters).

## Sample Availability

Samples of the compounds **4**–**6** and **11**–**14** are available from the authors.
