# Supplementary material for: Structure and Conformational Mobility of OLED-Relevant 1,3,5-Triazine Derivatives
Source: Molecules. 2023 Jan 27;28(3):1248. doi: 10.3390/molecules28031248 (PMC9921695; doi:10.3390/molecules28031248)
Supplement: Supplementary file 1 [file molecules-28-01248-s001.zip › molecules-2127320-supplementary.pdf]

Structure and Conformational Mobility of OLED relevant 1,3,5-Triazine Derivatives

Georgi M. Dobrikov [a], Yana Nikolova [a], Ivaylo Slavchev [a], Miroslav Dangalov [a], Vera Deneva [a], Liudmil Antonov [b], Nikolay G. Vassilev [a,*]

[a] Institute of Organic Chemistry with Centre of Phytochemistry, Bulgarian Academy of Sciences, Acad. G. Bonchev Str., Bl. 9, 1113 Sofia, Bulgaria

[b] Institute of Electronics, Bulgarian Academy of Sciences, 72 Tsarigradsko chaussee blvd., 1784 Sofia, Bulgaria

* Corresponding author: Nikolay.Vassilev@orgchm.bas.bg

Table of Contents:

Analytical data for synthesised compounds 2

^{1}H and ^{13}C NMR spectra of the studies compounds 8

Dynamic NMR spectra 29

^{15}N spectra of compound **11** in DMF-d7 at 243K 57

DFT calculations 59

References 94

Analytical data for synthesised compounds

Analytical data for compound *N*,*N*',*N*''-tris(9-ethyl-9*H*-carbazol-3-yl)-1,3,5-triazine-2,4,6-triamine (4):

^{1}H NMR (600 MHz, DMF-d_7, 223 K, ppm) δ: 50% : 50% mixture of conformers; signals for *propeller* conformer: 10.29 (s, 3H, N**H**), 9.15 (s, 3H, carbazole-H4), 8.18 (d, J = 7.8 Hz, 3H, carbazole-H5), 7.85-7.80 (m, 3H, carbazole-H2), 7.73-7.68 (m, 6H, carbazole-H1&H8), 7.53-7.49 (m, 3H, carbazole-H7), 7.26-7.22 (m, 3H, carbazole-H6), 4.55 (bs, 6H, C$\mathbf{H_2}$), 1.33 (t, J = 6.8 Hz, 9H, C$\mathbf{H_3}$); ^{13}C NMR (151 MHz, DMF-d_7, 223 K) δ 164.4 (triazine-^{4}C), 140.3 (carbazole-^{4}C), 135.7 (carbazole-^{4}C), 132.9 (carbazole-^{4}C), 125.8 (carbazole-C6), 122.7 (carbazole-^{4}C), 122.4 (carbazole-^{4}C), 120.6 (carbazole-C5), 119.4 (carbazole-C2), 118.4 (carbazole-C6), 111.7 (carbazole-4C), 109.1 (carbazole-C1&C8), 37.1 (**C**H_2), 13.7 (**C**H_3); signals for *asymmetric* conformer: ^{1}H NMR (600 MHz, DMF-d_7, 223 K) δ: 10.30 (s, 1H, N**H**), 9.95 (s, 1H, N**H**), 9.89 (s, 1H, N**H**), 9.12 (s, 1H, carbazole-H4), 8.70 (s, 1H, carbazole-H4), 8.59 (s, 1H, carbazole-H4), 8.17 (d, J = 7.8 Hz, 2H, carbazole-H5), 7.95 (d, J = 8.7 Hz, 1H, carbazole-H1), 7.93 (d, J = 8.7 Hz, 1H, carbazole-H1), 7.85-7.79 (m, 2H, carbazole-H5&H1), 7.74-7.68 (m, 3H, carbazole-H8), 7.65 (d, J=8.7 Hz, 1 H, carbazole-H2), 7.62 (d, J=8.7 Hz, 1 H, carbazole-H2), 7.59 (d, J=8.7 Hz, 1 H, carbazole-H2), 7.51 (t, J = 7.6 Hz, 1H, carbazole-H6), 7.45 (t, J = 7.6 Hz, 1H, carbazole-H6), 7.42 (t, J = 7.6 Hz, 1H, carbazole-H6), 7.23 (t, J = 7.6 Hz, 1H, carbazole-H7), 7.00 (t, J = 7.6 Hz, 1H, carbazole-H7), 6.92 (t, J = 7.6 Hz, 1H, carbazole-H7), 4.55 (bs, 2H, C$\mathbf{H_2}$), 4.48 (bs, 2H, C$\mathbf{H_2}$), 4.45 (bs, 2H, C$\mathbf{H_2}$), 1.33 (t, J = 7.4 Hz, 3H, C$\mathbf{H_3}$)1.26 (t, J = 7.4 Hz, 3H), 1.25 (t, J = 7.4 Hz, 3H); ^{13}C NMR (151 MHz, DMF-$d_{7,}$ 223K) δ 165.3 (triazine-^{4}C), 165.2(triazine-^{4}C), 164.6 (triazine-^{4}C), 140.2 (carbazole-^{4}C), 136.2 (carbazole-^{4}C), 135.9 (carbazole-^{4}C), 135.6 (carbazole-^{4}C), 133.0 (carbazole-^{4}C), 132.5 (carbazole-^{4}C), 132.3 (carbazole-^{4}C), 125.8 (carbazole-C6), 122.5 (carbazole-^{4}C), 122.4 (carbazole-^{4}C), 121.6 (carbazole-C5), 120.5 (carbazole-C1), 118.3 (carbazole-C7), 113.4 (carbazole-C4), 112.5 (carbazole-C4), 111.7 (carbazole-C4), 109.2 (carbazole-C1), 109.1 (carbazole- C8), 37.2 (**C**H_2), 13.7 (**C**H_3).

^{1}H NMR (600 MHz, DMSO-d_6, 393 K, ppm) δ: averaged signals due to fast exchange: 8.58 (s, 1H, NH), 8.48 (d, *J* = 1.9 Hz, 1H, carbazole-H4), 7.94 (d, *J* = 7.7 Hz, 1H, carbazole-H5), 7.78 (dd, *J* = 2.1, 8.7 Hz, 1H, carbazole-H2), 7.51 (d, *J* = 8.2 Hz, 1H, carbazole-H8), 7.41 (d, *J* = 8.5 Hz, 1H, carbazole-H1), 7.40 (ddd, *J* = 1.2, 7.0, 8.2 Hz, 1H, carbazole-H7), 7.05 (ddd, *J* = 0.6, 7.1, 7.7 Hz, 1H, carbazole-H6), 4.37 (q, *J* = 7.2 Hz, 2H, C$\mathbf{H_2}$), 1.35 (t, *J* = 7.1 Hz, 3H, C$\mathbf{H_3}$). ^{13}C NMR (151 MHz, DMSO-d6, 393 K) δ 164.48 (triazine-^{4}C), 139.71 and 135.72 (carbazole-C8a and carbazole-C9a), 131.44 (carbazole-C3), 124.69 (carbazole-C7), 122.01 and 121.79 (carbazole-C4a and carbazole-C4b), 120.60 (carbazole-C2), 119.53 (carbazole-C4), 117.51 (carbazole-C6), 112.69 (carbazole-C4), 108.15 (carbazole-C8), 107.76 (carbazole-C1), 36.47 (**C**H_2), 12.69 (s, **C**H_3).

Analytical data for compound *N*,*N*',*N*''-tris(3,5-dimethoxybenzyl)-*N*,*N*',*N*''-tris(9-ethyl-9*H*-carbazol-3-yl)-1,3,5-triazine-2,4,6-triamine (5):

^{1}H NMR (600 MHz, DMF-d_7, 223 K, ppm) δ: 17% : 83% mixture of conformers; signals for major *asymmetric* conformer: 8.45 (s, 1H, carbazole-H4), 8.30 (d, J = 7.7 Hz, 1H, carbazole-H5), 8.29 (s, 1H, carbazole-H4), 8.28 (d, J = 7.7 Hz, 1H, carbazole-H5), 8.20 (s, 1H, carbazole-H4), 7.98 (d, J = 7.7 Hz, 1H, carbazole-H5), 7.68-7.62 (m, 3H, carbazole-H2 & carbazole-H8), 7.57-7.52 (m, 3H, carbazole-H2 & carbazole-H8), 7.47-7.41 (m, 3H, carbazole-H7), 7.20 (dd, J = 7.1, 7.7 Hz, 1H, carbazole-H6), 7.17 (dd, J = 7.1, 7.7 Hz, 1H, carbazole-H6), 7.15 (dd, J = 7.1, 7.7 Hz, 1H, carbazole-H6), 7.09 (d, J = 8.7 Hz, 2H, carbazole-H1), 7.07 (d, J = 8.7 Hz, 1H, carbazole-H1), 6.57 (d, J = 1.9 Hz, 2H, 3,5-dimethoxybenxene-H2), 6.55 (d, J = 1.9 Hz, 2H, 3,5-dimethoxybenxene-H2), 6.40 (t, J = 1.9 Hz, 1H, 3,5-dimethoxybenxene-H4), 6.36 (t, J = 1.9 Hz, 1H, 3,5-dimethoxybenxene-H4), 6.31 (t, J = 1.9 Hz, 1H, 3,5-dimethoxybenxene-H4), 66.19 (d, J = 1.9 Hz, 2H, 3,5-dimethoxybenxene-H2), 5.49 (bs, 2H, benzyl-C$\mathbf{H_2}$), 5.46 (bs, 2H, benzyl-C$\mathbf{H_2}$), 5.05 (bs, 2H, benzyl-C$\mathbf{H_2}$), 4.47 (bs, 4H, -C$\mathbf{H_2}$), 3.99 (bs, 2H, -C$\mathbf{H_2}$), 3.73 (s, 6H, -O**Me**),

3.72 (s, 6H, -O**Me**), 3.57 (s, 6H, -O**Me**), 1.28 (t, J = 7.1 Hz, 3H, -C**H**$_3$), 0.88 (t, J = 7.1 Hz, 3H, -C**H**$_3$), 0.87 (t, J = 7.1 Hz, 3H, -C**H**$_3$). ^{13}C NMR (151 MHz, DMF-d$_7$, 223K) δ 166.4 (triazine-^{4}C), 166.3 (triazine-^{4}C), 166.3 (triazine-^{4}C), 161.0 (3,5-dimethoxybenxene-^{4}C3), 160.9 (3,5-dimethoxybenxene-^{4}C3), 160.8 (3,5-dimethoxybenxene-^{4}C3), 142.5 (3,5-dimethoxybenxene-^{4}C1), 142.4 (3,5-dimethoxybenxene-^{4}C1), 140.4 (carbazole-^{4}C), 140.2 (carbazole-^{4}C), 137.7 (carbazole-^{4}C), 137.7 (carbazole-^{4}C), 137.1 (carbazole-^{4}C), 135.1 (carbazole-^{4}C), 134.9 (carbazole-^{4}C), 126.4 (carbazole-C7), 126.2 (carbazole-C7), 126.1 (carbazole-C2), 125.9 (carbazole-C2), 122.8 (carbazole-^{4}C), 122.5 (carbazole-^{4}C), 122.2 (carbazole-C5), 122.2 (carbazole-C5), 120.8 (carbazole-C4), 119.6 (carbazole-C4), 118.9 (carbazole-C4), 118.8 (carbazole-C6), 118.7 (carbazole-C6), 109.4 (carbazole-C8), 108.8 (carbazole-C8), 108.1 (carbazole-C1), 105.9 (3,5-dimethoxybenxene-C2), 105.6 (3,5-dimethoxybenxene-C2), 105.3 (3,5-dimethoxybenxene-C2), 97.8 (3,5-dimethoxybenxene-C4), 97.7 (3,5-dimethoxybenxene-C4), 97.6 (3,5-dimethoxybenxene-C4), 54.0 (-O**Me**), 54.9 (-O**Me**), 54.8 (-O**Me**), 53.4 (benzyl-C**H**$_2$), 53.2 (benzyl-C**H**$_2$), 52.8 (benzyl-C**H**$_2$), 37.35 (**C**H$_2$), 37.02 (**C**H$_2$), 13.85 (**C**H$_3$), 13.67 (**C**H$_3$). Minor ^{1}H NMR (600 MHz, DMF-d$_7$, 223K) δ 8.33 (s, 3H, carbazole-H4), 7.91 (d, J = 7.7 Hz, 3H, carbazole-H5), 7.67 (d, J = 8.8 Hz, 3H, carbazole-H2), 7.55-7.52 (m, 3H, carbazole-H8), 7.44-7.41 (m, 3H, carbazole-H7), 7.18 (dd, J = 7.1, 7.7 Hz, 3H, carbazole-H6), 7.08 (d, J = 8.7 Hz, 3H, carbazole-H1), 6.34 (t, J = 1.9 Hz, 6H, 3,5-dimethoxybenxene-H4), .26 (d, J = 1.9 Hz, 3H, 3,5-dimethoxybenxene-H2), 5.09 (bs, 6H, benzyl-C**H**$_2$), 3.99 (bs, 6H, -C**H**$_2$), 3.61 (s, 18H, -O**Me**), 1.26 (t, J = 7.1 Hz, 9H, -C**H**$_3$). ^{13}C NMR (151 MHz, DMF-d$_7$, 223K) δ 166.1 (triazine-^{4}C), 161.0 (3,5-dimethoxybenxene-^{4}C3), 142.4 (3,5-dimethoxybenxene-^{4}C1), 126.2 (carbazole-C7), 126.1 (carbazole-C2), 122.6 (carbazole-^{4}C), 122.2 (carbazole-C5), 121.0 (carbazole-C4), 118.9 (carbazole-C6),108.8 (carbazole-C8), 108.1 (carbazole-C1), 106.0 (3,5-dimethoxybenxene-C2), 97.6 (3,5-dimethoxybenxene-C4), 54.9 (-O**Me**), 52.6 (benzyl-C**H**$_2$), 37.0 (**C**H$_2$), 13.9 (**C**H$_3$).

^{1}H NMR (600 MHz, DMSO-d_6, 403 K, ppm) δ: averaged signals due to fast exchange: 7.90 (d, J = 1.1 Hz, 1H, carbazole-H4), 7.86 (d, J = 7.7 Hz, 1H, carbazole-H5), 7.43 (d, J = 8.2 Hz, 1H, carbazole-H8), 7.38 (ddd, J = 1.1, 7.1, 8.1 Hz, 1H, carbazole-H7), 7.25 (dd, J = 2.0, 8.7 Hz, 1H, carbazole-H2), 7.12 (d, J = 8.8 Hz, 1H, carbazole-H1), 7.09 (ddd, J = 0.9, 7.0, 7.9 Hz, 1H, carbazole-H6), 6.30 (d, J = 2.1 Hz, 2H, 3,5-dimethoxybenxene-H2 and 3,5-dimethoxybenxene-H6), 6.27 (t, J = 2.2 Hz, 1H, 3,5-dimethoxybenxene-H4), 5.06 (s, 2H, benzyl-C**H**$_2$), 4.16 (q, J = 7.0 Hz, 2H, C**H**$_2$), 3.59 (s, 6H, O**Me**), 1.19 (t, J = 7.1 Hz, 3H, C**H**$_3$). ^{13}C NMR (151 MHz, DMSO-d6, 403K) δ 165.44 (triazine-^{4}C), 159.89 (3,5-dimethoxybenxene-^{4}C3 and 3,5-dimethoxybenxene-^{4}C5), 140.99 (carbazole-C8a), 139.57 (3,5-dimethoxybenxene-^{4}C1), 136.80 (carbazole-C3), 134.61 (carbazole-C8a), 125.06 (carbazole-C2), 124.65 (carbazole-C7), 121.89 and 121.47 (carbazole-C4a and carbazole-C4b), 119.23 (carbazole-C5), 117.81 (carbazole-C4), 117.73 (carbazole-C6), 108.15 (carbazole-C8), 107.27 (carbazole-C1), 105.64 (3,5-dimethoxybenxene-C2 and 3,5-dimethoxybenxene-C6), 98.52 (3,5-dimethoxybenxene-^{4}C4), 54.41 (O**Me**), 52.52 (benzyl-C**H**$_2$), 36.32 (**C**H$_2$), 12.42 (**C**H$_3$).

Analytical data for compound *N*,*N*',*N*''-tris(9-ethyl-9*H*-carbazol-3-yl)-*N*,*N*',*N*''-trimethyl-1,3,5-triazine-2,4,6-triamine (6):

^{1}H NMR (600 MHz, DMF-d_7, 223 K, ppm) δ: 14% : 86% mixture of conformers; signals for major *asymmetric* conformer: 8.47 (s, 1H, carbazole-H4), 8.37 (d, J = 7.7 Hz, 1H, carbazole-H5), 8.33 (d, J = 7.7 Hz, 1H, carbazole-H5), 8.27 (d, J = 7.7 Hz, 1H, carbazole-H5), 8.21 (s, 1H, carbazole-H4), 8.08 (s, 1H, carbazole-H4), 7.80 (d, J = 8.8 Hz, 1H, carbazole-H1), 7.74 (d, J = 8.5 Hz, 1H, carbazole-H8), 7.69 (d, J = 8.8 Hz, 1H, carbazole-H2), 7.62 (d, J = 8.5 Hz, 1H, carbazole-H8), 7.61 (d, J = 8.8 Hz, 1H, carbazole-H2), 7.58 (d, J = 8.5 Hz, 1H, carbazole-H8), 7.53-7.43 (m, 3H, carbazole-H7), 7.50 (d, J = 8.8 Hz, 1H, carbazole-H2), 7.30-7.20 (m, 3H, carbzole-H6), 7.18 (d, J = 8.7 Hz, 1H, carbazole-H1), 7.11 (d, J = 8.7 Hz, 1H, carbazole-H1), 4.58 (bs, 2H, C**H**$_2$), 4.08 (bs, 4H, C**H**$_2$), 3.75 (s, 3H, NC**H**$_3$), 3.69 (s, 3H, NC**H**$_3$), 3.20 (s, 3H, NC**H**$_3$), 1.36 (t, J = 7.6 Hz, 3H, C**H**$_3$), 0.96 (t, J = 7.6 Hz, 3H, C**H**$_3$), 0.94 (t, J = 7.6 Hz, 3H,

C$\mathbf{H_3}$). ^{13}C NMR (151 MHz, DMF-d_7, 223K) δ: 165.9 (triazine-^{4}C), 165.6 (triazine-^{4}C), 165.3 (triazine-^{4}C), 140.5 (carbazole-^{4}C), 140.2 (carbazole-^{4}C), 140.2 (carbazole-^{4}C), 137.8 (carbazole-^{4}C), 137.3 (carbazole-^{4}C), 137.0 (carbazole-^{4}C), 136.9 (carbazole-^{4}C), 136.7 (carbazole-^{4}C), 125.9 (carbazole-C2), 125.6 (carbazole-C7 & carbazole-C2), 125.5 (carbazole-C2), 122.9 (carbazole-^{4}C), 122.8 (carbazole-^{4}C), 122.7 (carbazole-^{4}C), 122.6 (carbazole-^{4}C), 122.2 carbazole-^{4}C (), 122.1 (carbazole-^{4}C), 120.7 (carbazole-C5), 118.9 (carbazole-C4), 118.8 (carbzole-C6), 118.5 (carbzole-C6), 117.9 (carbazole-C4), 117.6 (carbazole-C4), 109.3 (carbazole-C8), 109.1 (carbazole-C8), 108.7 (carbazole-C1), 107.9 (carbazole-C1), 107.9 (carbazole-C1), 38.1 (N$\mathbf{C}H_3$), 37.9 (N$\mathbf{C}H_3$), 37.4 (N$\mathbf{C}H_3$), 37.2 ($\mathbf{C}H_2$), 36.8 ($\mathbf{C}H_2$), 13.7 ($\mathbf{C}H_3$), 13.5 ($\mathbf{C}H_3$), 13.4 ($\mathbf{C}H_3$); signals for minor *propeller* conformer: ^{1}H NMR (600 MHz, DMF-d_7, 223K) δ: 8.35 (s, 3H, carbazole-H4), 8.33 (d, J = 7.7 Hz, 3H, carbazole-H5), 7.72 (d, J = 8.7 Hz, 3H, carbazole-H8), 7.58 (d, J = 8.5 Hz, 3H, carbazole-H2), 7.53 (d, J = 8.5 Hz, 3H, carbazole-H1), 4.58 (bs, 6H, C$\mathbf{H_2}$), 3.26 (s, 9H), 1.34 (t, J = 7.6 Hz, 9H, C$\mathbf{H_3}$); ^{13}C NMR (151 MHz, DMF-d_7, 223K) 165.8 (triazine-^{4}C), 140.4 (carbazole-^{4}C), 137.7 (carbazole-^{4}C), 122.9 (carbazole-^{4}C), 122.6 (carbazole-^{4}C), 120.7 (carbazole-C5), 118.9 (carbazole-C4), 108.7 (carbazole-C1), 37.3 (N$\mathbf{C}H_3$), 13.7 ($\mathbf{C}H_3$).
^{1}H NMR (600 MHz, DMSO-d_6, 373 K, ppm) δ: averaged signals due to fast exchange: 8.02 (d, *J* = 7.7 Hz, 1H, carbazole-H5), 7.99 (d, *J* = 1.9 Hz, 1H, carbazole-H4), 7.48 (td, *J* = 0.8, 8.3 Hz, 1H, carbazole-H8), 7.41 (ddd, *J* = 1.2, 7.1, 8.2 Hz, 1H, carbazole-H7), 7.38 (dd, *J* = 2.1, 8.7 Hz, 1H, carbazole-H2), 7.22 (d, *J* = 8.6 Hz, 1H, carbazole-H1), 7.12 (ddd, *J* = 0.9, 7.0, 7.9 Hz, 1H, carbazole-H6), 4.21 (q, *J* = 7.1 Hz, 2H, C$\mathbf{H_2}$), 3.43 (s, 3H, NC$\mathbf{H_3}$), 1.21 (t, *J* = 7.1 Hz, 3H, C$\mathbf{H_3}$); ^{13}C NMR (151 MHz, DMSO-d6, 373K) δ 165.17 (triazine-^{4}C), 139.64 (carbazole-C8a), 136.66 (carbazole-C9a), 136.34 (carbazole-^{3}C), 124.89 (carbazole-C7), 124.41 (carbazole-C2), 121.98 and 121.56 (carbazole-C4a and carbazole-C4b), 119.59 (s, carbazole-C5), 117.92 (carbazole-C6), 117.13 (carbazole-C4), 108.38 (carbazole-C8), 107.52 (carbazole-C1), 37.16 (N$\mathbf{C}H_3$), 36.46 ($\mathbf{C}H_2$), 12.78 ($\mathbf{C}H_3$).

Analytical data for compound 2,2',2''-(1,3,5-triazine-2,4,6-triyltriimino)triphenol (11):
^{1}H NMR (600 MHz, DMF-d_7, 223 K, ppm) δ: 42% : 58% mixture of conformers; signals for major *asymmetric* conformer: 10.71 (s, 3H, -O**H**), 8.96 (s, 3H, -N**H**), 7.98 (d, J = 7.8 Hz, 3H, 2-aminophenol-H3), 6.97 (m, 6H, 2-aminophenol-H6 & 2-aminophenol-H5), 6.85 (t, J = 7.7 Hz, 3H, 2-aminophenol-H4). ^{13}C NMR (151 MHz, DMF-d_7, 223K) δ 163.9 (triazine-^{4}C), 148.8 (2-aminophenol-^{4}C1), 127.2 (2-aminophenol-^{4}C2), 124.6 (2-aminophenol-C6), 123.6 (2-aminophenol-C3), 119.3 (2-aminophenol-C5), 116.1 (2-aminophenol-C4), ; signals for minor *propeller* conformer: ^{1}H NMR (600 MHz, DMF-d_7 223 K) δ: 10.84 (s, 1H, -O**H**), 10.63 (s, 1H, -O**H**), 10.55 (s, 1H, -O**H**), 9.05 (s, 1H, -N**H**), 8.54 (s, 1H, -N**H**), 8.50 (s, 1H, -N**H**), 8.08 (d, J = 7.8 Hz, 1H, 2-aminophenol-H3), 7.87 (d, J = 7.8 Hz, 1H, 2-aminophenol-H3), 7.82 (d, J = 7.8 Hz, 1H, 2-aminophenol-H3), 6.97 (m, 6H, 2-aminophenol-H6 & 2-aminophenol-H5), 6.85 (t, J = 7.7 Hz, 1H, 2-aminophenol-H4), 6.79 (t, J = 7.7 Hz, 1H, 2-aminophenol-H4), 6.75 (t, J = 7.7 Hz, 1H, 2-aminophenol-H4). ^{13}C NMR (151 MHz, DMF-d_7, 223K) δ 164.6 (triazine-^{4}C), 164.5 (triazine-^{4}C), 164.2 (triazine-^{4}C), 149.6 (2-aminophenol-^{4}C1), 148.9 (2-aminophenol-^{4}C1), 147.97 (2-aminophenol-^{4}C1), 127.8 (2-aminophenol-^{4}C2), 126.8 (2-aminophenol-^{4}C2), 126.6 (2-aminophenol-^{4}C2), 124.8 (2-aminophenol-C6), 124.4 (2-aminophenol-C3), 124.2 (2-aminophenol-C6), 123.8 (2-aminophenol-C6), 123.2 (2-aminophenol-C3), 122.1 (2-aminophenol-C3), 119.4 (2-aminophenol-C5), 118.9 (2-aminophenol-C5), 116.0 (2-aminophenol-C4), 115.5 (2-aminophenol-C4), 115.2 (2-aminophenol-C4)
^{1}H NMR (600 MHz, DMSO-d_6, 353 K, ppm) δ: averaged signals due to fast exchange: 9.55 (bs, 1H, OH), 8.01 (s, 1H, NH), 7.82 (dd, *J* = 1.4, 8.0 Hz, 1H, 2-aminophenol-H3), 6.93 (ddd, *J* = 1.5, 7.3, 7.9 Hz, 1H, 2-aminophenol-H5), 6.88 (dd, *J* = 1.5, 8.0 Hz, 1H, 2-aminophenol-H6), 6.77 (ddd, *J* = 1.4, 7.4, 7.8 Hz, 1H, 2-aminophenol-H4). ^{13}C NMR (151 MHz, DMSO-d6, 353K) δ 163.76 (triazine-^{4}C), 147.68 (2-aminophenol-^{4}C1), 126.62 (2-aminophenol-^{4}C2), 123.47 (2-

aminophenol-C5), 122.53 (2-aminophenol-C3), 118.66 (2-aminophenol-C4), 115.46 (2-aminophenol-C6).

Analytical data for compound 2,2',2''-(1,3,5-triazine-2,4,6-triyltriimino)tris(5-*tert*-butylphenol) (12):

^{1}H NMR (600 MHz, DMF-d_7, 233 K, ppm) δ: 46% : 54% mixture of conformers; signals for major *asymmetric* conformer: 10.17 (s, 3H, -O**H**), 9.41 (s, 3H, -N**H**), 7.66 (d, J = 2.3 Hz, 3H, 2-amino-5-(tert-butyl)phenol-H6), 7.12 (dd, J = 2.3, 8.5 Hz, 3H, 2-amino-5-(tert-butyl)phenol-H4), 6.91 (d, J = 8.5 Hz, 3H, 2-amino-5-(tert-butyl)phenol-H3), 1.25 (s, 27H, *t*-Bu-C$\mathbf{H_3}$). ^{13}C NMR (151 MHz, DMF-d_7, 233K) δ 163.9 (triazine-^{4}C), 147.6 (2-amino-5-(tert-butyl)phenol-^{4}C2), 142.3 (2-amino-5-(tert-butyl)phenol-^{4}C5), 126.6 (2-amino-5-(tert-butyl)phenol-^{4}C1), 122.7 (2-amino-5-(tert-butyl)phenol-C4), 121.5 (2-amino-5-(tert-butyl)phenol-C6), 117.2 (2-amino-5-(tert-butyl)phenol-C3), 31.3 (*t*-Bu-**C**H_3); ; signals for minor *propeller* conformer: ^{1}H NMR (600 MHz, DMF-d_7 233 K) δ: 10.39 (s, 1H, -O**H**), 10.11 (s, 1H, -O**H**), 9.96 (s, 3H, -O**H**), 9.41 (s, 1H, -N**H**), 9.10 (s, 1H, -N**H**), 8.94 (s, 1H, -N**H**), 7.69 (d, J = 2.3 Hz, 1H, 2-amino-5-(tert-butyl)phenol-H6), 7.47 (d, J = 2.3 Hz, 1H, 2-amino-5-(tert-butyl)phenol-H6), 7.40 (d, J = 2.3 Hz, 1H, 2-amino-5-(tert-butyl)phenol-H6), 7.08 (dd, J = 2.3, 8.5 Hz, 1H, 2-amino-5-(tert-butyl)phenol-H4), 7.06 (dd, J = 2.3, 8.5 Hz, 1H, 2-amino-5-(tert-butyl)phenol-H4), 7.00 (dd, J = 2.3, 8.5 Hz, 1H, 2-amino-5-(tert-butyl)phenol-H4), 6.90 (d, J = 8.5 Hz, 1H, 2-amino-5-(tert-butyl)phenol-H3), 6.78 (d, J = 8.5 Hz, 1H, 2-amino-5-(tert-butyl)phenol-H3), 6.77 (d, J = 8.5 Hz, 1H, 2-amino-5-(tert-butyl)phenol-H3), 1.26 (s, 9H, *t*-Bu-C$\mathbf{H_3}$), 1.15 (s, 9H, *t*-Bu-C$\mathbf{H_3}$), 1.12 (s, 9H, *t*-Bu-C$\mathbf{H_3}$). ^{13}C NMR (151 MHz, DMF-d_7, 233K) δ 164.9 (triazine-^{4}C), 164.6 (triazine-^{4}C), 164.2 (triazine-^{4}C), 148.7 (2-amino-5-(tert-butyl)phenol-^{4}C2), 147.8 (2-amino-5-(tert-butyl)phenol-^{4}C2), 146.8 (2-amino-5-(tert-butyl)phenol-^{4}C2), 142.4 (2-amino-5-(tert-butyl)phenol-^{4}C5), 141.9 (2-amino-5-(tert-butyl)phenol-^{4}C5), 127.4 (2-amino-5-(tert-butyl)phenol-^{4}C1), 126.0 (2-amino-5-(tert-butyl)phenol-^{4}C1), 125.5 (2-amino-5-(tert-butyl)phenol-^{4}C1), 123.3 (2-amino-5-(tert-butyl)phenol-C4), 122.6 (2-amino-5-(tert-butyl)phenol-C6), 122.4 (2-amino-5-(tert-butyl)phenol-C4), 121.9 (2-amino-5-(tert-butyl)phenol-C4), 121.5 (2-amino-5-(tert-butyl)phenol-C6), 120.5 (2-amino-5-(tert-butyl)phenol-C6), 117.5 (2-amino-5-(tert-butyl)phenol-C3), 116.8 (2-amino-5-(tert-butyl)phenol-C3), 116.4 (2-amino-5-(tert-butyl)phenol-C3), 31.3 (*t*-Bu-**C**H_3), 31.3 (*t*-Bu-**C**H_3), 31.3 (*t*-Bu-**C**H_3).

^{1}H NMR (600 MHz, DMSO-d_6, 373 K, ppm) δ: averaged signals due to fast exchange: 9.17 (bs, 1H, OH), 8.07 (bs, 1H, NH), 7.63 (d, *J* = 2.3 Hz, 1H, 2-amino-5-(tert-butyl)phenol-H6), 6.96 (dd, *J* = 2.5, 8.4 Hz, 1H, 2-amino-5-(tert-butyl)phenol-H4), 6.78 (d, *J* = 8.4 Hz, 1H, 2-amino-5-(tert-butyl)phenol-H3), 1.24 (s, 9H, *t*-Bu-C$\mathbf{H_3}$). ^{13}C NMR (151 MHz, DMSO-d6, 373K) δ 163.62 (triazine-^{4}C), 145.77 (2-amino-5-(tert-butyl)phenol-^{4}C1), 141.38 (2-amino-5-(tert-butyl)phenol-^{4}C4), 125.70 (2-amino-5-(tert-butyl)phenol-^{4}C2), 120.56 (2-amino-5-(tert-butyl)phenol-^{4}C5), 119.94 (2-amino-5-(tert-butyl)phenol-^{4}C3), 115.61 (2-amino-5-(tert-butyl)phenol-^{4}C6), 33.20 (C), 30.83 (*t*-Bu-**C**H_3).

Analytical data for compound 2,2',2''-[1,3,5-triazine-2,4,6-triyltris(methylimino)]triphenol (13):

^{1}H NMR (600 MHz, DMF-d_7, 223 K, ppm) δ: 37% : 63% mixture of conformers; signals for major *asymmetric* conformer: ; signals for minor *propeller* conformer: ^{1}H NMR (600 MHz, DMF-d_7 223 K) δ: 10.05 (s, 3H, -O**H**), 7.25 (dd, J = 1.4, 7.7 Hz, 3H, 2-aminophenol-H3), 7.16 (ddd, J = 1.2, 7.7, 7.9 Hz, 3H, 2-aminophenol-H4), 7.01 (dd, J = 1.2, 8.1 Hz, 3H, 2-aminophenol-H6), 6.88 (ddd, J = 1.4, 7.9, 8.1 Hz, 3H, 2-aminophenol-H5), 3.04 (s, 9H, -C$\mathbf{H_3}$). ^{13}C NMR (151 MHz, DMF-d_7, 223K) δ 165.47 (triazine-^{4}C), 153.59 (2-aminophenol-^{4}C1), 132.24 (2-aminophenol-^{4}C2), 129.26, 127.80 (2-aminophenol-C4), 127.61 (2-aminophenol-C3), 119.16 (2-aminophenol-C5), 116.78 (2-aminophenol-C6), 35.82 (-N**C**H_3); signals for minor *propeller* conformer: ^{1}H NMR (600 MHz, DMF-d_7, 223K) δ: 7.35 (dd, J = 1.4, 7.7 Hz, 1H, 2-

aminophenol-H3), 7.20 (ddd, J = 1.2, 7.7, 7.9 Hz, 1H, 2-aminophenol-H4), 7.18 (d, J=7.7 Hz, 1 H, 2-aminophenol-H3), 7.07 (d, J=7.7 Hz, 1 H, 2-aminophenol-H3), 7.05 (dd, J = 1.2, 8.1 Hz, 1H, 2-aminophenol-H6), 7.00-6.95 (m, 2H, 2-aminophenol-H4), 6.92 (ddd, J = 1.4, 7.9, 8.1 Hz, 1H, 2-aminophenol-H5), 6.76 (dd, J = 1.2, 8.1 Hz, 1H, 2-aminophenol-H6), 6.74 (dd, J = 1.2, 8.1 Hz, 1H, 2-aminophenol-H6), 6.71 (ddd, J = 1.4, 7.9, 8.1 Hz, 1H, 2-aminophenol-H5), 6.67 (ddd, J = 1.4, 7.9, 8.1 Hz, 1H, 2-aminophenol-H5). ^{13}C NMR (151 MHz, DMF-d$_7$, 223K) δ 165.65 (triazine-^{4}C), 165.15 (triazine-^{4}C), 164.90 (triazine-^{4}C), 153.74 (2-aminophenol-^{4}C1), 152.84 (2-aminophenol-^{4}C1), 152.82 (2-aminophenol-^{4}C1), 132.36 (2-aminophenol-^{4}C2), 132.24 (2-aminophenol-^{4}C2), 132.06 (2-aminophenol-^{4}C2), 129.49 (2-aminophenol-C4), 128.22 (2-aminophenol-C3), 127.80 (2-aminophenol-C3), 126.88 (2-aminophenol-C4), 119.16 (2-aminophenol-C5), 116.69 (2-aminophenol-C6), 37.02 (-N**C**H$_3$), 36.41 (-N**C**H$_3$), 36.28 (-N**C**H$_3$)
^{1}H NMR (600 MHz, DMSO-*d$_6$*, 343 K, ppm) δ: averaged signals due to fast exchange: 7.07 (d, *J* = 7.6 Hz, 1H, 2-aminophenol-H3), 7.02 (t, *J* = 7.1 Hz, 1H, 2-aminophenol-H5), 6.81 (d, *J* = 7.8 Hz, 1H, 2-aminophenol-H6), 6.74 (t, *J* = 7.4 Hz, 1H, 2-aminophenol-H4), 3.17 (s, 1H, -C**H$_3$**). ^{13}C NMR (151 MHz, DMSO-d6, 353K) δ 164.55 (triazine-^{4}C), 152.08 (aminophenol-^{4}C1), 131.75 (aminophenol-^{4}C2), 127.72 (aminophenol-^{4}C3), 126.47 (aminophenol-^{4}C5), 118.64 (aminophenol-^{4}C4), 116.73 (aminophenol-^{4}C6), 36.04 (-N**C**H$_3$).

Analytical data for compound 2,2',2''-(1,3,5-triazine-2,4,6-triyltriimino)tris(3-methylphenol) (14):
^{1}H NMR (600 MHz, DMF-*d$_7$*, 223 K, ppm) δ: 48% : 52% mixture of conformers; signals for major *asymmetric* conformer: ^{1}H NMR (600 MHz, DMF-d$_7$ 223 K) δ: 9.85 (s, 3H, -O**H**), 9.03 (s, 3H, -N**H**), 7.05 (dd, J = 7.4, 8.0 Hz, 3H, 3-methylphenol-H5), 6.81 (d, J = 8.0 Hz, 3H, 3-methylphenol-H6), 6.77 (d, J = 7.4 Hz, 3H, 3-methylphenol-H4), 2.25 (s, 9H, -C**H$_3$**). ^{13}C NMR (151 MHz, DMF-d$_7$, 223K) δ 165.18 (triazine-^{4}C), 153.26 (3-methylphenol-^{4}C1), 136.04 (3-methylphenol-^{4}C2), 126.66 (3-methylphenol-C5), 125.91 (3-methylphenol-^{4}C3), 121.16 (3-methylphenol-C4), 115.19 (3-methylphenol-C6), 18.29 (-**C**H$_3$) ; signals for minor *propeller* conformer: ^{1}H NMR (600 MHz, DMF-d$_7$, 223K) δ: 9.77 (s, 1H, -O**H**), 9.70 (s, 1H, -O**H**), 9.08 (s, 1H, -N**H**), 8.93 (s, 1H, -N**H**), 8.81 (s, 1H, -N**H**), 7.07 (dd, J = 7.4, 8.0 Hz, 1H, 3-methylphenol-H5), 6.91 (dd, J = 7.4, 8.0 Hz, 1H, 3-methylphenol-H5), 6.90 (dd, J = 7.4, 8.0 Hz, 1H, 3-methylphenol-H5), 6.84 (d, J = 8.0 Hz, 1H, 3-methylphenol-H6), 6.79 (d, J = 7.4 Hz, 1H, 3-methylphenol-H4), 6.64 (d, J = 7.4 Hz, 1H, 3-methylphenol-H4), 6.63-6.59 (m, 3H, 2*3-methylphenol-H6 & 3-methylphenol-H4), 6.61 (d, J = 7.3 Hz, 1H), 2.31 (s, 3H, -C**H$_3$**), 2.21 (s, 3H, -C**H$_3$**), 2.13 (s, 3H, -C**H$_3$**). ^{13}C NMR (151 MHz, DMF-d$_7$, 223K) δ 165.5 (triazine-^{4}C), 165.4 (triazine-^{4}C), 165.4 (triazine-^{4}C), 153.41 (3-methylphenol-^{4}C1), 153.28 (3-methylphenol-^{4}C1), 153.19 (3-methylphenol-^{4}C1), 136.60 (3-methylphenol-^{4}C2), 135.25 (3-methylphenol-^{4}C2), 135.20 (3-methylphenol-^{4}C2), 126.69 (3-methylphenol-C5), 126.49 (3-methylphenol-C5), 126.36 (3-methylphenol-C5), 125.87 (3-methylphenol-^{4}C3), 125.61 (3-methylphenol-^{4}C3), 125.38 (3-methylphenol-^{4}C3), 121.10 (3-methylphenol-C4), 121.02 (3-methylphenol-C4), 120.96 (3-methylphenol-C4), 115.34 (3-methylphenol-C6), 115.26 (3-methylphenol-C6), 114.82 (3-methylphenol-C6), 18.37 (-**C**H$_3$), 18.14 (-**C**H$_3$), 18.06 (-**C**H$_3$).
^{1}H NMR (600 MHz, DMSO-*d$_6$*, 353 K, ppm) δ: averaged signals due to fast exchange: 8.91 (bs, 1H, OH), 7.83 (bs, 1H, NH), 6.92 (t, *J* = 7.8 Hz, 1H, 3-methylphenol-H5), 6.65 (t, *J* = 7.5 Hz, 2H, 3-methylphenol-H4 and 3-methylphenol-H6), 2.16 (s, 3H, -C**H$_3$**). ^{13}C NMR (151 MHz, DMSO-d6, 353K) δ 164.80 (triazine-^{4}C), 152.32 (3-methylphenol-^{4}C1), 135.28 (3-methylphenol-^{4}C3), 125.67 (3-methylphenol-^{4}C2), 124.97 (3-methylphenol-^{4}C5), 120.44 (3-methylphenol-^{4}C4), 114.29 (3-methylphenol-^{4}C6), 26.46 (C), 17.74 (C**H$_3$**).

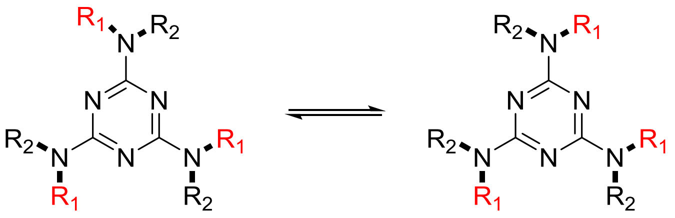

(propeller) (asymmetric)

IR typical vibrational frequencies of studied compounds.

Comp.	R_1	R_2	υ_{NH}	υ_{OH}	$\upsilon_{CH}(Ar)$	$\upsilon_{C=N}$
**4**	H	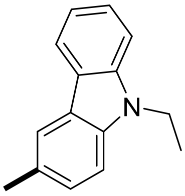	3400, 3376	-	3050<br>3020	1505
**5**	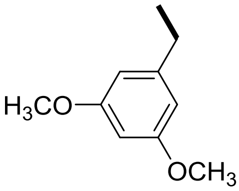	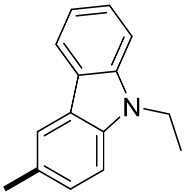	-	-	3050<br>3021	1539
**6**	CH_3	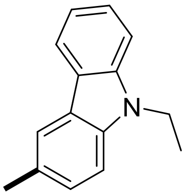	-	-	3050<br>3025	1538
**11**	H	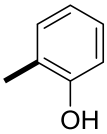	3409, 3396,<br>3388	3280<br>(broad)	3050	1510
**12**	H	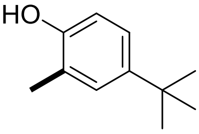	3383, 3282,<br>3189	3280<br>(broad)	3063	1507
**13**	CH_3	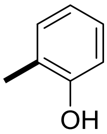	-	3247<br>(broad)	3069	1540
**14**	H	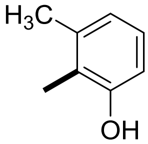	3359	3274<br>(broad)	3085	1504

^{1}H and ^{13}C NMR spectra of the studies compounds:

Compound **4**:

^{1}H NMR (600 MHz, DMSO-d6, 393 K):

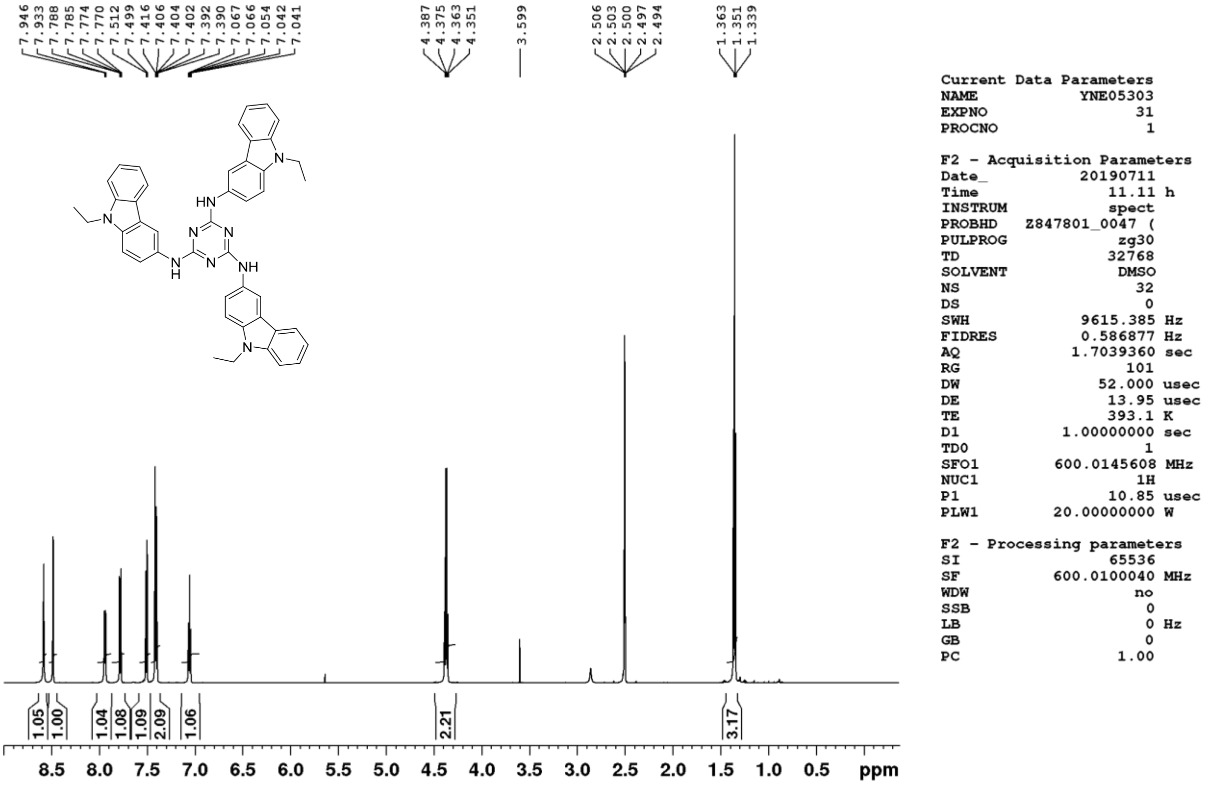

^{1}H NMR (600 MHz, DMSO-d6, 293 K):

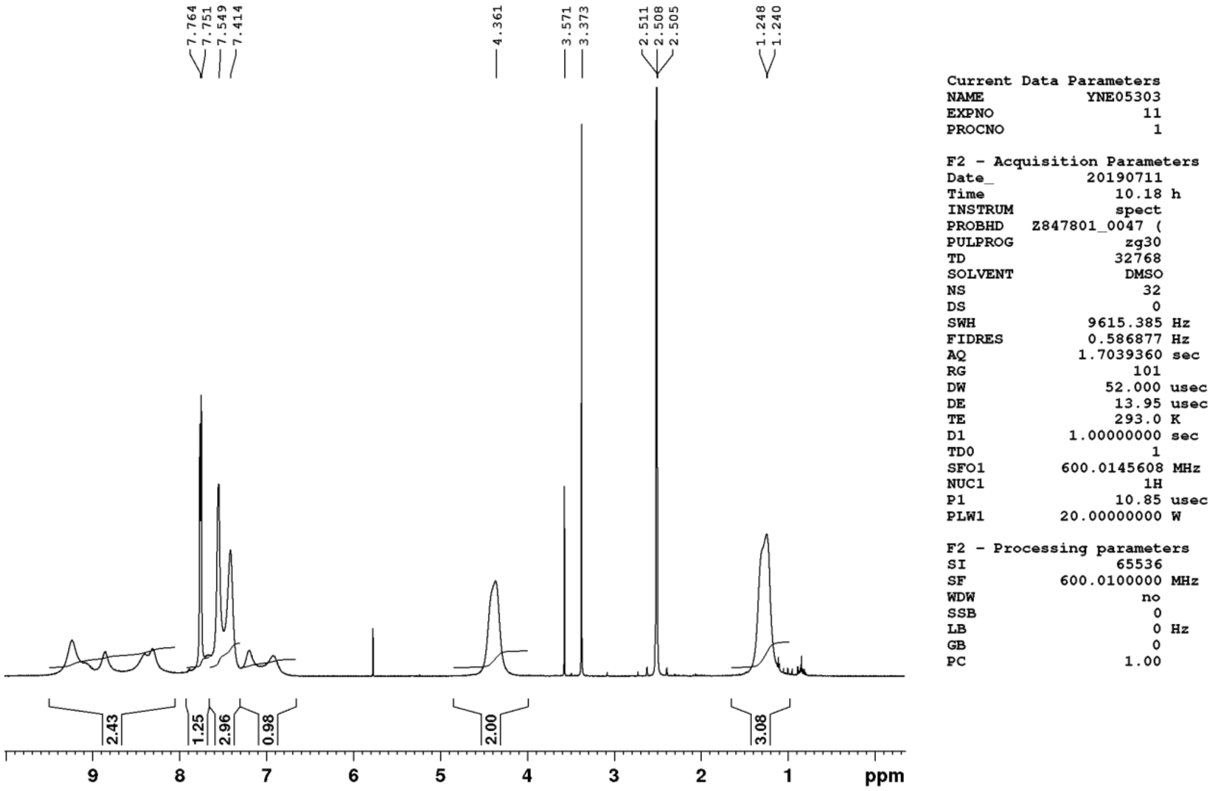

^{13}C NMR (150.9 MHz, DMSO-d6, 393 K):

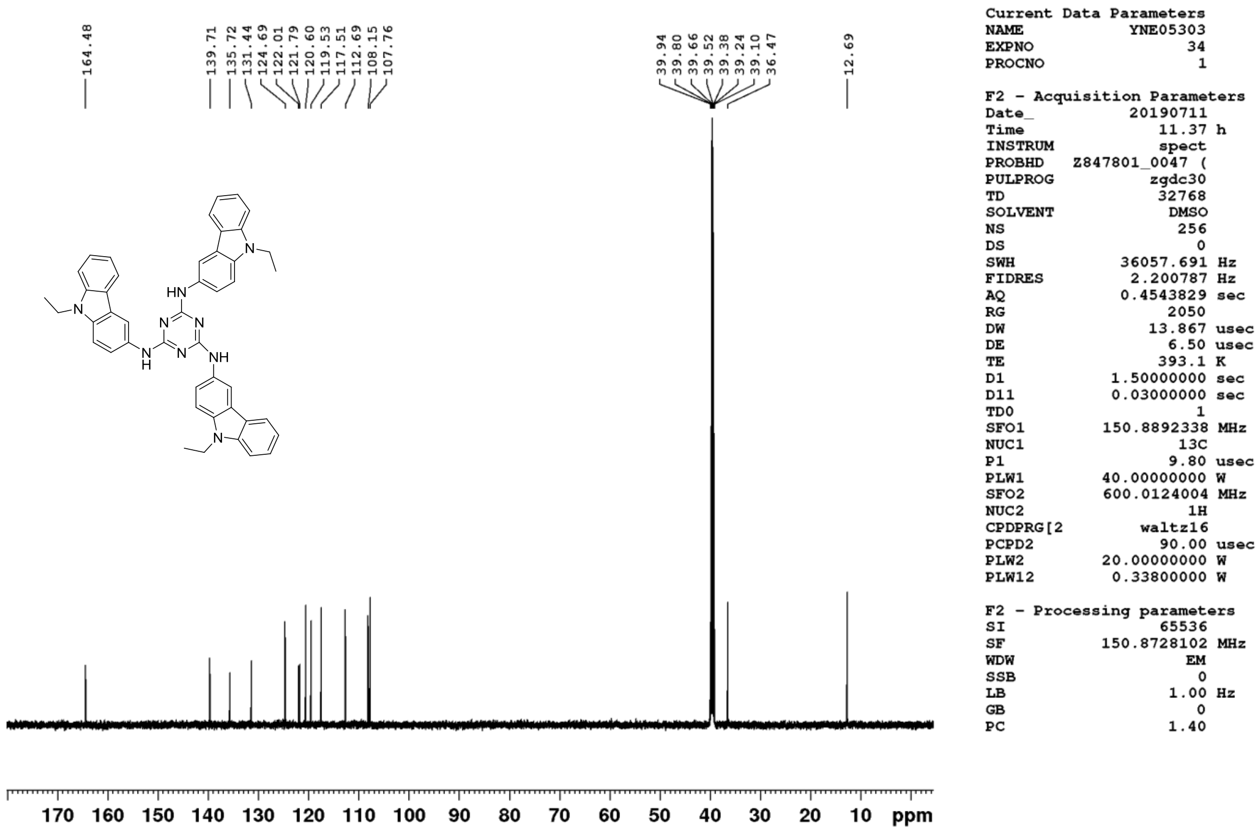

^{1}H NMR (600 MHz, DMF-d7, 293 K):

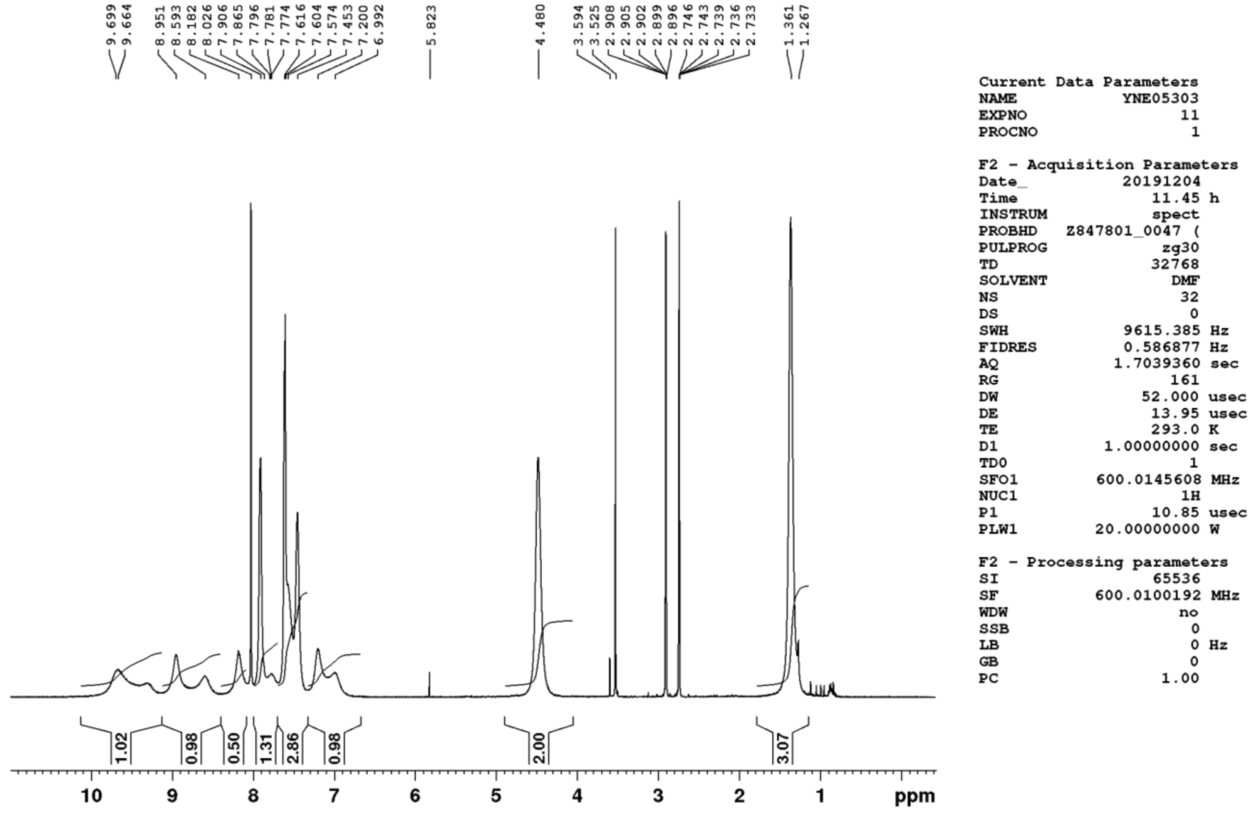

^{1}H NMR (600 MHz, DMF-d7, 223 K):

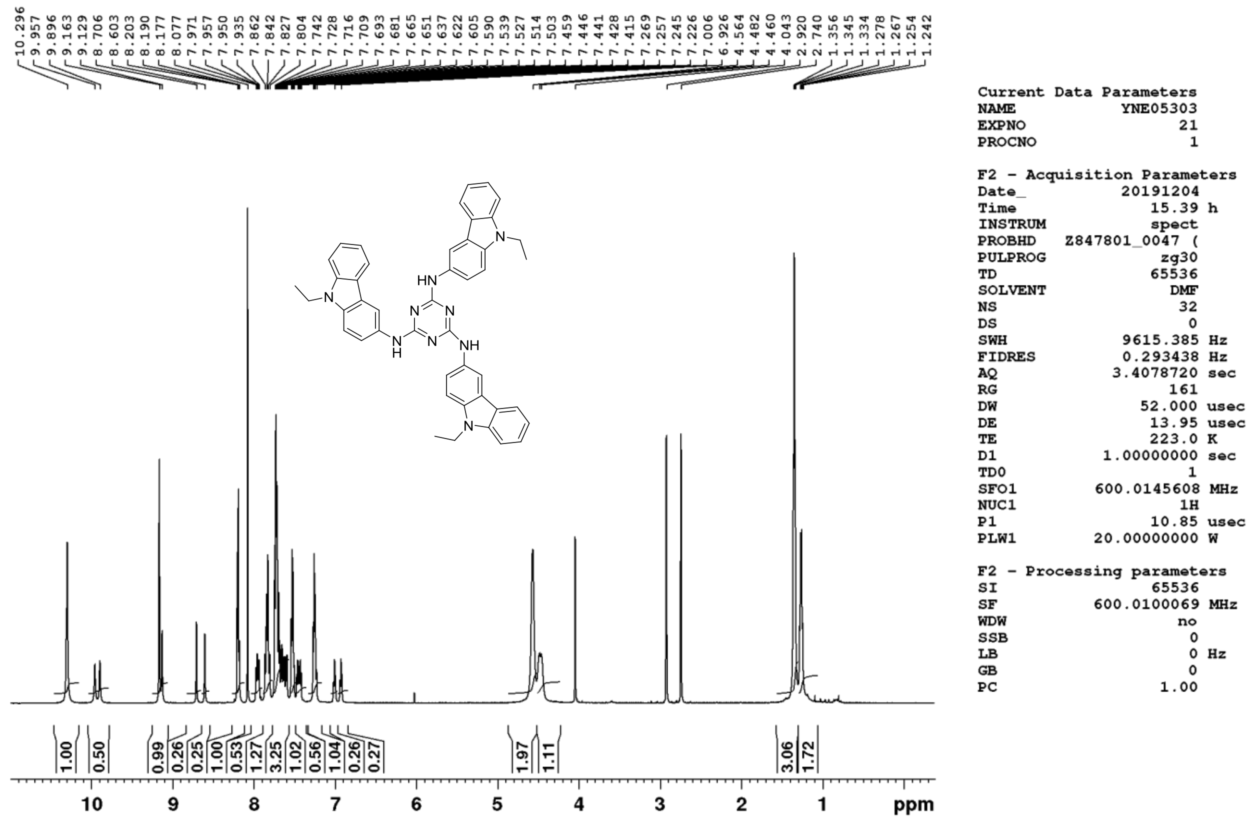

^{13}C NMR (150.9 MHz, DMF-d7, 223 K):

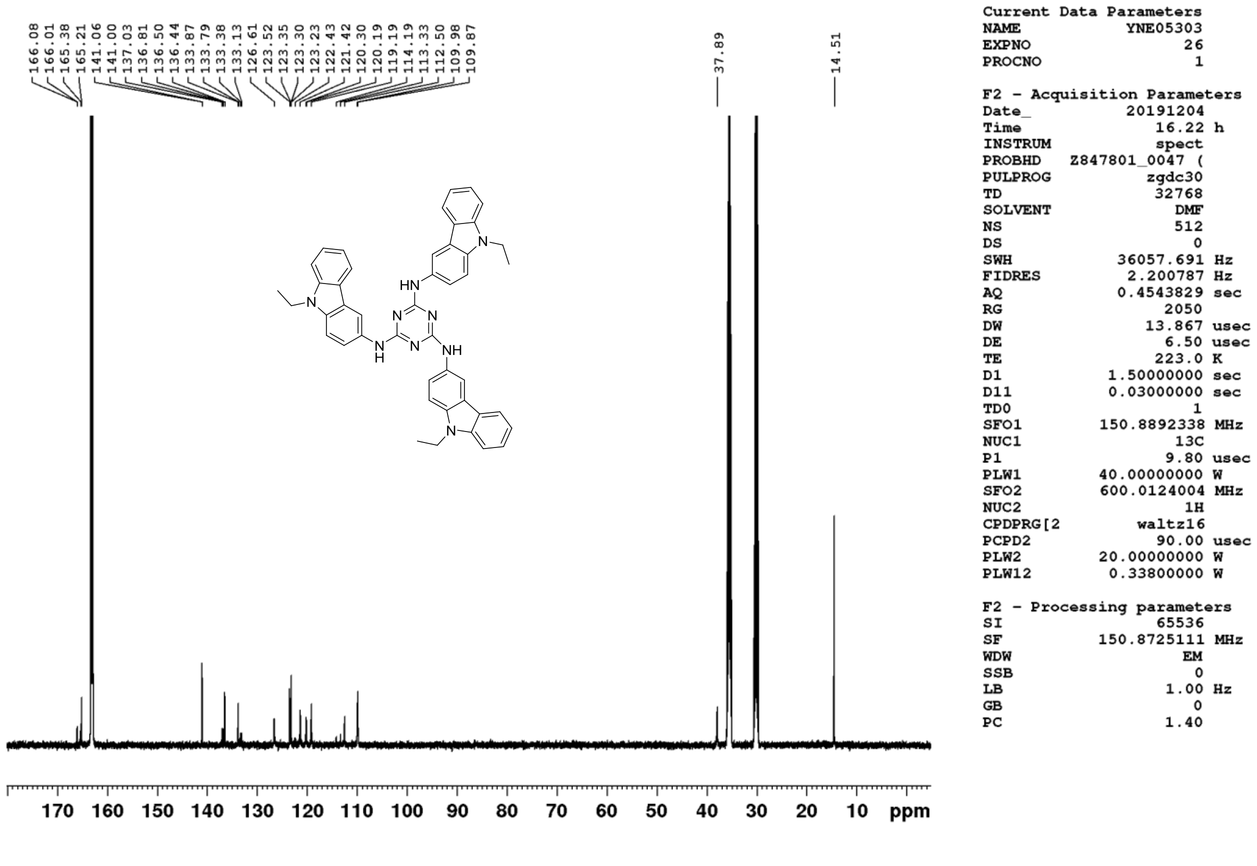

Compound **5**:

^{1}H NMR (600 MHz, DMSO-d6, 403 K):

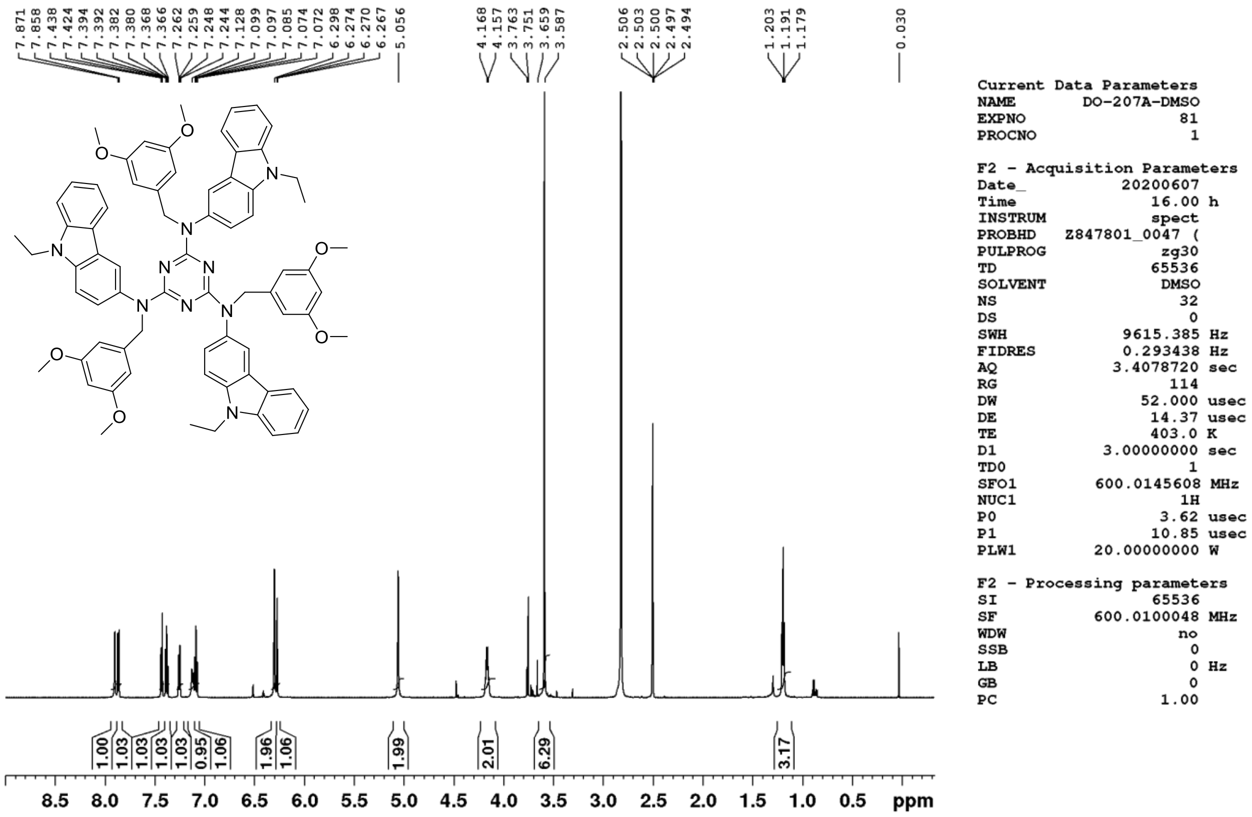

^{1}H NMR (600 MHz, DMSO-d6, 293 K):

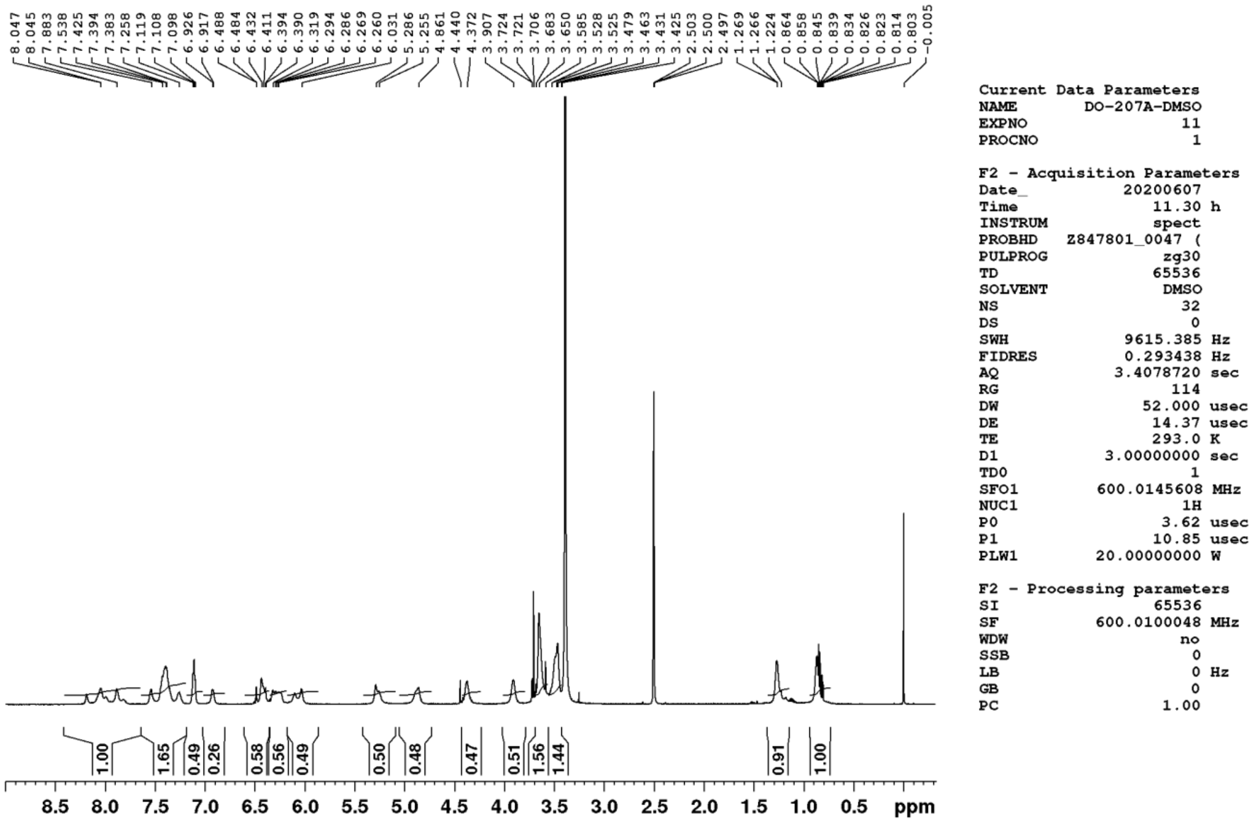

^{13}C NMR (150.9 MHz, DMSO-d6, 403 K):

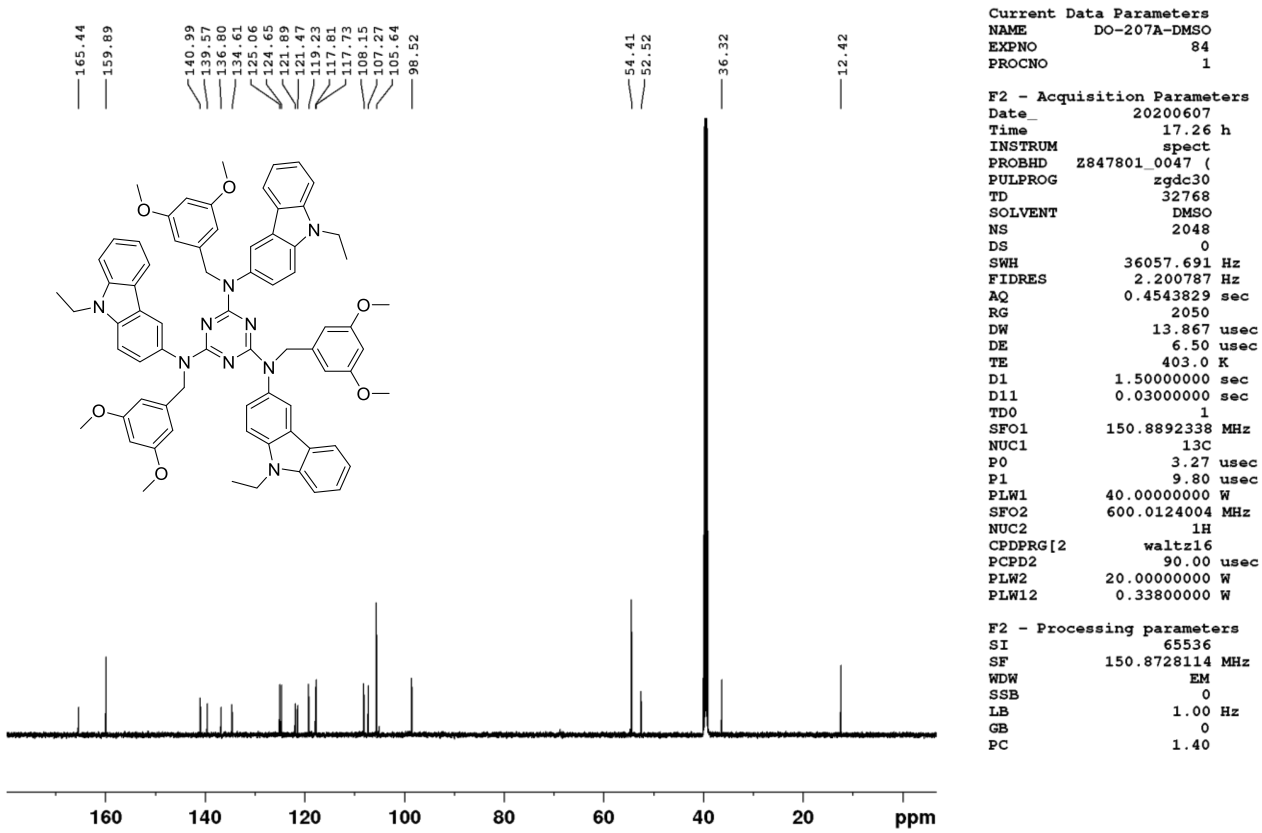

^{1}H NMR (600 MHz, DMF-d7, 223 K):

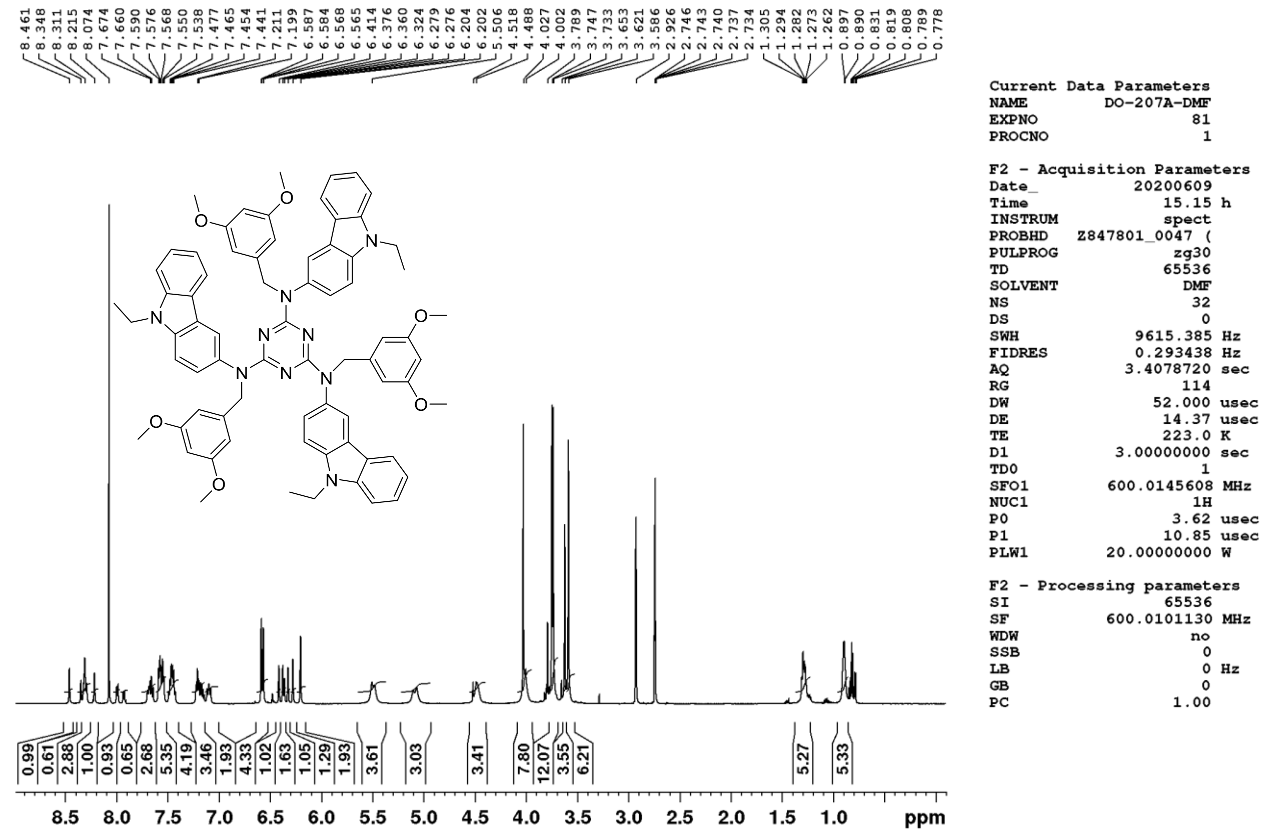

^{1}H NMR (600 MHz, DMF-d7, 293 K):

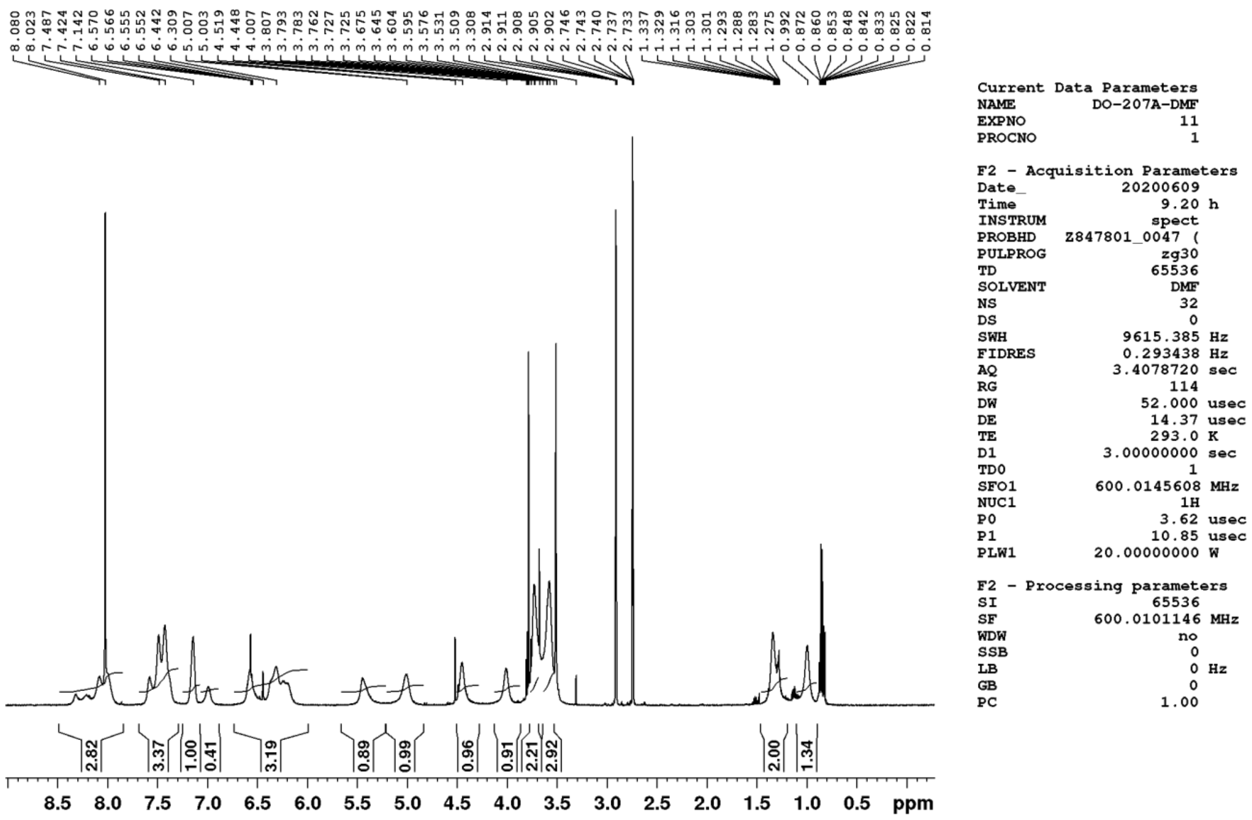

^{13}C NMR (150.9 MHz, DMF-d7, 223 K):

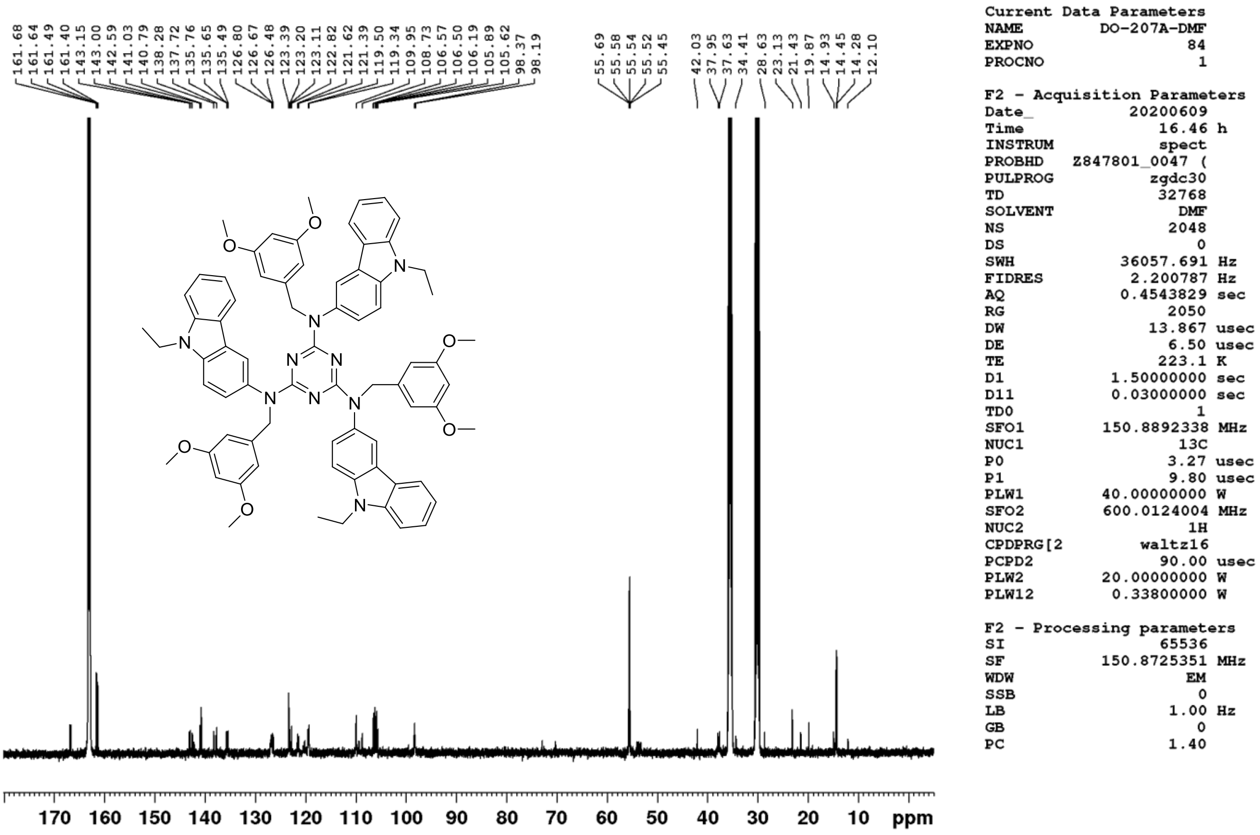

Compound **6**:

^{1}H NMR (600 MHz, DMSO-d6, 373 K):

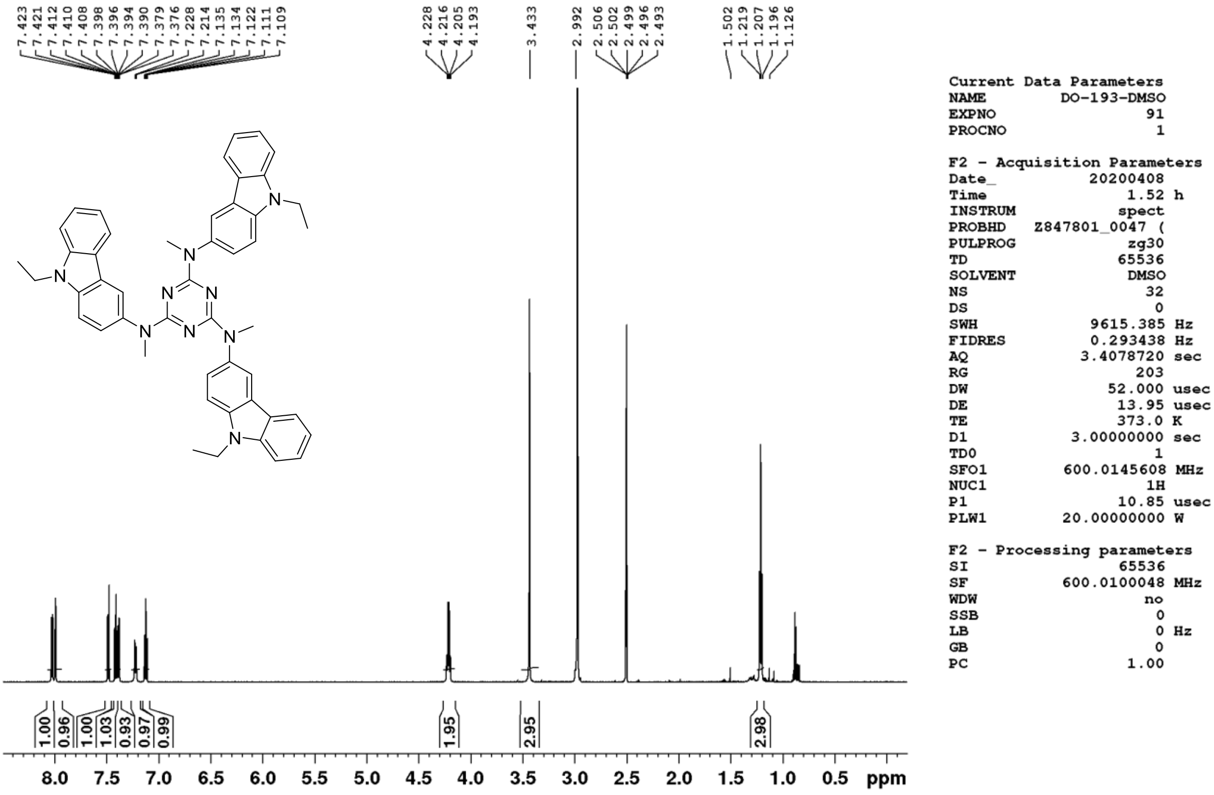

^{1}H NMR (600 MHz, DMSO-d6, 293 K):

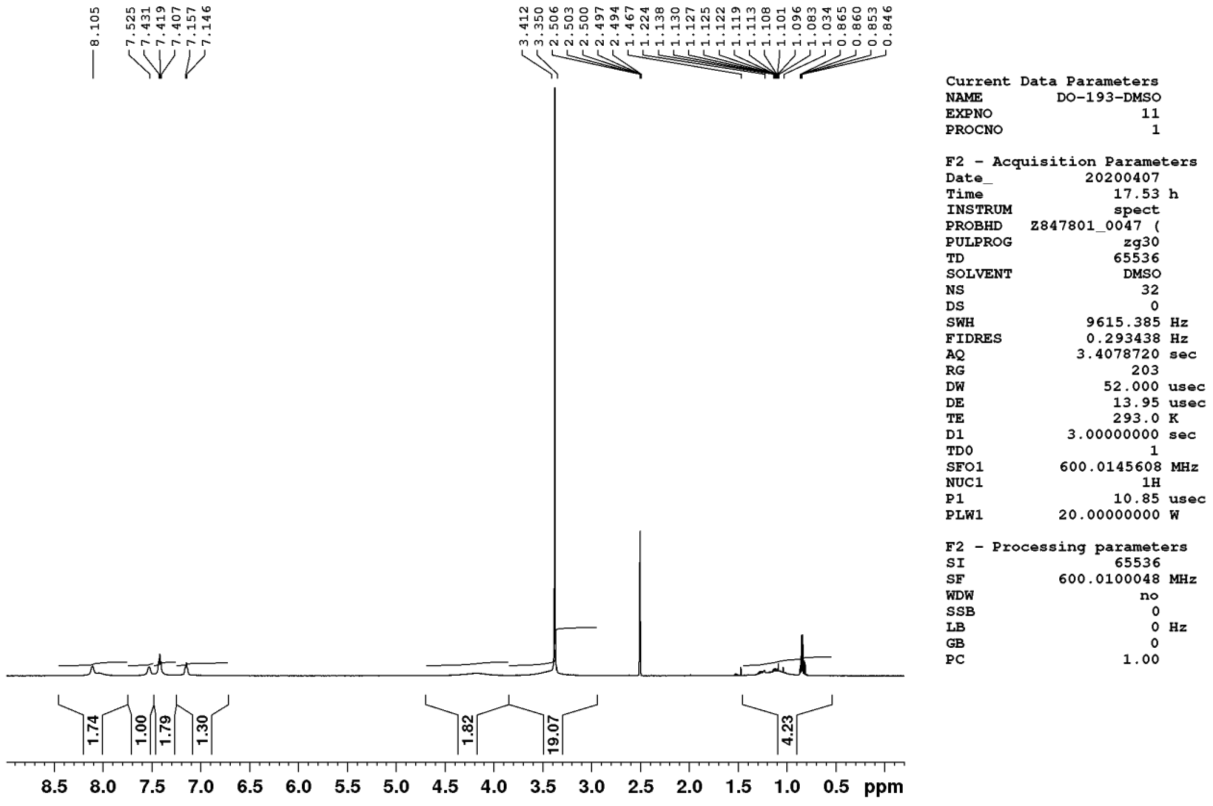

^{13}C NMR (150.9 MHz, DMSO-d6, 373 K):

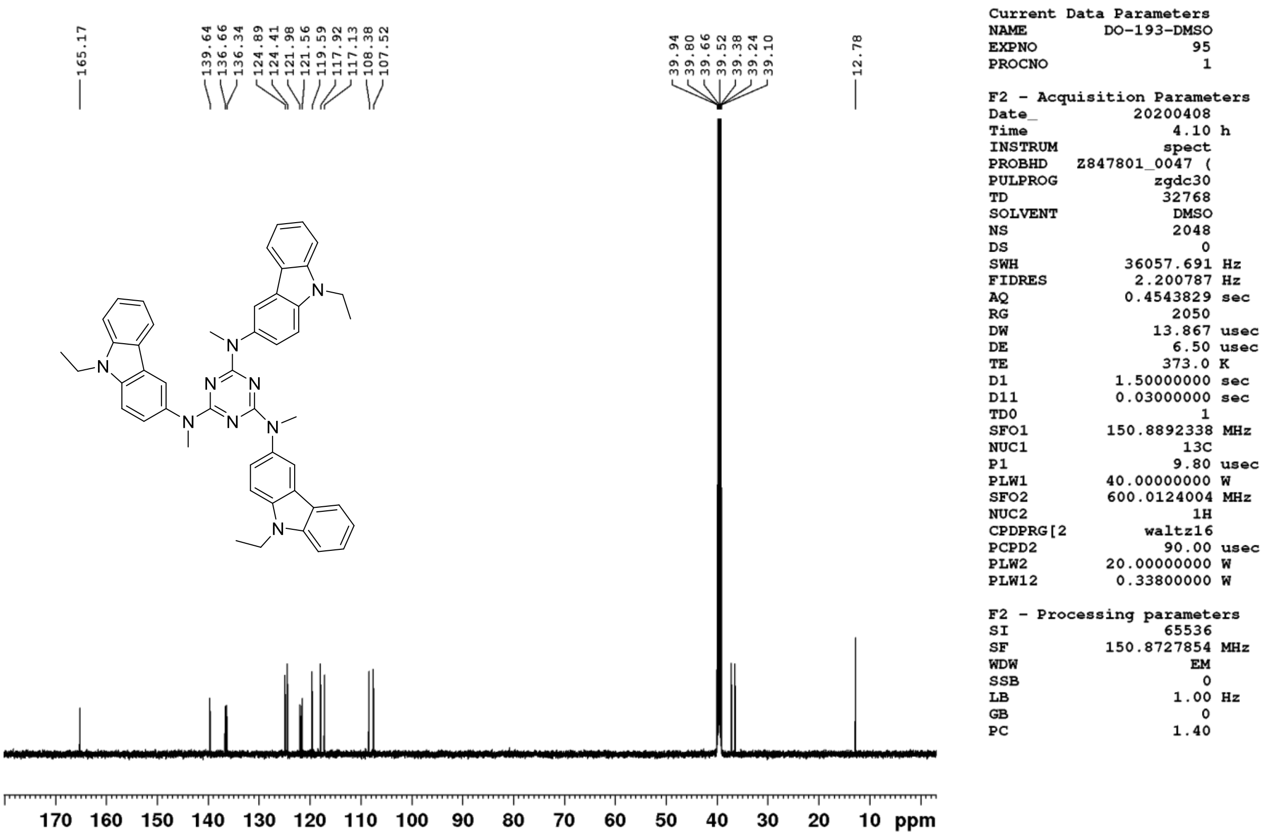

^{1}H NMR (600 MHz, DMF-d7, 293 K):

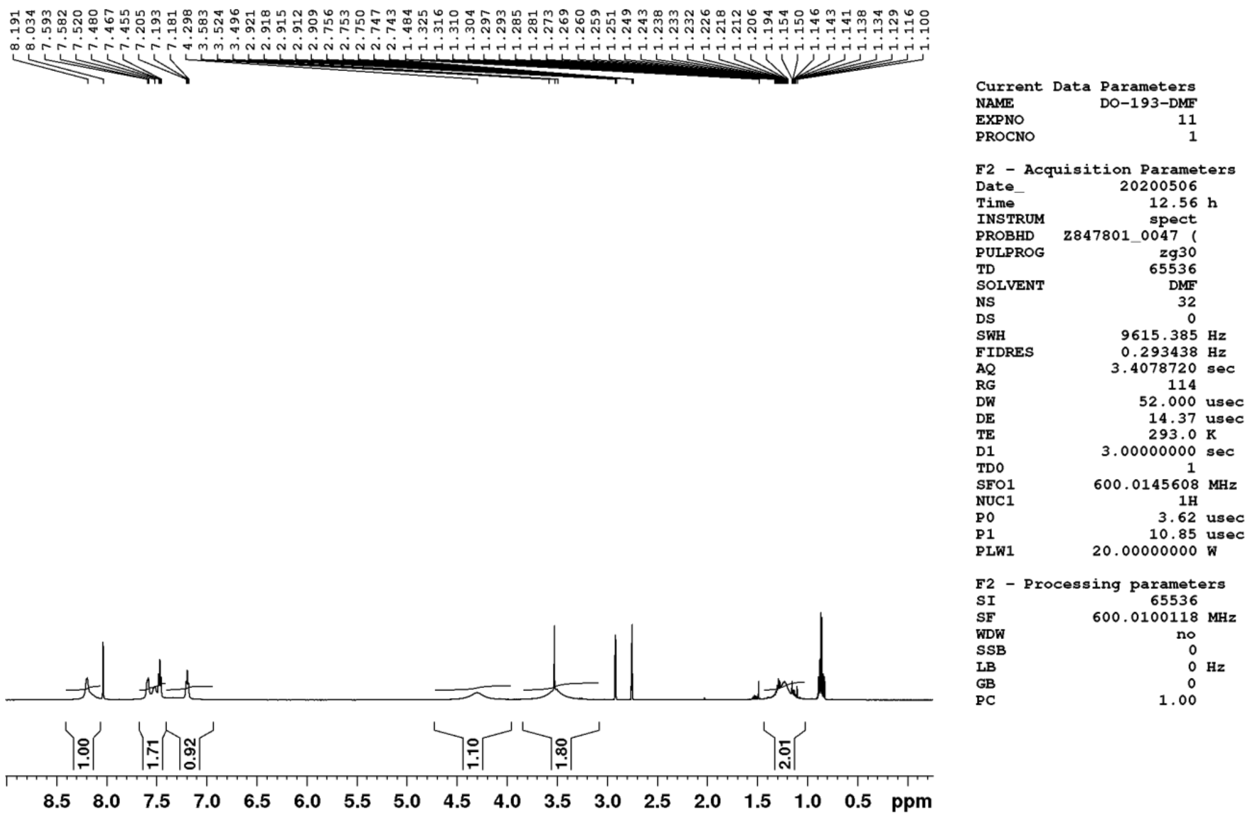

^{1}H NMR (600 MHz, DMF-d7, 223 K):

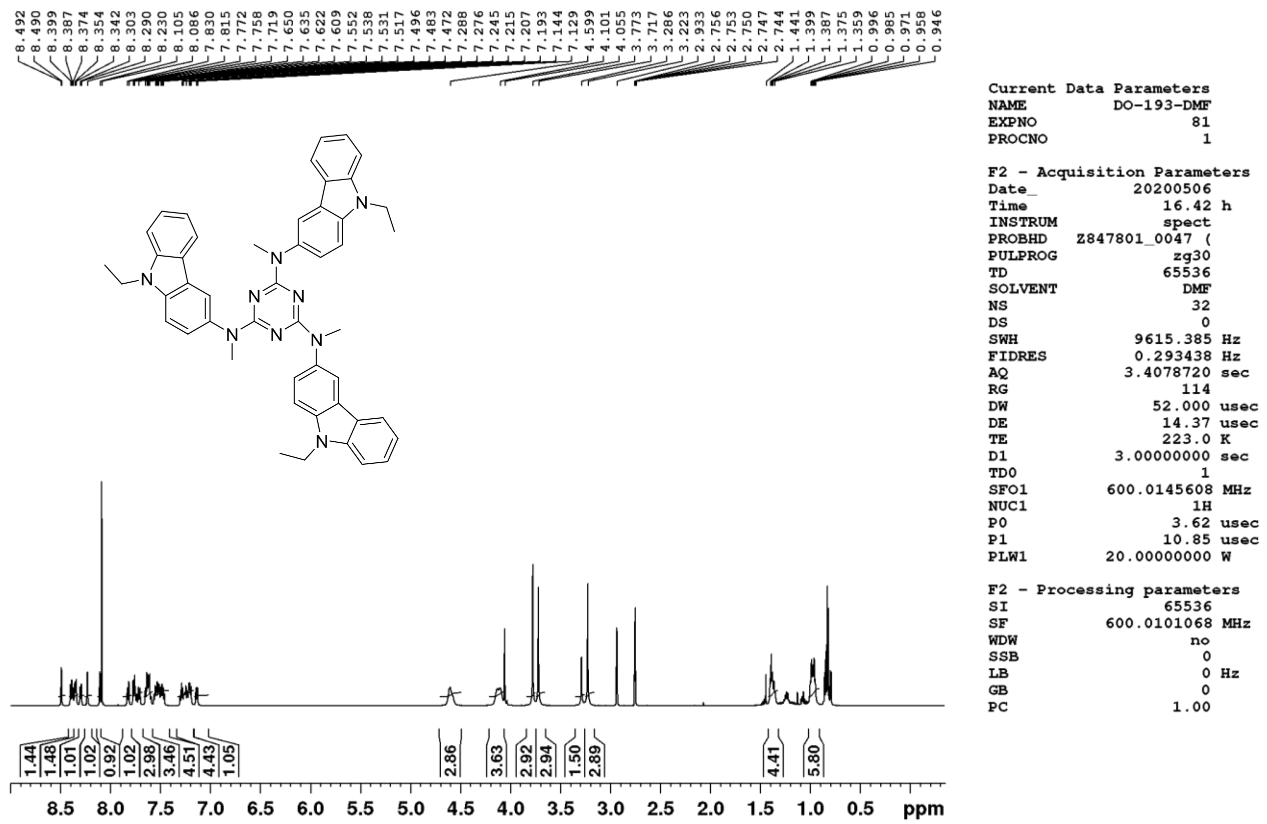

^{13}C NMR (150.9 MHz, DMF-d7, 223 K):

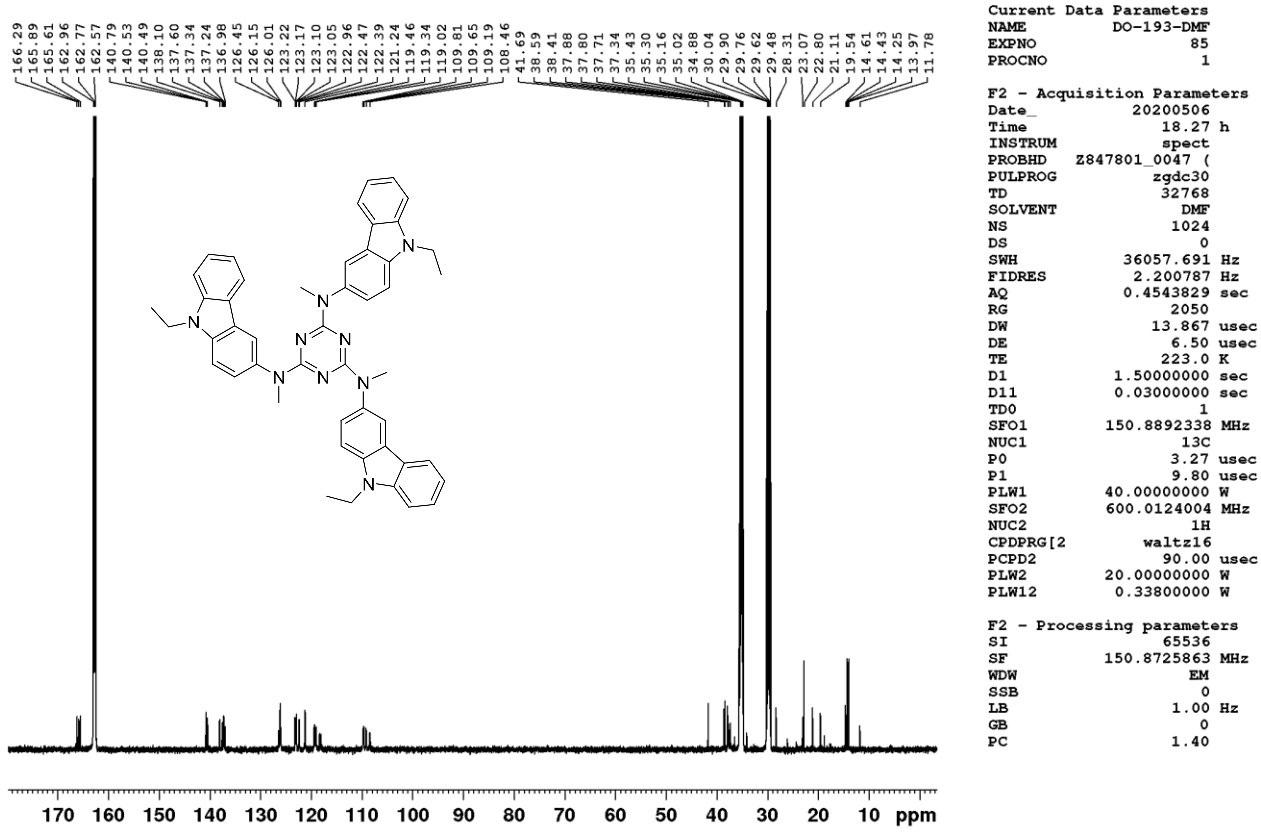

Compound **11**:

^{1}H NMR (600 MHz, DMSO-d6, 293 K):

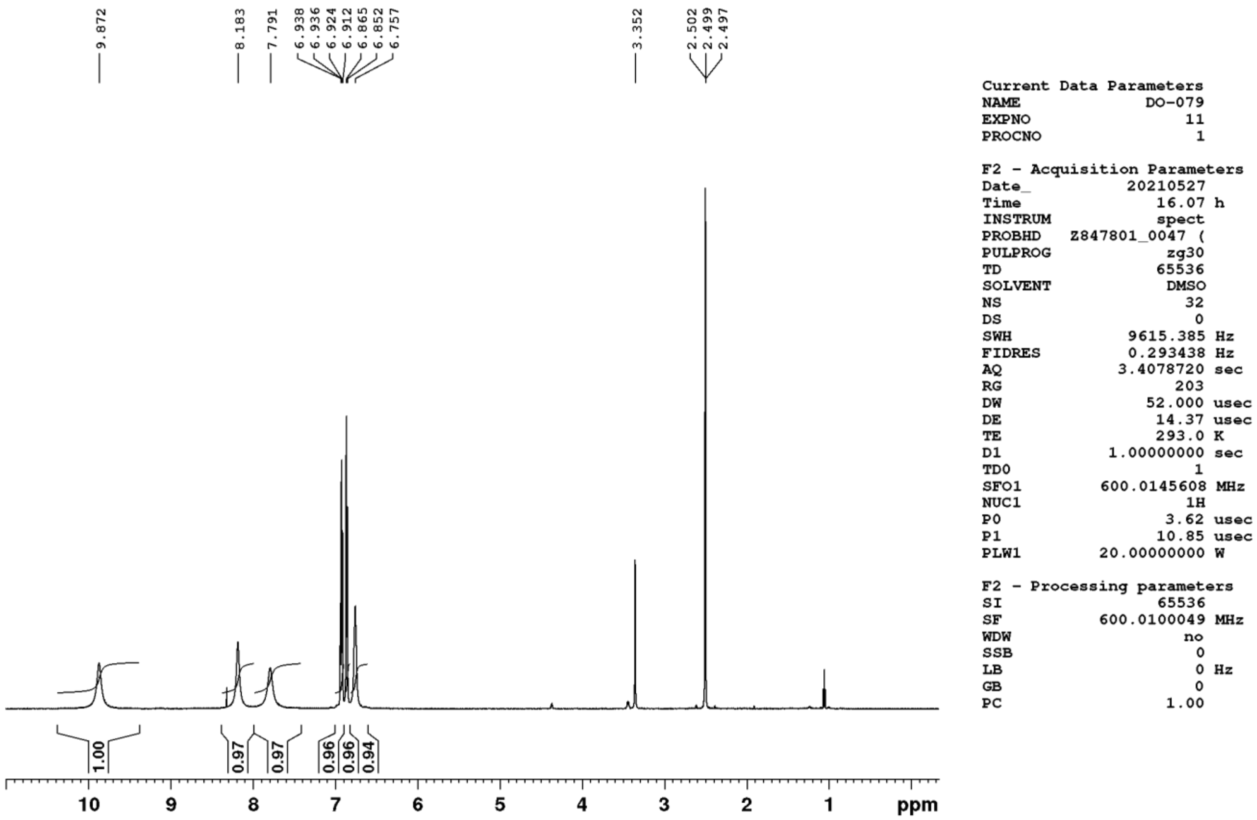

^{1}H NMR (600 MHz, DMSO-d6, 353 K):

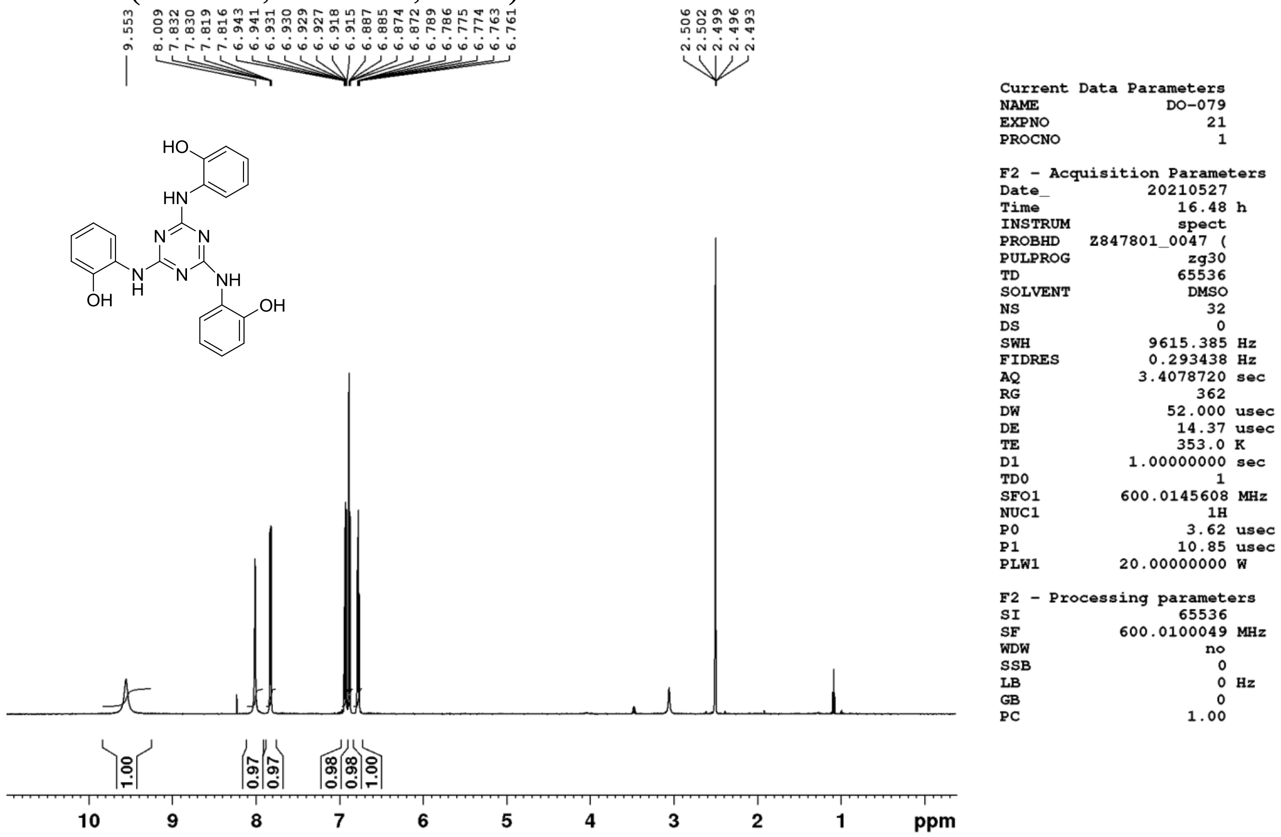

^{13}C NMR (150.9 MHz, DMSO-d6, 353 K):

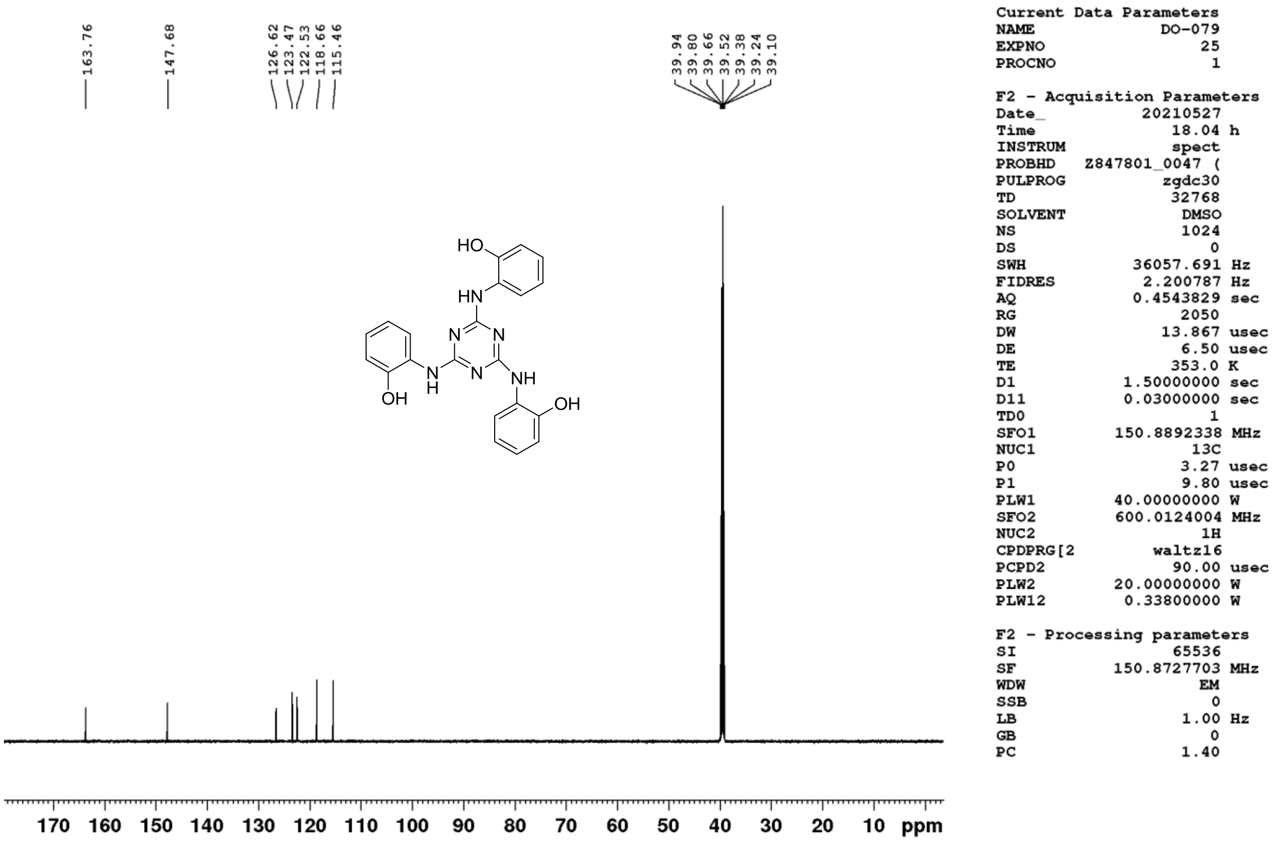

^{1}H NMR (600 MHz, DMF-d7, 293 K):

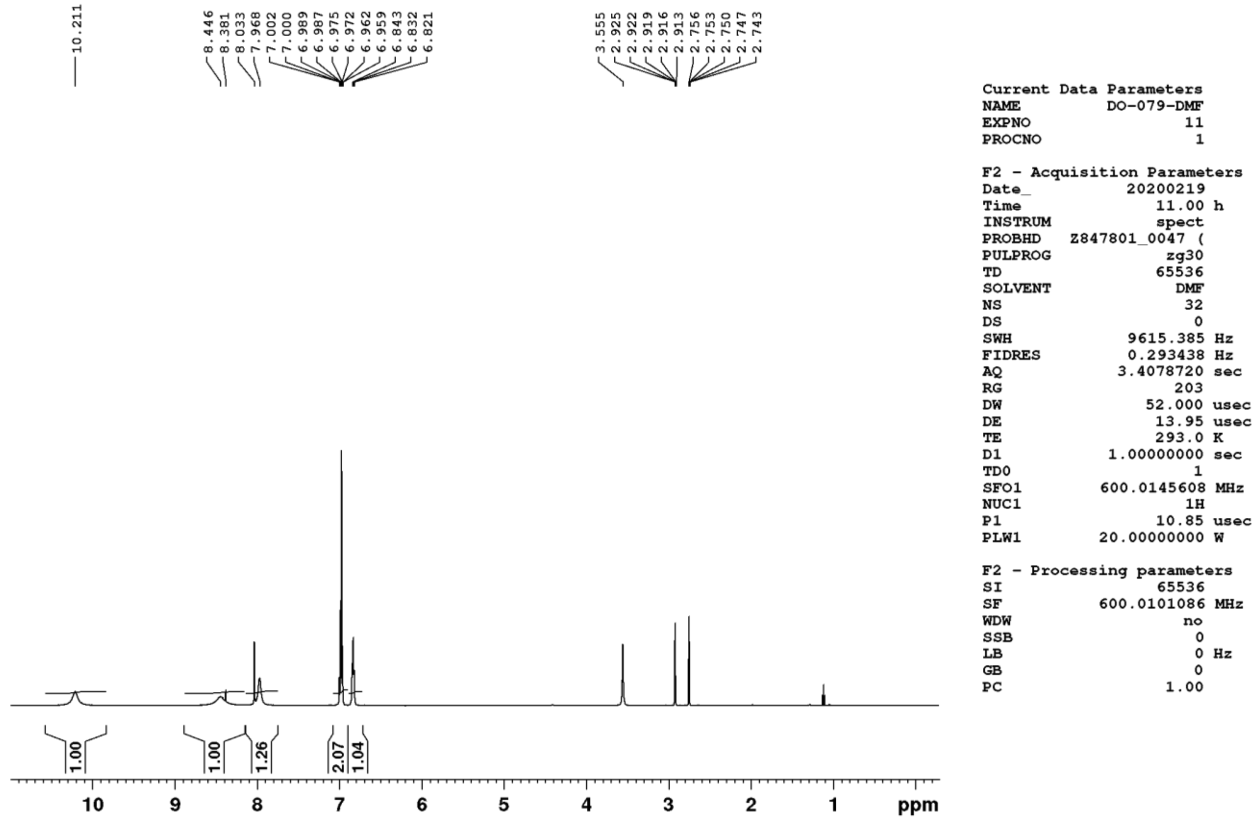

^{1}H NMR (600 MHz, DMF-d7, 223 K):

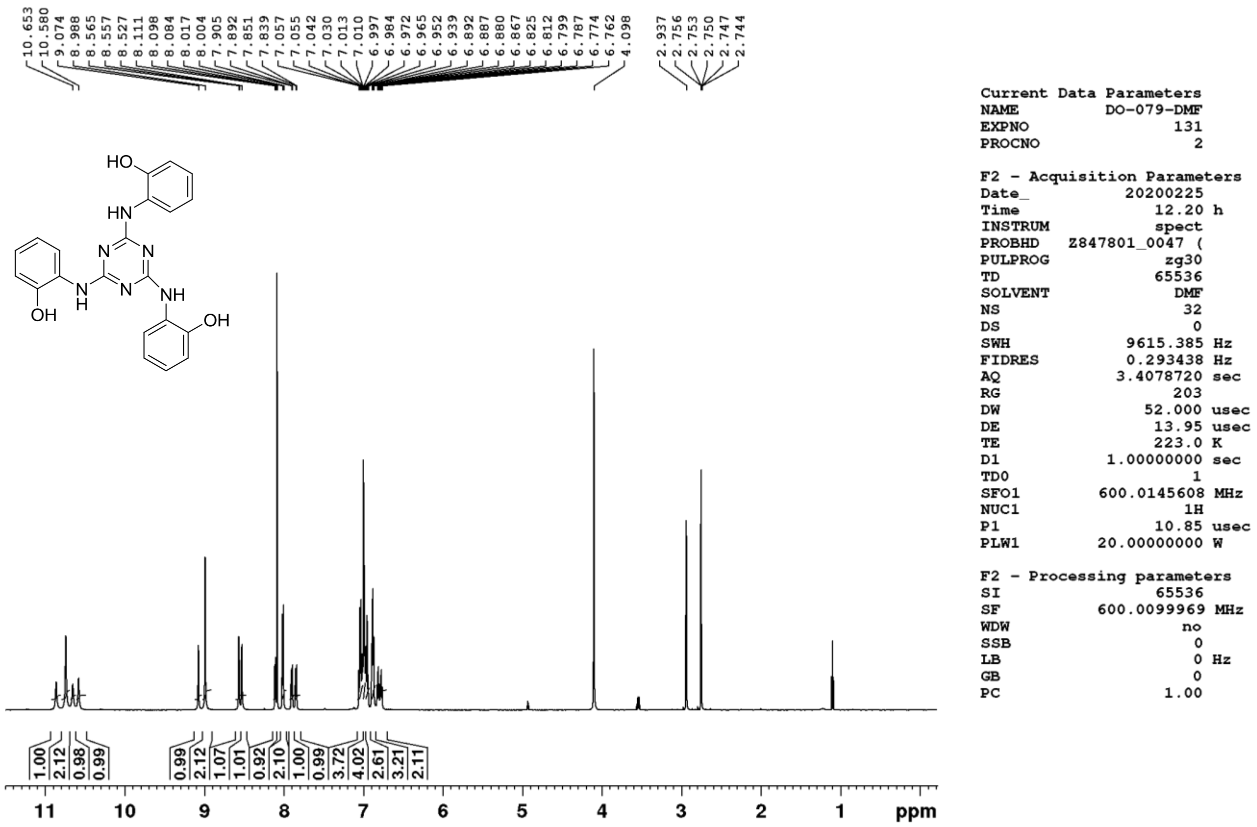

^{13}C NMR (150.9 MHz, DMF-d7, 223 K):

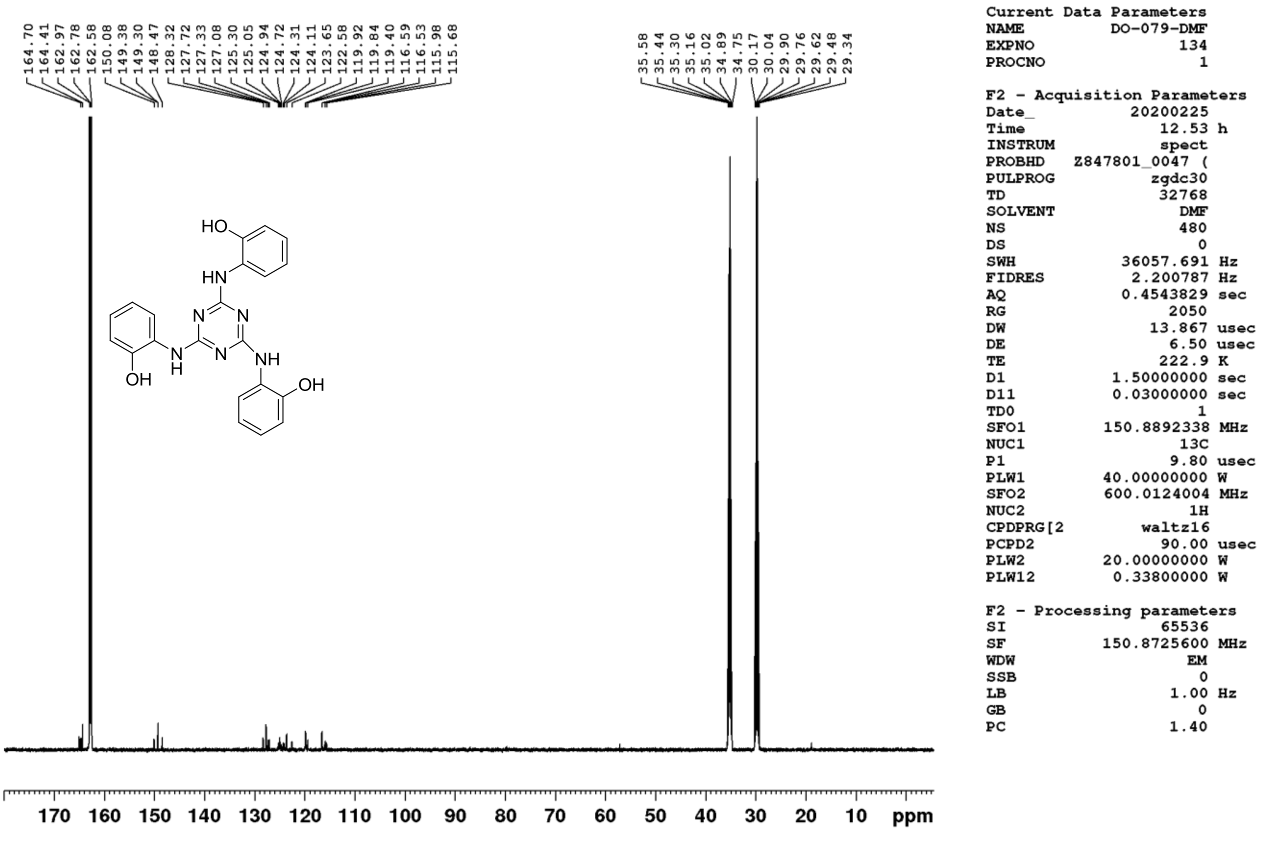

Compound **12**:

^{1}H NMR (600 MHz, DMSO-d6, 293 K):

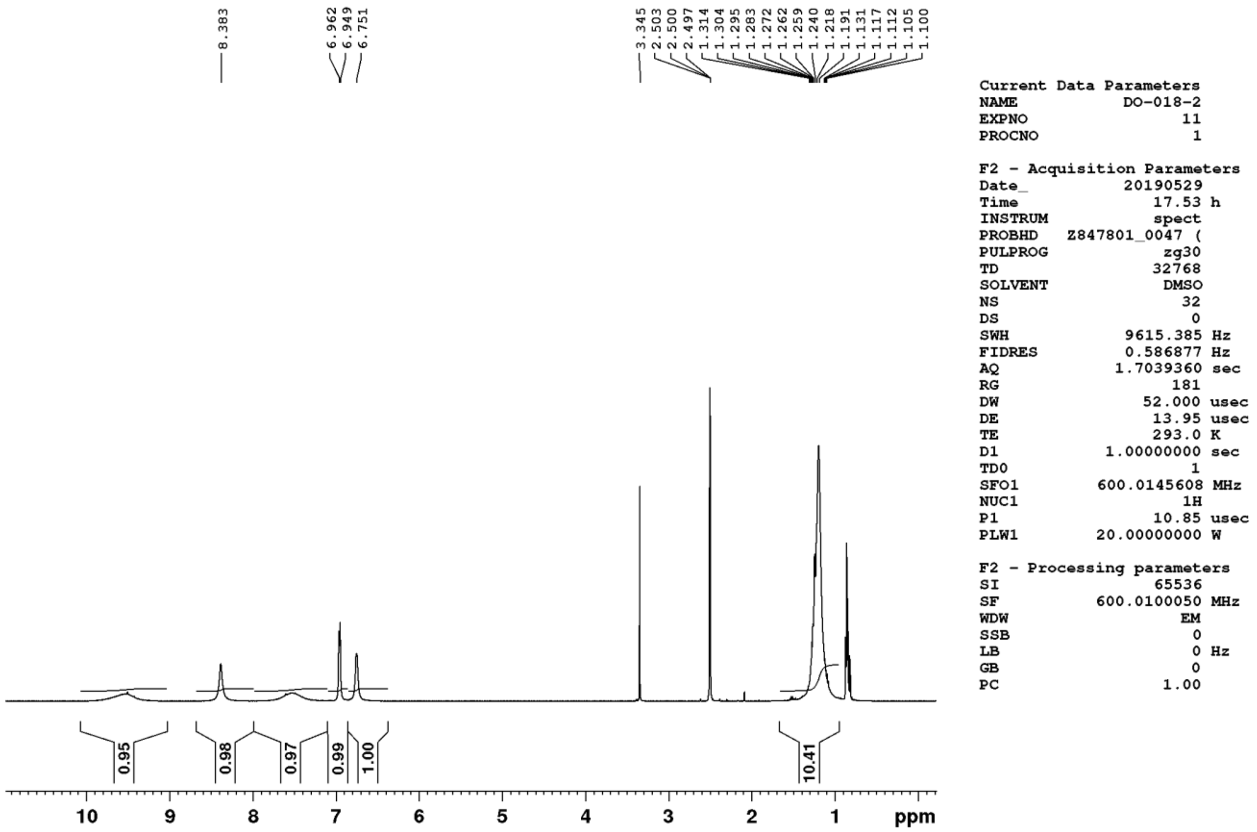

^{1}H NMR (600 MHz, DMSO-d6, 373 K):

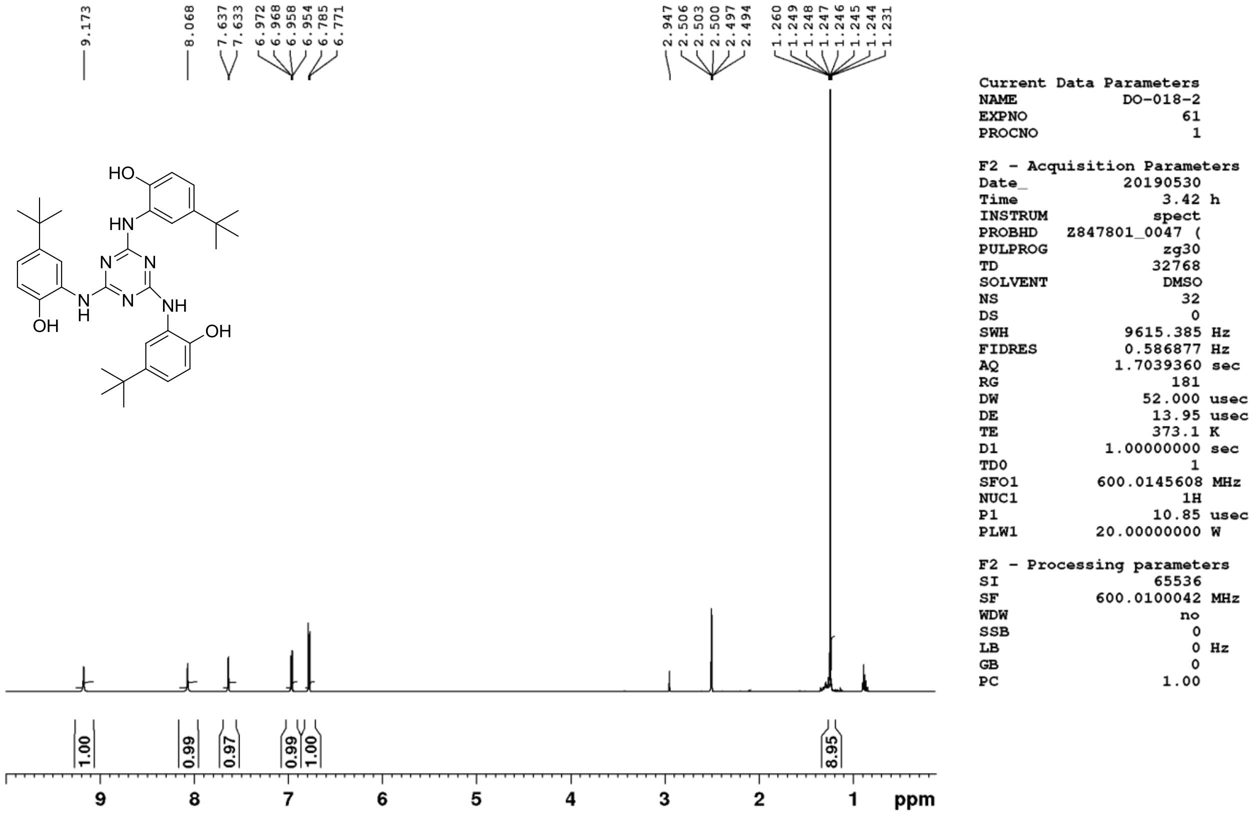

^{13}C NMR (150.9 MHz, DMSO-d6, 373 K):

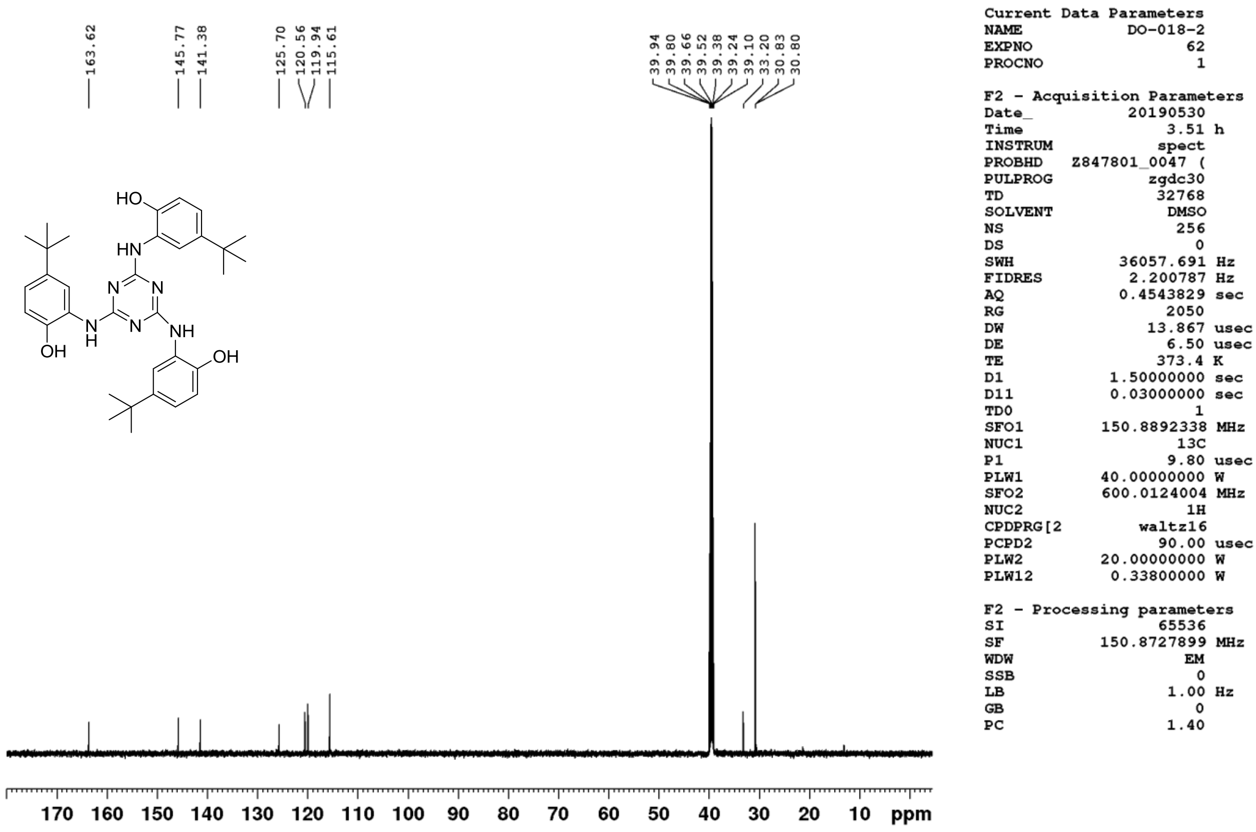

^{1}H NMR (600 MHz, DMF-d7, 293 K):

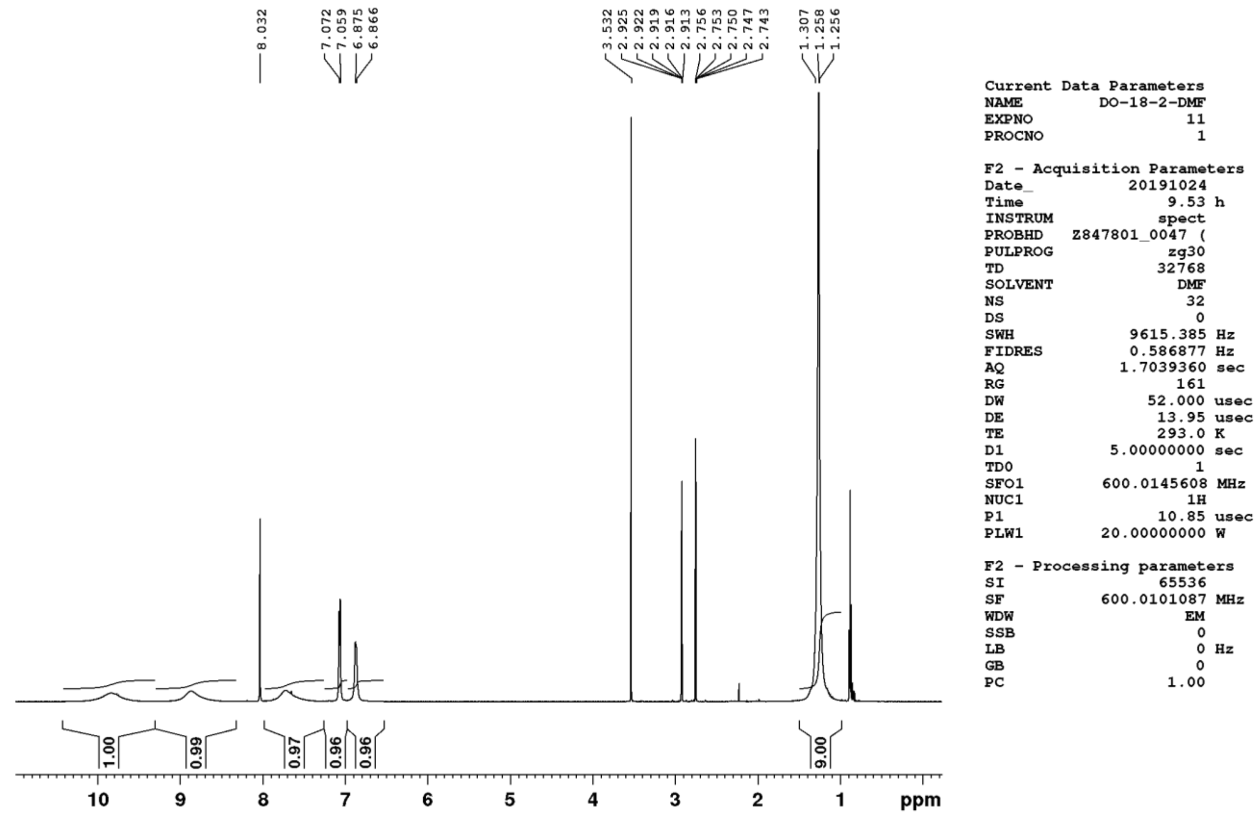

^{1}H NMR (600 MHz, DMF-d7, 233 K):

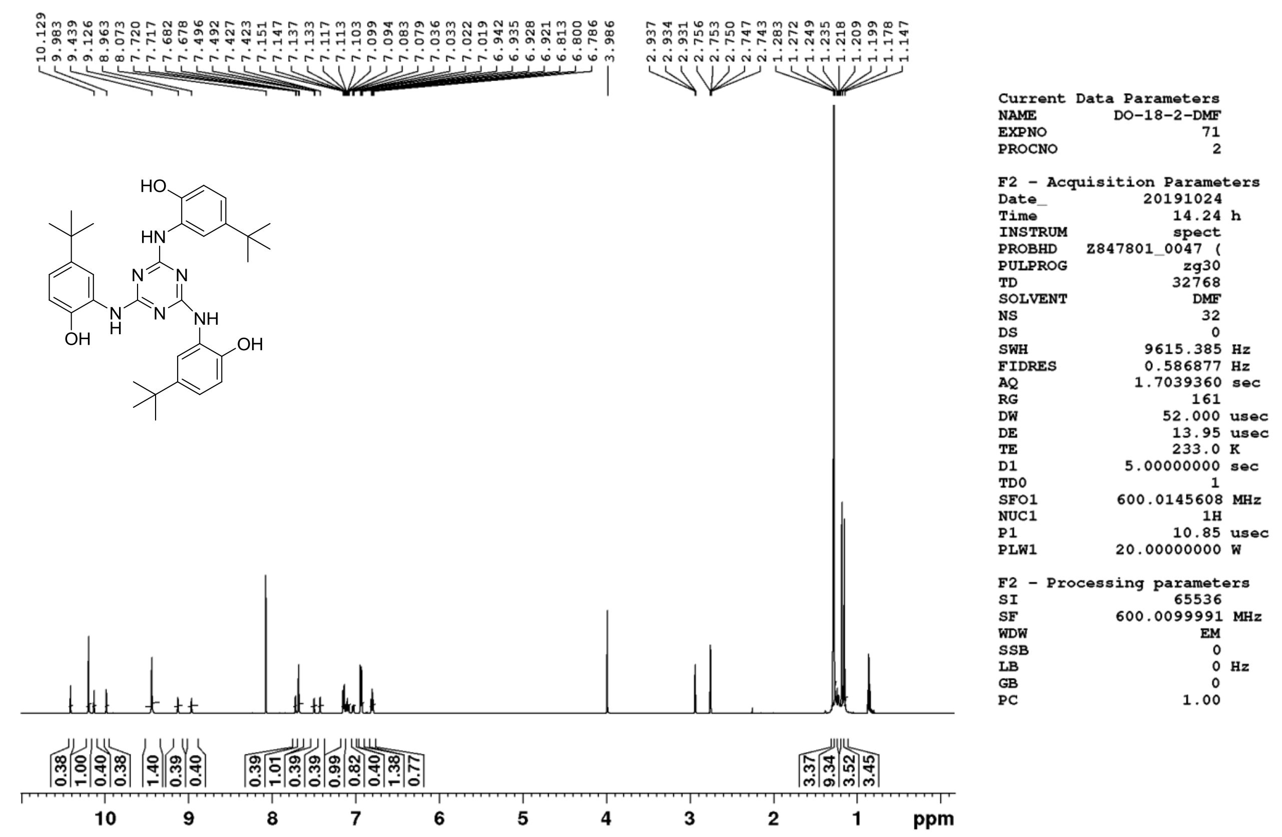

^{13}C NMR (150.9 MHz, DMF-d7, 233 K):

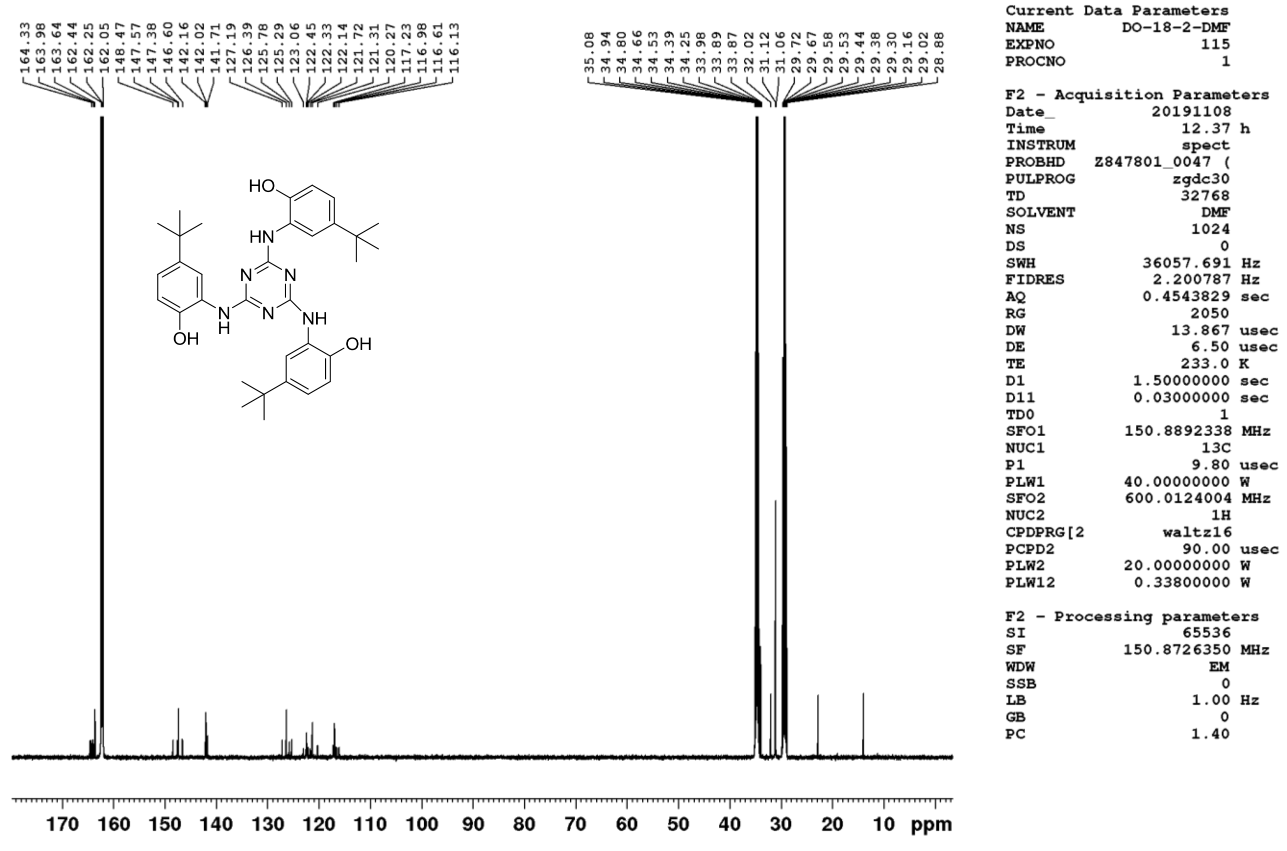

Compound **13**:
^{1}H NMR (600 MHz, DMSO-d6, 293 K):

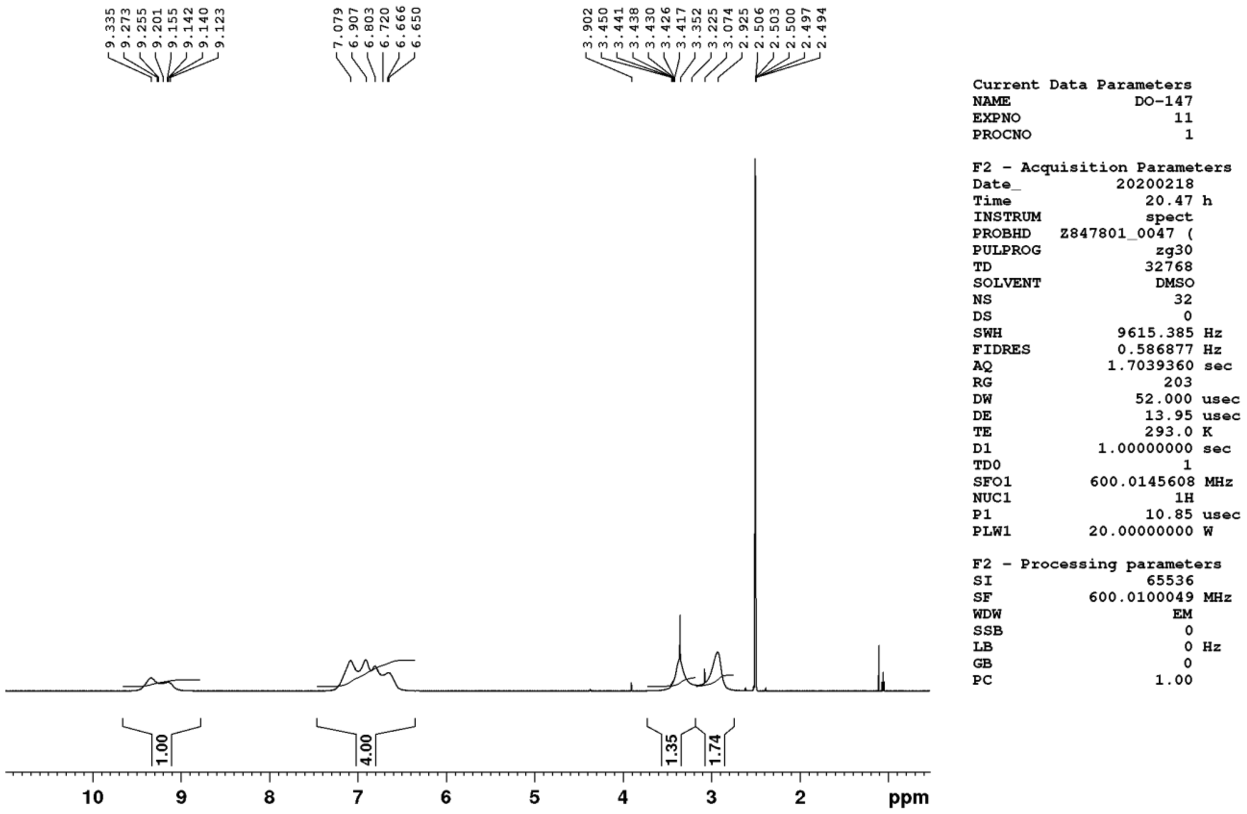

^{1}H NMR (600 MHz, DMSO-d6, 353 K):

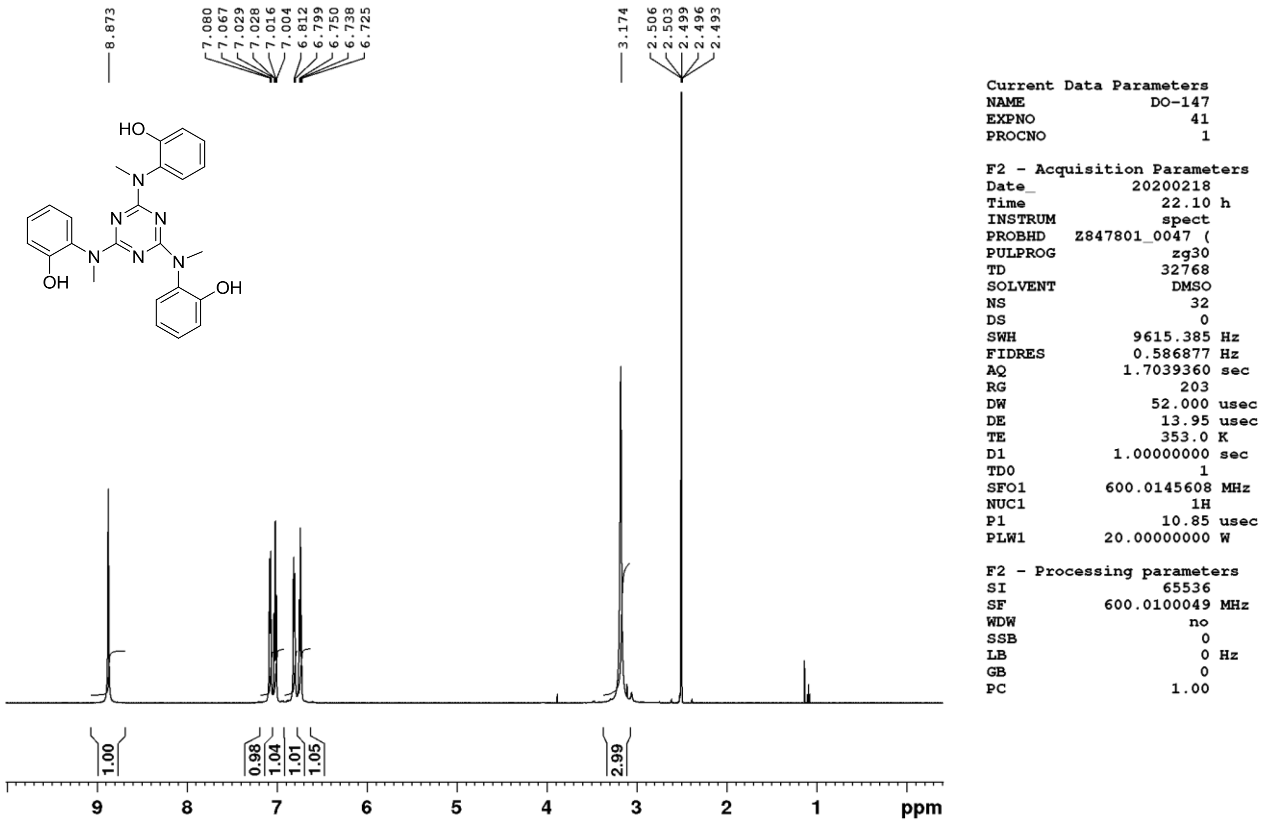

^{13}C NMR (150.9 MHz, DMSO-d6, 353 K):

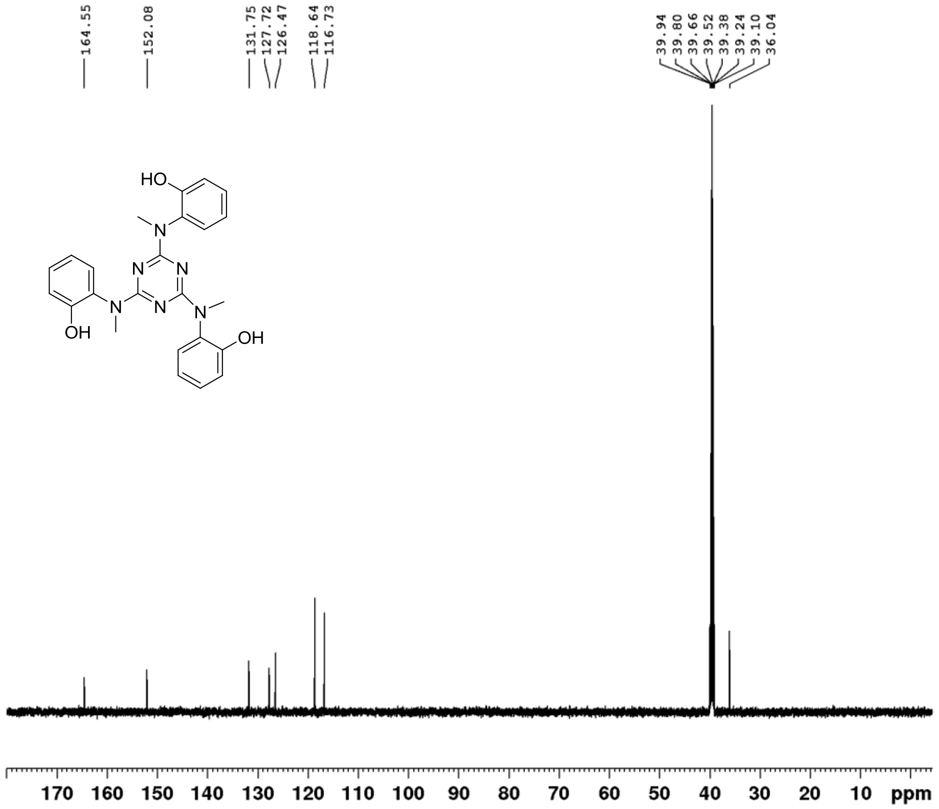


```
Current Data Parameters
NAME             DO-147
EXPNO                44
PROCNO                1

F2 - Acquisition Parameters
Date_          20200218
Time              22.48 h
INSTRUM           spect
PROBHD   Z847801_0047 (
PULPROG          zgdc30
TD                32768
SOLVENT            DMSO
NS                  128
DS                    0
SWH          36057.691 Hz
FIDRES        2.200787 Hz
AQ           0.4543829 sec
RG                 2050
DW               13.867 usec
DE                 6.50 usec
TE                353.2 K
D1           1.50000000 sec
D11          0.03000000 sec
TD0                   1
SFO1        150.8892338 MHz
NUC1                13C
P1                 9.80 usec
PLW1        40.00000000 W
SFO2        600.0124004 MHz
NUC2                 1H
CPDPRG[2        waltz16
PCPD2             90.00 usec
PLW2        20.00000000 W
PLW12        0.33800000 W

F2 - Processing parameters
SI                65536
SF          150.8727707 MHz
WDW                  EM
SSB                   0
LB                 1.00 Hz
GB                    0
PC                 1.40
```

^{1}H NMR (600 MHz, DMF-d7, 293 K):

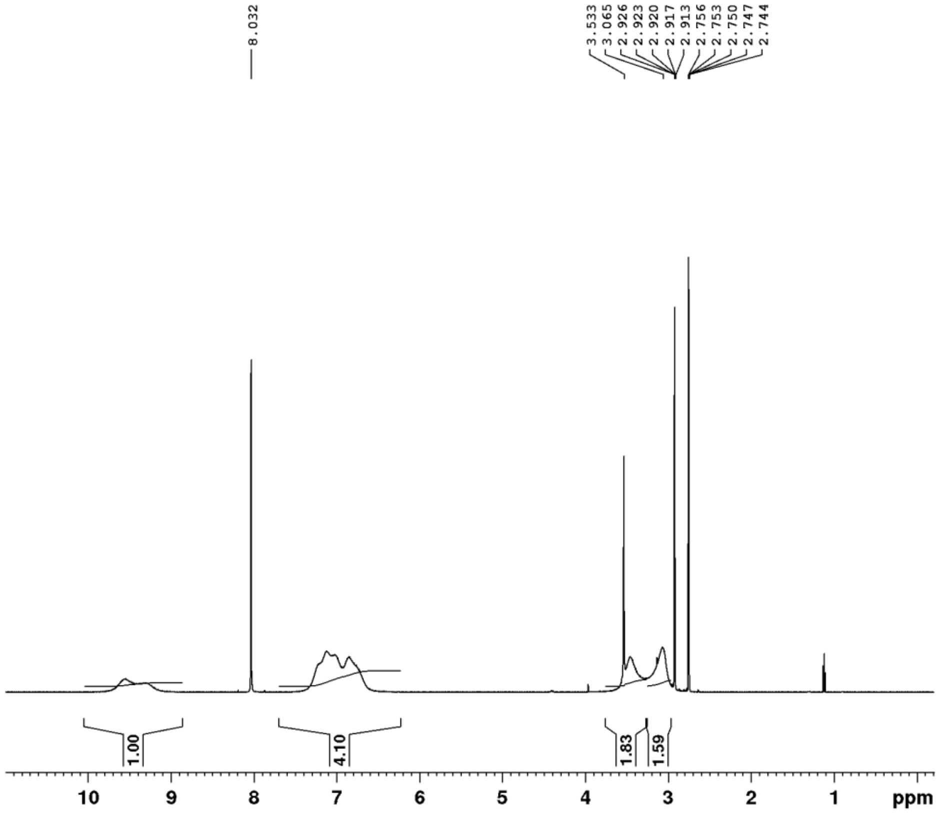


```
Current Data Parameters
NAME         DO-147-DMF
EXPNO                11
PROCNO                1

F2 - Acquisition Parameters
Date_          20200406
Time              11.25 h
INSTRUM           spect
PROBHD   Z847801_0047 (
PULPROG            zg30
TD                65536
SOLVENT             DMF
NS                   32
DS                    0
SWH            9615.385 Hz
FIDRES         0.293438 Hz
AQ            3.4078720 sec
RG                  203
DW               52.000 usec
DE                13.95 usec
TE                293.0 K
D1           3.00000000 sec
TD0                   1
SFO1        600.0145608 MHz
NUC1                 1H
P1                10.85 usec
PLW1        20.00000000 W

F2 - Processing parameters
SI                65536
SF          600.0100088 MHz
WDW                  no
SSB                   0
LB                    0 Hz
GB                    0
PC                 1.00
```

^{1}H NMR (600 MHz, DMF-d7, 233 K):

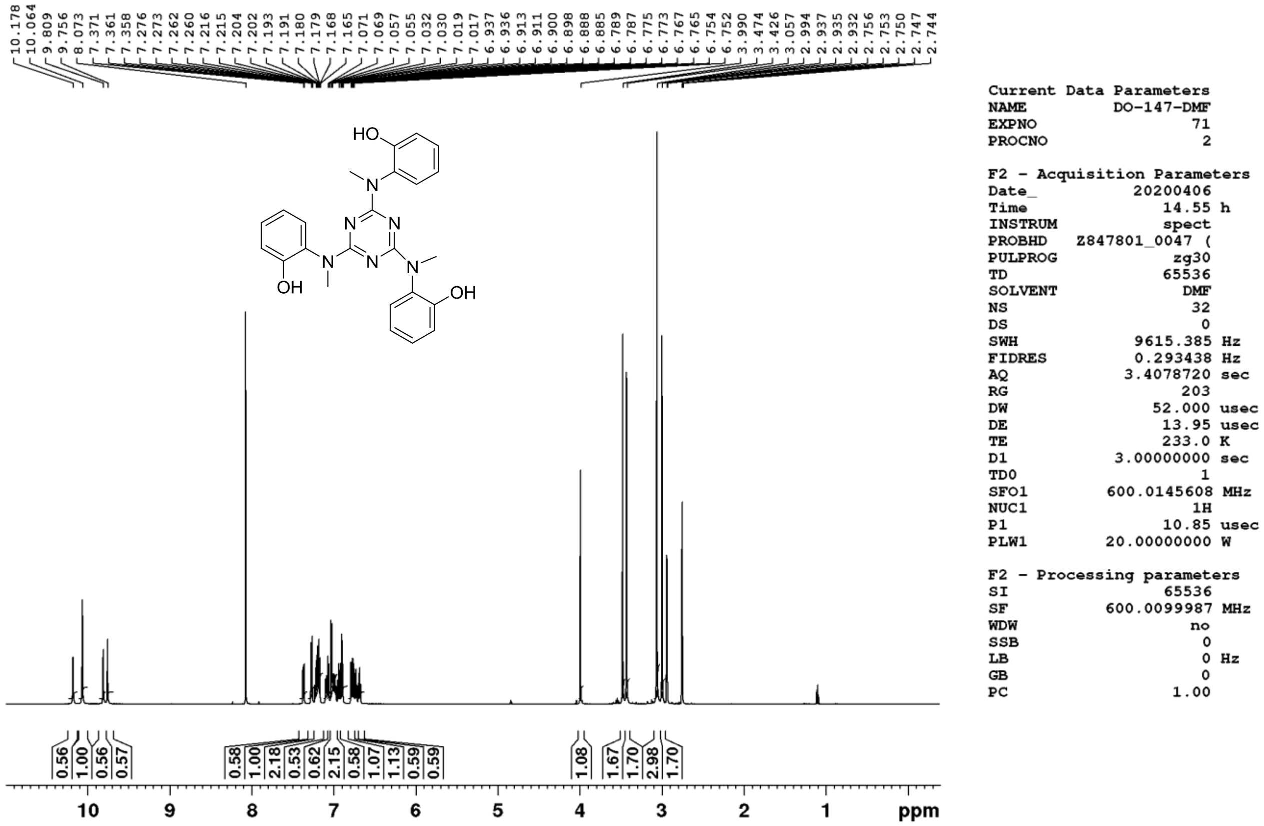

^{13}C NMR (150.9 MHz, DMF-d7, 233 K):

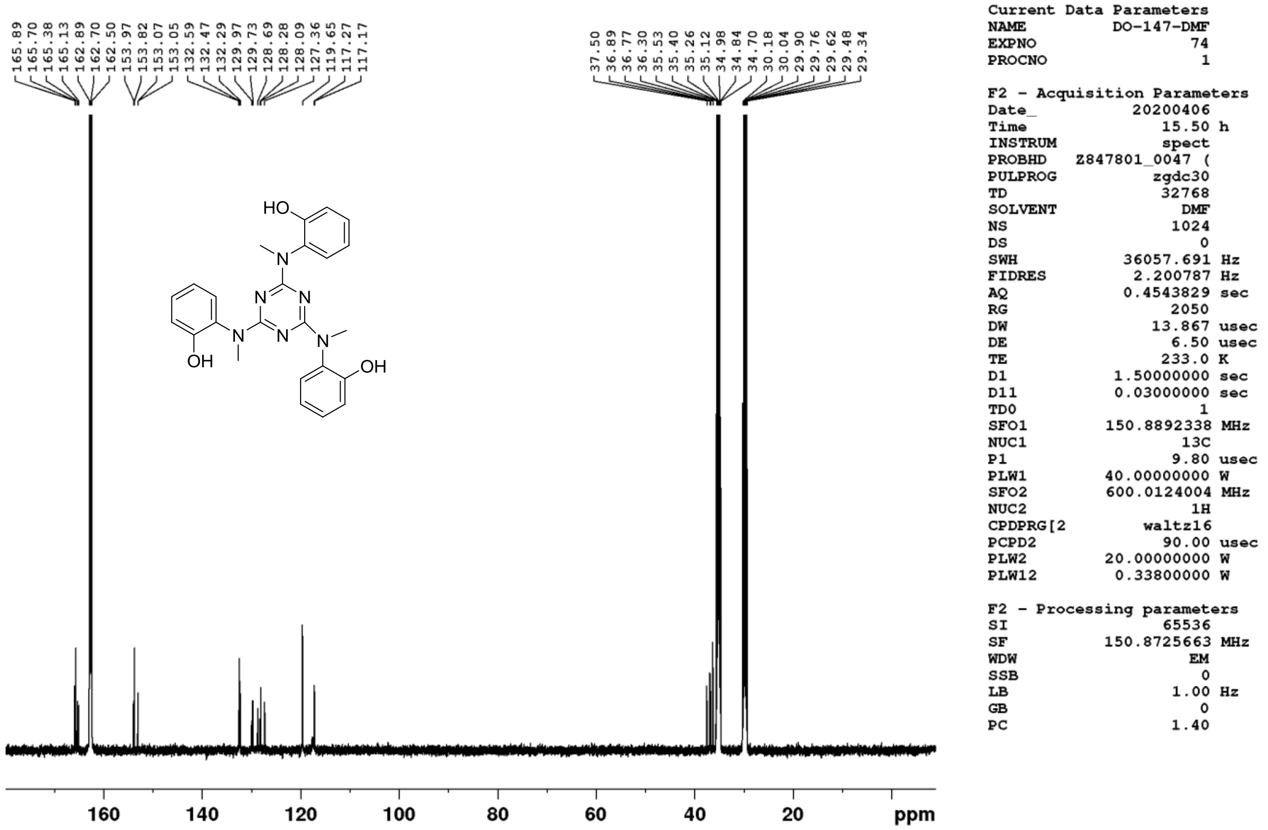

Compound **14**:
^{1}H NMR (600 MHz, DMSO-d6, 293 K):

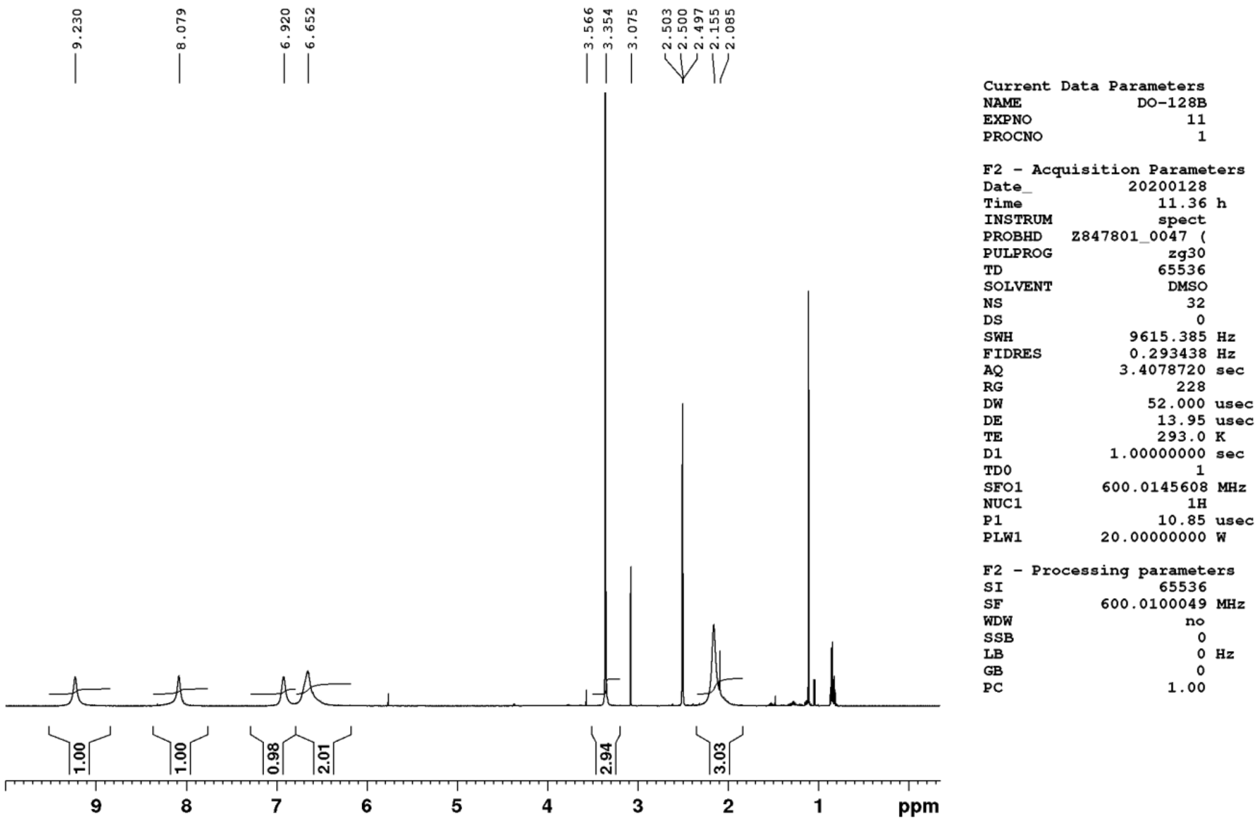

^{1}H NMR (600 MHz, DMSO-d6, 353 K):

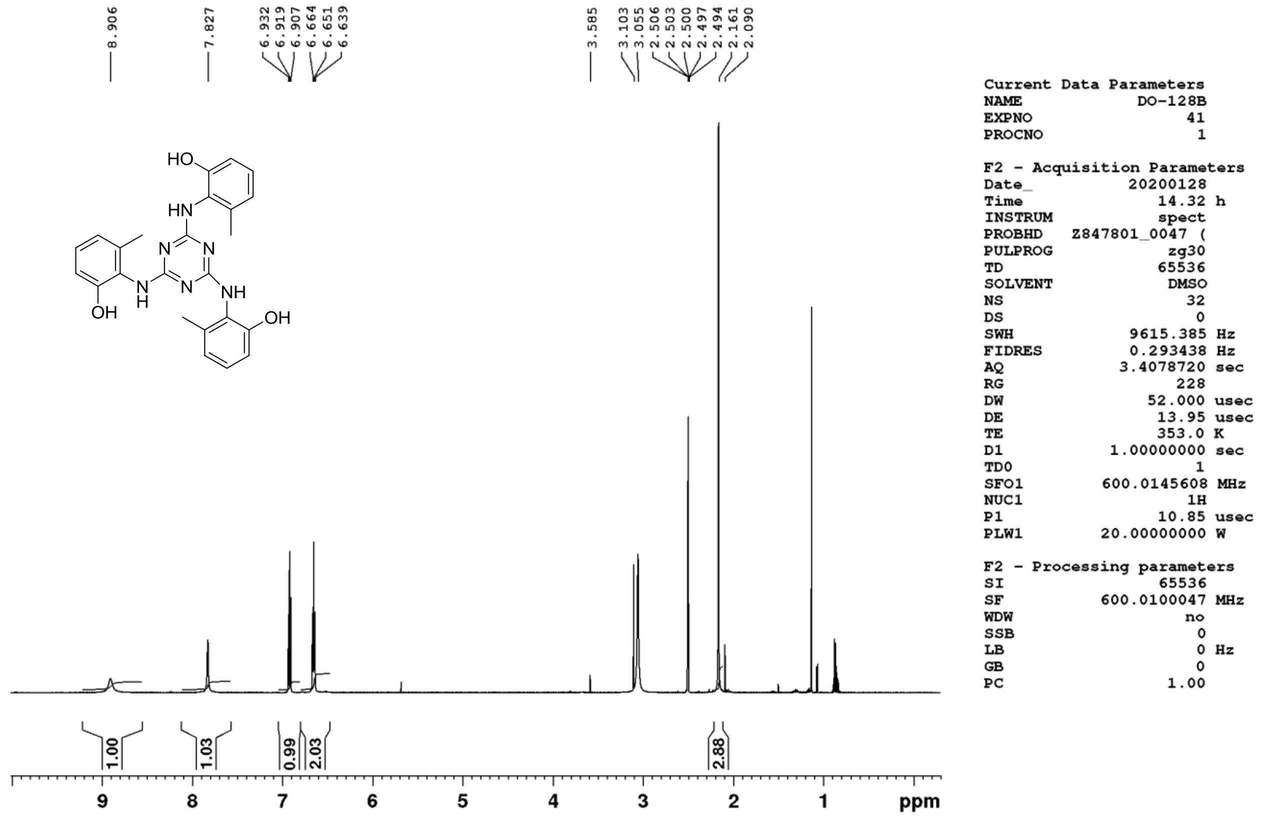

^{13}C NMR (150.9 MHz, DMSO-d6, 353 K):

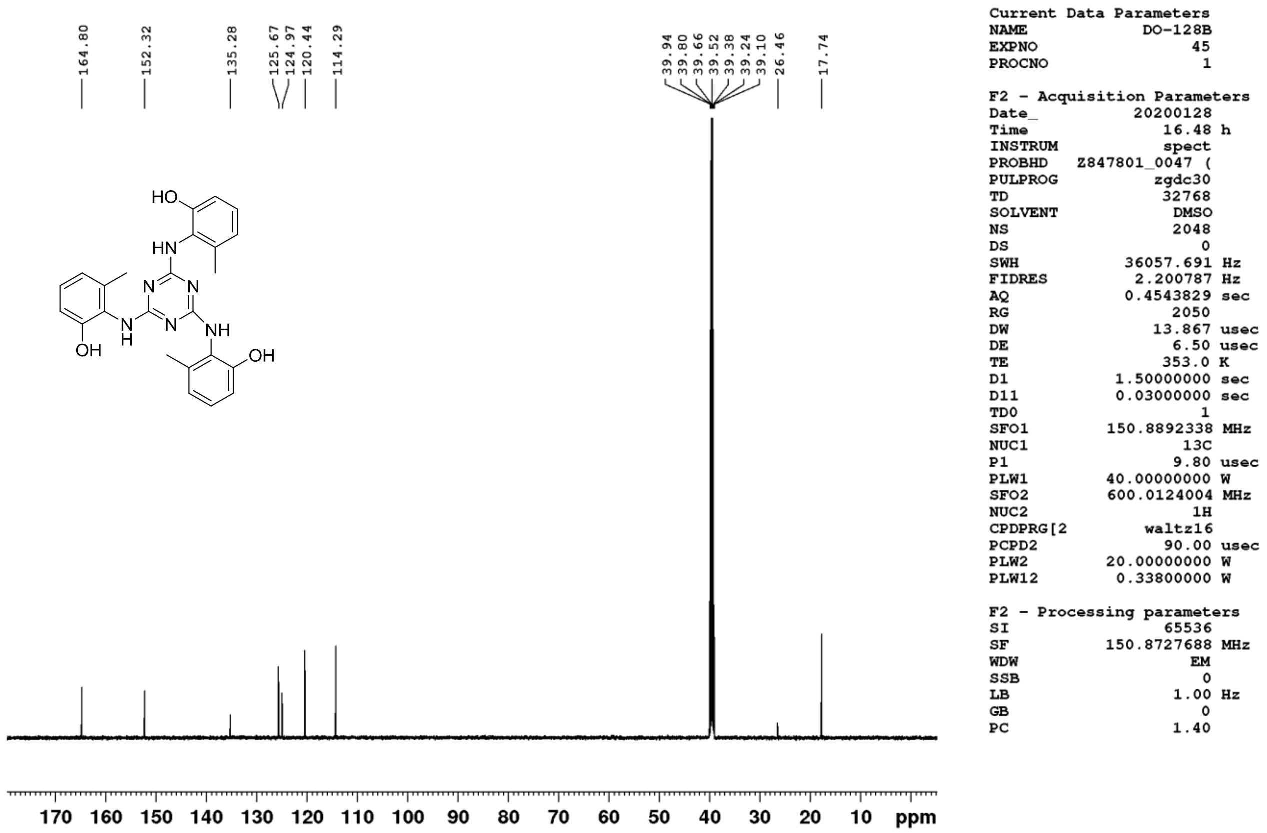

^{1}H NMR (600 MHz, DMF-d7, 293 K):

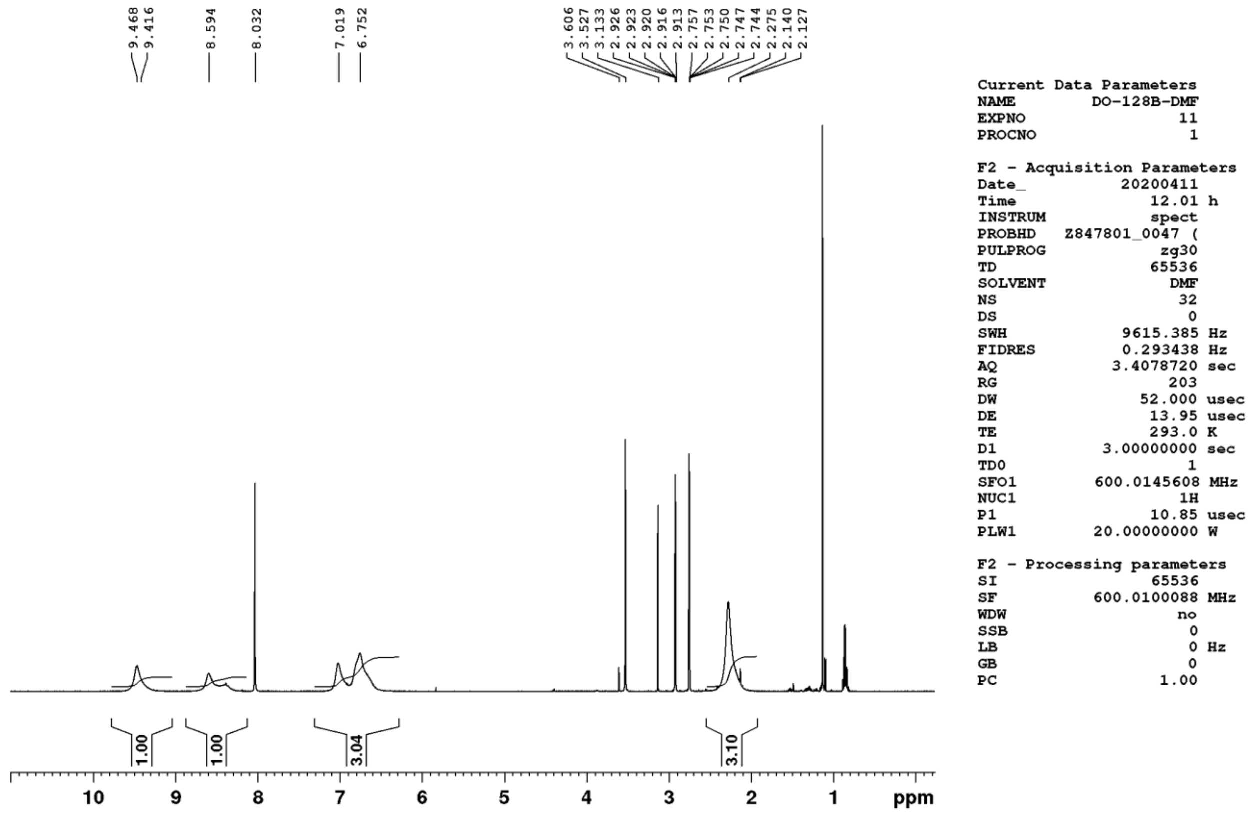

^{1}H NMR (600 MHz, DMF-d7, 233 K):

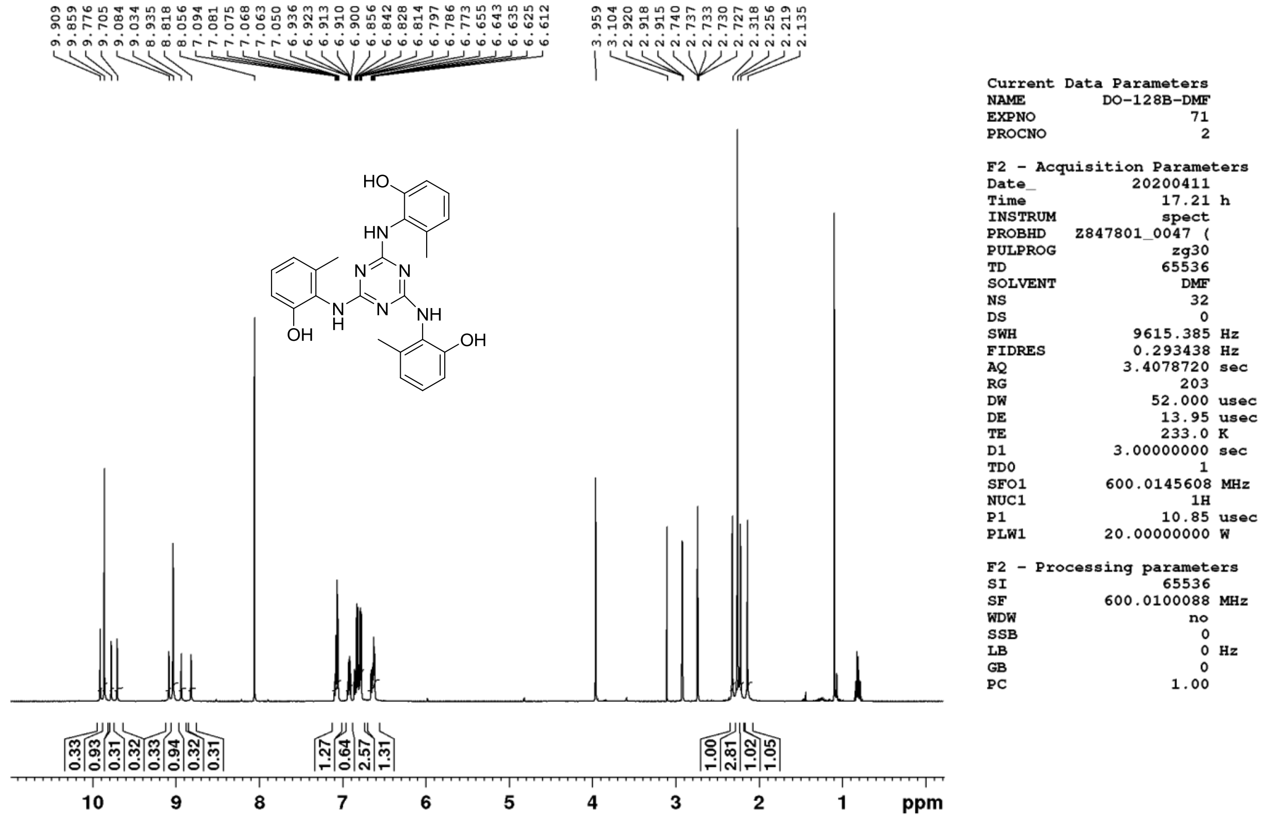

^{13}C NMR (150.9 MHz, DMF-d7, 233 K):

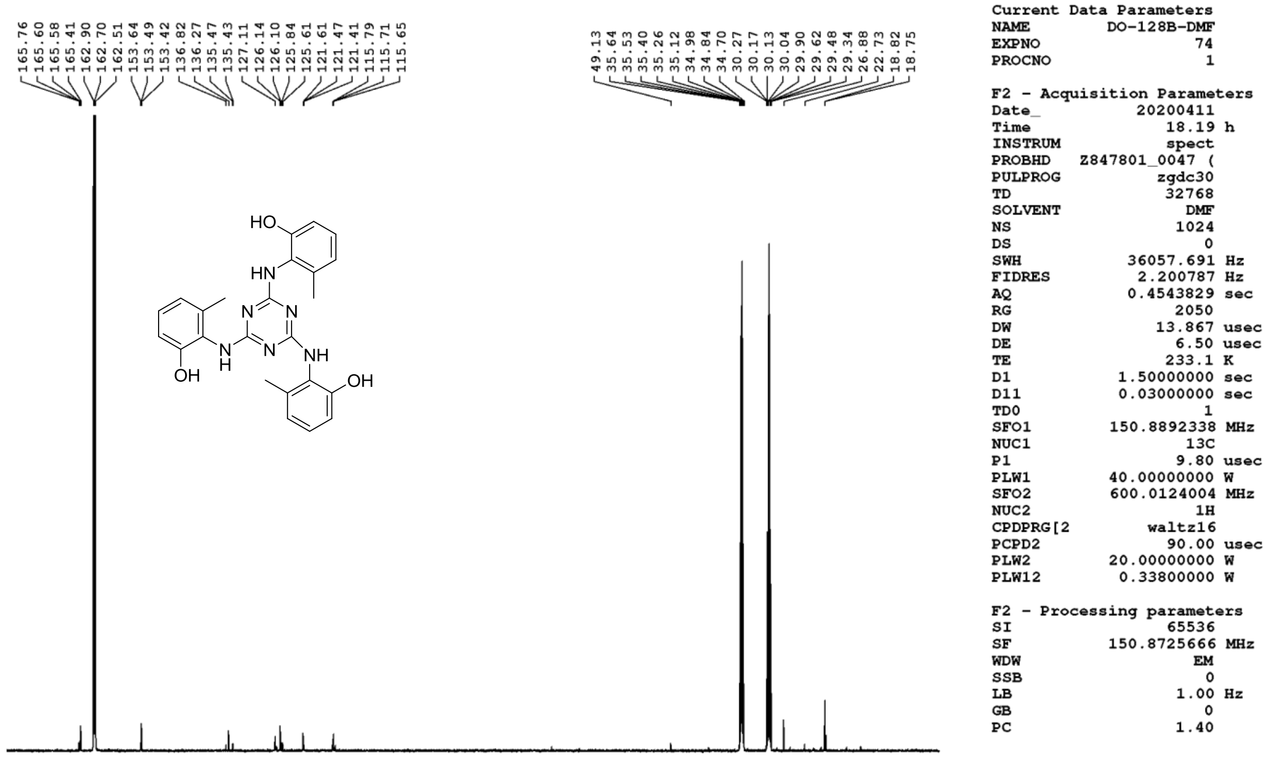

Dynamic NMR spectra

<u>Compound **4**</u>

^1H and ^{13}C <u>spectra</u> were recorded on a Bruker II+ 600 spectrometer (BBO probe) at 600.13 for ^1H NMR and 150.92 MHz for ^{13}C NMR with TMS as internal standard for chemical shifts (δ, ppm). The spectra were recorded in steps of 10 K between 273 and 323 K (0.05M in 600 μL DMF-d7). Temperature calibration was done with B-VT 3000 unit (it was checked and calibrated with methanol and ethylene glycol reference samples). ^1H NMR spectra were acquired using a spectral width of 10 kHz, an acquisition time of 3.4 s and 32 scans, zerofilled to 64k datapoints (0.15 Hz per point) and processed without apodization.

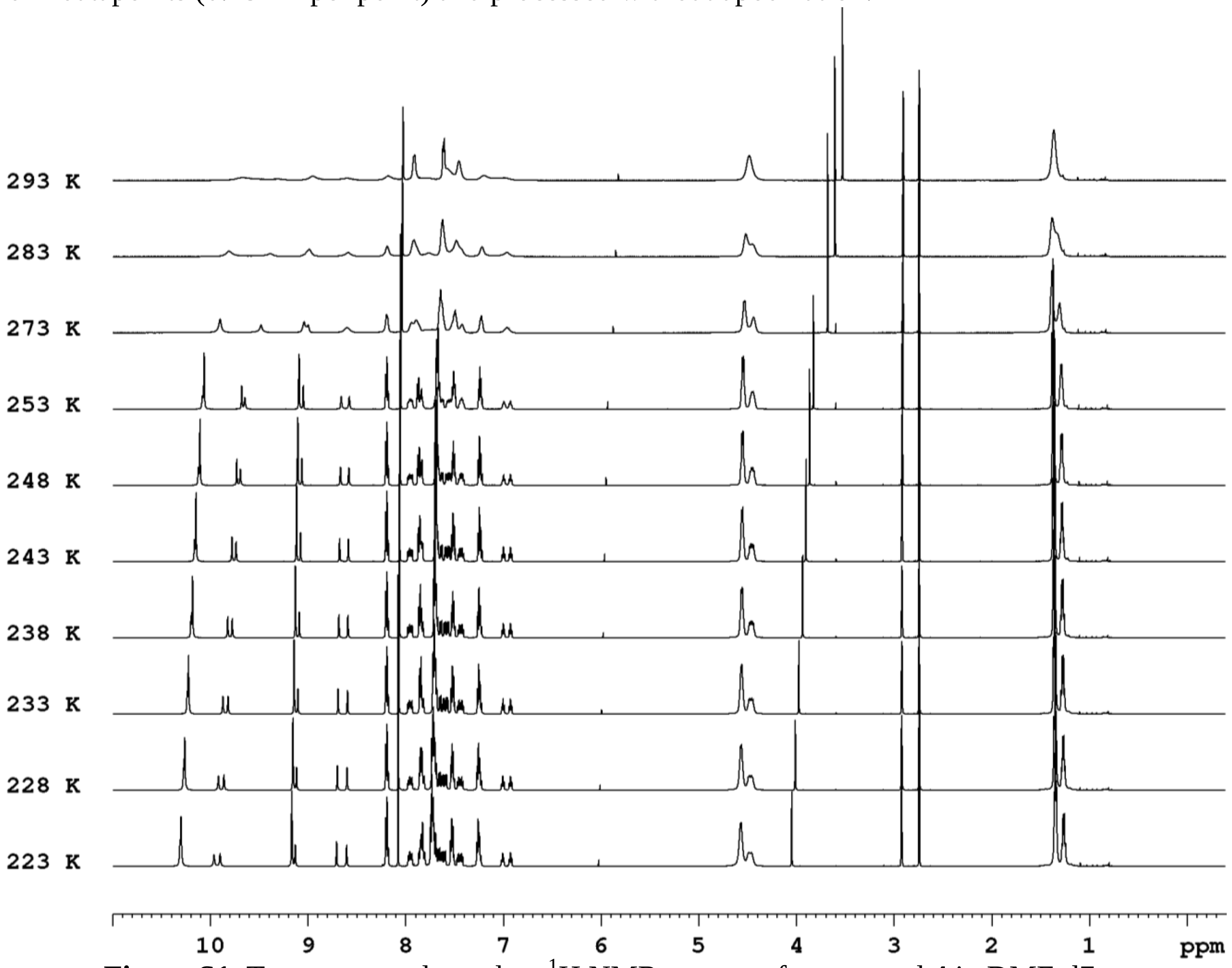

Figure S1. Temperature-dependent ^1H NMR spectra of compound **4** in DMF-d7.

^1H ROESY spectra (roesyph.2) were recorded on a BBO probe in steps of 5 K between 223 and 253 K. The spectra were acquired using a spectral width of 3.0 kHz, 2048 x 256 complex time domain datapoints, mixing times in the range of 0.03 to 0.1 s and 8 scans in about 75 min. The spectra were zerofilled to 4096 x 4096 datapoints and processed with a shifted square sine bell apodization in both dimensions. Populations and exchange rates were obtained from diagonal- and crosspeak integrals using EXSYCalc (MestreLab Research S.L.).

In the ^1H ROESY spectra of **4** the intensity of following peaks were calculated by volume integration in order to obtain the rate constants of *asym* to *sym* exchange:

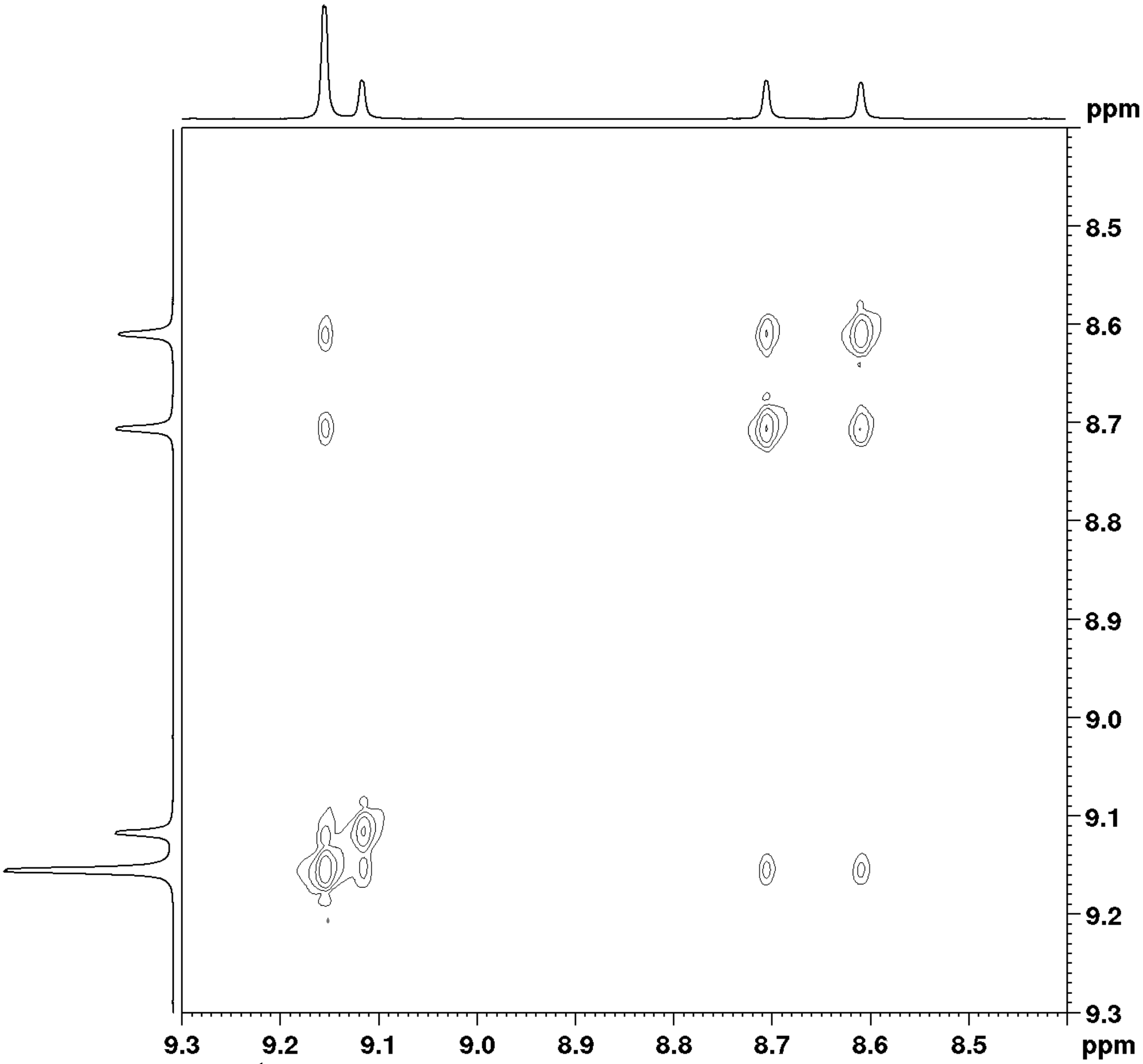

Figure S2. ^{1}H ROESY spectrum of compound **4** in DMF-d7 at 233K using mixing time of 0.3 s (region of carbazol-H-4 protons).

In the ^{1}H ROESY spectra of **4** the intensity of following peaks were calculated by volume integration:

	Sym	*Asym*	*Asym*	*Asym*
Sym	I_{11}	I_{12}	I_{13}	I_{14}
Asym	I_{21}	I_{22}	I_{23}	I_{24}
Asym	I_{31}	I_{32}	I_{33}	I_{34}
Asym	I_{41}	I_{42}	I_{43}	I_{44}

It can be transformed into:

	Sym	*Asym*
Sym	I_{11}	$I_{12} + I_{13} + I_{14}$
Asym	$I_{21} + I_{31} + I_{41}$	$I_{22} + I_{23} + I_{24} + I_{32} + I_{33} + I_{34} + I_{42} + I_{43} + I_{44}$

Table S1. Rate constants of compound **4** calculated from 2D integrals

T, K	*Asym* to *Sym*	*Sym* to *Asym*
223	0.076	0.092
228	0.142	0.174
233	0.296	0.292
238	0.657	0.555
243	1.202	1.145
248	2.659	2.435
253	5.465	5.086

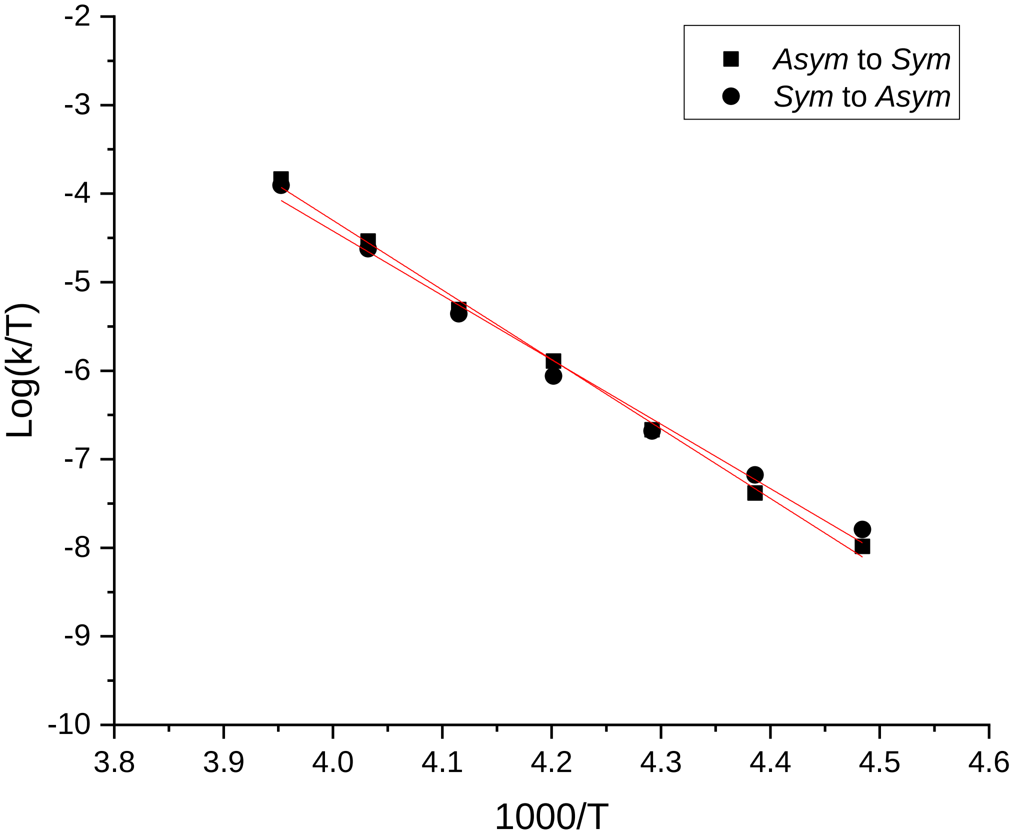

Figure S3. Eyring plot of rate constants of compound **4**

Errors analysis: Usually the presented errors in activation parameters are the statistical errors based on scattering of the data points around the Eyring straight line only. The errors in this analysis are due to inaccuracies in both the calculated rate constants, ***k*,** and the measured temperatures, ***T*** and are computed according to the error propagation equations of Binsch [1] and Heinzer and Oth [2]. The absolute error in temperature is assumed to be not more than ±0.5 K. The relative errors in ***k*** are estimated to be not more than ±10% at all temperatures according the precision of the volume integration of peaks. The errors analysis was performed using self-made computer program using the cited equations.

Table S2. Experimental thermodynamic parameters of exchange processes in compound **4** in DMF-d7

Exchange	$\Delta H^{\neq}$(298K)	$\Delta S^{\neq}$(298K)	$\Delta G^{\neq}$(298K)	R^2
Asym to *Sym*	15.6 ± 0.7	6.6 ± 3.1	13.6 ± 0.1	0.998
Sym to *Asym*	14.4 ± 0.8	1.8 ± 3.0	13.9 ± 0.1	0.995

$\Delta G^{\neq}$ and $\Delta H^{\neq}$ in kcal mol^{-1} and $\Delta S^{\neq}$ in cal mol^{-1} K^{-1}

Table S3. Experimental Thermodynamic parameters of compound **4** in DMF-d7

Conformer	ΔH^0 (298K)	ΔS^0 (298K)	ΔG^0 (298K)
Asym	0.00	0.00	0.00
Sym	0.9 ± 0.03	4.0 ± 0.1	-0.31 ± 0.0

$\Delta H^0 = H^0$ (*Sym*) - H^0 (*Asym*)
$\Delta S^0 = S^0$ (*Sym*) - S^0 (*Asym*)
$\Delta G^0 = G^0$ (*Sym*) - G^0 (*Asym*)
ΔG^0 and ΔH^0 in kcal mol^{-1} and ΔS^0 in cal mol^{-1} K^{-1}

Compound **5**

^{1}H and ^{13}C spectra were recorded on a Bruker II+ 600 spectrometer (BBO probe) at 600.13 for ^{1}H NMR and 150.92 MHz for ^{13}C NMR with TMS as internal standard for chemical shifts (δ, ppm). The spectra were recorded in steps of 10 K between 223 and 293 K (0.025M in 600 μL DMF-d7). Temperature calibration was done with B-VT 3000 unit (it was checked and calibrated with methanol and ethylene glycol reference samples). ^{1}H NMR spectra were acquired using a spectral width of 10 kHz, an acquisition time of 3.4 s and 32 scans, zerofilled to 64k datapoints (0.15 Hz per point) and processed without apodization.

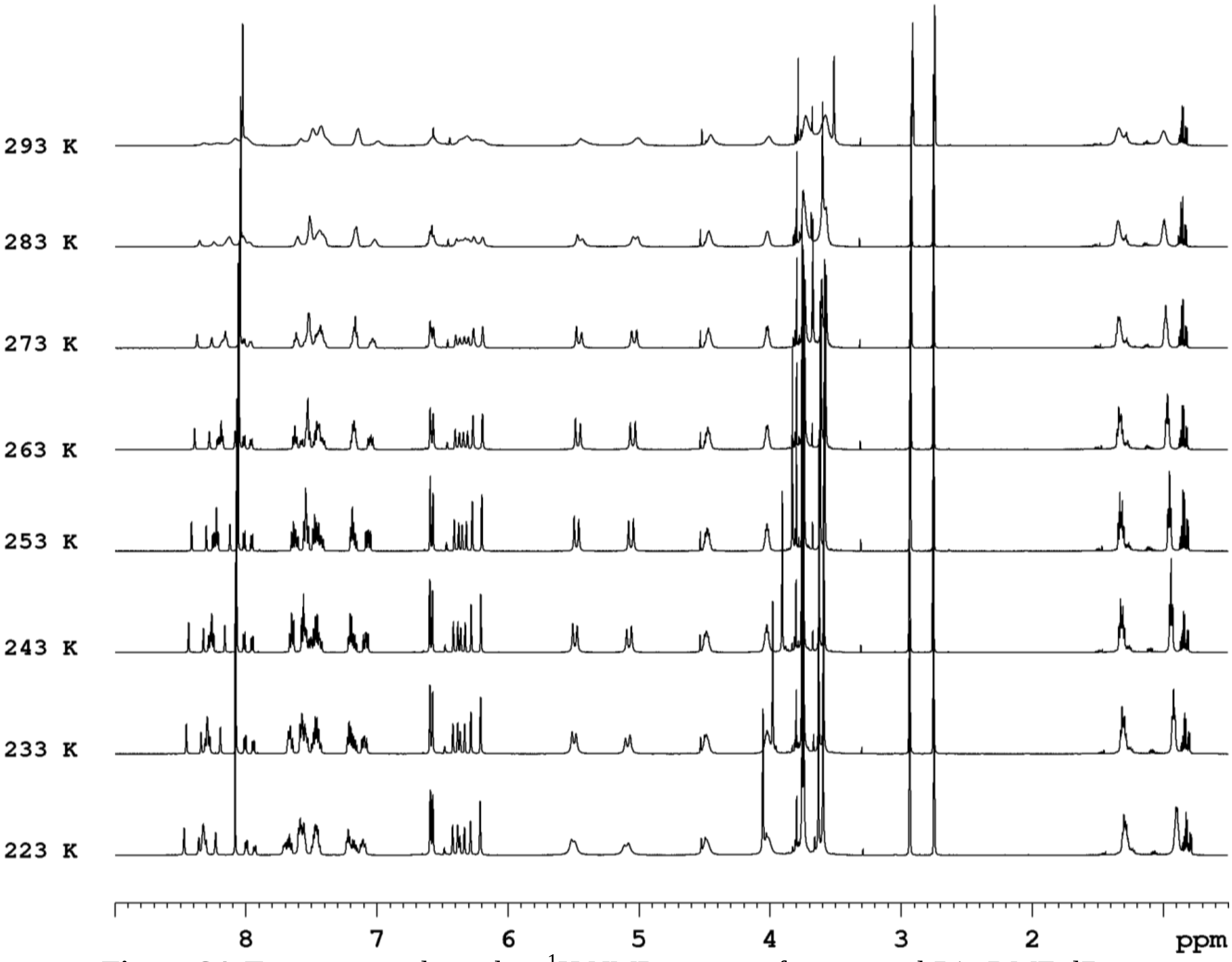

Figure S4. Temperature-dependent ^{1}H NMR spectra of compound **5** in DMF-d7.

^{1}H ROESY spectra (roesyph.2) were recorded on a BBO probe in steps of 5 K between 223 and 253 K. The spectra were acquired using a spectral width of 3.0 kHz, 2048 x 256 complex time domain datapoints, mixing times in the range of 0.03 to 0.1 s and 8 scans in about 75 min. The spectra were zerofilled to 4096 x 4096 datapoints and processed with a shifted square sine bell apodization in both dimensions. Populations and exchange rates were obtained from diagonal- and crosspeak integrals using EXSYCalc (MestreLab Research S.L.).

In the ^{1}H ROESY spectra of **5** the intensity of following peaks were calculated by volume integration in order to obtain the rate constants of *asym* to *sym* exchange:

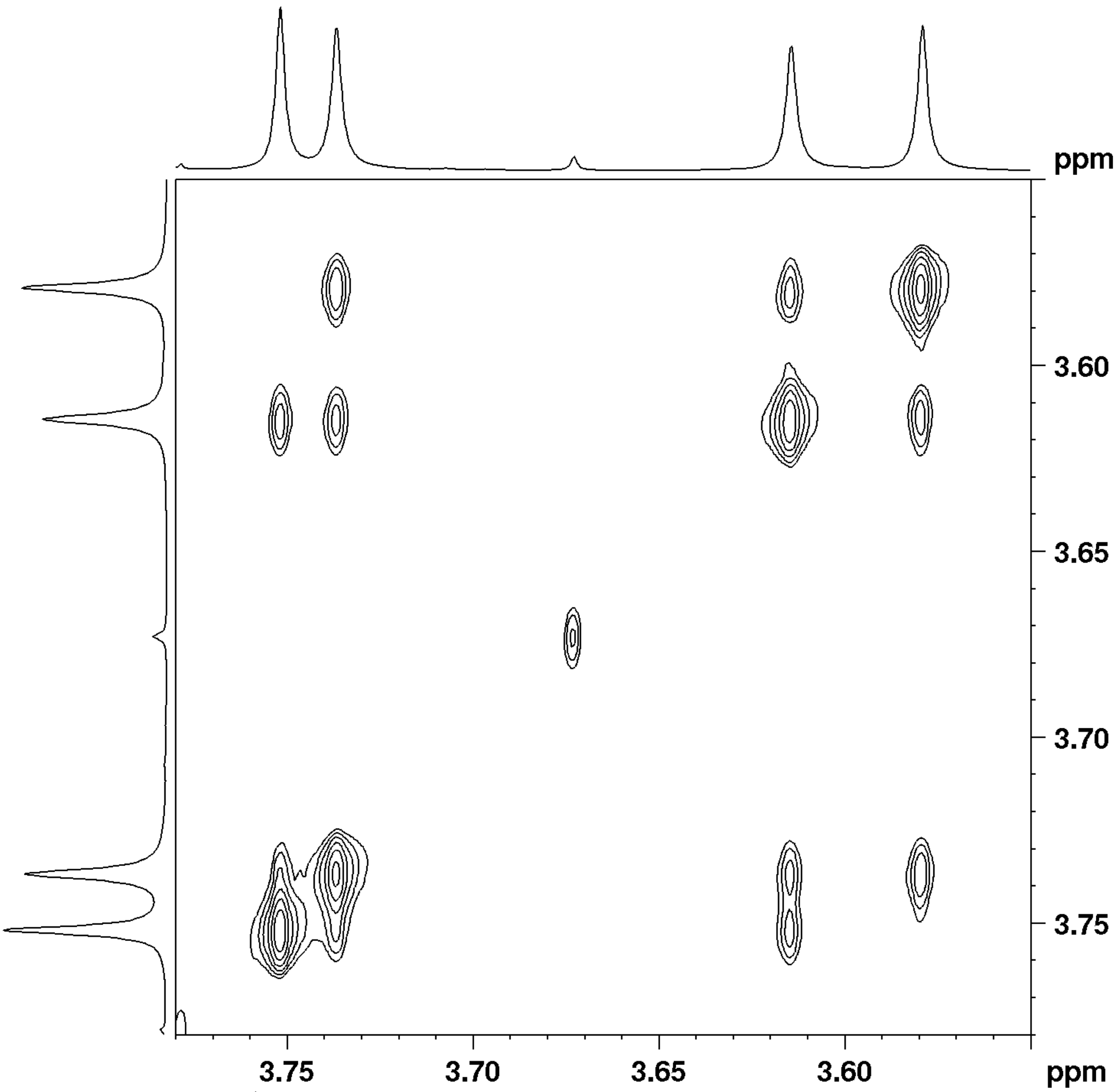

Figure S5. ^{1}H ROESY spectrum of compound **5** in DMF-d7 at 253K using mixing time of 0.3 s (region of N-CH_3 protons).

In the ^{1}H ROESY spectra of **5** the intensity of following peaks were calculated by volume integration:

	Asym	*Asym*	*Sym*	*Asym*
Asym	I_{11}	I_{12}	I_{13}	I_{14}
Asym	I_{21}	I_{22}	I_{23}	I_{24}
Sym	I_{31}	I_{32}	I_{33}	I_{34}
Asym	I_{41}	I_{42}	I_{43}	I_{44}

It can be transformed into:

	Sym	*Asym*
Sym	I_{33}	$I_{31} + I_{32} + I_{34}$
Asym	$I_{13} + I_{23} + I_{43}$	$I_{11} + I_{12} + I_{14} + I_{21} + I_{22} + I_{24} + I_{41} + I_{42} + I_{44}$

Table S4. Rate constants of compound **5** calculated from 2D integrals

T, K	*Asym* to *Sym*	*Sym* to *Asym*
233	0.035	0.140
243	0.148	0.506
248	0.320	0.996
253	0.633	1.935

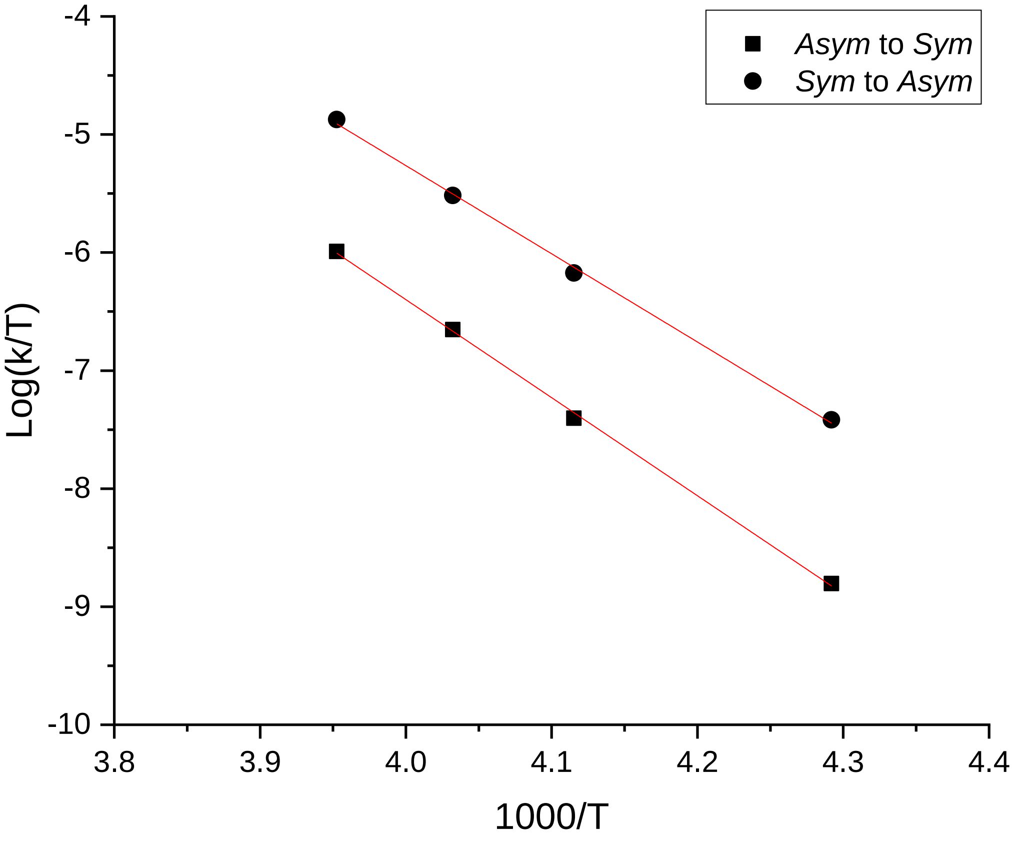

Figure S6. Eyring plot of rate constants of compound **5**

Errors analysis: Usually the presented errors in activation parameters are the statistical errors based on scattering of the data points around the Eyring straight line only. The errors in this analysis are due to inaccuracies in both the calculated rate constants, ***k,*** and the measured temperatures, ***T*** and are computed according to the error propagation equations of Binsch [1] and Heinzer and Oth [2]. The absolute error in temperature is assumed to be not more than ±0.5 K. The relative errors in ***k*** are estimated to be not more than ±10% at all temperatures according the precision of the volume integration of peaks. The errors analysis was performed using self-made computer program using the cited equations.

Table S5. Experimental thermodynamic parameters of exchange processes in compound **5** in DMF-d7

Exchange	$\Delta H^{\neq}$(298K)	$\Delta S^{\neq}$(298K)	$\Delta G^{\neq}$(298K)	R^2
Asym to *Sym*	16.5 ± 1.0	6.1 ± 4.4	14.7 ± 0.1	0.9997
Sym to *Asym*	14.8 ± 1.0	1.7 ± 3.7	14.3 ± 0.1	0.9993

$\Delta G^{\neq}$ and $\Delta H^{\neq}$ in kcal mol^{-1} and $\Delta S^{\neq}$ in cal mol^{-1} K^{-1}

Table S6. Experimental Thermodynamic parameters of compound **5** in DMF-d7

Conformer	ΔH^0 (298K)	ΔS^0 (298K)	ΔG^0 (298K)
Asym	0.00	0.00	0.00
Sym	-1.5 ± 0.0	-3.7 ± 0.1	-0.41 ± 0.0

$\Delta H^0 = H^0$ (*Sym*) - H^0 (*Asym*)

$\Delta S^0 = S^0$ (*Sym*) - S^0 (*Asym*)

$\Delta G^0 = G^0$ (*Sym*) - G^0 (*Asym*)

ΔG^0 and ΔH^0 in kcal mol^{-1} and ΔS^0 in cal mol^{-1} K^{-1}

Compound **6**

^{1}H and ^{13}C spectra were recorded on a Bruker II+ 600 spectrometer (BBO probe) at 600.13 for ^{1}H NMR and 150.92 MHz for ^{13}C NMR with TMS as internal standard for chemical shifts (δ, ppm). The spectra were recorded in steps of 10 K between 223 and 293 K (0.05M in 600 μL DMF-d7). Temperature calibration was done with B-VT 3000 unit (it was checked and calibrated with methanol and ethylene glycol reference samples). ^{1}H NMR spectra were acquired using a spectral width of 10 kHz, an acquisition time of 3.4 s and 32 scans, zerofilled to 64k datapoints (0.15 Hz per point) and processed without apodization.

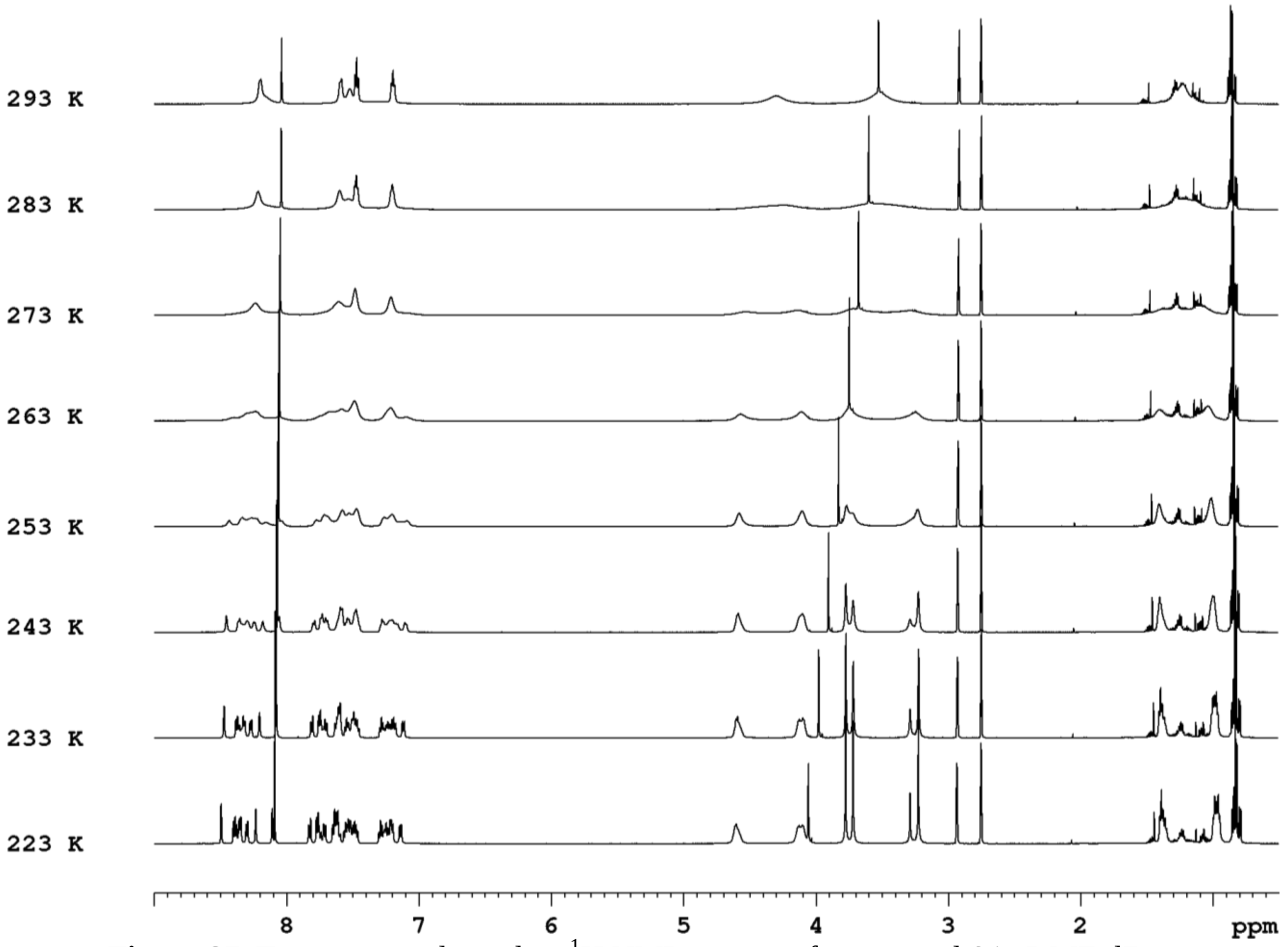

Figure S7. Temperature-dependent ^{1}H NMR spectra of compound **6** in DMF-d7.

^{1}H ROESY spectra (roesyph.2) were recorded on a BBO probe in steps of 5 K between 223 and 243 K. The spectra were acquired using a spectral width of 3.0 kHz, 2048 x 256 complex time domain datapoints, mixing times in the range of 0.03 to 0.1 s and 8 scans in about 75 min. The spectra were zerofilled to 4096 x 4096 datapoints and processed with a shifted square sine bell apodization in both dimensions. Populations and exchange rates were obtained from diagonal- and crosspeak integrals using EXSYCalc (MestreLab Research S.L.).

In the ^{1}H ROESY spectra of **6** the intensity of following peaks were calculated by volume integration in order to obtain the rate constants of *asym* to *sym* exchange:

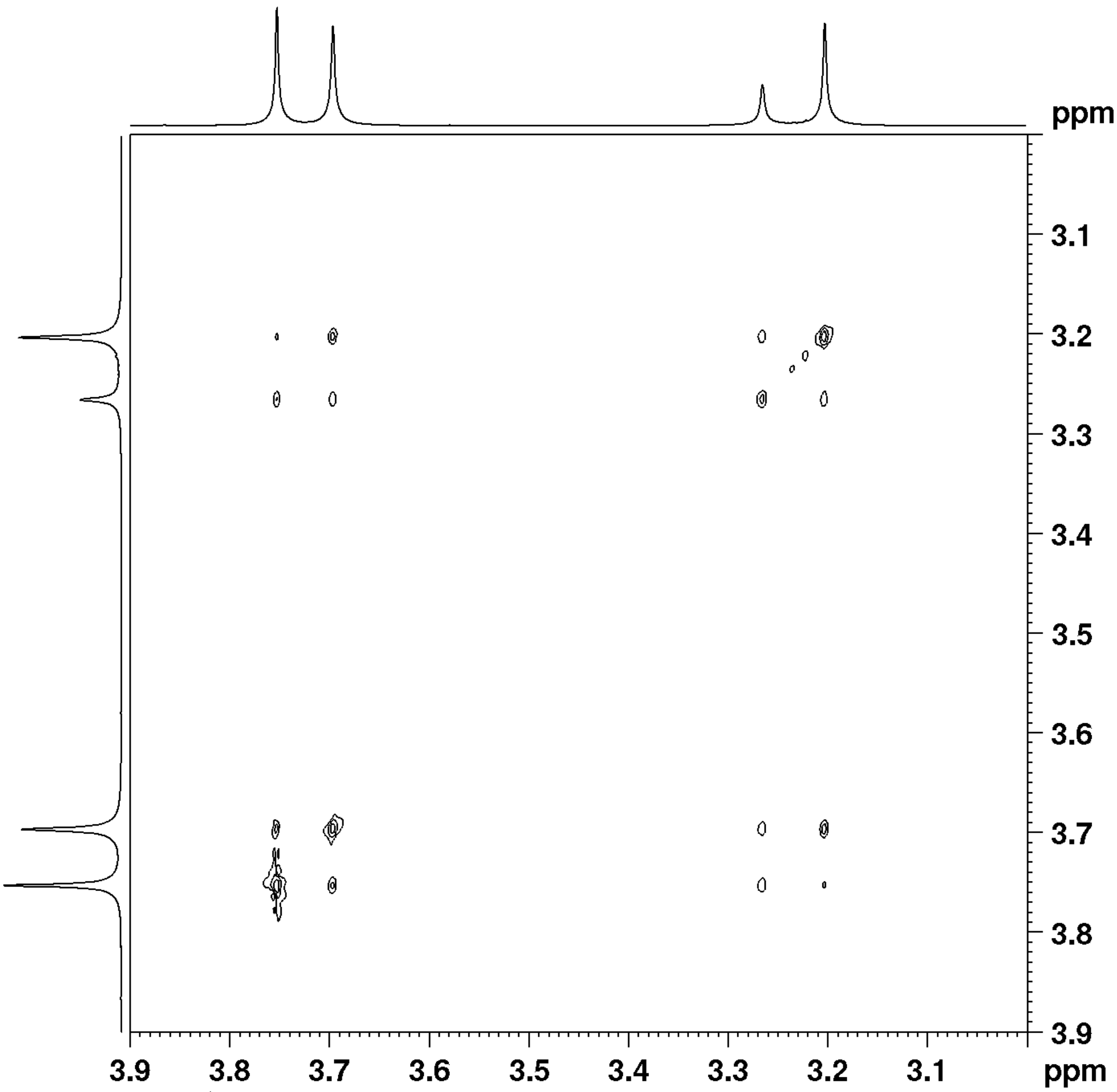

Figure S8. ^{1}H ROESY spectrum of compound **6** in DMF-d7 at 223K using mixing time of 0.3 s (region of OCH_3 protons).

In the ^{1}H ROESY spectra of **6** the intensity of following peaks were calculated by volume integration:

	Asym	*Asym*	*Sym*	*Asym*
Asym	I_{11}	I_{12}	I_{13}	I_{14}
Asym	I_{21}	I_{22}	I_{23}	I_{24}
Sym	I_{31}	I_{32}	I_{33}	I_{34}
Asym	I_{41}	I_{42}	I_{43}	I_{44}

It can be transformed into:

	Sym	*Asym*
Sym	I_{33}	$I_{31} + I_{32} + I_{34}$
Asym	$I_{13} + I_{23} + I_{43}$	$I_{11} + I_{12} + I_{14} + I_{21} + I_{22} + I_{24} + I_{41} + I_{42} + I_{44}$

Table S7. Rate constants of compound **6** calculated from 2D integrals

T, K	*Asym* to *Sym*	*Sym* to *Asym*
223	0.535	3.160
228	1.161	6.345
233	2.493	13.429
238	5.049	26.646
243	10.029	50.966

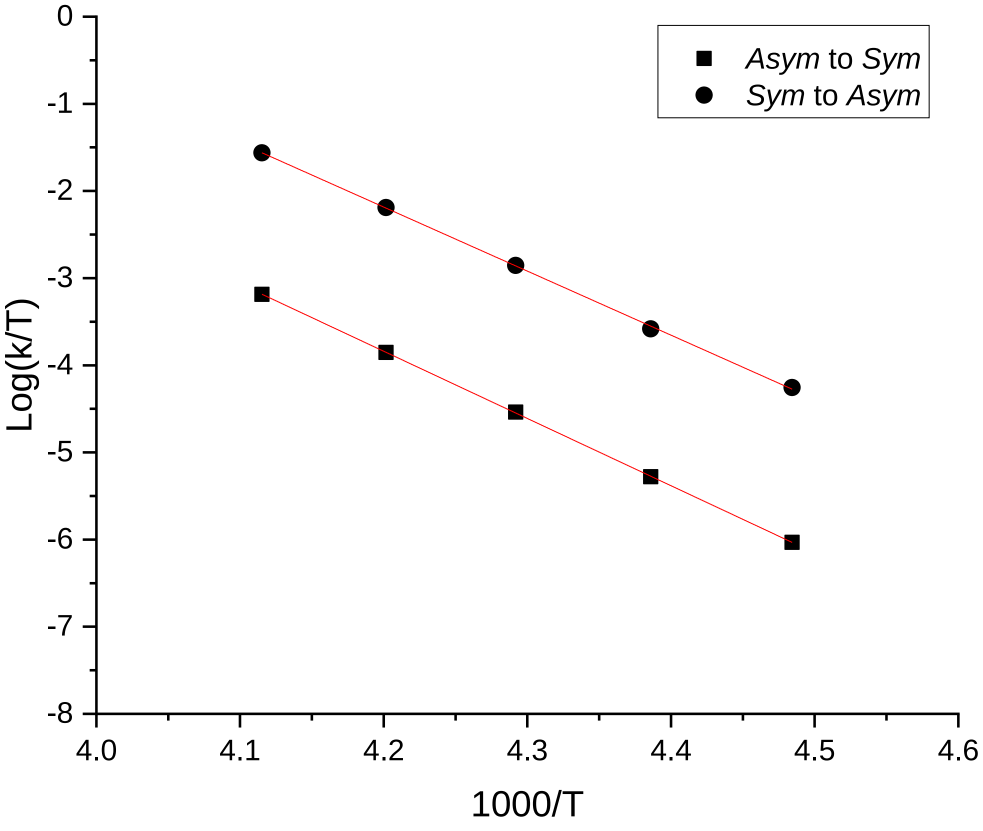

Figure S9. Eyring plot of rate constants of compound **6**

Errors analysis: Usually the presented errors in activation parameters are the statistical errors based on scattering of the data points around the Eyring straight line only. The errors in this analysis are due to inaccuracies in both the calculated rate constants, ***k,*** and the measured temperatures, ***T*** and are computed according to the error propagation equations of Binsch [1] and Heinzer and Oth [2]. The absolute error in temperature is assumed to be not more than ±0.5 K. The relative errors in ***k*** are estimated to be not more than ±10% at all temperatures according the precision of the volume integration of peaks. The errors analysis was performed using self-made computer program using the cited equations.

Table S8. Experimental thermodynamic parameters of exchange processes in compound **6** in DMF-d7

Exchange	$\Delta H^{\neq}$(298K)	$\Delta S^{\neq}$(298K)	$\Delta G^{\neq}$(298K)	R^2
Asym to *Sym*	15.3 ± 1.0	9.6 ± 4.1	12.5 ± 0.1	0.999
Sym to *Asym*	14.6 ± 0.9	9.8 ± 4.2	11.7 ± 0.1	0.999

$\Delta G^{\neq}$ and $\Delta H^{\neq}$ in kcal mol^{-1} and $\Delta S^{\neq}$ in cal mol^{-1} K^{-1}

Table S9. Experimental Thermodynamic parameters of compound **6** in DMF-d7

Conformer	ΔH^0 (298K)	ΔS^0 (298K)	ΔG^0 (298K)
Asym	0.00	0.00	0.00
Sym	-0.6 ± 0.1	0.9 ± 0.4	-0.8 ± 0.0

$\Delta H^0 = H^0$ (*Sym*) - H^0 (*Asym*)
$\Delta S^0 = S^0$ (*Sym*) - S^0 (*Asym*)
$\Delta G^0 = G^0$ (*Sym*) - G^0 (*Asym*)
ΔG^0 and ΔH^0 in kcal mol^{-1} and ΔS^0 in cal mol^{-1} K^{-1}

Compound **11**

^{1}H and ^{13}C spectra were recorded on a Bruker II+ 600 spectrometer (BBO probe) at 600.13 for ^{1}H NMR and 150.92 MHz for ^{13}C NMR with TMS as internal standard for chemical shifts (δ, ppm). The spectra were recorded in steps of 10 K between 223 and 293 K (0.05M in 600 µL DMF-d7). Temperature calibration was done with B-VT 3000 unit (it was checked and calibrated with methanol and ethylene glycol reference samples). ^{1}H NMR spectra were acquired using a spectral width of 10 kHz, an acquisition time of 3.4 s and 32 scans, zerofilled to 64k datapoints (0.15 Hz per point) and processed without apodization.

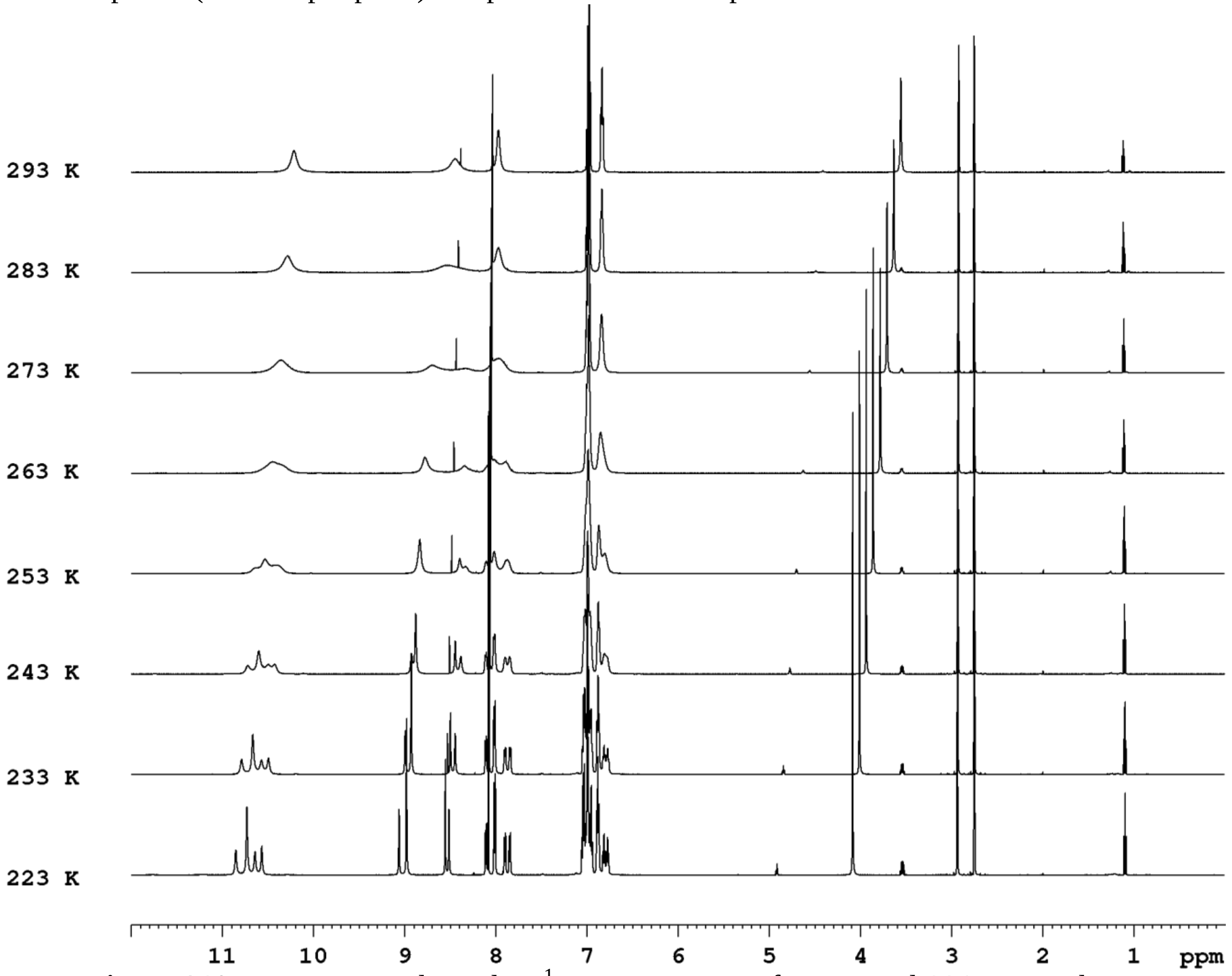

Figure S10. Temperature-dependent ^{1}H NMR spectra of compound **11** in DMF-d7.

^{1}H ROESY spectra (roesyph.2) were recorded on a BBO probe in steps of 5 K between 228 and 238 K. The spectra were acquired using a spectral width of 3.0 kHz, 2048 x 256 complex time domain datapoints, mixing times in the range of 0.05 to 0.3 s and 8 scans in about 75 min. The spectra were zerofilled to 4096 x 4096 datapoints and processed with a shifted square sine bell apodization in both dimensions. Populations and exchange rates were obtained from diagonal- and crosspeak integrals using EXSYCalc (MestreLab Research S.L.).

In the ^{1}H ROESY spectra of **11** the intensity of following peaks were calculated by volume integration in order to obtain the rate constants of *asym* to *sym* exchange:

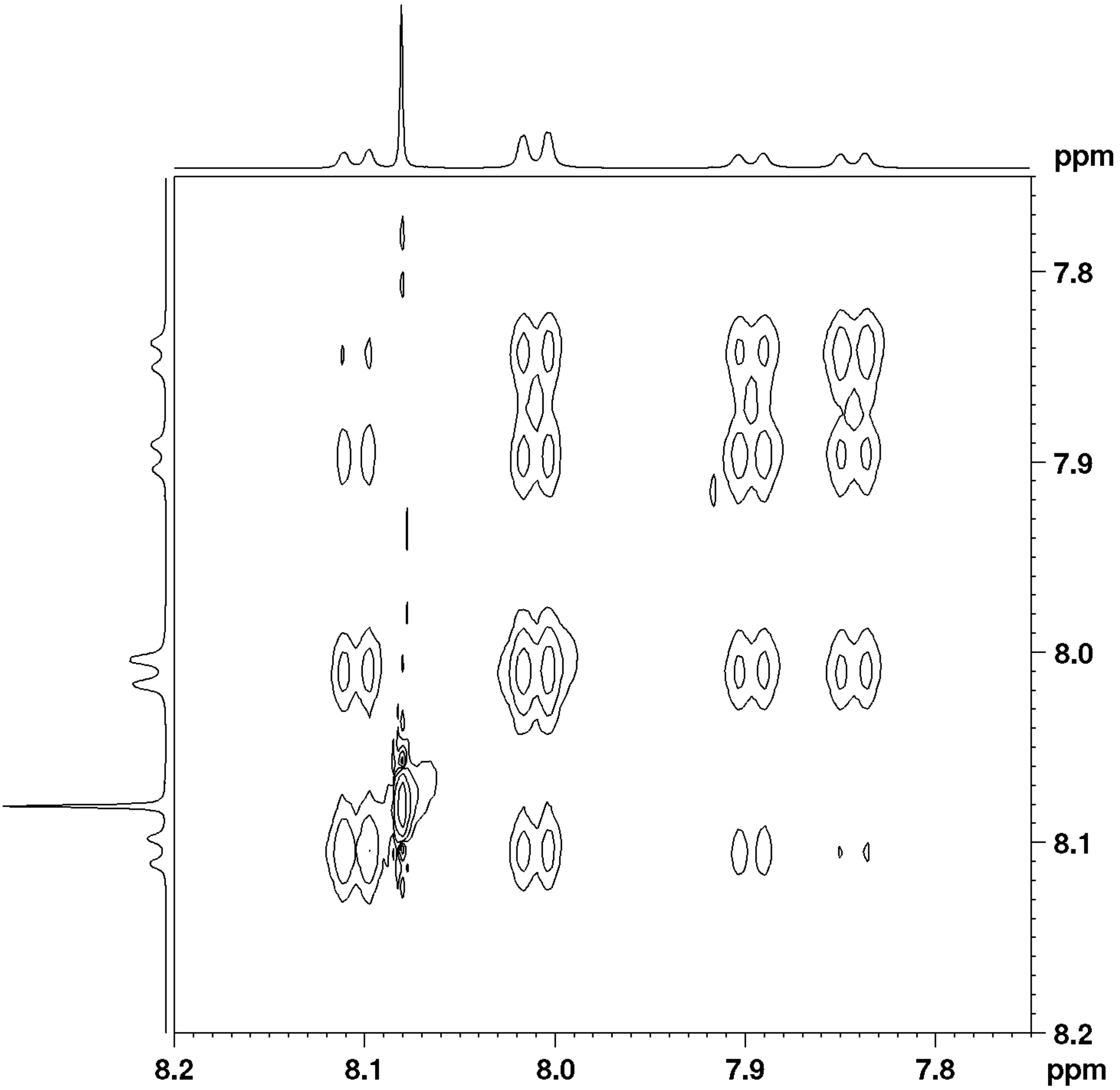

Figure S11. ^{1}H ROESY spectrum of compound **11** in DMF-d7 at 223K using mixing time of 0.3 s (region of Ar-H-3 protons).

In the ^{1}H ROESY spectra of **11** the intensity of following peaks were calculated by volume integration:

	Asym	*Sym*	*Asym*	*Asym*
Asym	I_{11}	I_{12}	I_{13}	I_{14}
Sym	I_{21}	I_{22}	I_{23}	I_{24}
Asym	I_{31}	I_{32}	I_{33}	I_{34}
Asym	I_{41}	I_{42}	I_{43}	I_{44}

It can be transformed into:

	Sym	*Asym*
Sym	I_{22}	$I_{21} + I_{23} + I_{24}$
Asym	$I_{12} + I_{32} + I_{42}$	$I_{11} + I_{13} + I_{14} + I_{31} + I_{33} + I_{34} + I_{41} + I_{43} + I_{44}$

Table S10. Rate constants of compound **11** calculated from 2D integrals

T, K	*Asym* to *Sym*	*Sym* to *Asym*
223	1.488	1.992
228	2.747	3.768
233	5.116	6.875
238	10.077	13.974

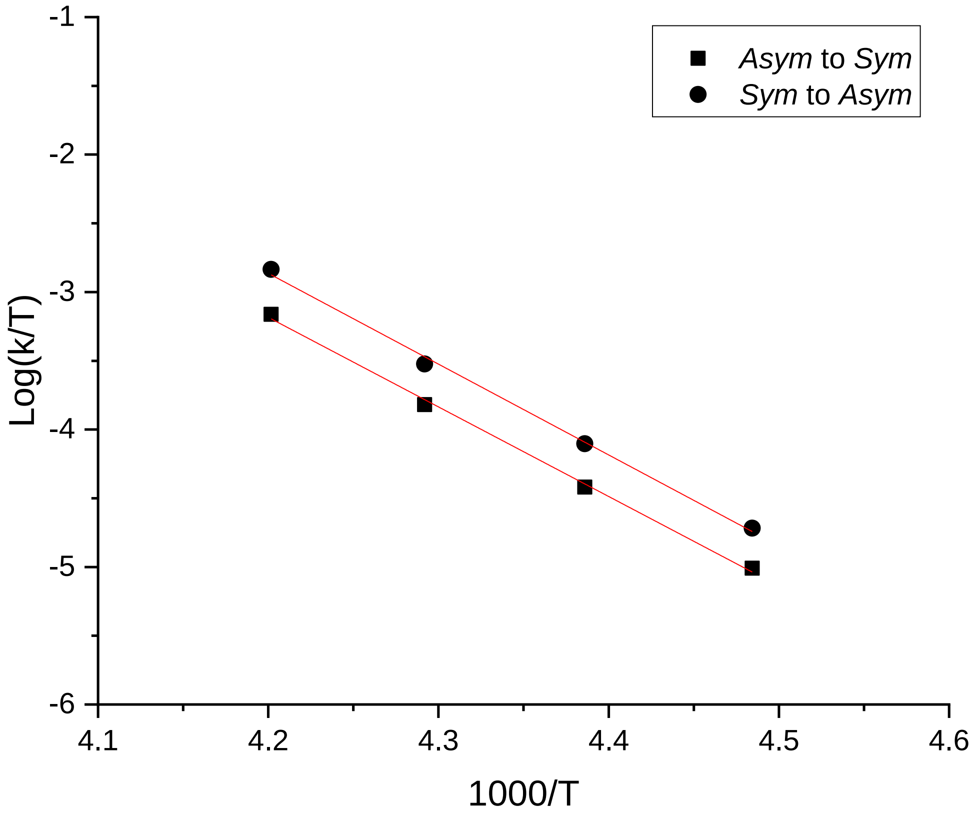

Figure S12. Eyring plot of rate constants of compound **11**

Errors analysis: Usually the presented errors in activation parameters are the statistical errors based on scattering of the data points around the Eyring straight line only. The errors in this analysis are due to inaccuracies in both the calculated rate constants, ***k*,** and the measured temperatures, ***T*** and are computed according to the error propagation equations of Binsch [1] and Heinzer and Oth [2]. The absolute error in temperature is assumed to be not more than ±0.5 K. The relative errors in ***k*** are estimated to be not more than ±10% at all temperatures according the precision of the volume integration of peaks. The errors analysis was performed using self-made computer program using the cited equations.

Table S11. Experimental thermodynamic parameters of exchange processes in compound **11** in DMF-d7

Exchange	$\Delta H^{\neq}$(298K)	$\Delta S^{\neq}$(298K)	$\Delta G^{\neq}$(298K)	R^2
Asym to *Sym*	12.9 ± 1.2	0.8 ± 4.2	12.7 ± 0.1	0.999
Sym to *Asym*	13.1 ± 1.2	2.2 ± 4.4	12.4 ± 0.1	0.999

$\Delta G^{\neq}$ and $\Delta H^{\neq}$ in kcal mol^{-1} and $\Delta S^{\neq}$ in cal mol^{-1} K^{-1}

Table S12. Experimental Thermodynamic parameters of compound **11** in DMF-d7

Conformer	ΔH^0 (298K)	ΔS^0 (298K)	ΔG^0 (298K)
Asym	0.00	0.00	0.00
Sym	0.3 ± 0.1	1.8 ± 0.4	-0.3 ± 0.0

$\Delta H^0 = H^0$ (*Sym*) - H^0 (*Asym*)
$\Delta S^0 = S^0$ (*Sym*) - S^0 (*Asym*)
$\Delta G^0 = G^0$ (*Sym*) - G^0 (*Asym*)
ΔG^0 and ΔH^0 in kcal mol^{-1} and ΔS^0 in cal mol^{-1} K^{-1}

Compound **12**

^{1}H and ^{13}C spectra were recorded on a Bruker II+ 600 spectrometer (BBO probe) at 600.13 for ^{1}H NMR and 150.92 MHz for ^{13}C NMR with TMS as internal standard for chemical shifts (δ, ppm). The spectra were recorded in steps of 10 K between 233 and 293 K (0.05M in 600 µL DMF-d7). Temperature calibration was done with B-VT 3000 unit (it was checked and calibrated with methanol and ethylene glycol reference samples). ^{1}H NMR spectra were acquired using a spectral width of 10 kHz, an acquisition time of 3.4 s and 32 scans, zerofilled to 64k datapoints (0.15 Hz per point) and processed without apodization.

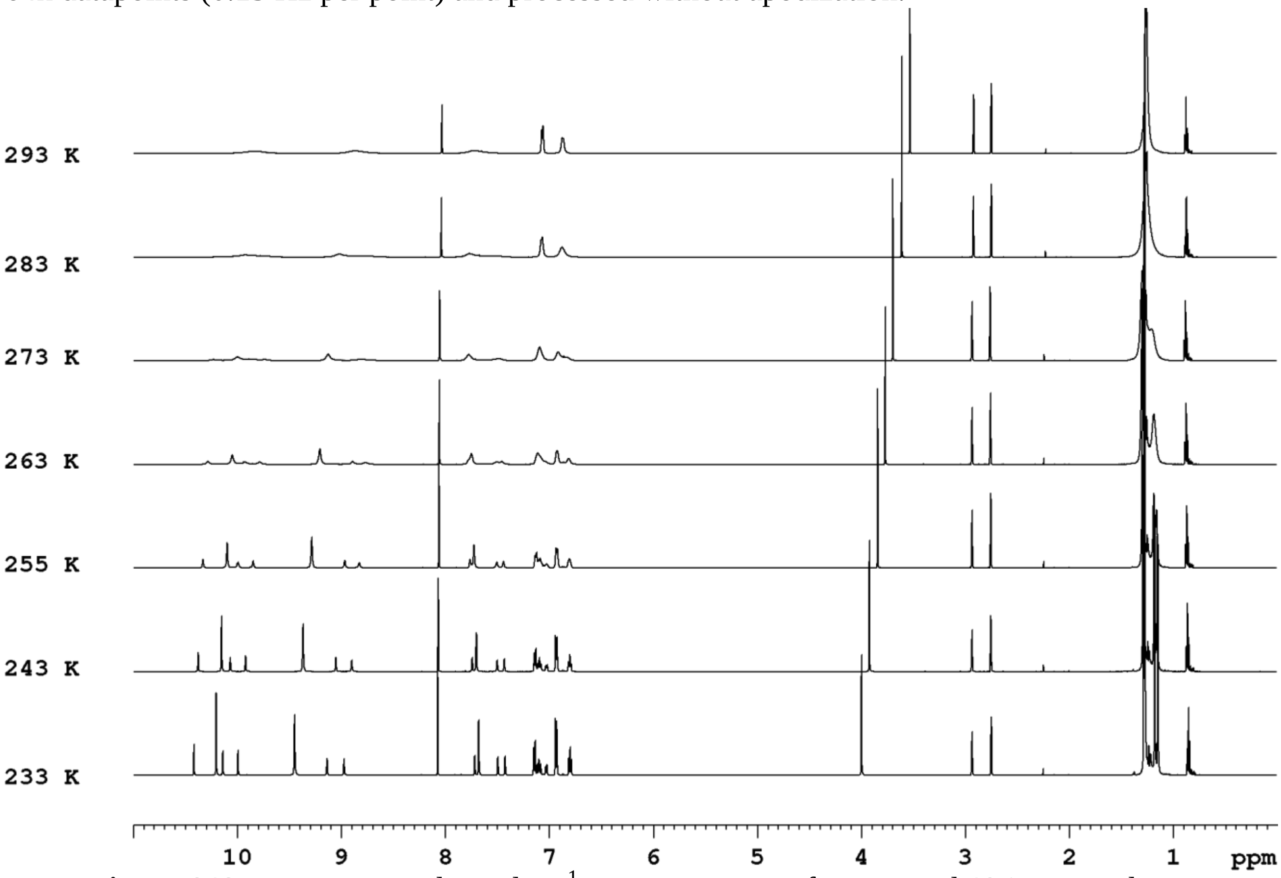

Figure S13. Temperature-dependent ^{1}H NMR spectra of compound **12** in DMF-d7.

^{1}H ROESY spectra (roesyph.2) were recorded on a BBO probe in steps of 5 K between 233 and 258 K. The spectra were acquired using a spectral width of 3.0 kHz, 2048 x 256 complex time domain datapoints, mixing times in the range of 0.01 to 0.3 s and 8 scans in about 75 min. The spectra were zerofilled to 4096 x 4096 datapoints and processed with a shifted square sine bell apodization in both dimensions. Populations and exchange rates were obtained from diagonal- and crosspeak integrals using EXSYCalc (MestreLab Research S.L.).

In the ^{1}H ROESY spectra of **12** the intensity of following peaks were calculated by volume integration in order to obtain the rate constants of *asym* to *sym* exchange:

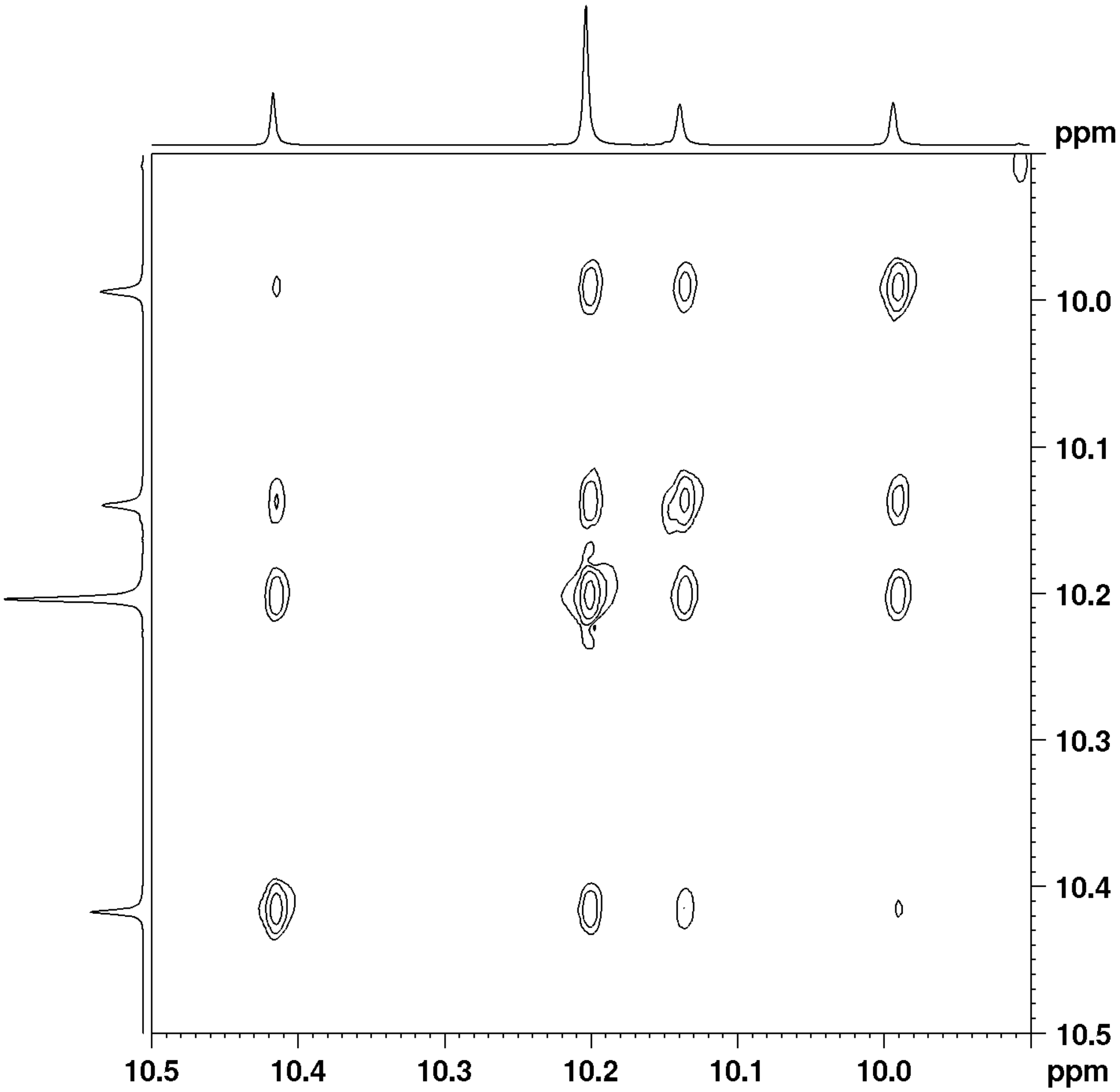

Figure S14. ^{1}H ROESY spectrum of compound **12** in DMF-d7 at 233K using mixing time of 0.3 s (region of Ar-H-6 protons).

In the ^{1}H ROESY spectra of **12** the intensity of following peaks were calculated by volume integration:

	Asym	*Sym*	*Asym*	*Asym*
Asym	I_{11}	I_{12}	I_{13}	I_{14}
Sym	I_{21}	I_{22}	I_{23}	I_{24}
Asym	I_{31}	I_{32}	I_{33}	I_{34}
Asym	I_{41}	I_{42}	I_{43}	I_{44}

It can be transformed into:

	Sym	*Asym*
Sym	I_{22}	$I_{21} + I_{23} + I_{24}$
Asym	$I_{12} + I_{32} + I_{42}$	$I_{11} + I_{13} + I_{14} + I_{31} + I_{33} + I_{34} + I_{41} + I_{43} + I_{44}$

Table S13. Rate constants of compound **12** calculated from 2D integrals

T, K	*Asym* to *Sym*	*Sym* to *Asym*
233	1.131	1.251
238	2.118	2.479
243	4.071	4.736
248	7.558	8.621
258	31.445	34.470

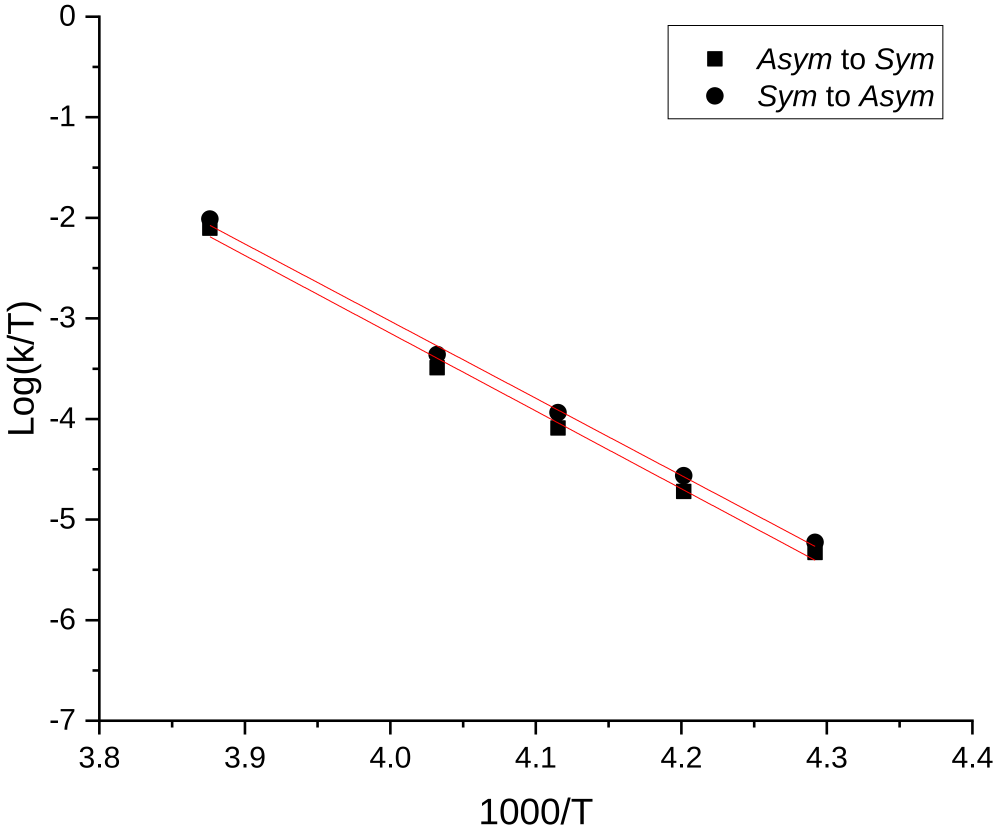

Figure S15. Eyring plot of rate constants of compound **12**

Errors analysis: Usually the presented errors in activation parameters are the statistical errors based on scattering of the data points around the Eyring straight line only. The errors in this analysis are due to inaccuracies in both the calculated rate constants, ***k*,** and the measured temperatures, ***T*** and are computed according to the error propagation equations of Binsch [1] and Heinzer and Oth [2]. The absolute error in temperature is assumed to be not more than ±0.5 K. The relative errors in ***k*** are estimated to be not more than ±10% at all temperatures according the precision of the volume integration of peaks. The errors analysis was performed using self-made computer program using the cited equations.

Table S14. Experimental thermodynamic parameters of exchange processes in compound **12** in DMF-d7

Exchange	$\Delta H^{\neq}$(298K)	$\Delta S^{\neq}$(298K)	$\Delta G^{\neq}$(298K)	R^2
Asym to *Sym*	15.3 ± 0.9	7.6 ± 3.6	13.0 ± 0.1	0.998
Sym to *Asym*	14.9 ± 0.8	6.4 ± 3.2	13.0 ± 0.1	0.999

$\Delta G^{\neq}$ and $\Delta H^{\neq}$ in kcal mol^{-1} and $\Delta S^{\neq}$ in cal mol^{-1} K^{-1}

Table S15. Experimental Thermodynamic parameters of compound **12** in DMF-d7

Conformer	ΔH^0 (298K)	ΔS^0 (298K)	ΔG^0 (298K)
Asym	0.00	0.00	0.00
Sym	0.3 ± 0.03	1.5 ± 0.2	-0.2 ± 0.0

$\Delta H^0 = H^0$ (*Sym*) - H^0 (*Asym*)

$\Delta S^0 = S^0$ (*Sym*) - S^0 (*Asym*)

$\Delta G^0 = G^0$ (*Sym*) - G^0 (*Asym*)

ΔG^0 and ΔH^0 in kcal mol^{-1} and ΔS^0 in cal mol^{-1} K^{-1}

Compound **13**

^{1}H and ^{13}C spectra were recorded on a Bruker II+ 600 spectrometer (BBO probe) at 600.13 for ^{1}H NMR and 150.92 MHz for ^{13}C NMR with TMS as internal standard for chemical shifts (δ, ppm). The spectra were recorded in steps of 10 K between 273 and 323 K (0.05M in 600 μL DMF-d7). Temperature calibration was done with B-VT 3000 unit (it was checked and calibrated with methanol and ethylene glycol reference samples). ^{1}H NMR spectra were acquired using a spectral width of 10 kHz, an acquisition time of 3.4 s and 32 scans, zerofilled to 64k datapoints (0.15 Hz per point) and processed without apodization.

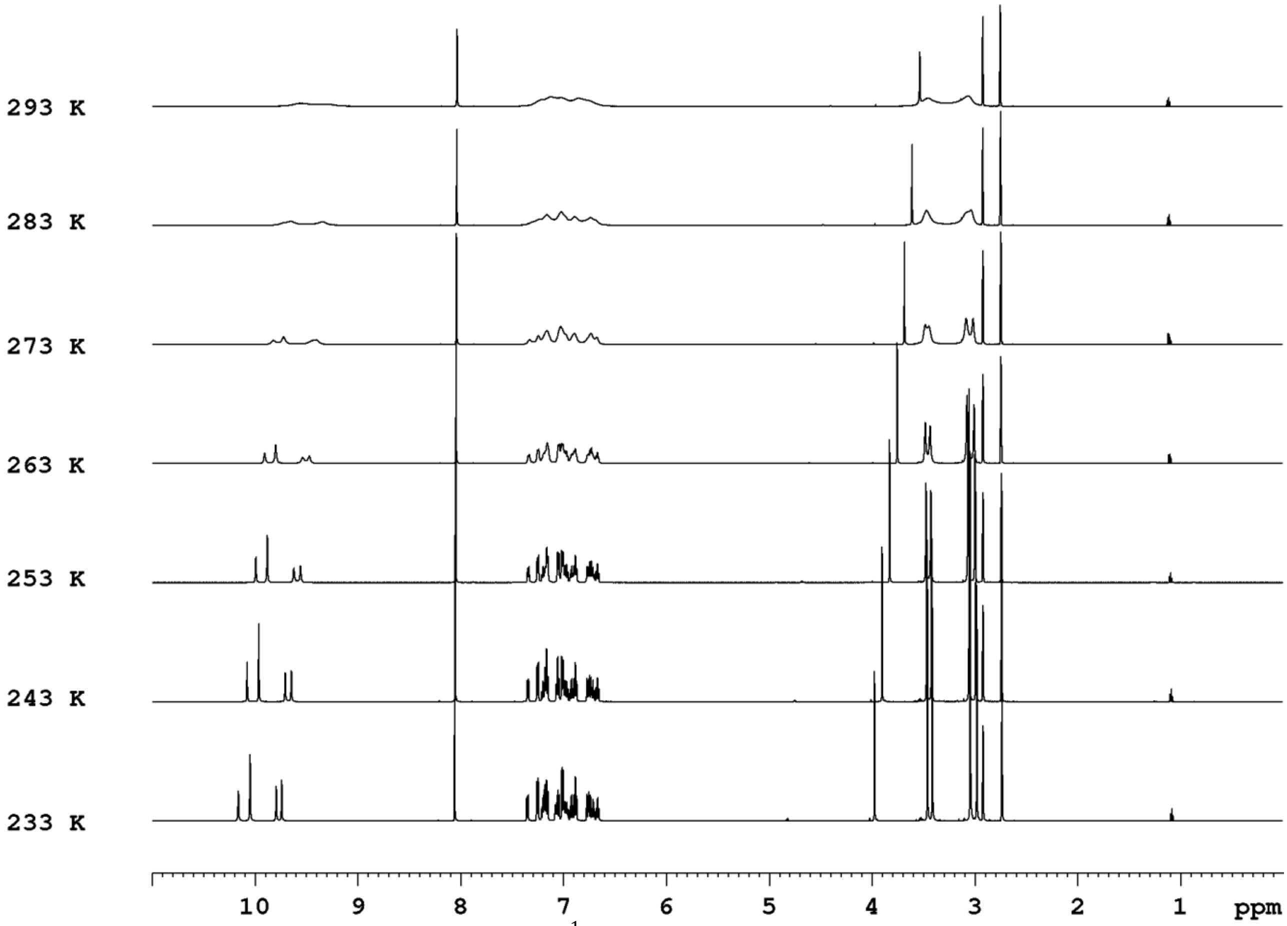

Figure S16. Temperature-dependent ^{1}H NMR spectra of compound **13** in DMF-d7.

^{1}H ROESY spectra (roesyph.2) were recorded on a BBO probe in steps of 5 K between 223 and 253 K. The spectra were acquired using a spectral width of 3.0 kHz, 2048 x 256 complex time domain datapoints, mixing times in the range of 0.03 to 0.3 s and 8 scans in about 75 min. The spectra were zerofilled to 4096 x 4096 datapoints and processed with a shifted square sine bell apodization in both dimensions. Populations and exchange rates were obtained from diagonal- and crosspeak integrals using EXSYCalc (MestreLab Research S.L.).

In the ^{1}H ROESY spectra of **13** the intensity of following peaks were calculated by volume integration in order to obtain the rate constants of *asym* to *sym* exchange:

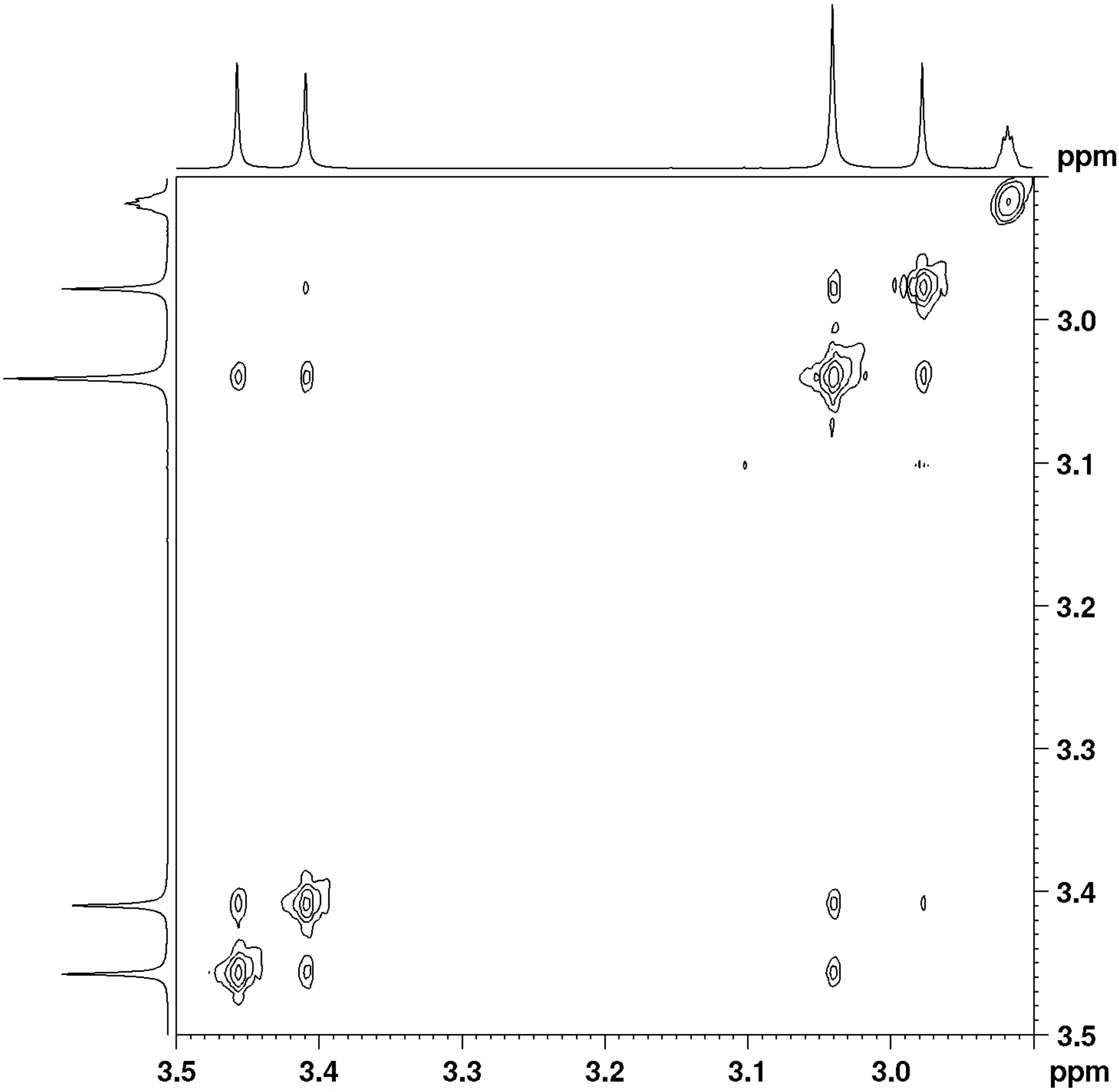

Figure S17. ^{1}H ROESY spectrum of compound **13** in DMF-d7 at 233K using mixing time of 0.3 s (region of N-CH_3 protons).

In the ^{1}H ROESY spectra of **13** the intensity of following peaks were calculated by volume integration:

	Asym	*Asym*	*Sym*	*Asym*
Asym	I_{11}	I_{12}	I_{13}	I_{14}
Asym	I_{21}	I_{22}	I_{23}	I_{24}
Sym	I_{31}	I_{32}	I_{33}	I_{34}
Asym	I_{41}	I_{42}	I_{43}	I_{44}

It can be transformed into:

	Sym	*Asym*
Sym	I_{33}	$I_{31} + I_{32} + I_{34}$
Asym	$I_{13} + I_{23} + I_{43}$	$I_{11} + I_{12} + I_{14} + I_{21} + I_{22} + I_{24} + I_{41} + I_{42} + I_{44}$

Table S16. Rate constants of compound **13** calculated from 2D integrals

T, K	*Asym* to *Sym*	*Sym* to *Asym*
233	0.264	0.412
238	0.453	0.801
243	0.941	1.645
248	2.026	3.202
253	3.726	3.659

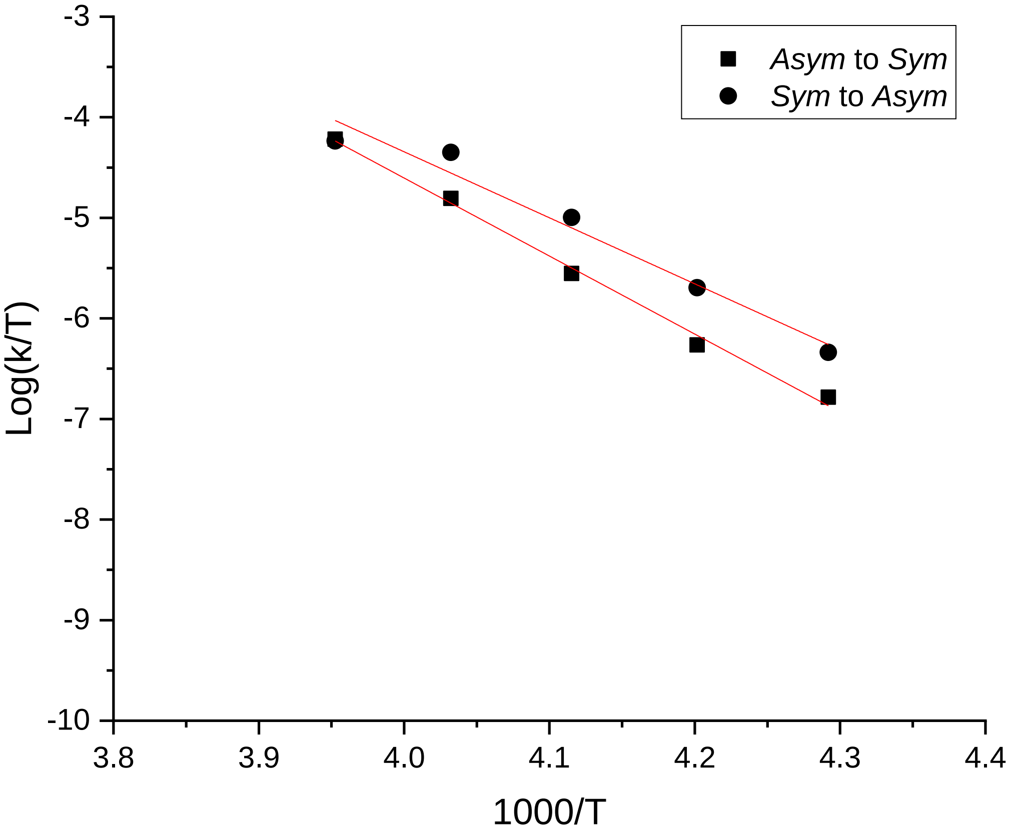

Figure S18. Eyring plot of rate constants of compound **13**

Errors analysis: Usually the presented errors in activation parameters are the statistical errors based on scattering of the data points around the Eyring straight line only. The errors in this analysis are due to inaccuracies in both the calculated rate constants, ***k*,** and the measured temperatures, ***T*** and are computed according to the error propagation equations of Binsch [1] and Heinzer and Oth [2]. The absolute error in temperature is assumed to be not more than ±0.5 K. The relative errors in ***k*** are estimated to be not more than ±10% at all temperatures according the precision of the volume integration of peaks. The errors analysis was performed using self-made computer program using the cited equations.

Table S17. Experimental thermodynamic parameters of exchange processes in compound **13** in DMF-d7

Exchange	$\Delta H^{\neq}$(298K)	$\Delta S^{\neq}$(298K)	$\Delta G^{\neq}$(298K)	R^2
Asym to *Sym*	15.4 ± 1.1	5.3 ± 6.1	13.8 ± 0.1	0.998
Sym to *Asym*	13.0 ± 1.5	-3.7 ± 4.8	14.1 ± 0.1	0.984

$\Delta G^{\neq}$ and $\Delta H^{\neq}$ in kcal mol^{-1} and $\Delta S^{\neq}$ in cal mol^{-1} K^{-1}

Table S18. Experimental Thermodynamic parameters of compound **13** in DMF-d7

Conformer	ΔH^0 (298K)	ΔS^0 (298K)	ΔG^0 (298K)
Asym	0.00	0.00	0.00
Sym	0.4 ± 0.02	2.8 ± 0.1	-0.4 ± 0.0

$\Delta H^0 = H^0$ (*Sym*) - H^0 (*Asym*)

$\Delta S^0 = S^0$ (*Sym*) - S^0 (*Asym*)

$\Delta G^0 = G^0$ (*Sym*) - G^0 (*Asym*)

ΔG^0 and ΔH^0 in kcal mol^{-1} and ΔS^0 in cal mol^{-1} K^{-1}

Compound **14**

^{1}H and ^{13}C spectra were recorded on a Bruker II+ 600 spectrometer (BBO probe) at 600.13 for ^{1}H NMR and 150.92 MHz for ^{13}C NMR with TMS as internal standard for chemical shifts (δ, ppm). The spectra were recorded in steps of 10 K between 273 and 323 K (0.05M in 600 μL DMF-d7). Temperature calibration was done with B-VT 3000 unit (it was checked and calibrated with methanol and ethylene glycol reference samples). ^{1}H NMR spectra were acquired using a spectral width of 10 kHz, an acquisition time of 3.4 s and 32 scans, zerofilled to 64k datapoints (0.15 Hz per point) and processed without apodization.

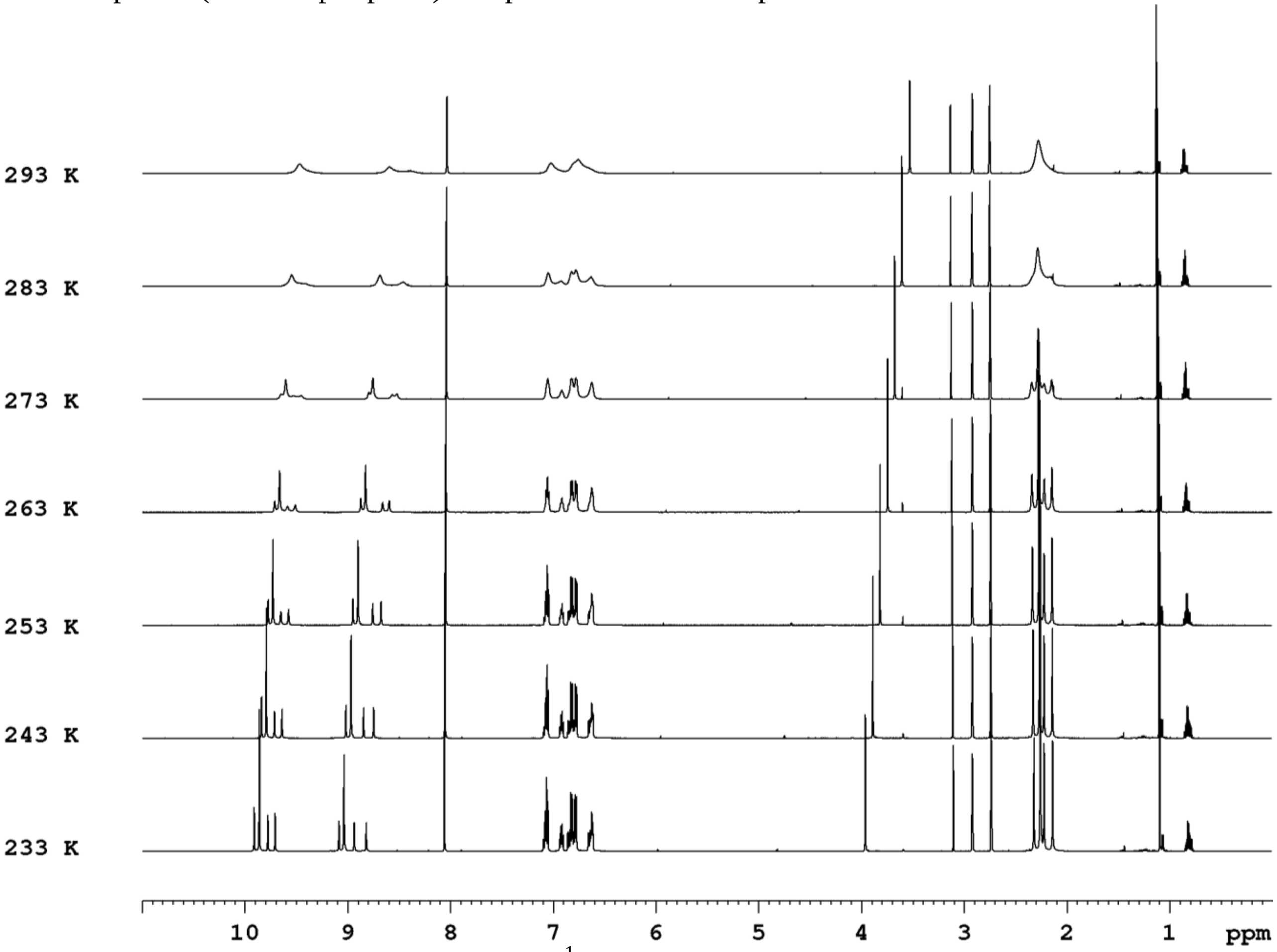

Figure S19. Temperature-dependent ^{1}H NMR spectra of compound **14** in DMF-d7.

^{1}H ROESY spectra (roesyph.2) were recorded on a BBO probe in steps of 5 K between 233 and 253 K. The spectra were acquired using a spectral width of 3.0 kHz, 2048 x 256 complex time domain datapoints, mixing times in the range of 0.03 to 0.3 s and 8 scans in about 75 min. The spectra were zerofilled to 4096 x 4096 datapoints and processed with a shifted square sine bell apodization in both dimensions. Populations and exchange rates were obtained from diagonal- and crosspeak integrals using EXSYCalc (MestreLab Research S.L.).

In the ^{1}H ROESY spectra of **14** the intensity of following peaks were calculated by volume integration in order to obtain the rate constants of *asym* to *sym* exchange:

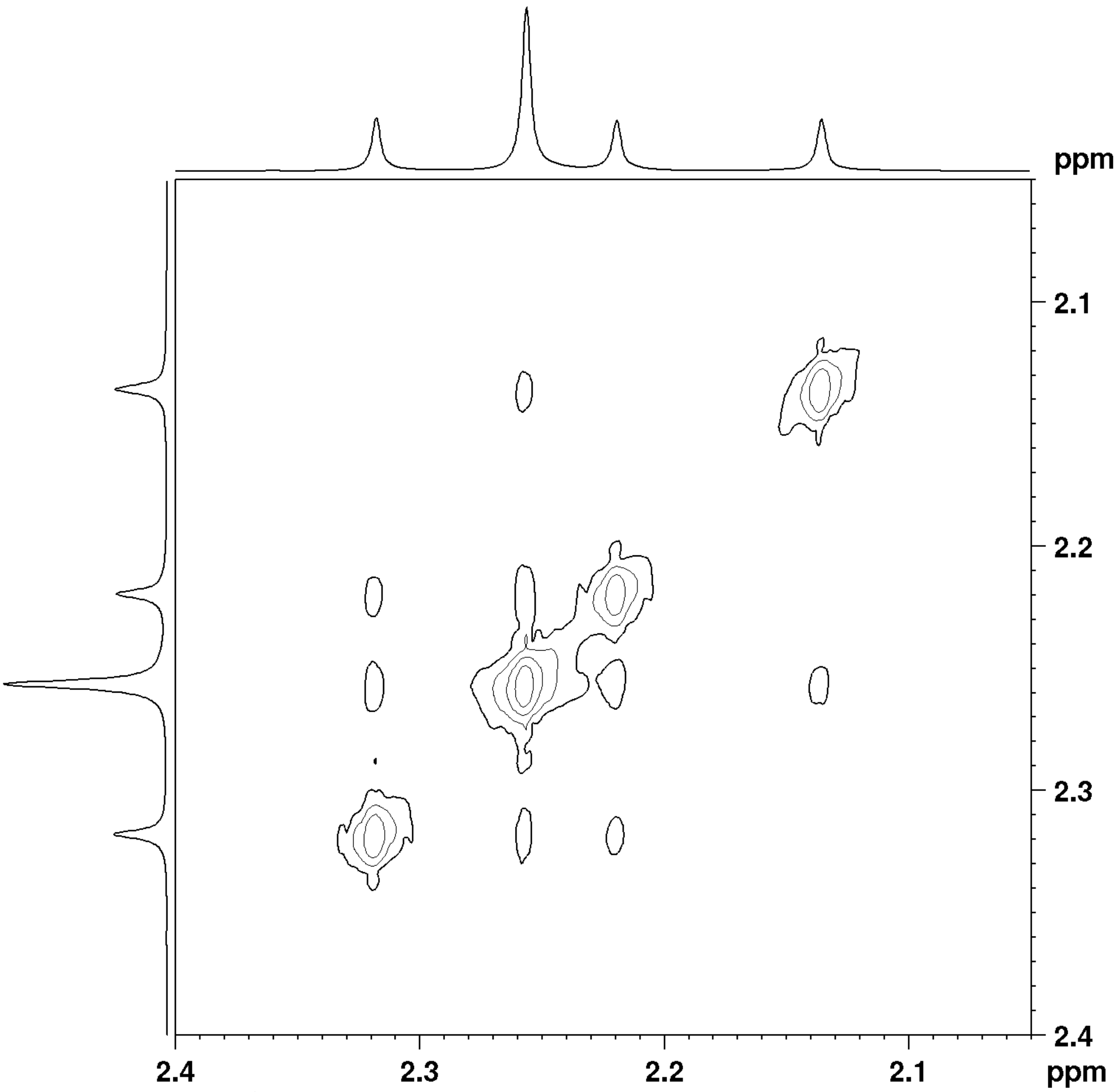

Figure S20. ^{1}H ROESY spectrum of compound **14** in DMF-d7 at 233K using mixing time of 0.3 s (region of Ar-CH_3 protons).

In the ^{1}H ROESY spectra of **14** the intensity of following peaks were calculated by volume integration:

	Asym	*Sym*	*Asym*	*Asym*
Asym	I_{11}	I_{12}	I_{13}	I_{14}
Sym	I_{21}	I_{22}	I_{23}	I_{24}
Asym	I_{31}	I_{32}	I_{33}	I_{34}
Asym	I_{41}	I_{42}	I_{43}	I_{44}

It can be transformed into:

	Sym	*Asym*
Sym	I_{22}	$I_{21} + I_{23} + I_{24}$
Asym	$I_{12} + I_{32} + I_{42}$	$I_{11} + I_{13} + I_{14} + I_{31} + I_{33} + I_{34} + I_{41} + I_{43} + I_{44}$

Table S19. Rate constants of compound **14** calculated from 2D integrals

T, K	*Asym* to *Sym*	*Sym* to *Asym*
233	0.211	0.213
238	0.364	0.373
243	0.745	0.735
248	1.273	1.595
253	2.885	3.134

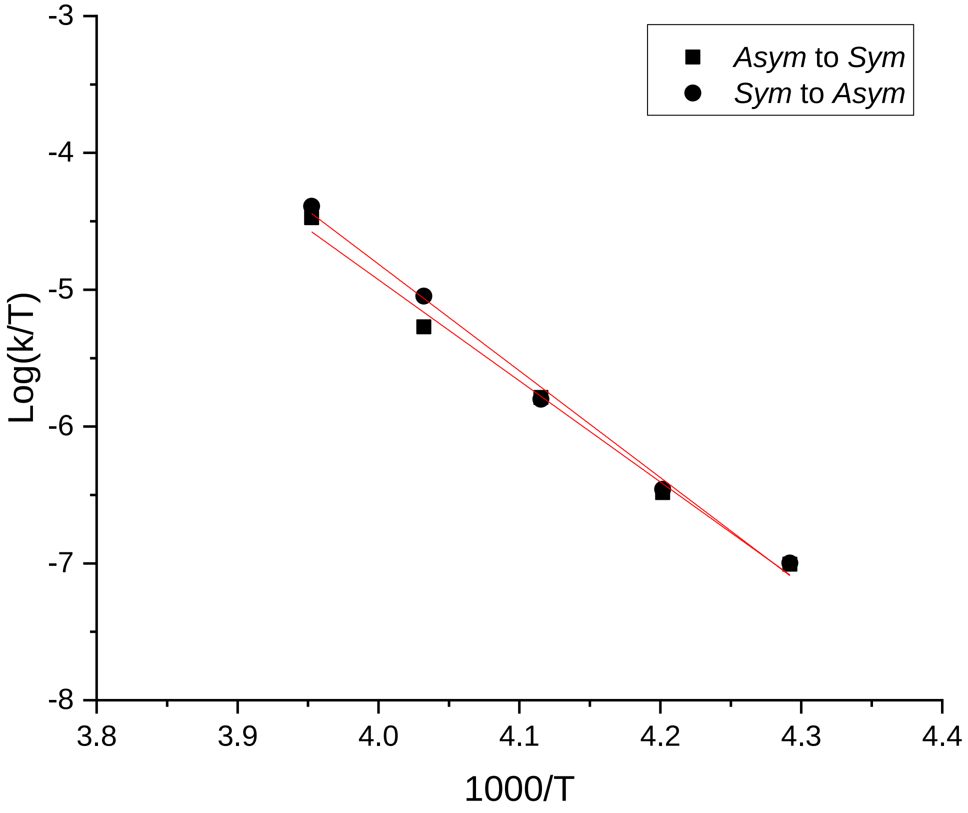

Figure S21. Eyring plot of rate constants of compound **14**

Errors analysis: Usually the presented errors in activation parameters are the statistical errors based on scattering of the data points around the Eyring straight line only. The errors in this analysis are due to inaccuracies in both the calculated rate constants, ***k,*** and the measured temperatures, ***T*** and are computed according to the error propagation equations of Binsch [1] and Heinzer and Oth [2]. The absolute error in temperature is assumed to be not more than ±0.5 K. The relative errors in ***k*** are estimated to be not more than ±10% at all temperatures according the precision of the volume integration of peaks. The errors analysis was performed using self-made computer program using the cited equations.

Table S20. Experimental thermodynamic parameters of exchange processes in compound **14** in DMF-d7

Exchange	$\Delta H^{\neq}$(298K)	$\Delta S^{\neq}$(298K)	$\Delta G^{\neq}$(298K)	R^2
Asym to *Sym*	14.7 ± 1.2	1.7 ± 3.7	14.2 ± 0.1	0.996
Sym to *Asym*	15.5 ± 1.1	5.2 ± 5.3	13.9 ± 0.1	0.997

$\Delta G^{\neq}$ and $\Delta H^{\neq}$ in kcal mol^{-1} and $\Delta S^{\neq}$ in cal mol^{-1} K^{-1}

Table S21. Experimental Thermodynamic parameters of compound **14** in DMF-d7

Conformer	ΔH^0 (298K)	ΔS^0 (298K)	ΔG^0 (298K)
Asym	0.00	0.00	0.00
Sym	0.2 ± 0.02	0.6 ± 0.1	0.0 ± 0.0

$\Delta H^0 = H^0$ (*Sym*) - H^0 (*Asym*)
$\Delta S^0 = S^0$ (*Sym*) - S^0 (*Asym*)
$\Delta G^0 = G^0$ (*Sym*) - G^0 (*Asym*)
ΔG^0 and ΔH^0 in kcal mol^{-1} and ΔS^0 in cal mol^{-1} K^{-1}

*^{15}N spectra of compound **11** in DMF-d7 at 243K*

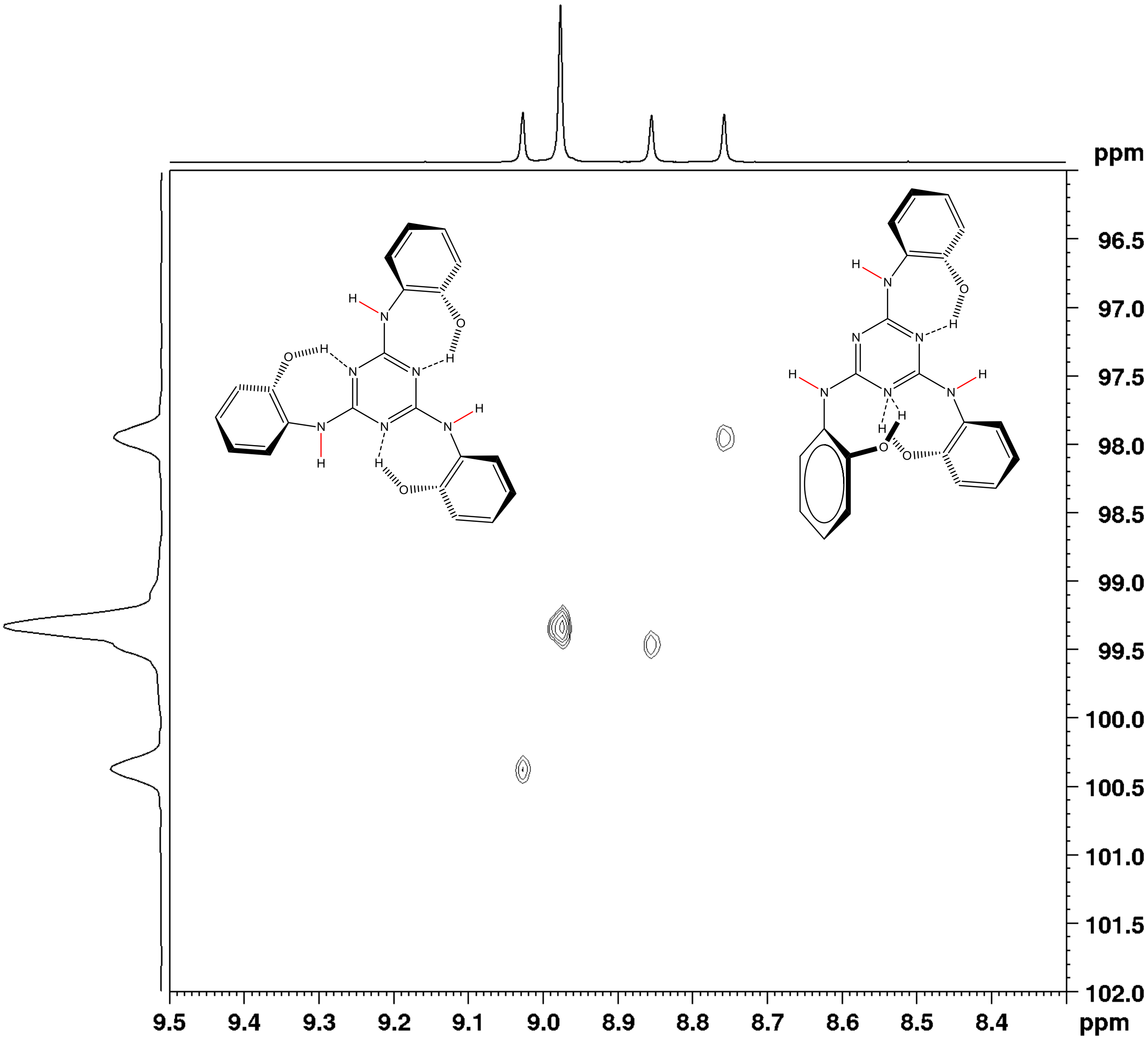

Figure S22. $^{1}H,^{15}N$-HSQC spectrum of compound **11** in DMF-d7 at 243K. The symmetric conformer has only one ^{15}N signal and only one proton signal for NH group. In asymmetric conformer there are 3 signals for NH group either in ^{15}N spectrum or in proton spectrum due to different environment.

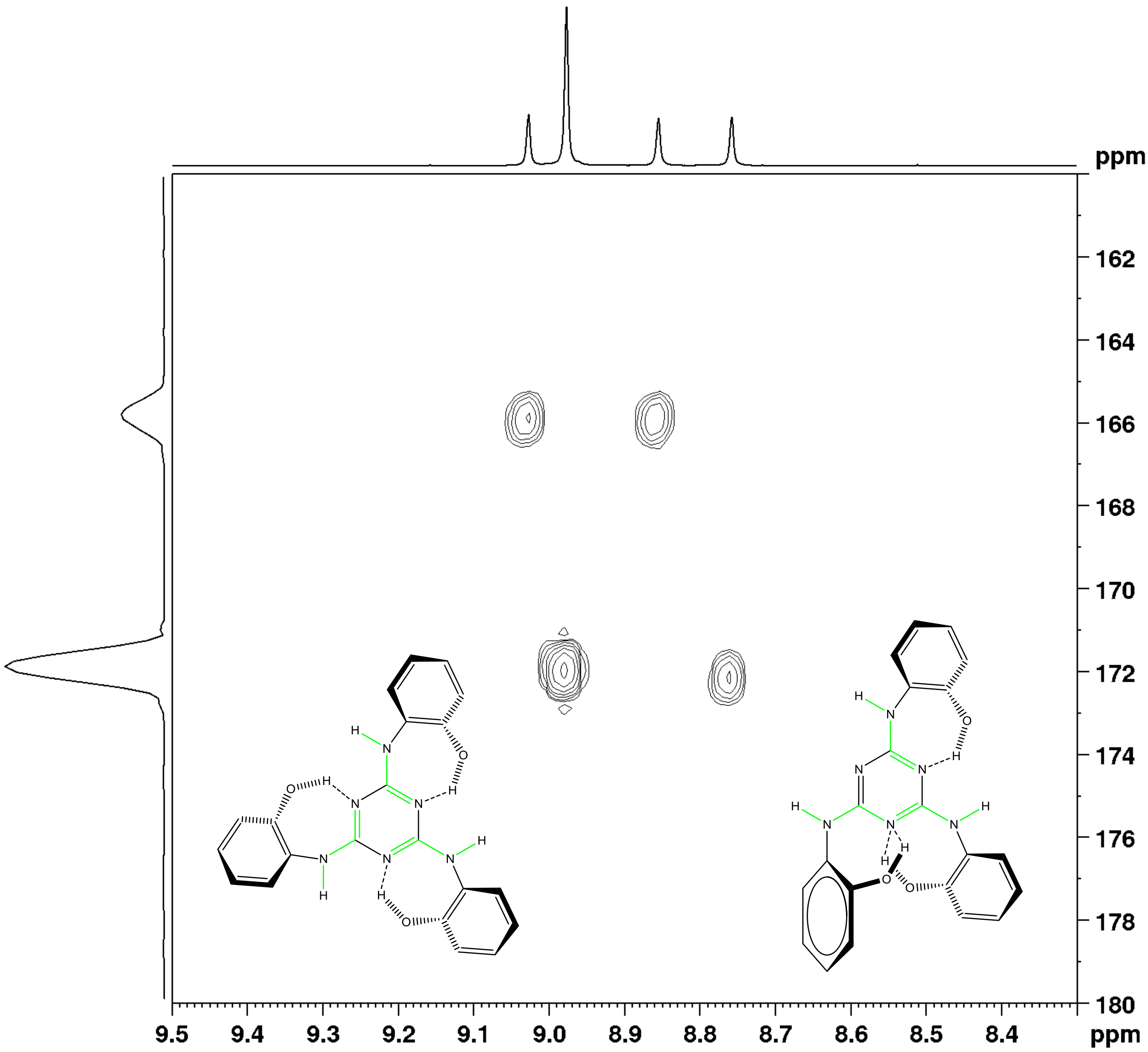

Figure S23. $^{1}H,^{15}N$-HMBC spectrum of compound **11** in DMF-d7 at 243K. The symmetric conformer has only one ^{15}N signal for the 3 nitrogen atoms of 1,3,5-triazine. In asymmetric conformer the two NH protons correlate with one nitrogen atom of 1,3,5-triazine (166 ppm). The third NH proton correlate with a nitrogen atom of 1,3,5-triazine with similar environment and has ^{15}N chemical shift close to that of the symmetric conformer (172 ppm).

DFT calculations

All calculations were performed by means of quantum chemical calculations at the density functional theory (DFT) level using Gaussian09 program package [1].

<u>Geometry optimization</u>

The geometries of all compounds have been fully optimized using B3LYP [2] functional with 6-31+G(d,p) basis set [3] or using M06-2X [4,5] functional with TZVP [6] basis set. All ground state structures were optimized without restrictions and using an ultrafine grid in the computation of two-electron integrals and their derivatives. Solvent effect was included implicitly to the optimizations via the SMD [7] model with the built in parameters for solvents (DMF or CH_3CN). The nature of all critical points was confirmed by means of the vibrational analysis. The thermal corrections to Gibbs free energy to 298.15 K have been calculated for all minima from unscaled vibrational frequencies obtained at the same level.

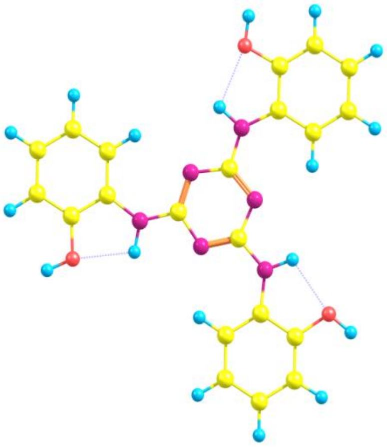

GS0

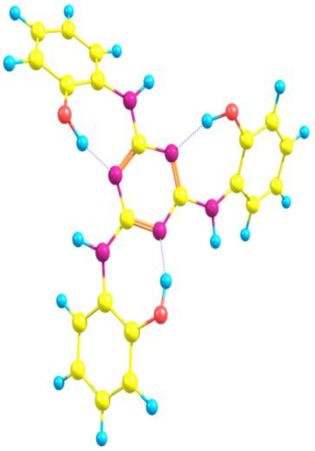

GS1

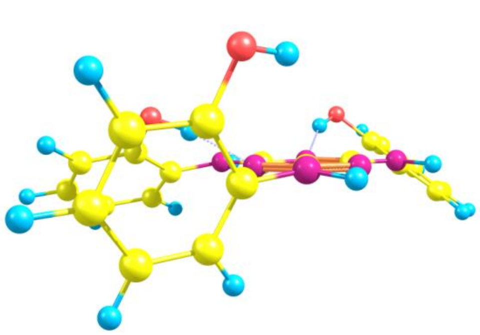

GS2 (Rotation around $C_{triazine}$-N bond)

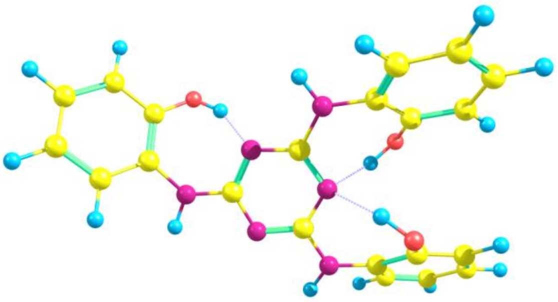

GS2_3H (Rotation around $C_{triazine}$-N bond with 3 hydrogen bonds)

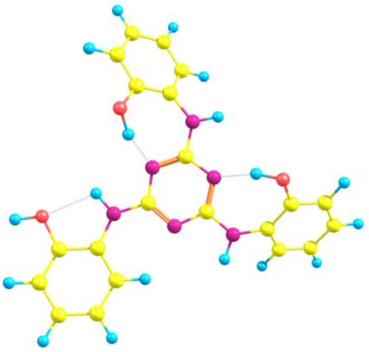

GS3 (Rotation around C_{Ph}-N bond)

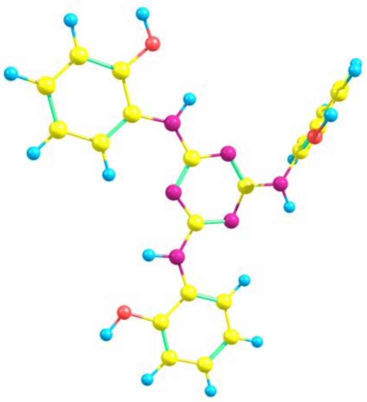

GS4

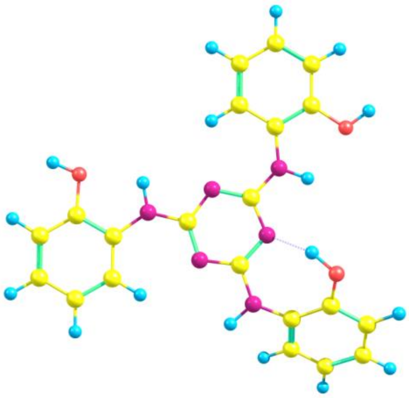

GS5

Figure S24. DFT calculated ground state (GS) conformations of compound **11.**

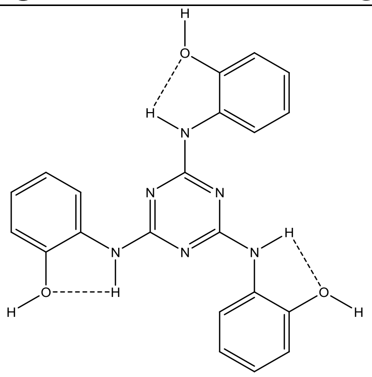

GS0

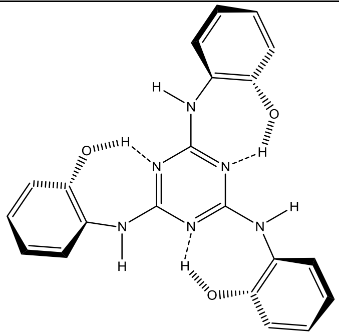

GS1

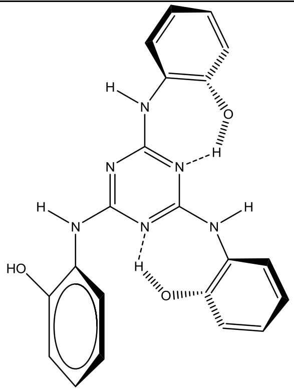

GS2

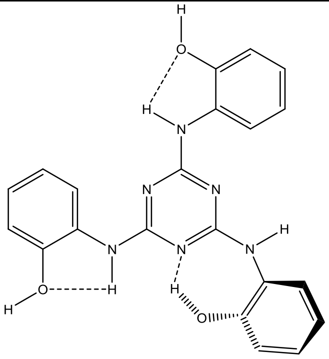

GS3

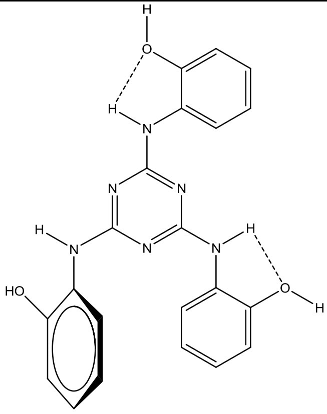

GS4

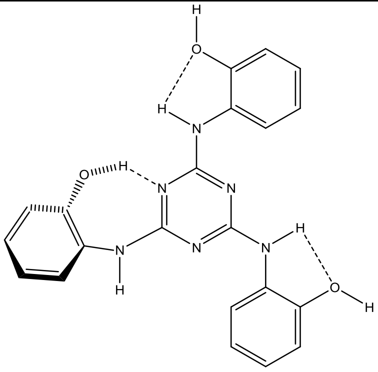

GS5

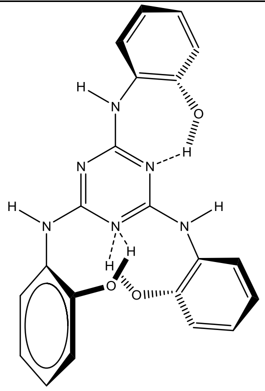

GS2_3H

Off: no hydrogen bond
O-bond: N-H…O hydrogen bond
N-bond: O-H…N hydrogen bond

Conformer	First subst.	Second subst.	Third subst.
GS0	O-bond	O-bond	O-bond
GS1	N-bond	N-bond	N-bond
GS2	Off	N-bond	N-bond
GS2_3H	N-bond	N-bond	N-bond
GS3	N-bond	N-bond	O-bond
GS4	Off	O-bond	O-bond
GS5	N-bond	O-bond	O-bond

Table S22. Theoretical thermodynamic parameters of studied GS structures

Comp.	Structure	Method	$\Delta H^{\neq}$ (298 K) kcal mol^{-1}	$\Delta S^{\neq}$ (298 K) cal K^{-1} mol^{-1}	$\Delta G^{\neq}$ (298 K) kcal mol^{-1}
**11**	***Sym GS GS1***	SMD(DMF)//M062X/TZVP	0.0	0.0	0.0
	GS2	SMD(DMF)//M062X/TZVP	0.2	1.9	-0.4
	GS2_3H	SMD(DMF)//M062X/TZVP	-1.2	-2.3	-0.5
	GS3	SMD(DMF)//M062X/TZVP	1.1	-3.7	2.2
	GS5	SMD(DMF)//M062X/TZVP	3.4	6.1	1.6
	GS0	SMD(DMF)//M062X/TZVP	3.8	-5.6	5.5
	GS4	SMD(DMF)//M062X/TZVP	6.4	9.7	3.6
**11**	***Sym GS GS1***	SMD(DMF)//B3LYP/6-31+G(d,p)	0.0	0.0	0.0
	GS2	SMD(DMF)//B3LYP/6-31+G(d,p)	2.1	1.9	1.5
	GS2_3H	SMD(DMF)//B3LYP/6-31+G(d,p)	1.2	-1.4	1.6
	GS3	SMD(DMF)//B3LYP/6-31+G(d,p)	1.6	2.5	0.9
	GS5	SMD(DMF)//B3LYP/6-31+G(d,p)	3.5	2.8	2.6
	GS0	SMD(DMF)//B3LYP/6-31+G(d,p)	4.9	2.4	4.2
	GS4	SMD(DMF)//B3LYP/6-31+G(d,p)	6.8	6.2	4.9

Cartesian coordinates of the SMD(DMF)//M062X/TZVP optimized GS geometries for **11-14** and SMD(DMF)//B3LYP/6-31+G(d,p) optimized geometries for **4-6**:

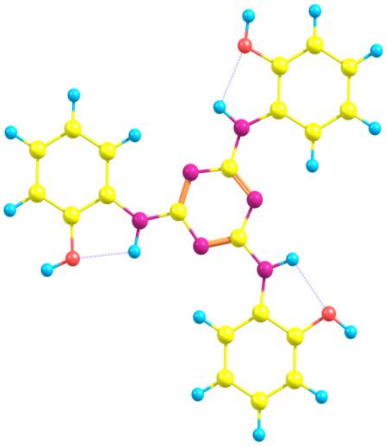

11-GS0

C	1.01053800	-0.79742700	-0.03077300
N	2.13687900	-1.55728400	-0.01401300
N	-0.18797300	-1.35910800	-0.04027800
C	2.31085500	-2.94977100	-0.02920000
N	1.25597800	0.53165900	-0.02192100
C	-1.21199000	-0.47922900	-0.04627900
C	3.64546700	-3.38624500	0.03792400
C	1.30183700	-3.90522300	-0.10674700
C	0.17433900	1.28801200	-0.02575500
N	-2.43124500	-1.07037000	-0.03772400
N	-1.09750900	0.84141900	-0.04179500
C	3.95282400	-4.73504300	0.02619200
C	1.61499600	-5.26239400	-0.11757300
H	0.27533200	-3.59079100	-0.15988100
N	0.28285400	2.63777400	-0.00656900
C	-3.72118000	-0.52067500	-0.01821600
C	2.93338600	-5.68125800	-0.04890500
H	0.81063600	-5.98445500	-0.17693800
C	1.40780400	3.46914900	0.02008800
C	-4.03581800	0.83351400	-0.04706300
C	-4.77198700	-1.44877600	0.05656000
C	2.73141400	3.05679700	0.13468600
C	1.13531000	4.84343500	-0.06339200
C	-5.36686900	1.24896700	-0.00287200
H	-3.24114000	1.55772900	-0.09716700
C	-6.08623900	-1.03457100	0.10400200
C	3.76187100	3.99311300	0.15405200
H	2.95455600	2.00479500	0.20764800
C	2.16306500	5.77021200	-0.04494300
C	-6.39353700	0.31963200	0.07427800
H	-5.58566700	2.30894800	-0.02404200
C	3.48586300	5.34733700	0.06590300
H	4.78429300	3.64650900	0.24068400
H	-2.39425700	-2.08286800	-0.02725500
H	-0.61177900	3.11230300	-0.04371000
H	2.99423100	-1.01774300	0.00329500
O	-4.41820700	-2.76325400	0.09437700
H	-5.20163200	-3.32236100	0.17085500
O	4.60371200	-2.40401000	0.11011100
H	5.49186800	-2.79727000	0.14811900
O	-0.18785100	5.17545200	-0.16564500
H	-0.30103400	6.13462700	-0.23572400
H	3.18012100	-6.73519700	-0.05353300
H	4.99313000	-5.03668600	0.08052200
H	4.28254300	6.07914300	0.07914500
H	1.91569100	6.82493700	-0.11588000
H	-7.42933900	0.63377100	0.11113800
H	-6.86337200	-1.78684800	0.16627300

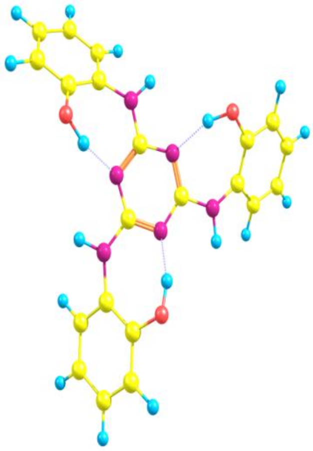

11-GS1

C	-0.20581300	1.28968500	0.07791100
N	-0.37462900	2.62469300	0.09252000
N	-1.27509900	0.49746100	0.07373900
C	-1.57052800	3.35828100	-0.11936900
N	1.07002100	0.85924800	0.07479200
C	-1.01050600	-0.82272000	0.07756500
C	-1.50448100	4.44999700	-0.98449100
C	-2.76683000	3.09478200	0.55938300
C	1.22084600	-0.46324300	0.07826900
N	-2.08337200	-1.63492300	0.09229800
N	0.21006400	-1.35310200	0.07433900
C	-2.60046900	5.27286800	-1.18127000
C	-3.86954800	3.91524000	0.34050200
N	2.46080900	-0.98591900	0.09367100
C	-2.12370300	-3.03701900	-0.12091700
C	-3.78975100	5.00332600	-0.51267800
H	-4.78357300	3.68661600	0.87509200
C	3.69495700	-0.31969300	-0.12012300
C	-1.29990400	-3.94304800	0.55846500
C	-3.10401100	-3.52311900	-0.98535000
C	4.06827000	0.84585900	0.56009700
C	4.60468700	-0.92420900	-0.98713700
C	-1.46296000	-5.30791300	0.34045200
C	-3.27307600	-4.88338600	-1.18060100
C	5.33102500	1.38815600	0.34026900
C	5.86633700	-0.38923100	-1.18455200
C	-2.44710600	-5.78037500	-0.51185600
H	-0.80977300	-5.98699000	0.87494000
C	6.23085100	0.77385700	-0.51489400

H	5.59333200	2.29236400	0.87607600
H	-2.97005600	-1.15477000	-0.01292600
H	2.48778200	-1.99343400	-0.01461800
H	0.48338300	3.15320900	-0.01729200
H	-4.65627100	5.63579700	-0.65934400
H	-2.52537600	6.11767100	-1.85364100
H	7.21262400	1.20610500	-0.66220100
H	6.55872200	-0.87768300	-1.85791100
H	-2.56473800	-6.84678600	-0.65785100
H	-4.04392300	-5.23903100	-1.85195200
O	3.26162200	1.45248800	1.47800600
H	2.33915800	1.43742900	1.12406800
O	-2.88906000	2.09040600	1.47446000
H	-2.41211500	1.30120300	1.11899600
O	-0.36836000	-3.54754300	1.47352900
H	0.07822900	-2.74106900	1.11761000
H	-3.73954500	-2.80948900	-1.49735100
H	-0.56950800	4.64590500	-1.49705800
H	4.30373300	-1.83063200	-1.50014700

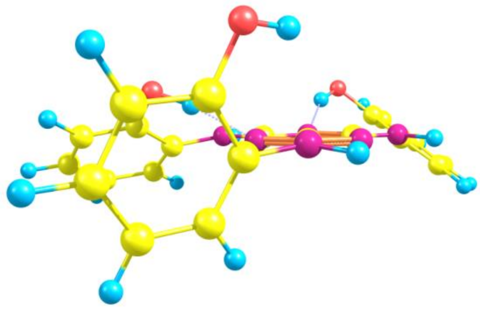

11-GS2

C	-0.35211300	0.50399800	-0.09402800
N	-0.06181300	1.81725600	-0.19075600
N	0.64608500	-0.37461700	0.00609700
C	1.18410000	2.44875900	0.05904700
N	-1.65423900	0.18245800	-0.11581600
C	0.26288600	-1.65999100	0.09465000
C	1.18217700	3.57452900	0.88122100
C	2.38060600	2.04718300	-0.54721500
C	-1.91492400	-1.12504300	-0.01482800
N	1.23603000	-2.59469800	0.17224400
N	-0.99568400	-2.09023400	0.10047000
C	2.34348200	4.29434500	1.10834300
C	3.54788800	2.76161600	-0.29581900
N	-3.20323600	-1.53042900	-0.03091300
C	2.61501500	-2.27043100	0.32182100
C	3.53285800	3.88421200	0.51586400
H	4.46045700	2.42399900	-0.77232300
C	-4.37210600	-0.74087800	0.11436600
C	3.35452900	-1.93331600	-0.81263800
C	3.22578600	-2.27730700	1.56940400
C	-4.62915200	0.40180200	-0.65405700
C	-5.34702400	-1.19505700	1.00239600
C	4.69255300	-1.57785500	-0.68807000
C	4.56548900	-1.94048500	1.69470900
C	-5.84054800	1.06959600	-0.50105200
C	-6.55844300	-0.53677700	1.13357700

C	5.29147700	-1.58381200	0.56210700
H	5.24630700	-1.30849600	-1.57905200
C	-6.80620300	0.60209300	0.37518700
H	-6.01122400	1.95255600	-1.10502300
H	0.93201300	-3.54346000	0.35374200
H	-3.32291700	-2.51838100	0.15999300
H	-0.87188100	2.42504200	-0.14793000
H	4.44935500	4.43403300	0.68946500
H	2.31861000	5.16851800	1.74605300
H	-7.74702400	1.12965600	0.46960900
H	-7.30217100	-0.91144900	1.82493800
H	6.33543300	-1.30969800	0.65165800
H	5.03718800	-1.94747700	2.66864300
O	-3.75536800	0.86717700	-1.59303100
H	-2.84388200	0.79014500	-1.21456600
O	2.44330700	1.00448000	-1.42343400
H	1.87599900	0.27969300	-1.06489700
O	2.79598100	-1.91624800	-2.05289000
H	1.91071600	-2.31090700	-2.02416600
H	2.63322800	-2.54231000	2.43696400
H	0.24688000	3.87877400	1.33691800
H	-5.13847600	-2.08475000	1.58560700

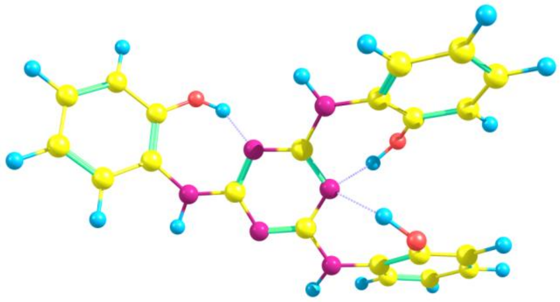

11-GS2_3H

C	0.39845300	0.52964700	0.12836300
N	0.10205400	1.82525900	0.33976300
N	-0.61236100	-0.31817600	-0.08358700
C	-1.16032100	2.43588000	0.10738200
N	1.68782500	0.17544300	0.15611100
C	-0.26272400	-1.60290600	-0.27120400
C	-1.19462500	3.57199100	-0.69848000
C	-2.34531600	1.97986600	0.69863700
C	1.92121700	-1.12409300	-0.07665000
N	-1.26088400	-2.48979400	-0.46336600
N	0.98564800	-2.05735000	-0.29572800
C	-2.38179500	4.24881200	-0.92551900
C	-3.53821700	2.64876900	0.44466600
N	3.19899600	-1.55417500	-0.08561700
C	-2.63866900	-2.26206000	-0.19849200
C	-3.55955900	3.78182500	-0.35250100
H	-4.44128400	2.26684500	0.90553800
C	4.38400000	-0.77537200	-0.11415600
C	-3.36428000	-1.21761100	-0.78619600
C	-3.29906800	-3.16328200	0.63464800
C	4.64642300	0.25616200	0.79597100
C	5.36467700	-1.13129300	-1.03960700
C	-4.71954500	-1.08134100	-0.50373900

C	-4.65464900	-3.03680800	0.89094000
C	5.86970500	0.91746200	0.74462200
C	6.58748500	-0.48209000	-1.07079900
C	-5.36706800	-1.98828600	0.31970500
H	-5.25258300	-0.25803500	-0.96396100
C	6.84076600	0.54804100	-0.17173600
H	6.04424000	1.71444400	1.45722600
H	-0.96681400	-3.45949900	-0.47375600
H	3.30572600	-2.52020600	-0.37220300
H	0.90118500	2.44842300	0.36589000
H	-4.49579700	4.29606500	-0.52974500
H	-2.38634800	5.13189600	-1.55127300
H	7.79048500	1.06777200	-0.18709200
H	7.33592500	-0.77893200	-1.79408800
H	-6.42503400	-1.87259600	0.51905300
H	-5.14864000	-3.75039200	1.53764600
O	3.76577500	0.61223700	1.77604000
H	2.86146200	0.61161600	1.37923900
O	-2.38206300	0.92251800	1.56330300
H	-1.81391300	0.20999200	1.20277200
O	-2.80973200	-0.33806700	-1.67290000
H	-1.92641500	-0.07903800	-1.33598500
H	-2.72731300	-3.97039300	1.07799500
H	-0.26866000	3.91682400	-1.14386300
H	5.15165600	-1.93661300	-1.73308800

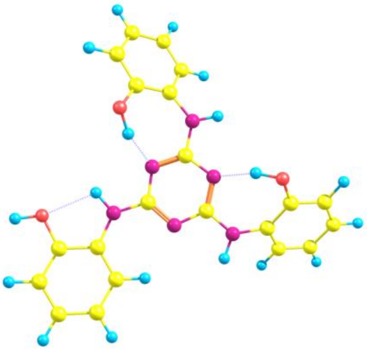

11-GS3

C	0.45523500	1.16304700	0.08544600
N	0.77501700	2.47292200	0.12776500
N	-0.82102700	0.80382400	0.01997500
C	-0.06919500	3.58506900	-0.12059300
N	1.49033000	0.30399900	0.12581600
C	-1.04740500	-0.53088400	0.00991000
C	0.42358800	4.59111700	-0.95137900
C	-1.31498900	3.75773200	0.49607800
C	1.15084300	-0.98494400	0.10114100
N	-2.35263900	-0.86678400	-0.03954600
N	-0.10410700	-1.45978100	0.04230500
C	-0.29724800	5.75250400	-1.17291600
C	-2.04236800	4.91878400	0.25116600
N	2.12637500	-1.91618000	0.14392700
C	-2.98290000	-2.12063100	-0.06706700
C	-1.53765300	5.91676600	-0.56606700
H	-3.00422900	5.02493100	0.73805200
C	3.52178400	-1.73673100	-0.03190500

C	-4.38840000	-2.08011200	-0.09076900
C	-2.34904100	-3.35971500	-0.07909700
C	4.27035100	-0.77878000	0.66437700
C	4.18037600	-2.63297500	-0.87388400
C	-5.13081900	-3.24600100	-0.12387300
C	-3.10274100	-4.52989200	-0.11132300
C	5.64951600	-0.72743200	0.48354400
C	5.55534400	-2.58879000	-1.03123700
C	-4.48685600	-4.47869500	-0.13381200
H	-6.21271600	-3.17752400	-0.14117600
C	6.29388800	-1.62974700	-0.34657500
H	6.20299100	0.02630700	1.03044500
H	-2.98274000	-0.07328800	-0.06200700
H	1.79263200	-2.86392000	0.01354100
H	1.76898300	2.66026700	0.06041500
H	-2.11437000	6.81781500	-0.73303700
H	0.10778200	6.52246700	-1.81678400
H	7.36924100	-1.58007100	-0.46291100
H	6.04493400	-3.29825000	-1.68579700
H	-5.07260000	-5.38872500	-0.15931400
H	-2.59114100	-5.48388200	-0.11984300
O	3.70979100	0.08385500	1.55992400
H	2.84651300	0.38819100	1.18000200
O	-1.83130100	2.85156600	1.37533600
H	-1.64272000	1.94785600	1.01562700
O	-4.95461200	-0.83661900	-0.07967400
H	-5.91887700	-0.90135200	-0.11041000
H	-1.27300300	-3.40786600	-0.06364500
H	1.39215200	4.44749300	-1.41660900
H	3.59064700	-3.37501100	-1.39992500

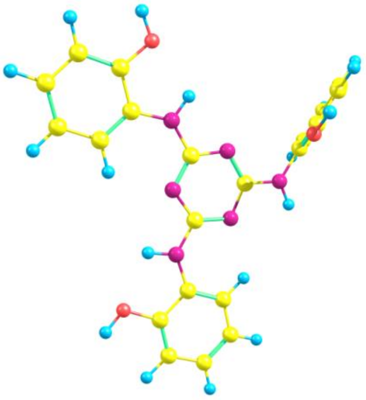

11-GS4

C	0.29728200	-1.44932700	-0.12061300
N	0.68597100	-2.74357700	-0.15240200
N	1.22430900	-0.50051900	-0.14806500
C	2.03423000	-3.17619800	-0.19719300
N	-1.03421200	-1.24602500	-0.07289700
C	0.73744400	0.75316100	-0.10186800
C	2.87056100	-2.97115300	0.90477200
C	2.52216000	-3.84213900	-1.31356700
C	-1.38997500	0.02895300	-0.04100100
N	1.69222400	1.71799600	-0.11874100
N	-0.54553800	1.07985900	-0.04570100
C	4.19096500	-3.40291800	0.85877300

C	3.83430800	-4.29562500	-1.35165600
H	1.85669100	-3.99684000	-2.15459100
N	-2.70037900	0.37865100	0.00211800
C	1.59583500	3.11440100	-0.09183000
C	4.66746200	-4.06546000	-0.26429800
C	-3.86395800	-0.39993900	0.01811200
C	0.42040300	3.86105800	-0.10722900
C	2.82451500	3.79950900	-0.05554000
C	-3.92529700	-1.79058800	0.05658800
C	-5.07034400	0.32465500	0.00230300
C	0.47286100	5.25269600	-0.08105700
H	-0.52962400	3.35396800	-0.13707800
C	2.87050800	5.18055100	-0.03189400
C	-5.15832300	-2.43821200	0.07285200
H	-3.01190100	-2.36150900	0.07116700
C	-6.29085100	-0.32370500	0.01945200
C	1.68873400	5.91498400	-0.04436000
C	-6.33862600	-1.71388200	0.05533100
H	2.63638100	1.35385700	-0.14452000
H	-2.85446700	1.37938300	0.00956800
H	-0.04865200	-3.42879600	-0.26949200
O	3.94835900	3.02040900	-0.04585300
H	4.74077500	3.57394900	-0.03385400
O	2.33790500	-2.36030100	1.99697000
H	3.00411200	-2.28095700	2.69367600
O	-4.95240400	1.68658200	-0.03049500
H	-5.82463300	2.10350600	-0.02836500
H	5.69579900	-4.40445000	-0.28371800
H	4.83101600	-3.23173100	1.71717400
H	-7.29667300	-2.21772200	0.06845300
H	-7.19828900	0.26955000	0.00548100
H	1.72870000	6.99663700	-0.02482800
H	3.83693000	5.67147000	-0.00408900
H	-5.18224800	-3.52027800	0.10193500
H	-0.45348600	5.81302900	-0.09252500
H	4.20269400	-4.81621200	-2.22602100

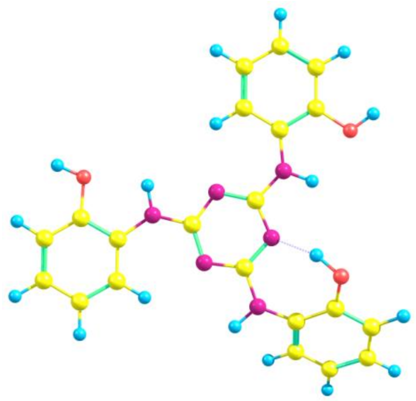

11-GS5

C	-0.06504500	1.39458100	0.17455700
N	-0.25503800	2.73548000	0.19795000
N	-1.13017300	0.59621200	0.16539700
C	-1.45040900	3.44833600	-0.06957100
N	1.21036600	0.98553800	0.17396500
C	-0.85197100	-0.72567800	0.12775900
C	-1.37063100	4.53752300	-0.93735300
C	-2.66966300	3.16892500	0.56214100
C	1.36245900	-0.33359300	0.13344600
N	-1.94429200	-1.52242300	0.12141500
N	0.36538200	-1.24006200	0.09287200
C	-2.47227500	5.34013700	-1.18329300
C	-3.77723700	3.96795200	0.29352600
N	2.60489500	-0.86637100	0.11556300
C	-2.04877600	-2.92111100	0.07957000
C	-3.68278500	5.05347900	-0.56194600
H	-4.70808700	3.72574400	0.79206100
C	3.84437400	-0.20328200	0.14705700
C	-3.31207100	-3.42223000	-0.27654400
C	-1.03685700	-3.82106100	0.39976400
C	4.89756100	-0.80001500	-0.55878800
C	4.09032500	0.95220900	0.88108000
C	-3.54566300	-4.78462900	-0.32821800
C	-1.27496600	-5.19103700	0.34005200
C	6.16382900	-0.23653900	-0.54134300
C	5.35704300	1.52373400	0.88483200
C	-2.52247900	-5.67498200	-0.02114900
H	-4.53179100	-5.13736900	-0.60874800
C	6.39285900	0.93032000	0.17680400
H	6.96121600	-0.71845300	-1.09573800
H	-2.82395000	-1.02278900	0.06454100
H	2.63271800	-1.86265800	-0.06388700
H	0.59910500	3.26783000	0.08277000
H	-4.55395100	5.66904300	-0.74797500
H	-2.38454200	6.18239500	-1.85746300
H	7.38280900	1.36874600	0.18151900
H	5.53204300	2.42638800	1.45605600
H	-2.70844100	-6.74054200	-0.06598600
H	-0.47438300	-5.87609300	0.58844500
O	4.60193400	-1.94626000	-1.23931100
H	5.39313700	-2.29543100	-1.67240500
O	-2.80855500	2.16437800	1.47457700
H	-2.29224500	1.38597700	1.13634000
O	-4.27167600	-2.49132300	-0.55845800
H	-5.12035800	-2.92194100	-0.72904300
H	-0.06724600	-3.44948900	0.68913200
H	-0.42034700	4.74749200	-1.41506700
H	3.28696800	1.39995000	1.44652800

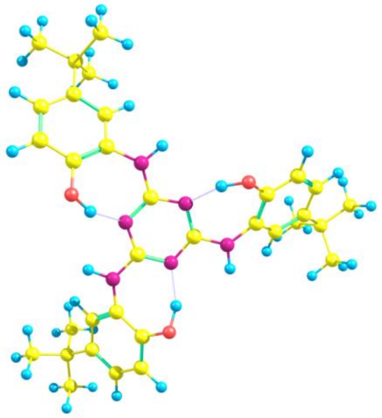

12-GS1

C	1.07563600	0.74020800	-0.84417100
N	2.15691000	1.54108200	-0.85732200
N	1.24182200	-0.58059100	-0.84383200
C	3.51476600	1.18087000	-0.65396600
N	-0.11690600	1.36598100	-0.83854300
C	0.10383400	-1.30035500	-0.84978100
C	4.26007200	1.97323300	0.21363400
C	4.14471700	0.13962500	-1.34647700
C	-1.17743000	0.56178600	-0.84588200
N	0.25664500	-2.63732500	-0.86697900
N	-1.12292900	-0.78372600	-0.84486900
C	5.62335300	1.77398900	0.42224900
C	5.49684900	-0.08066500	-1.12900900
N	-2.41182900	1.09706000	-0.86323600
C	-0.73325500	-3.63339900	-0.66023700
C	6.22957500	0.72550400	-0.26919200
H	5.97266000	-0.89133200	-1.66783400
C	-2.78058000	2.45213500	-0.65599900
C	-1.95306600	-3.65769900	-1.34802500
C	-0.41668900	-4.67424400	0.20721900
C	-2.19055200	3.52135900	-1.34115100
C	-3.84309200	2.69758400	0.20844200
C	-2.81920200	-4.71835800	-1.12709100
C	-1.27127200	-5.75416000	0.42021400
C	-2.67710600	4.80144600	-1.12017200
C	-4.35226900	3.97711400	0.42139700
C	-2.48454700	-5.75527000	-0.26738300
H	-3.76066100	-4.72582100	-1.66332900
C	-3.74492700	5.02917600	-0.26347100
H	-2.21050800	5.62110500	-1.65346100
H	1.21513300	-2.94674200	-0.75448800
H	-3.16018700	0.42143400	-0.75743500
H	1.94714100	2.52637500	-0.74430300
H	7.28417200	0.51934100	-0.14595400
H	-4.09369400	6.04504200	-0.13672900
H	-3.18993600	-6.56535200	-0.14114300
O	-1.19277300	3.35342700	-2.25944200
H	-0.56401800	2.68421800	-1.89617300
O	3.49898000	-0.63378600	-2.26942200
H	2.60595900	-0.84521600	-1.90611200
O	-2.30290100	-2.71197600	-2.27020100
H	-2.03939800	-1.83147600	-1.90924900
H	0.53848100	-4.62440800	0.71939200
H	3.74070600	2.77543400	0.72699400
H	-4.28033400	1.84486000	0.71681200
C	-0.85370400	-6.86479300	1.38352600
C	0.45865500	-7.49509400	0.89811500
C	-0.64058800	-6.27070100	2.78243600
C	-1.91148400	-7.96380300	1.47975000
H	0.33845600	-7.92116000	-0.10099300
H	1.26744600	-6.76272400	0.86200000
H	0.76074400	-8.29624400	1.57740300
H	-1.56032800	-5.81216100	3.15399600
H	-0.34438800	-7.05768200	3.48070900
H	0.14313000	-5.51053500	2.77960000
H	-1.56770400	-8.73346200	2.17401100
H	-2.86264100	-7.57728400	1.85291100
H	-2.08826300	-8.43970200	0.51236900
C	6.37928200	2.69266300	1.38195500
C	6.27792300	4.14122100	0.88563000
C	5.75503400	2.59457200	2.78054500
C	7.85757300	2.31844600	1.48461800
H	6.71256800	4.24124200	-0.11200200
H	5.24052200	4.47855900	0.84182000
H	6.81979400	4.80613800	1.56303500
H	5.80546600	1.57004100	3.15744000
H	6.29531100	3.24179200	3.47607800
H	4.70854600	2.90581600	2.77595700
H	8.35336600	3.00045000	2.17857800
H	7.99156400	1.30168600	1.86110700
H	8.36217800	2.39813500	0.51899700
C	-5.52740000	4.16914000	1.37981000
C	-6.72994600	3.35745200	0.87936400
C	-5.13199200	3.67425300	2.77761600
C	-5.94344800	5.63602200	1.48566200
H	-7.03166600	3.68542400	-0.11844300
H	-6.50311700	2.29043000	0.83424300
H	-7.57834000	3.49295500	1.55498300
H	-4.27253200	4.23171100	3.15811200
H	-5.96459500	3.81380300	3.47177900
H	-4.87484300	2.61322200	2.77015500
H	-6.78282600	5.72249300	2.17886700
H	-5.13057100	6.25980400	1.86470800
H	-6.26354100	6.03557100	0.52056200

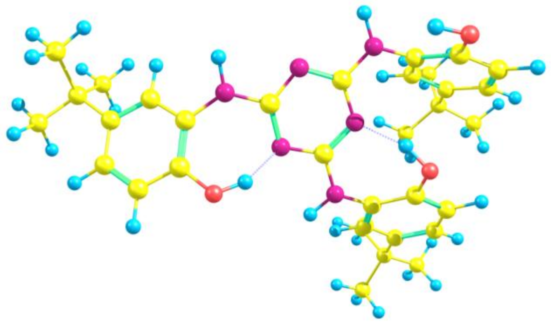

12-GS2_3H

C	0.33827600	0.35768200	0.57362800
N	0.07282800	1.58826800	1.04984000
N	-0.66577200	-0.33249200	0.02416900
C	-1.10789600	2.33814300	0.79119400
N	1.59077700	-0.09615400	0.69263500
C	-0.34864900	-1.55986700	-0.42427900
C	-0.96134300	3.62155700	0.27748700
C	-2.38994000	1.85571500	1.08328300
C	1.79850900	-1.31992100	0.18649900
N	-1.34069000	-2.29308000	-0.97046100
N	0.86681700	-2.09604900	-0.38248700

C	-2.05217900	4.45549900	0.03466800
C	-3.48348100	2.66660800	0.82164300
N	3.04095100	-1.83961100	0.25110500
C	-2.73262100	-2.02335200	-0.86330100
C	-3.32071300	3.94921600	0.31522400
H	-4.47049000	2.27724600	1.04173100
C	4.25365400	-1.17304800	0.56476000
C	-3.30485100	-0.81279400	-1.27522300
C	-3.55464000	-3.03794300	-0.38521300
C	4.42505200	-0.39550000	1.71668300
C	5.33839400	-1.39135800	-0.27913100
C	-4.67713400	-0.65091000	-1.16390300
C	-4.93893900	-2.89643100	-0.29476000
C	5.67416900	0.15085700	1.97089900
C	6.60284700	-0.86452200	-0.02156100
C	-5.48533000	-1.67671300	-0.69183400
H	-5.10518400	0.29444500	-1.47590300
C	6.74952400	-0.08514100	1.12524000
H	5.79371100	0.75316600	2.86357800
H	-1.09037400	-3.25397400	-1.17308900
H	3.14395800	-2.71809700	-0.24310100
H	0.88639300	2.12158300	1.33462300
H	-4.20463100	4.54713700	0.13895300
H	7.70558600	0.35400800	1.37590000
H	-6.55183400	-1.50551100	-0.63641200
O	3.42815800	-0.19858900	2.63105100
H	2.59407600	-0.03351300	2.12949200
O	-2.61131700	0.63554900	1.66215400
H	-2.03595800	-0.02084100	1.21742600
O	-2.57590000	0.20471900	-1.82904800
H	-1.75570200	0.31194500	-1.30329800
H	-3.07877200	-3.96392200	-0.08101700
H	0.04618800	3.96361700	0.06708300
H	5.17022300	-2.00446500	-1.15827800
C	-1.81523600	5.85588600	-0.53018500
C	-5.78005400	-4.05840200	0.23330500
C	7.75065800	-1.15484200	-0.98851900
C	-0.92575300	6.65083900	0.43509800
H	0.04633000	6.17312200	0.57163300
H	-0.75412300	7.65576200	0.04122600
H	-1.40177500	6.74423100	1.41426200
C	-3.12367300	6.62104800	-0.72500500
H	-3.78646700	6.11455400	-1.43036500
H	-3.65889400	6.75176100	0.21834300
H	-2.90375300	7.61278400	-1.12571400
C	-1.11043400	5.74355500	-1.88894500
H	-0.14133600	5.24892500	-1.79868100
H	-1.71964700	5.17558700	-2.59632400
H	-0.94246300	6.74008700	-2.30514500
C	9.05187600	-0.48604600	-0.54628300
H	9.83914500	-0.72086000	-1.26572300
H	8.95222200	0.60096800	-0.50231200
H	9.37689600	-0.84280400	0.43376400
C	7.38673500	-0.63255400	-2.38489700
H	6.49022500	-1.11866500	-2.77444700
H	7.20882500	0.44538500	-2.36210500
H	8.20562900	-0.82930500	-3.08150100
C	7.98305900	-2.66997200	-1.06173900
H	8.24224000	-3.07089700	-0.07878300
H	7.09602000	-3.19475200	-1.42175200
H	8.80524700	-2.88856800	-1.74788600
C	-5.33411500	-4.40463800	1.66041600
H	-4.28660400	-4.71053100	1.69111300
H	-5.45962200	-3.54674100	2.32550400
H	-5.93674400	-5.22951500	2.04895700
C	-5.57777300	-5.28314700	-0.66892400
H	-6.17801900	-6.11949800	-0.30188700
H	-5.88582700	-5.06510700	-1.69443700
H	-4.53338900	-5.60064800	-0.68595500
C	-7.26956700	-3.71757300	0.26108300
H	-7.47710700	-2.86752700	0.91516500
H	-7.64909500	-3.48577100	-0.73672300
H	-7.82760400	-4.57599600	0.64076100

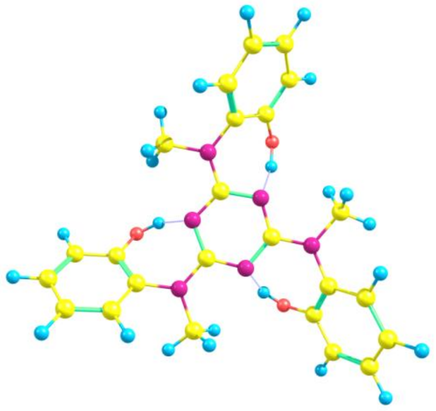

13-GS1

C	0.92257400	-0.92628600	0.09703600
N	1.87274400	-1.88839700	0.09037100
N	1.32580900	0.35301200	0.09930000
C	3.24974700	-1.59562100	-0.15609000
N	-0.35575800	-1.32484100	0.10131300
C	0.34156700	1.26071900	0.09889100
C	3.88880000	-2.23368100	-1.21734500
C	3.99355100	-0.75190600	0.67681500
C	-1.26215500	-0.33594700	0.10139800
N	0.69991700	2.56463500	0.09184600
N	-0.96797400	0.97033000	0.10494600
C	5.24476100	-2.05618100	-1.44390100
C	5.35168300	-0.57001700	0.44092100
N	-2.57066900	-0.67704700	0.09830200
C	-0.24222300	3.61039100	-0.15620300
C	5.97984000	-1.22551700	-0.60611100
H	5.90054800	0.08826700	1.10336000
C	-3.00811800	-2.01436200	-0.15314900
C	-1.34440700	3.83451000	0.67673600
C	-0.00956600	4.48036700	-1.21958700

C	-2.64697400	-3.08559300	0.67202400
C	-3.88509000	-2.24158700	-1.21200200
C	-2.18059600	4.91973100	0.43920100
C	-0.84097600	5.56572300	-1.44794700
C	-3.17169900	-4.35057900	0.43144100
C	-4.41223600	-3.50252200	-1.44334800
C	-1.92724100	5.78919500	-0.60978900
H	-3.02463400	5.06770000	1.10186700
C	-4.05844900	-4.55959600	-0.61279100
H	-2.87446200	-5.15918500	1.08811700
H	7.03999900	-1.08021000	-0.77196100
H	5.72231800	-2.56377200	-2.27207700
H	-4.46504900	-5.54880000	-0.78222200
H	-5.09433400	-3.65672600	-2.26951000
H	-2.58294500	6.63456200	-0.77696300
H	-0.64063800	6.23112700	-2.27781400
O	-1.81507000	-2.92444300	1.74402500
H	-1.06718600	-2.36532300	1.44308300
O	3.43479000	-0.12063000	1.75239600
H	2.57744000	0.24970900	1.45308800
O	-1.61141400	3.03672200	1.75360900
H	-1.50198500	2.10855000	1.45590400
H	0.84056900	4.30012900	-1.86657400
H	3.30902700	-2.88121600	-1.86417100
H	-4.15753400	-1.41162800	-1.85290900
C	-3.57719900	0.38743400	0.04475900
H	-3.40858300	1.10014600	0.84955300
H	-3.54924600	0.91664300	-0.90928600
H	-4.55498000	-0.06509800	0.18203400
C	2.12427700	2.90519700	0.02633000
H	2.66506500	2.39927900	0.82352300
H	2.55967800	2.62087300	-0.93321500
H	2.22214900	3.97768900	0.16742600
C	1.45587100	-3.29242600	0.02891700
H	0.74877900	-3.50583600	0.82787800
H	0.99043100	-3.52977400	-0.92928000
H	2.33619500	-3.91282100	0.17011700

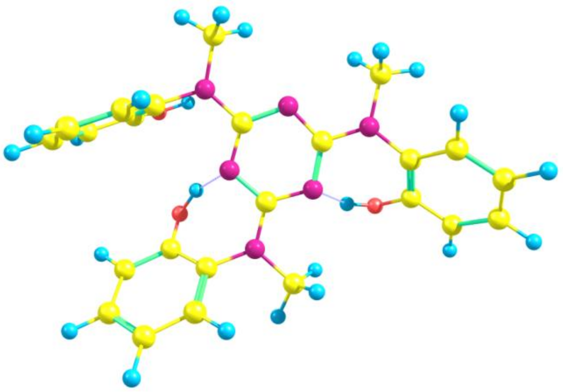

13-GS2_3H

C	0.30705100	0.62542600	0.14703800
N	0.00170600	1.92953000	0.32644800
N	-0.70922500	-0.22234000	-0.06435400
C	-1.30039800	2.43440000	0.01493800
N	1.59055200	0.26054300	0.19826200
C	-0.36901600	-1.51275300	-0.22723600
C	-1.41420400	3.43060100	-0.95254500
C	-2.45237800	1.99859300	0.68147900
C	1.82301600	-1.04482400	-0.03814700
N	-1.38081200	-2.39598200	-0.39138300
N	0.88197700	-1.97145000	-0.25174100
C	-2.64254800	4.00146800	-1.24754100
C	-3.68333800	2.56908000	0.37657000
N	3.11185800	-1.45684500	-0.05050800
C	-2.73423600	-2.04131100	-0.09146500
C	-3.78069000	3.57239800	-0.57459900
H	-4.55604100	2.21081800	0.90936200
C	4.21316300	-0.54652400	-0.09647700
C	-3.41578000	-1.02848500	-0.77861200
C	-3.41057900	-2.76656100	0.88765800
C	4.44184500	0.40670700	0.90338700
C	5.13699800	-0.67248100	-1.13286500
C	-4.74873300	-0.76799400	-0.47988600
C	-4.74142100	-2.50757500	1.17671700
C	5.58073300	1.20326900	0.85125500
C	6.27499300	0.11763400	-1.17570100
C	-5.41450000	-1.50729000	0.48488000
H	-5.24715600	0.02295200	-1.02740400
C	6.50052300	1.05497900	-0.17425100
H	5.72898500	1.93116200	1.63969900
H	-4.74552000	4.01156100	-0.79554500
H	-2.70858800	4.77582400	-2.00098200
H	7.38686000	1.67671400	-0.19537800
H	6.98022000	0.00036000	-1.98842500
H	-6.45421300	-1.29600000	0.70144200
H	-5.24747400	-3.08376700	1.94062600
O	3.59352400	0.56247500	1.96206700
H	2.68127700	0.55123200	1.59261100
O	-2.41417800	1.04137000	1.65747700
H	-1.85307800	0.30958100	1.32782200
O	-2.82459300	-0.28936900	-1.76586300
H	-1.94476600	-0.01309300	-1.43269000
H	-2.87886500	-3.54410700	1.42287300
H	-0.52157700	3.75871700	-1.47175000
H	4.95664900	-1.40720900	-1.90822100
C	3.39394800	-2.86830100	-0.32570900
H	2.77291900	-3.49374500	0.31094700
H	3.19635200	-3.12132600	-1.36889200
H	4.43938000	-3.05555300	-0.09727800
C	1.09104000	2.89364100	0.49813400
H	1.73295400	2.58367800	1.32074300
H	1.69247600	2.98416300	-0.40818100
H	0.65393900	3.85734800	0.74373500
C	-1.05947900	-3.81893100	-0.51658100
H	-0.30171000	-3.95356500	-1.28489600
H	-0.68679300	-4.23111500	0.42304400
H	-1.96271700	-4.34562200	-0.81244500

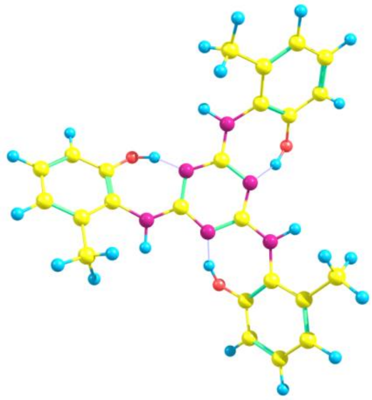

14-GS1

C	-0.76199800	-1.05918700	-0.22682200
N	-1.51090600	-2.17672900	-0.24429900
N	-1.36532900	0.12763800	-0.22406900
C	-2.91289600	-2.29111700	-0.05546000
N	0.57182300	-1.24654200	-0.22358500
C	-0.53630300	1.18917400	-0.22687900
C	-3.36913700	-3.24915200	0.86256500
C	-3.82902700	-1.54787200	-0.80879300
C	1.29790100	-0.13061700	-0.22639200
N	-1.12994300	2.39646800	-0.24392300
N	0.79305300	1.11825300	-0.22496300
C	-4.73796800	-3.44851900	1.00216400
C	-5.19333200	-1.75432400	-0.63947100
N	2.64027900	-0.21991800	-0.24333700
C	-0.52782200	3.66761100	-0.05514100
C	-5.64616800	-2.70963600	0.25402000
H	-5.87887100	-1.16203300	-1.23285800
C	3.44063700	-1.37653600	-0.05475500
C	0.57337400	4.08907700	-0.80921500
C	-1.12885700	4.54197900	0.86313300
C	3.25585000	-2.54062900	-0.80950700
C	4.49804500	-1.29319900	0.86372100
C	1.07721900	5.37363900	-0.64003800
C	-0.61697600	5.82709900	1.00223700
C	4.11716100	-3.61877000	-0.64120400
C	5.35579100	-2.37863800	1.00210600
C	0.47691700	6.24367400	0.25364700
H	1.93271000	5.67102200	-1.23383800
C	5.17077600	-3.53379200	0.25250300
H	3.94734500	-4.50799800	-1.23561800
H	-2.13961100	2.36174900	-0.16410000
H	3.11499700	0.67226100	-0.16626200
H	-0.97591400	-3.03382800	-0.16612300
H	-6.70994300	-2.87247200	0.37534700
H	-5.09039300	-4.18905700	1.71000900
H	5.84401700	-4.37347900	0.37296100
H	6.17300000	-2.31410700	1.71036600
H	0.86814500	7.24624300	0.37460300
H	-1.08172100	6.50290300	1.71001100
O	2.27448800	-2.64423000	-1.75459600
H	1.44961800	-2.26269700	-1.37193100
O	-3.42856500	-0.64542400	-1.75338300
H	-2.68570300	-0.12179200	-1.37007800
O	1.15341700	3.29148500	-1.75493200
H	1.23504600	2.38592200	-1.37359700
C	4.70152800	-0.04444800	1.67368400
H	4.99259300	0.79991000	1.04174500
H	3.78728700	0.24039800	2.19887100
H	5.49035400	-0.19693000	2.40858200
C	-2.31362900	4.09423400	1.67107800
H	-3.18811100	3.92037800	1.03699400
H	-2.10303400	3.16205500	2.19978000
H	-2.57973100	4.85538100	2.40277300
C	-2.38885400	-4.05116500	1.67056700
H	-1.80179500	-4.72255300	1.03690000
H	-1.68623100	-3.40245600	2.19820800
H	-2.91501000	-4.66097400	2.40328400

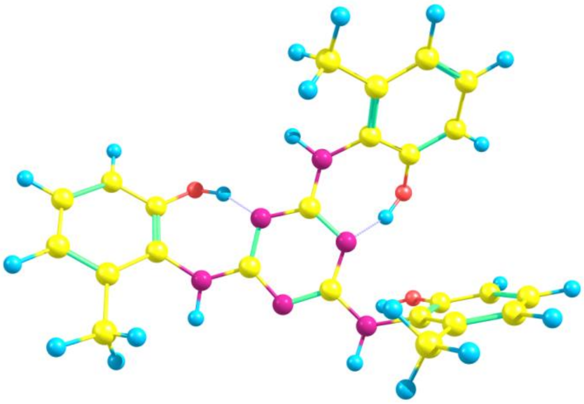

14-GS2_3H

C	-0.30052800	0.60256800	-0.25800000
N	0.00524900	1.88118100	-0.54511200
N	0.70445100	-0.22253400	0.04916600
C	1.25934000	2.50310300	-0.29098500
N	-1.58685400	0.23983000	-0.31142600
C	0.35230400	-1.49044300	0.32290000
C	1.27148200	3.65945000	0.50166900
C	2.45060400	2.01619200	-0.84227700
C	-1.82495500	-1.03893000	0.01602700
N	1.34799200	-2.35227600	0.61442100
N	-0.89565700	-1.94744900	0.34289900
C	2.48070100	4.31028500	0.72227300
C	3.64998500	2.67156200	-0.59057900
N	-3.10127800	-1.47194200	0.01136100
C	2.73238700	-2.13440500	0.37351200
C	3.66287500	3.82165600	0.18040200
H	4.55658900	2.26653400	-1.02320700
C	-4.28642800	-0.69744000	-0.08455400
C	3.42057000	-1.04835700	0.92913700
C	3.42028500	-3.07916200	-0.40200600
C	-4.50177100	0.20566000	-1.13214800
C	-5.29470000	-0.92634100	0.86418700
C	4.78157700	-0.89480500	0.69230600

C	4.78627400	-2.91627600	-0.60661100
C	-5.71284600	0.88131000	-1.22734300
C	-6.50334500	-0.25039700	0.73876700
C	5.46458200	-1.83218400	-0.06397300
H	5.28394700	-0.03914100	1.12673800
C	-6.71471400	0.64568700	-0.30169900
H	-5.84872600	1.57730000	-2.04588000
H	1.05186200	-3.31560600	0.71718800
H	-3.21080300	-2.42438600	0.33900400
H	-0.79296100	2.49291000	-0.67114900
H	4.59846400	4.33396200	0.36758400
H	2.49111400	5.20418000	1.33422700
H	-7.65939100	1.16853900	-0.38537100
H	-7.28176300	-0.42790800	1.47099800
H	6.52669800	-1.71315100	-0.23879600
H	5.31762300	-3.64641700	-1.20514200
O	-3.56874300	0.41929700	-2.10774400
H	-2.69048000	0.49886500	-1.66653300
O	2.48225900	0.92506100	-1.66752000
H	1.92701900	0.22406700	-1.26800800
O	2.80966000	-0.13415100	1.74310500
H	1.95117400	0.10849300	1.33458800
C	-5.06898200	-1.89522400	1.98969500
H	-4.96474100	-2.91985800	1.62113600
H	-4.15869500	-1.65358800	2.54282200
H	-5.91057200	-1.87394600	2.68026500
C	2.68649600	-4.24812300	-0.99553900
H	2.30389100	-4.91609100	-0.21841000
H	1.83254900	-3.91742800	-1.59092900
H	3.35153000	-4.82629000	-1.63503200
C	-0.00528900	4.18081100	1.09688900
H	-0.69676900	4.52291400	0.32162600
H	-0.51737800	3.40677400	1.67264700
H	0.20169000	5.02276900	1.75546400

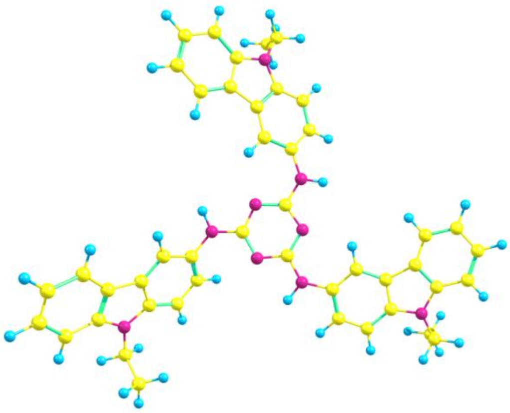

4-GS1

C	0.76881100	0.97357400	-0.06964900
N	1.38973000	2.17932100	0.06334000
N	-0.55797500	0.89939200	-0.20066300
C	0.90365400	3.50558000	0.09472600
N	1.62133500	-0.08408400	-0.05312200
C	-1.03963800	-0.36442000	-0.31806400
C	-0.43992300	3.86808900	-0.04880400
C	1.88840100	4.50948900	0.28162400
C	1.02809400	-1.27439000	-0.17287800
N	-2.39484400	-0.43861700	-0.45083100
N	-0.30697900	-1.47981200	-0.30870500
C	-0.77749900	5.22946800	-0.00281300
H	-1.19949700	3.11347200	-0.19333900
C	1.56499000	5.86039800	0.33011900
H	2.92829600	4.21049300	0.38769300
N	1.76299900	-2.42274600	-0.16492200
C	-3.22559800	-1.58878800	-0.51546300
C	0.21869700	6.22136300	0.18975800
C	-2.03702900	5.93391800	-0.11938600
H	2.34517500	6.60161700	0.47002000
C	3.14887600	-2.66805500	-0.04123900
C	-2.90326300	-2.69627700	-1.33125400
C	-4.42584200	-1.56865500	0.20112700
N	-0.37423100	7.47924900	0.21254200
C	-3.36363900	5.52391300	-0.31682800
C	-1.73760300	7.32120600	0.00886400
C	3.52978700	-4.03421500	-0.07316900
C	4.12650400	-1.67763600	0.10266000
C	-3.75128600	-3.79823200	-1.42262300
H	-1.98037200	-2.68848300	-1.89750000
C	-5.29749900	-2.66017600	0.10649900
C	0.33378200	8.75398500	0.30623300
C	-4.36745800	6.48933400	-0.38977600
H	-3.60458600	4.46852500	-0.41372900
C	-2.74465100	8.29406700	-0.06835500
C	4.85664800	-4.43352200	0.03649900
H	2.75750600	-4.79082800	-0.18802500
C	5.46956600	-2.06946600	0.21401600
C	-4.95268500	-3.77656900	-0.70307100
H	-3.47894700	-4.64153600	-2.04900800
C	-6.58326400	-2.96332400	0.69838500
C	0.77061100	9.30861300	-1.05034100
H	-0.32448300	9.46363200	0.81547500
H	1.20100000	8.60592800	0.95623600
C	-4.05537000	7.85897800	-0.26675900
H	-5.39921800	6.18663200	-0.54438900
H	-2.51932700	9.35221700	0.01935000
C	5.83346300	-3.44059300	0.18561900
H	5.11181200	-5.48776800	0.00415000
C	6.70105200	-1.32408200	0.36985300
N	-5.96783400	-4.72350700	-0.63940400
C	-6.95324000	-4.25191300	0.21718200
H	-0.09199600	9.48711600	-1.70098300
H	1.29373700	10.26100300	-0.90827200
H	1.45153800	8.61820600	-1.55928200
H	-4.85231300	8.59507800	-0.33025100
N	7.21129200	-3.56037700	0.33235900
C	7.75087400	-2.28587200	0.42871900
C	-5.95060600	-6.03754100	-1.27868200

C	-8.15606300	-4.85749300	0.60718400
C	7.97042500	-4.80712800	0.26889300
C	9.08999800	-1.89528000	0.57219200
C	-5.28156700	-7.11809400	-0.42828900
H	-6.98574900	-6.31200200	-1.50070600
H	-5.43806100	-5.93415900	-2.23944300
C	-8.98614600	-4.15216300	1.47914500
H	-8.43692100	-5.84295800	0.24957300
C	8.38210700	-5.19655000	-1.15152400
H	7.35718100	-5.59337500	0.71866900
H	8.85279800	-4.69200900	0.90491800
C	9.36306800	-0.53008800	0.66349700
H	9.89270000	-2.62495800	0.60952700
H	-4.23646700	-6.86647700	-0.21918900
H	-5.30168000	-8.07379200	-0.96407300
H	-5.80362300	-7.25042500	0.52525600
C	-8.63507900	-2.87416300	1.96002600
H	-9.92345500	-4.60188400	1.79557000
H	9.01897700	-4.42862100	-1.60319400
H	8.94528100	-6.13632700	-1.12816000
H	7.50542900	-5.34025000	-1.79194800
C	8.33438000	0.43299500	0.61102100
H	10.39364400	-0.20386600	0.77504800
C	-7.43594400	-2.27571500	1.57385600
H	-9.30532500	-2.35417300	2.63855400
C	7.00360300	0.04209700	0.46484700
H	8.58358500	1.48781900	0.68402100
H	-7.16482100	-1.29131000	1.94625400
H	6.21139000	0.78506400	0.42322500
H	3.85031600	-0.63341200	0.12618300
H	-4.67334900	-0.71184800	0.82254400
H	-2.86843000	0.44344400	-0.29528300
H	1.19081900	-3.25301100	-0.26499100
H	2.39398300	2.09151400	0.16388300

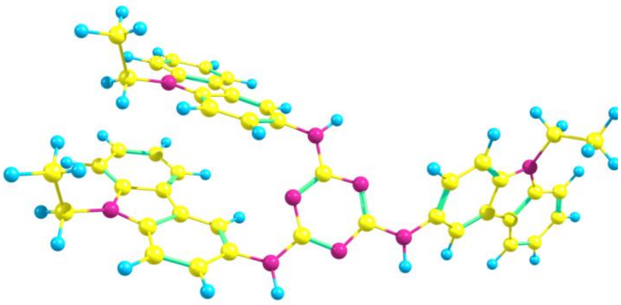

4-GS2

C	0.31115200	-2.44688900	0.28186200
N	-0.54504700	-3.49959100	0.14765100
N	1.61434700	-2.78987900	0.31812800
C	-1.95503100	-3.56238000	0.04940000
N	-0.17044500	-1.19497700	0.37198200
C	2.46557100	-1.75058500	0.43120700
C	-2.76209300	-2.49813200	-0.36437200
C	-2.53969700	-4.81785400	0.34937400
C	0.77559200	-0.24871200	0.49473800
N	3.78798100	-2.08916300	0.45407900
N	2.10916300	-0.46115200	0.51693800
C	-4.14644700	-2.70177300	-0.46190500
H	-2.32687800	-1.53736700	-0.60201500
C	-3.90954900	-5.03581400	0.24590400
H	-1.89505200	-5.63329300	0.66780900
N	0.40543200	1.05791600	0.60034800
C	4.92480000	-1.23811300	0.48053000
C	-4.71695000	-3.96732000	-0.16345700
C	-5.24436800	-1.84074400	-0.84920800
H	-4.32622600	-6.00901300	0.48432800
C	-0.89652300	1.62646400	0.59699600
C	5.01764700	-0.14993900	1.37687700
C	5.99879100	-1.55245700	-0.35714900
N	-6.09227500	-3.91618500	-0.36206500
C	-5.33013700	-0.50170500	-1.25728100
C	-6.42720600	-2.63068400	-0.76310400
C	-1.94674000	1.08039500	1.36823400
C	-1.09362600	2.80592800	-0.12650400
C	6.15733700	0.64997800	1.42851500
H	4.18917000	0.06333300	2.04091200
C	7.16009700	-0.77167000	-0.30304000
C	-7.03233800	-5.00206300	-0.09326100
C	-6.58056800	0.03363100	-1.56569100
H	-4.43500000	0.11008100	-1.32880700
C	-7.68602500	-2.09305500	-1.06775700
C	-3.20232600	1.68254600	1.40933500
H	-1.77033300	0.17997600	1.94359200
C	-2.34365900	3.43550600	-0.07972500
C	7.22862300	0.33878600	0.58149300
H	6.20561800	1.48056700	2.12529600
C	8.42452600	-0.81489000	-1.00655000
C	-7.54129800	-5.02270900	1.34859900
H	-7.86718700	-4.90029400	-0.79255800
H	-6.53143600	-5.94396800	-0.33554500
C	-7.74320000	-0.75824200	-1.46984700
H	-6.66230300	1.06949300	-1.88231800
H	-8.59007300	-2.68914400	-0.99483000
C	-3.39923300	2.86223200	0.68017700
H	-3.99403600	1.24687900	2.01009400
C	-2.87742700	4.63922100	-0.68096700
N	8.45679300	0.97220000	0.43298000
C	9.19704700	0.27515900	-0.51245300
H	-8.06897000	-4.09521600	1.59498000
H	-8.23848400	-5.85761100	1.48194200
H	-6.71641200	-5.15072800	2.05749400
H	-8.70791100	-0.32035300	-1.71187200
N	-4.53878600	3.64712800	0.55303800
C	-4.23258600	4.73558000	-0.25280300
C	8.93289800	2.10308600	1.22684600
C	10.49463700	0.52074700	-0.98343400
C	-5.80096000	3.43488100	1.25850400
C	-5.04701000	5.80587200	-0.64874500

C	9.63929800	1.68537400	2.51720100
H	9.60413600	2.69352200	0.59666300
H	8.07044500	2.73751000	1.45083400
C	11.00434300	-0.33427600	-1.96114800
H	11.08930800	1.34512400	-0.60291100
C	-5.84713100	4.10154700	2.63400700
H	-5.95249200	2.35580200	1.35061200
H	-6.60614300	3.81341200	0.62227600
C	-4.48928200	6.77186900	-1.48703800
H	-6.07679400	5.88874400	-0.31621700
H	8.96911700	1.11342500	3.16761300
H	9.96633200	2.57710100	3.06373100
H	10.52194500	1.07293600	2.30469200
C	10.24921400	-1.41382100	-2.46330400
H	12.00800300	-0.16332000	-2.34108500
H	-5.72214500	5.18644200	2.55227700
H	-6.81567500	3.90435000	3.10715700
H	-5.06169300	3.71061400	3.28942400
C	-3.15172600	6.68571600	-1.92436900
H	-5.10178900	7.61061100	-1.80682800
C	8.96021700	-1.65852100	-1.99030200
H	10.67822800	-2.06023400	-3.22365500
C	-2.34187000	5.62295200	-1.52463200
H	-2.75028300	7.45635200	-2.57627800
H	8.37915300	-2.49186000	-2.37651900
H	-1.31008600	5.55809800	-1.85991400
H	-0.27923500	3.22739500	-0.71003600
H	5.92726200	-2.39618300	-1.03861700
H	3.96461400	-3.06060500	0.22654900
H	1.17880400	1.71023100	0.54634600
H	-0.07634000	-4.39761000	0.17330400

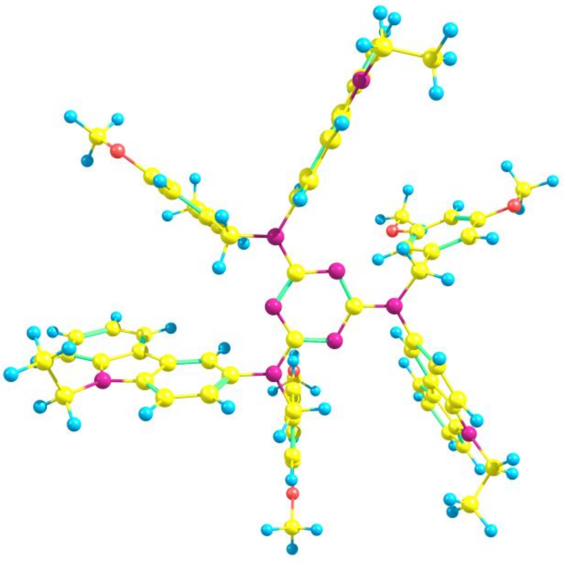

5-GS1

C	1.21839800	0.40390200	-1.54758400
N	1.05614000	-0.93199600	-1.52484100
C	-0.22558800	-1.34520100	-1.50230000
N	0.21907600	1.30711300	-1.54393200
C	-1.01921500	0.77988500	-1.52413400
N	-1.30203700	-0.53721100	-1.49779300
N	2.49986500	0.88906300	-1.57972000
N	-0.44787500	-2.69780400	-1.48815700
N	-2.07998700	1.64791200	-1.53443700
C	2.79636200	3.04575500	-0.43515200
C	3.30885100	4.28270200	-2.94827900
C	3.08104800	4.41568700	-0.50242200
C	2.75866800	2.30520500	-1.61578700
C	3.01691600	2.92319700	-2.85727500
C	3.34212400	5.02644100	-1.76083400
C	3.19012000	5.46179900	0.49241400
C	3.53829200	7.87986700	1.84408200
C	3.04994200	5.49380600	1.88722700
C	3.51207700	6.65549900	-0.21339600
C	3.68439900	7.87482700	0.45633700
C	3.22540700	6.70456700	2.55688000
N	3.61635800	6.37341600	-1.56992200
C	-4.05631200	0.82341600	-0.32376900
C	-5.47272300	0.66986300	-2.78928100
C	-5.38733600	0.38778100	-0.34697000
C	-3.43728600	1.16704200	-1.52477600
C	-4.14584600	1.09297400	-2.74235300
C	-6.09105800	0.31825100	-1.58170500
C	-6.31294500	-0.04566900	0.67836300
C	-8.53403100	-0.95703600	2.10356500
C	-6.22175800	-0.19162800	2.06996100
C	-7.53237900	-0.35730000	0.01313500
C	-8.65122700	-0.81938200	0.71991400
C	-7.33467400	-0.64713800	2.77643300
N	-7.38757700	-0.11830200	-1.34794900
C	1.29326100	-3.96271100	-0.29427600
C	2.06553600	-5.17221700	-2.75387200
C	2.34111700	-4.89174300	-0.32568100
C	0.64753500	-3.63288500	-1.48512000
C	1.03198600	-4.23921200	-2.69944200
C	2.71878400	-5.49623800	-1.55710100
C	3.21581400	-5.44321300	0.68766700
C	5.17466300	-6.85463900	2.08972000
C	3.34017100	-5.25224500	2.07122600
C	4.08160500	-6.35425100	0.01889100
C	5.07065600	-7.06391000	0.71401500
C	4.32044700	-5.96024900	2.76618700
N	3.75831400	-6.38771700	-1.33212400
C	3.66284600	0.00083700	-1.69643800
C	-1.79970400	-3.25875700	-1.57809700
C	-1.89463700	3.09780800	-1.66869100
C	4.46705000	-7.14666900	-2.36092800
C	5.65103100	-6.39092000	-2.96519900
C	-8.39588000	-0.38415100	-2.37236000
C	-8.34037200	-1.80902100	-2.92432100
C	3.86391100	7.35193600	-2.62721400
C	2.58791800	8.00192200	-3.16328100

H	2.59594800	2.56471000	0.51694200
H	3.49685800	4.74116000	-3.91370800
H	2.98024300	2.32202200	-3.76135200
H	3.66735600	8.81333200	2.38523700
H	2.80712200	4.58828400	2.43691900
H	3.92102400	8.78849500	-0.07959700
H	3.11843700	6.74541000	3.63710700
H	-3.50600500	0.88249800	0.60985400
H	-5.99893700	0.61271500	-3.73655800
H	-3.63865000	1.36701500	-3.66313600
H	-9.38815900	-1.31340000	2.67305400
H	-5.29687400	0.04592300	2.58889200
H	-9.57951000	-1.06643200	0.21474800
H	-7.27893000	-0.76690200	3.85464400
H	0.99431400	-3.49244500	0.63700200
H	2.35116500	-5.62176500	-3.69922200
H	0.51153000	-3.96353900	-3.61230000
H	5.93421900	-7.39330100	2.64988200
H	2.68298000	-4.56135800	2.59263300
H	5.73872300	-7.75206200	0.20598000
H	4.43010900	-5.82151000	3.83797900
H	4.29351300	0.36790400	-2.51224300
H	3.29630500	-0.98617800	-1.98369900
H	-1.78201100	-4.06675700	-2.31608400
H	-2.45981400	-2.47984700	-1.96551200
H	-2.55197700	3.45384800	-2.46810900
H	-0.86570400	3.27118900	-1.98863100
H	3.74371600	-7.40912200	-3.13814300
H	4.80132800	-8.08583700	-1.91123500
H	6.13593300	-7.01324800	-3.72570600
H	6.39523600	-6.14514700	-2.20036600
H	5.32449200	-5.46107300	-3.44266800
H	-8.25318800	0.34382000	-3.17601200
H	-9.37692000	-0.18145900	-1.93341500
H	-9.12074400	-1.94106100	-3.68220100
H	-8.50579200	-2.54682400	-2.13217600
H	-7.37203400	-2.01459800	-3.39263400
H	4.54115400	8.11094700	-2.22540000
H	4.40222600	6.84344900	-3.43203400
H	2.84297700	8.72136800	-3.94947800
H	2.05381900	8.53703300	-2.37108800
H	1.91299800	7.25346600	-3.59169500
C	-2.36698000	-3.79546500	-0.27186400
C	-3.26551000	-4.87661600	-0.33446600
C	-2.05519600	-3.21451500	0.95384800
C	-3.84517000	-5.35197200	0.84340300
H	-3.49237600	-5.32712500	-1.29356100
C	-2.64132700	-3.71142800	2.13085800
H	-1.36019000	-2.38473100	1.02360000
C	-3.53929900	-4.77717600	2.08883000
H	-4.00457200	-5.18199700	2.97930200
C	4.50326700	-0.11849600	-0.43476000
C	5.89521600	-0.17648900	-0.55274400
C	3.90792500	-0.22041400	0.82671500

C	6.68625500	-0.34596900	0.59062600
H	6.37712900	-0.08938800	-1.52237600
C	4.71048500	-0.37953400	1.96178900
H	2.83070500	-0.17223200	0.94626100
C	6.10802700	-0.44761100	1.86293800
H	6.72122100	-0.57033900	2.74428200
C	-2.17364600	3.89650000	-0.40494500
C	-2.81160500	5.13577400	-0.51646300
C	-1.76172900	3.43721900	0.85011300
C	-3.02594600	5.91585300	0.62683600
H	-3.14818800	5.50540500	-1.48090100
C	-1.99078800	4.22229600	1.98554700
H	-1.27158700	2.47623200	0.96460500
C	-2.62167900	5.47158100	1.89285400
H	-2.79638000	6.07081100	2.77486900
O	-3.65705400	7.11665100	0.41516600
O	-1.55853800	3.68587200	3.17386400
O	4.03731100	-0.46324800	3.15611500
O	8.04096000	-0.39412400	0.37180000
O	-2.26542500	-3.07743100	3.28909900
O	-4.73222100	-6.39921400	0.89285500
C	-5.10136900	-7.04283900	-0.33402100
H	-4.22933400	-7.49383300	-0.82006800
H	-5.80748300	-7.82553400	-0.05285200
H	-5.58770500	-6.33881700	-1.01795200
C	-2.81779100	-3.53198500	4.53133100
H	-3.90761700	-3.42101300	4.54190400
H	-2.54654900	-4.57578500	4.72427200
H	-2.37864200	-2.89300300	5.29889500
C	4.78969200	-0.61238200	4.36707200
H	4.04988700	-0.64940500	5.16817700
H	5.36816700	-1.54264800	4.36130400
H	5.45668900	0.24224000	4.52498000
C	8.91894700	-0.55853100	1.49305700
H	8.82418300	0.27771400	2.19441700
H	8.72516200	-1.50450500	2.01045100
H	9.92695100	-0.57139100	1.07592500
C	-3.91976900	7.96848100	1.53787000
H	-4.41862600	8.84767500	1.12752700
H	-4.58055800	7.47648200	2.25998400
H	-2.98875500	8.27193500	2.02913000
C	-1.77656100	4.41936000	4.38605200
H	-1.36608800	3.79660200	5.18232000
H	-1.25088300	5.38031200	4.36666700
H	-2.84572600	4.58084300	4.56258600

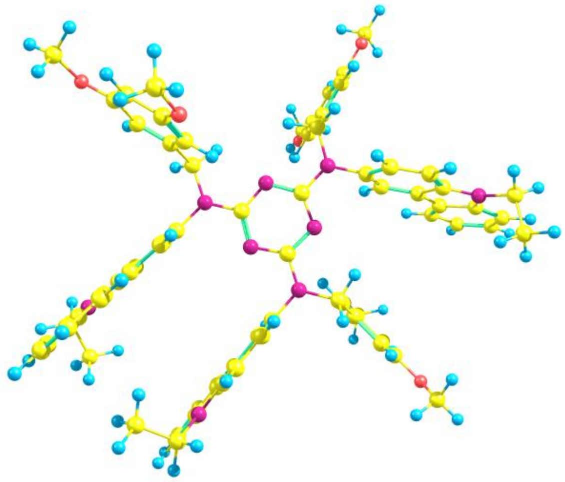

5-GS2

C	0.58248200	1.61234100	0.09388900
N	0.82529400	0.41663400	-0.47254300
C	-0.21245500	-0.11275000	-1.14086800
N	-0.59261100	2.26844600	0.04634500
C	-1.55807300	1.64376600	-0.65523300
N	-1.41540300	0.46948700	-1.29698600
N	1.60707800	2.17808700	0.80166800
N	-0.02667200	-1.34719100	-1.71084500
N	-2.78866000	2.24421700	-0.71864900
C	3.72666000	1.43608000	-0.20581200
C	4.50240000	0.36392000	2.31904900
C	4.96002300	0.79226600	-0.06137400
C	2.88236000	1.52307300	0.90021100
C	3.26855200	0.98887400	2.14575400
C	5.34944000	0.27766000	1.20741700
C	6.04809500	0.50505800	-0.97102500
C	8.43567900	-0.33201000	-2.14891400
C	6.25315700	0.73510300	-2.33796700
C	7.05215700	-0.15296800	-0.20487000
C	8.25402300	-0.57524600	-0.78722000
C	7.44831800	0.31353600	-2.92152800
N	6.62243600	-0.26451300	1.11141800
C	-4.76855200	0.78324700	-0.76741600
C	-5.02368700	1.26967700	-3.56252100
C	-5.80354700	0.18924100	-1.49614500
C	-3.86571300	1.61150000	-1.43424400
C	-4.00370600	1.85707900	-2.81519800
C	-5.92612700	0.43350400	-2.89164900
C	-6.86216800	-0.72952800	-1.14231300
C	-9.03006700	-2.48482000	-1.15470300
C	-7.25423400	-1.33786600	0.05777800
C	-7.57631200	-1.00107800	-2.34348100
C	-8.66205500	-1.88665100	-2.36134400
C	-8.33924700	-2.21446900	0.04487300
N	-7.01784100	-0.26851600	-3.38410200
C	1.56396600	-2.56910400	-0.27683900
C	3.22822000	-2.98473800	-2.55258900
C	2.74468000	-3.30689800	-0.16123800
C	1.21595600	-2.04836400	-1.52321600
C	2.04503500	-2.25101800	-2.64330400
C	3.56318700	-3.52446100	-1.30508100
C	3.37953200	-4.00238700	0.93653200
C	5.05895200	-5.53225800	2.55484600
C	3.06017100	-4.16411800	2.29100300
C	4.54851800	-4.61669000	0.40377500
C	5.39787000	-5.38587500	1.20924300
C	3.90476400	-4.92923700	3.09597300
N	4.63799100	-4.32746300	-0.95246700
C	1.49567700	3.50298200	1.41894300
C	-1.19832800	-2.17615800	-2.09195900
C	-3.15045500	3.34445700	0.19083500
C	5.74646300	-4.67229400	-1.83845600
C	6.80176600	-3.56982600	-1.90560900
C	-7.38309600	-0.38527400	-4.79323200
C	-6.63138900	-1.51105600	-5.50304400
C	7.31390800	-0.97411600	2.18473700
C	6.89404100	-2.43943600	2.27867900
H	3.41680500	1.84424200	-1.16185200
H	4.78391700	-0.04855200	3.28223000
H	2.58145500	1.06429300	2.98358800
H	9.35616700	-0.65808100	-2.62544700
H	5.48904000	1.23137100	-2.93032200
H	9.01336700	-1.08939900	-0.20731700
H	7.62118200	0.48011000	-3.98094300
H	-4.65383300	0.57899400	0.29005700
H	-5.10448400	1.45428800	-4.62870900
H	-3.28296700	2.50621100	-3.30367300
H	-9.86836000	-3.17621300	-1.14261100
H	-6.71536900	-1.13184900	0.97699900
H	-9.19821200	-2.10725700	-3.27874500
H	-8.65436000	-2.69788000	0.96525900
H	0.92407300	-2.40309600	0.58231100
H	3.85949400	-3.12892300	-3.42306500
H	1.74783500	-1.82323700	-3.59640900
H	5.70598500	-6.11902500	3.20120100
H	2.16873800	-3.69848800	2.70266400
H	6.29677200	-5.84313500	0.80898700
H	3.67446900	-5.06207700	4.14920000
H	1.87850600	3.44325100	2.44199300
H	0.43632500	3.75811300	1.47786200
H	-0.83327000	-2.93745600	-2.78422700
H	-1.91419600	-1.54662800	-2.62024600
H	-3.80668200	4.02661000	-0.35550900
H	-2.23755000	3.88484100	0.44440300
H	5.33055800	-4.87158200	-2.83087600
H	6.18253900	-5.60951300	-1.48326000
H	7.59804900	-3.85125500	-2.60359400
H	7.24964900	-3.40022200	-0.92248700
H	6.36134600	-2.62842000	-2.24813200
H	-7.18213200	0.57697600	-5.27251500
H	-8.46332400	-0.54646300	-4.84665800
H	-6.93734800	-1.56177800	-6.55384900

H	-6.84578800	-2.47744000	-5.03438400
H	-5.55021200	-1.34007000	-5.46671100
H	8.38889000	-0.88622000	2.00680500
H	7.10388600	-0.44894900	3.12156500
H	7.41285700	-2.92848900	3.11049500
H	7.14099900	-2.97371600	1.35725600
H	5.81635400	-2.52747700	2.44647700
C	-1.83465700	-2.81215800	-0.86928000
C	-1.32188000	-4.01783500	-0.35692300
C	-2.84604200	-2.13669600	-0.19172300
C	-1.81071500	-4.50005800	0.85892300
H	-0.53475400	-4.53355000	-0.89122800
C	-3.32939700	-2.63567700	1.02761700
H	-3.23767300	-1.20515000	-0.57557500
C	-2.81926700	-3.81920600	1.56330700
H	-3.16127100	-4.22623400	2.50705900
C	2.24854800	4.57695900	0.65324200
C	2.99319500	5.53053700	1.35108600
C	2.18610800	4.62366800	-0.74295600
C	3.66841500	6.53601600	0.64709300
H	3.06609400	5.50121500	2.43432000
C	2.86991700	5.62856500	-1.43343500
H	1.62616500	3.88445500	-1.30565700
C	3.61882500	6.59981700	-0.75177700
H	4.14786700	7.37193300	-1.29088800
C	-3.84858200	2.85276800	1.44915900
C	-5.12752300	3.31649100	1.76026100
C	-3.23883500	1.88748000	2.26067000
C	-5.79701300	2.81230900	2.88396800
H	-5.62582500	4.04808200	1.13118900
C	-3.92156900	1.38539000	3.37130000
H	-2.25216500	1.50089800	2.03049700
C	-5.20796100	1.84035500	3.70166400
H	-5.73089500	1.44571300	4.56044500
O	-7.05187400	3.32312900	3.10301300
O	-3.25860700	0.42486200	4.09363400
O	2.75610900	5.58886100	-2.80145800
O	4.37496800	7.42531100	1.41799600
O	-4.28968400	-1.86770300	1.63112300
O	-1.35174700	-5.64467600	1.46771700
C	-0.20406100	-6.29921400	0.90658000
H	0.65517200	-5.61966500	0.86681600
H	0.01784000	-7.12965400	1.57880100
H	-0.41924700	-6.68892500	-0.09485200
C	-4.85439200	-2.31718300	2.86765400
H	-5.37026500	-3.27579200	2.73691700
H	-4.08579600	-2.41095200	3.64204400
H	-5.57388300	-1.55134300	3.15919900
C	3.42945100	6.58653800	-3.58129900
H	3.19462600	6.35344900	-4.62105300
H	3.06268500	7.59089800	-3.34012800
H	4.51423400	6.53897800	-3.43196600
C	5.11752600	8.46641800	0.76819200
H	5.88529300	8.04958500	0.10633100
H	4.45466700	9.12957200	0.20063100
H	5.59560000	9.02991600	1.57093400
C	-7.81301000	2.83167000	4.21519200
H	-8.76106500	3.37067200	4.18236800
H	-7.99988000	1.75571800	4.12067500
H	-7.30598400	3.04107600	5.16410500
C	-3.81045600	-0.00186100	5.34710000
H	-3.09617100	-0.72043000	5.75194300
H	-3.91081100	0.84472200	6.03625800
H	-4.78113800	-0.49075700	5.21536900

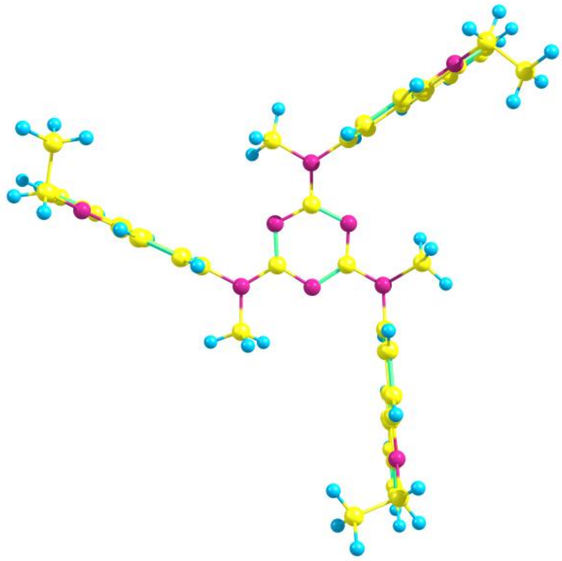

6-GS1

C	-0.88587400	0.96750900	-0.26920600
N	-1.80418000	1.97989800	-0.23868500
N	-1.35270500	-0.29970500	-0.27481200
C	-1.36633800	3.37780400	-0.31575500
C	-3.20676200	1.71821400	-0.42964400
N	0.41243900	1.31942600	-0.27926700
C	-0.39881100	-1.24846000	-0.27838000
H	-2.22387600	4.01517800	-0.09655400
H	-0.98315400	3.63221600	-1.31246800
H	-0.58303800	3.57795400	0.41820700
C	-4.05409900	1.63774900	0.67395200
C	-3.72109600	1.60675900	-1.73855600
C	1.27669200	0.28159700	-0.28378900
N	-0.81588100	-2.54997700	-0.25570800
N	0.93191900	-1.01886600	-0.29099600
C	-5.42439300	1.43299300	0.46455400
H	-3.64998800	1.72901000	1.67818000
C	-5.07991400	1.40396300	-1.97171900
H	-3.03766400	1.67587700	-2.58008500
N	2.61245200	0.57168400	-0.27122100
C	-2.24608400	-2.86967500	-0.32297700
C	0.11234900	-3.63370600	-0.44446000
C	-5.93048900	1.32063500	-0.86063100
C	-6.56074800	1.29728100	1.35111800

H	-5.45621000	1.31305700	-2.98552600
C	3.60441800	-0.50533100	-0.35836400
C	3.08446100	1.91898600	-0.45532100
H	-2.36608000	-3.93413800	-0.11768500
H	-2.66810800	-2.65136800	-1.31238200
H	-2.80474500	-2.30282600	0.42475600
C	0.60889800	-4.32242100	0.66074200
C	0.46436200	-4.02769000	-1.75254800
N	-7.30618200	1.14147700	-0.81365500
C	-6.71259400	1.31886400	2.74497700
C	-7.69998700	1.10808200	0.51839100
H	4.58812100	-0.07827500	-0.15904700
H	3.61649000	-0.96981200	-1.35280400
H	3.40075900	-1.28016400	0.38362300
C	3.45776900	2.68027100	0.65074700
C	3.22365900	2.43488300	-1.76105100
C	1.47090000	-5.40751900	0.45380900
H	0.32833700	-4.01538500	1.66429300
C	1.32046600	-5.10282400	-1.98332300
H	0.06068800	-3.47382500	-2.59530000
C	-8.17620000	0.91586300	-1.96643300
C	-7.98546100	1.14984700	3.28934900
H	-5.85034600	1.46439900	3.39035600
C	-8.97987300	0.93259000	1.06297700
C	3.96296400	3.97132900	0.44738500
H	3.35232300	2.27296400	1.65229400
C	3.72266300	3.71603400	-1.98809600
H	2.92777900	1.81759200	-2.60446100
C	1.81987300	-5.79373800	-0.87055900
C	2.15777500	-6.32098600	1.34263200
H	1.58572300	-5.38679500	-2.99661200
C	-8.30460700	-0.55862300	-2.35060100
H	-9.15749200	1.33707000	-1.73016700
H	-7.77798300	1.49610400	-2.80349400
C	-9.10379000	0.95834400	2.45255900
H	-8.11895100	1.16332500	4.36730400
H	-9.84950700	0.77971100	0.43189400
C	4.09502800	4.48159500	-0.87443200
C	4.43015700	5.01242000	1.33814200
H	3.81425700	4.09868200	-2.99944000
N	2.66360700	-6.89461700	-0.82082400
C	2.21727800	-6.45869600	2.73689400
C	2.89039700	-7.21543400	0.51186800
H	-8.72830200	-1.14615900	-1.52928400
H	-8.96697700	-0.65720800	-3.21789700
H	-7.33090700	-0.98466400	-2.61466600
H	-10.08617900	0.82513200	2.89754400
N	4.62733300	5.76263200	-0.82157100
C	4.55006200	5.11612000	2.73141900
C	4.82057200	6.10371500	0.51135900
C	3.29383600	-7.53776100	-1.97205700
C	3.00146700	-7.47430100	3.28341200
H	1.66064500	-5.78304400	3.38098900
C	3.68325500	-8.23422100	1.05840400
C	4.84485600	6.64291000	-1.96804400
C	5.04901200	6.29670000	3.28131700
H	4.25667100	4.28873600	3.37215600
C	5.31879600	7.29300900	1.06144800
C	4.63624200	-6.91425200	-2.35541200
H	3.41747900	-8.59821000	-1.73460500
H	2.59244300	-7.48165800	-2.80933700
C	3.72541500	-8.34963000	2.44836200
H	3.05864000	-7.59280300	4.36169900
H	4.25038600	-8.91211300	0.42847400
C	3.62119200	7.48721400	-2.32588200
H	5.69897400	7.28552400	-1.73629800
H	5.14111900	6.01819900	-2.81555300
C	5.42755100	7.37086500	2.45038700
H	5.14619800	6.39277300	4.35897200
H	5.60882900	8.13043500	0.43501000
H	5.35597200	-6.98786300	-1.53329600
H	5.05247000	-7.44001800	-3.22204200
H	4.51988900	-5.85803600	-2.62035400
H	4.33318800	-9.13184500	2.89514800
H	3.33074200	8.13577500	-1.49259900
H	3.85119100	8.12222200	-3.18877500
H	2.76637300	6.85419800	-2.58638500
H	5.81071600	8.28314500	2.89959800

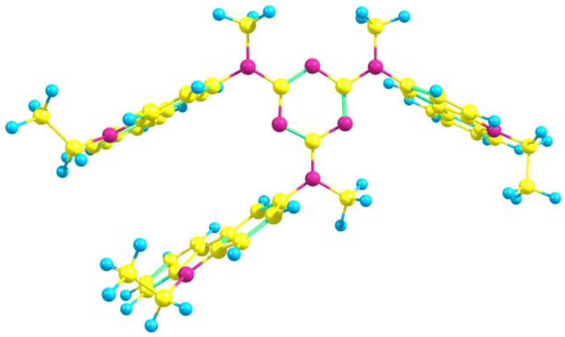

6-GS2

C	-1.08974100	0.14202900	-0.96327300
N	-0.86692700	-1.20006000	-0.81685600
N	-0.01325900	0.95213700	-1.01867400
C	-1.99131300	-2.14192000	-0.83520100
C	0.45067300	-1.75652800	-0.98087000
N	-2.37367200	0.54381000	-1.02594400
C	-0.30378400	2.26269600	-1.12994800
H	-1.62371500	-3.11599600	-0.50932600
H	-2.42349400	-2.24574600	-1.83917700
H	-2.77710000	-1.81650200	-0.15103200
C	1.22313900	-2.05812800	0.13903000
C	0.92047200	-2.05467000	-2.27723800
C	-2.54103300	1.87742700	-1.13651800
N	0.74006700	3.14282300	-1.20369800
N	-1.54641300	2.78523100	-1.18483800
C	2.47842500	-2.65409100	-0.04208600
H	0.85193700	-1.82900400	1.13389400

C	2.16374500	-2.64966900	-2.48236800
H	0.29640200	-1.80994500	-3.13204100
N	-3.81939500	2.35104200	-1.21689500
C	0.48958700	4.58597900	-1.28049900
C	2.08517500	2.72951700	-0.89958000
C	2.93946500	-2.95251800	-1.35492500
C	3.51859600	-3.08714300	0.86673200
H	2.51041500	-2.86564600	-3.48779000
C	-4.07548200	3.79310800	-1.29260900
C	-4.94935900	1.49278500	-0.97378600
H	1.42592400	5.08005200	-1.54408000
H	0.13541800	4.99102400	-0.32337700
H	-0.25325600	4.80811600	-2.04891400
C	2.99746400	2.51368700	-1.95164800
C	2.49457500	2.61439900	0.42910900
N	4.18776300	-3.55486000	-1.27793100
C	3.65918800	-3.06705700	2.26177500
C	4.55848000	-3.62890400	0.05903200
H	-5.12738600	3.93815800	-1.54246200
H	-3.86709800	4.29605100	-0.33948700
H	-3.46331600	4.25478300	-2.07029700
C	-5.62582600	0.91022400	-2.06433800
C	-5.40428500	1.29707200	0.33013600
C	4.32545800	2.16827600	-1.70428200
H	2.65221800	2.62102400	-2.97587300
C	3.82264600	2.26466300	0.70253900
C	5.00875800	-3.96581400	-2.41566900
C	4.82482400	-3.57850300	2.83162500
H	2.87151000	-2.65723000	2.88846300
C	5.73280400	-4.14041100	0.62890000
C	-6.75758100	0.11759500	-1.87928400
H	-5.24931000	1.08279100	-3.06837200
C	-6.53923300	0.50376900	0.54121700
C	4.73126200	2.03871600	-0.36952500
H	5.01504000	2.00918900	-2.52691000
C	4.57139100	2.06207400	1.92505500
C	5.93000200	-2.86054300	-2.93252400
H	5.59225800	-4.83789300	-2.10685100
H	4.33429700	-4.30313900	-3.20782300
C	5.84785000	-4.10820200	2.01910700
H	4.94897900	-3.56816700	3.91073000
H	6.53092400	-4.54669900	0.01568900
C	-7.21337700	-0.07871100	-0.56879800
H	-7.26139400	-0.32958200	-2.72998800
C	-7.26781700	0.09635600	1.72401300
N	5.97378500	1.69644500	0.14577800
C	5.89712200	1.72222500	1.53278300
H	6.62872700	-2.53228700	-2.15592600
H	6.51383500	-3.23459900	-3.78107700
H	5.35482500	-1.99219200	-3.27049800
H	6.74922700	-4.49886900	2.48360000
N	-8.31094800	-0.79665700	-0.11465100
C	-8.34824500	-0.71302600	1.27177700
C	7.18437400	1.46008000	-0.63847600
C	6.89574000	1.46743300	2.48302000
C	-9.21192100	-1.59204400	-0.94677400
C	-9.25819000	-1.28376200	2.17260100
C	7.99612200	2.72940500	-0.89963700
H	7.78905800	0.72177000	-0.10416800
H	6.88231700	0.99777500	-1.58227400
C	6.54617500	1.55110200	3.83133200
H	7.90938600	1.21630100	2.18739900
C	-8.73710600	-3.03102500	-1.15150000
H	-9.31364700	-1.08130700	-1.90856200
H	-10.19895700	-1.57782600	-0.47604000
C	-9.07527800	-1.02625900	3.53173600
H	-10.07825800	-1.90780300	1.83194000
H	7.40938400	3.46597800	-1.45868700
H	8.88589900	2.48279900	-1.48977600
H	8.32576100	3.18857500	0.03829200
C	5.23656200	1.88215900	4.23449600
H	7.30379800	1.35865500	4.58619800
H	-8.65460300	-3.55991500	-0.19612800
H	-9.45534100	-3.57031400	-1.77927700
H	-7.76161700	-3.05884900	-1.64836800
C	-8.01257900	-0.22344400	3.99368800
H	-9.76828900	-1.45784100	4.24890100
C	4.24586600	2.13852400	3.28680000
H	5.00013200	1.93921600	5.29320900
C	-7.10646600	0.33979100	3.09547000
H	-7.89975300	-0.04478400	5.05922700
H	3.23664000	2.39533100	3.59775600
H	-6.28639500	0.95744800	3.45205100
H	-4.87985200	1.75395900	1.16475100
H	1.78750500	2.79143700	1.23474800

Transition structure calculations

The geometries of all compounds have been fully optimized and the corresponding transition states were localized using B3LYP [2] functional with 6-31+G(d,p) basis set [3] or using M06-2X [4,5] functional with TZVP [6] basis set. All ground state structures were optimized without restrictions and using an ultrafine grid in the computation of two-electron integrals and their derivatives. Solvent effect was included implicitly to the optimizations via the SMD [7] model with the built in parameters for solvents (DMF or CH_3CN). The nature of all critical points was confirmed by means of the vibrational analysis.

The ΔH, ΔS and ΔG values were calculated at T = 298.15 K at the same level of theory including zero-point energy in the particular solvent environment (represented by relative permittivity) and vibrational, rotational and translational thermal energy corrections.

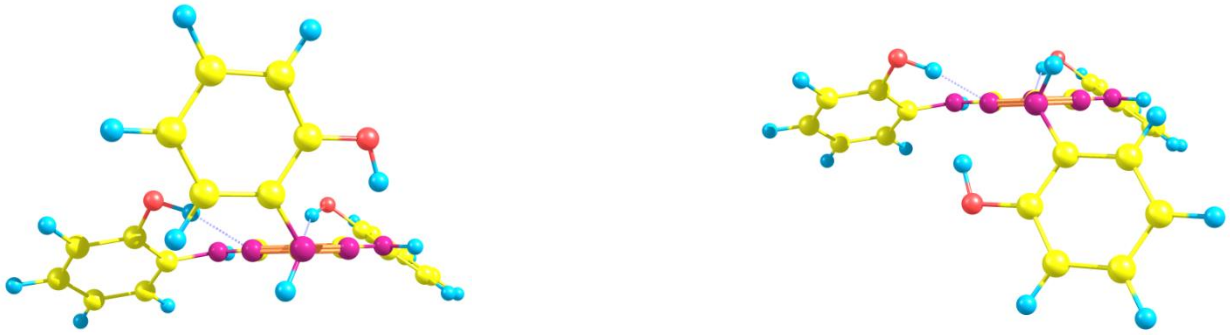

TS1 (syn, the oxygen atoms are on the same side) TS2 (anti)

Figure S25. The transition states (TS) structures of **11** regarding rotation around triazine-N bond.

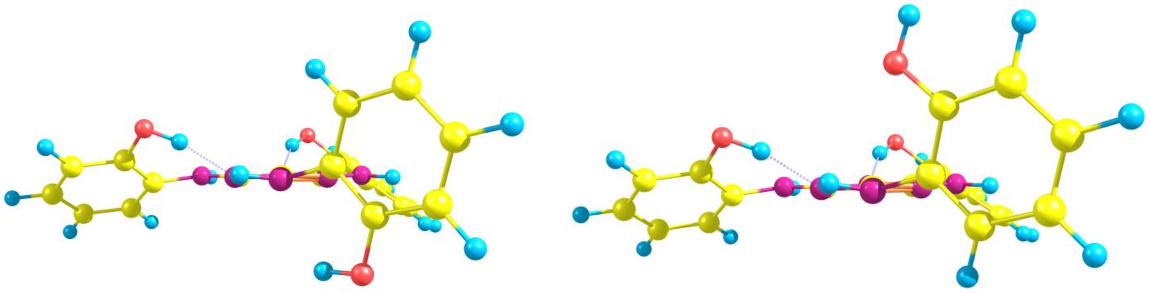

TS3 (anti) TS4 (syn, the oxygen atoms are on the same side)

Figure S26. The transition states (TS) structures of **11** regarding rotation around N-Ph bond.

Cartesian coordinates of the SMD(DMF)//M062X/TZVP optimized TS geometries for **11-14** and SMD(DMF)//B3LYP/6-31+G(d,p) optimized geometries for **4-6**:

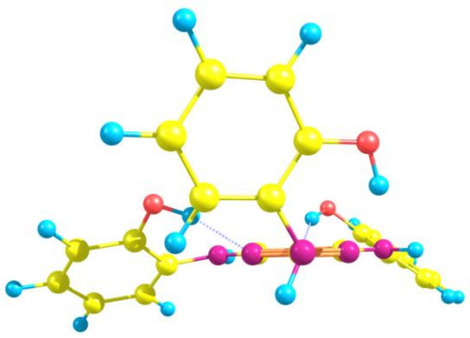

11-TS1 (syn)

C	0.15184100	0.83222000	-0.22414300
N	-0.13655300	2.06788800	0.21320300
N	-0.82459800	0.07515100	-0.74304400
C	-1.34359100	2.79386900	0.03851700
N	1.42575600	0.43017000	-0.09448800
C	-0.45152800	-1.14837500	-1.11787700
C	-1.23872900	4.11669300	-0.38896200
C	-2.60324500	2.27558100	0.36240700
C	1.68320700	-0.80250900	-0.52104700
N	-1.46729800	-2.01652200	-1.62502400
N	0.75781600	-1.64679300	-1.04307000
C	-2.36014800	4.92167800	-0.49702600
C	-3.72721700	3.08527200	0.23152400
N	2.93221900	-1.28764900	-0.44009700
C	-2.49534200	-2.28063500	-0.66538100
C	-3.61123500	4.40158000	-0.18379700
H	-4.69021900	2.65885500	0.48500000
C	4.12823600	-0.58446200	-0.14517000
C	-2.20865200	-3.18858600	0.35874700
C	-3.75309000	-1.69512800	-0.72875400
C	4.27137100	0.25677000	0.96444800
C	5.23033800	-0.82788100	-0.96434300
C	-3.16655900	-3.48116700	1.31910600
C	-4.72024700	-2.00025500	0.22062900
C	5.50402100	0.85349700	1.21241400
C	6.45836500	-0.24710500	-0.69807800
C	-4.41891600	-2.88530500	1.24906500
H	-2.91999600	-4.18459700	2.10500400
C	6.59458500	0.59839100	0.39787900
H	5.58659400	1.50402500	2.07459000
H	-1.87287000	-1.59609100	-2.45864400
H	3.05305700	-2.19517000	-0.87529700
H	0.66887400	2.61633100	0.49350100
H	-4.49724200	5.01834400	-0.26689400
H	-2.25640300	5.94781000	-0.82510800
H	7.54789800	1.06323800	0.61583500
H	7.30234600	-0.45301700	-1.34355400
H	-5.16296800	-3.12102100	1.99967900
H	-5.69818200	-1.54002100	0.16183800
O	3.26345100	0.47713700	1.85796800
H	2.42757100	0.59707900	1.35033900
O	-2.77753900	1.01615700	0.86015300
H	-2.21400600	0.40101900	0.33756900
O	-0.99153400	-3.79951500	0.44178800
H	-0.47004300	-3.61030400	-0.35584300
H	-3.96178700	-0.99348400	-1.52905000
H	-0.25551800	4.50572600	-0.62758000
H	5.10571200	-1.48950700	-1.81395400

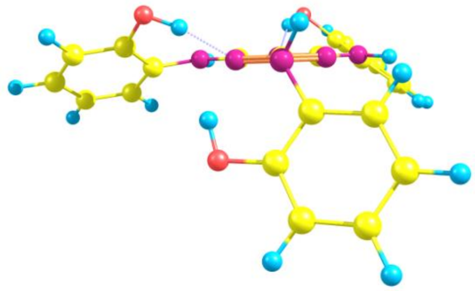

11-TS2 (anti)

C	-0.15075700	-1.10985400	-0.31475400
N	-0.47990600	-2.37462600	-0.00954200
N	-1.12063400	-0.24358900	-0.65062200
C	-1.77228900	-2.91233800	0.23001700
N	1.15116100	-0.79585600	-0.26809100
C	-0.70221300	0.98424000	-0.94957200
C	-1.91957100	-3.73550600	1.34594000
C	-2.85732500	-2.73287800	-0.63680200
C	1.44789400	0.46609100	-0.57265500
N	-1.73337700	1.90257000	-1.31822300
N	0.53863700	1.40649200	-0.92018500
C	-3.11853600	-4.37638000	1.60706900
C	-4.06469200	-3.36565600	-0.35557400
N	2.72830700	0.87027000	-0.55042800
C	-1.79143800	3.06890900	-0.49570000
C	-4.19677400	-4.19084500	0.74858900
H	-4.88911800	-3.20720400	-1.04009100
C	3.85913400	0.18254900	-0.04016100
C	-2.36835300	2.93225000	0.77049900
C	-1.34410600	4.31559000	-0.91179300
C	4.17264800	-1.13933600	-0.37855400
C	4.73829300	0.90750500	0.76405900
C	-2.46386100	4.02693900	1.61719200
C	-1.45679000	5.41861800	-0.07442900
C	5.34153000	-1.71056800	0.11540500
C	5.90931200	0.33815300	1.23393500
C	-2.00640800	5.26782900	1.19330200
H	-2.91196000	3.89443300	2.59440100
C	6.21175700	-0.97935500	0.90647700
H	5.55772700	-2.73625300	-0.15766800
H	-1.61651900	2.15997600	-2.29578700
H	2.85078400	1.86570600	-0.69857900

H	0.29306900	-2.93819900	0.32711600
H	-5.14175200	-4.68292700	0.94161700
H	-3.20879000	-5.01524200	2.47598700
H	7.12196400	-1.43910200	1.27040100
H	6.57981100	0.92013900	1.85285700
H	-2.08805000	6.12027200	1.85614300
H	-1.10825600	6.38733000	-0.40879300
O	3.40625000	-1.88550900	-1.22680200
H	2.46071300	-1.73100800	-0.99827300
O	-2.77271100	-2.00110100	-1.78541900
H	-2.26033600	-1.18142600	-1.58376200
O	-2.84142400	1.72556400	1.20000400
H	-2.88282900	1.10648600	0.45225600
H	-0.90551200	4.40990200	-1.89879500
H	-1.06881700	-3.86999700	2.00411700
H	4.48691700	1.93291200	1.00929400

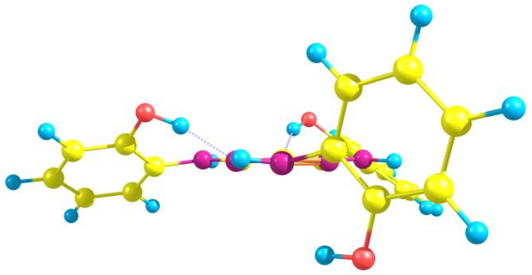

11-TS3 (anti)

C	1.28601800	-0.15027500	-0.11957200
N	2.62765300	-0.27866100	-0.16236300
N	0.52658600	-1.24165400	-0.07842800
C	3.40441200	-1.44171500	0.07315700
N	0.81519800	1.10836500	-0.13621200
C	-0.80271000	-1.01309000	-0.06400900
C	4.51657400	-1.30929900	0.90424100
C	3.16325400	-2.67005200	-0.55538600
C	-0.51533500	1.21224600	-0.10116600
N	-1.58380900	-2.11307700	-0.03793900
N	-1.37451600	0.18359300	-0.06033900
C	5.38187900	-2.36950200	1.11523800
C	4.02639800	-3.73662300	-0.32090100
N	-1.07040100	2.44241300	-0.11375800
C	-3.00275700	-2.08160900	-0.15722300
C	5.13461700	-3.59036900	0.49690800
H	3.81449100	-4.67646100	-0.81625600
C	-0.43345500	3.69497800	0.07262300
C	-3.77673800	-2.33167400	0.97638900
C	-3.62074100	-1.81076600	-1.37192900
C	0.70317700	4.09434200	-0.64216500
C	-1.04038000	4.59961300	0.94387500
C	-5.16472300	-2.32024600	0.88378600
C	-5.00350900	-1.79023800	-1.46544900
C	1.21526700	5.37421500	-0.45088900
C	-0.53685700	5.87852400	1.11237800
C	-5.77032000	-2.04895300	-0.33227700

H	-5.74901500	-2.52165100	1.77315200
C	0.59806900	6.26756500	0.40921100
H	2.09771900	5.65474400	-1.01316300
H	-1.10622400	-3.00553100	-0.09189700
H	-2.07304000	2.44025900	0.03183100
H	3.12919300	0.59850600	-0.08180400
H	5.80011000	-4.42965100	0.65537700
H	6.24202100	-2.24197900	1.75968400
H	1.00550700	7.26302000	0.53351200
H	-1.02780100	6.56520300	1.78983300
H	-6.85147100	-2.03948200	-0.39600600
H	-5.48051200	-1.57859400	-2.41348100
O	1.30844600	3.29401300	-1.56693500
H	1.32765600	2.37550200	-1.19849300
O	2.13821000	-2.85671600	-1.43564800
H	1.34610500	-2.38147500	-1.07624700
O	-3.21577900	-2.59224000	2.18774600
H	-2.24990400	-2.54630700	2.12126300
H	-2.99966000	-1.61590700	-2.23820600
H	4.69497800	-0.35077100	1.37834200
H	-1.92498300	4.28083400	1.48348500

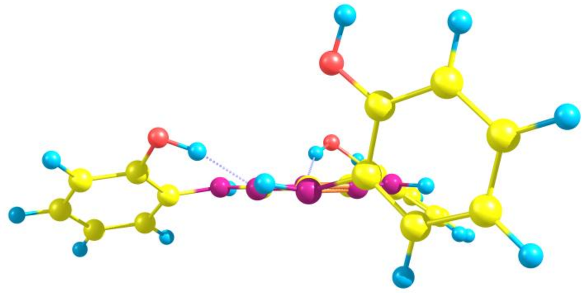

11-TS4 (syn)

C	-1.26269700	0.24320700	0.00375200
N	-2.57487400	0.52846700	0.13360100
N	-0.88415100	-1.01854500	-0.17453200
C	-3.68177700	-0.31949000	-0.12589300
N	-0.42321100	1.29014200	0.08064600
C	0.44974300	-1.21075700	-0.26391300
C	-4.73686700	0.21290200	-0.86651600
C	-3.80026900	-1.61298000	0.39876400
C	0.87145700	0.98286900	-0.03319500
N	0.84575500	-2.48368700	-0.44277700
N	1.36576300	-0.25035700	-0.20545300
C	-5.89378100	-0.51479800	-1.08880000
C	-4.95787300	-2.34549300	0.15231700
N	1.78132300	1.97838000	0.03540000
C	2.20801200	-2.89303900	-0.40572100
C	-6.00418500	-1.80208400	-0.57459000
H	-5.02111900	-3.34486500	0.56553600
C	1.55991100	3.37722200	-0.03070900
C	2.76147800	-3.30730100	0.80698800
C	2.98514900	-2.88578300	-1.55450700
C	0.62361800	4.04845700	0.76658300
C	2.39325000	4.11862500	-0.86838700

C	4.08821800	-3.72041700	0.85616500
C	4.31231000	-3.29030000	-1.50801900
C	0.53138700	5.43516700	0.69043300
C	2.30929600	5.49973800	-0.92257700
C	4.85649100	-3.70914500	-0.29922900
H	4.50550600	-4.04607000	1.80244400
C	1.37253600	6.16151000	-0.13637400
H	-0.20407700	5.92770400	1.31498900
H	0.12563900	-3.19071400	-0.35485000
H	2.72844400	1.68067100	-0.16655300
H	-2.78408600	1.52003700	0.15236600
H	-6.90091300	-2.38481700	-0.74394100
H	-6.70197100	-0.07852300	-1.66144700
H	1.29223200	7.24075800	-0.17050300
H	2.97093600	6.05371900	-1.57592000
H	5.88927000	-4.03164100	-0.25094500
H	4.91424500	-3.28258900	-2.40725500
O	-0.17779800	3.40119000	1.66065100
H	-0.48241100	2.56086800	1.23090500
O	-2.84240100	-2.17547500	1.19031600
H	-1.96073400	-1.93659800	0.80481300
O	1.95562300	-3.28365200	1.90277700
H	2.44195400	-3.59199600	2.68002700
H	2.53340700	-2.55780800	-2.48303700
H	-4.63536800	1.21786500	-1.26009400
H	3.11895500	3.58803700	-1.47410200

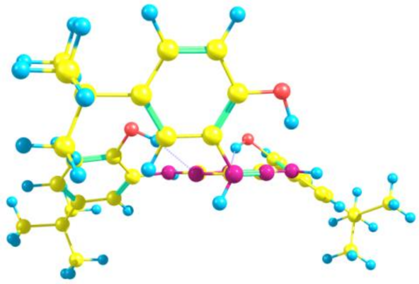

12-TS1 (syn)

C	0.49481700	0.25973900	-0.36716300
N	0.14532200	1.47976900	0.06827200
N	-0.45012600	-0.55498800	-0.85659800
C	-1.12383200	2.10603400	-0.03959700
N	1.79263300	-0.06896800	-0.26719800
C	-0.01662100	-1.75755300	-1.23643700
C	-1.17264700	3.41464100	-0.50331100
C	-2.31204900	1.50367300	0.39240300
C	2.10646300	-1.29350700	-0.68030900
N	-0.99139600	-2.67223000	-1.74316700
N	1.21823500	-2.19145700	-1.17673900
C	-2.36627800	4.12159400	-0.54144700
C	-3.50053600	2.21661800	0.34068300
N	3.38057600	-1.71161600	-0.61608300
C	-1.96642800	-3.02707300	-0.75155100
C	-3.55749200	3.53202800	-0.11875700
H	-4.39228900	1.70927200	0.69128900
C	4.54603800	-0.94253800	-0.36433400
C	-1.63476600	-4.03508400	0.14896900
C	-3.20903100	-2.40583700	-0.67297700
C	4.67752400	-0.06029700	0.71527200
C	5.64573600	-1.15105300	-1.18931800
C	-2.54740800	-4.39955200	1.13022100
C	-4.13974400	-2.75678700	0.30053300
C	5.88392100	0.59561100	0.91878900
C	6.85195500	-0.50603700	-0.96242700
C	-3.77730700	-3.76455600	1.20154200
H	-2.28280800	-5.18566500	1.82715400
C	6.99340900	0.38543600	0.10142000
H	5.93400400	1.27311400	1.76350800
H	-1.45922200	-2.23465600	-2.53433100
H	3.53879100	-2.61932200	-1.03873100
H	0.91960800	2.08150100	0.32526500
H	-2.35058900	5.13836200	-0.90871400
H	7.67711800	-0.70837200	-1.63090800
H	-4.46903700	-4.06752100	1.97883300
O	3.67800500	0.14662800	1.62335900
H	2.82724000	0.20439300	1.12795800
O	-2.34315000	0.24561400	0.92531000
H	-1.79555000	-0.33871400	0.35603000
O	-0.42890400	-4.67301400	0.09088500
H	0.05001600	-4.37839000	-0.70178300
H	-3.41901100	-1.62496700	-1.39394200
H	-0.25130100	3.88324000	-0.82976600
H	5.54629200	-1.84288700	-2.01833600
C	-4.90318000	4.25432100	-0.14696100
C	-5.87420300	3.47442900	-1.04440300
C	-5.47380100	4.32093700	1.27609400
C	-4.77784000	5.67795300	-0.68785300
H	-5.48968400	3.40593500	-2.06504200
H	-6.03852500	2.46071200	-0.67285600
H	-6.84090700	3.98316600	-1.07669500
H	-4.79737400	4.86352600	1.94098900
H	-6.43464000	4.84150900	1.26694100
H	-5.63484900	3.32407700	1.69098000
H	-5.76299500	6.14902600	-0.69109800
H	-4.11676600	6.28822400	-0.06825600
H	-4.39807900	5.68847200	-1.71203800
C	8.29614200	1.12673000	0.39819400
C	8.04344200	2.63950400	0.34502100
C	8.78683200	0.74645700	1.80179100
C	9.39278800	0.78243000	-0.60906600
H	7.68358200	2.93916300	-0.64232200
H	7.30520000	2.94930000	1.08695300
H	8.97212500	3.17854500	0.54841500
H	8.96894200	-0.32873400	1.87169800
H	9.72239600	1.26667500	2.02204400
H	8.06034500	1.02101500	2.56889500
H	10.29904600	1.33688600	-0.35665000
H	9.63629700	-0.28231700	-0.59267700
H	9.10576000	1.05529300	-1.62718100

C	-5.50004200	-2.07025200	0.42386400
C	-5.61370400	-1.41446400	1.80724200
C	-5.69225500	-0.98825200	-0.63903000
C	-6.61463100	-3.11299700	0.26763200
H	-5.53665200	-2.15200900	2.60850800
H	-4.82563600	-0.67095500	1.95030400
H	-6.57999600	-0.91254400	1.90367300
H	-5.66496700	-1.40468900	-1.64888300
H	-6.66562800	-0.51321300	-0.49850600
H	-4.92572800	-0.21234200	-0.56642700
H	-7.59213200	-2.63162500	0.35473800
H	-6.55823100	-3.59831400	-0.70986100
H	-6.55010900	-3.88534900	1.03661600

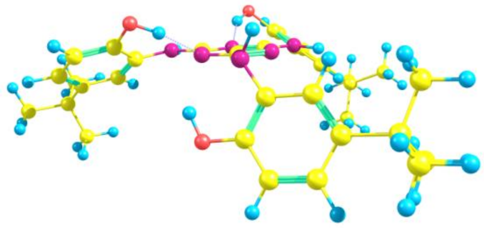

12-TS2 (anti)

C	-0.15800500	-1.14377100	-0.77536200
N	-0.51372200	-2.37536600	-0.38084200
N	-1.11064100	-0.28292700	-1.17127900
C	-1.81809300	-2.86746600	-0.10979100
N	1.15062900	-0.85490000	-0.75261500
C	-0.66705600	0.91136800	-1.55754100
C	-1.99351100	-3.60815700	1.05974900
C	-2.89914800	-2.73184700	-0.98137000
C	1.47292100	0.37561600	-1.14684700
N	-1.68074500	1.82322300	-1.98544900
N	0.58276400	1.30797900	-1.56053300
C	-3.20445100	-4.20117100	1.35692800
C	-4.12232600	-3.31957800	-0.66041400
N	2.76166300	0.75192800	-1.15539700
C	-1.70126300	3.05144800	-1.25726600
C	-4.30093800	-4.06762800	0.49547100
H	-4.92528500	-3.17503800	-1.37142100
C	3.87770400	0.07540900	-0.59899400
C	-2.24838600	3.03008500	0.02970800
C	-1.25218800	4.25651100	-1.76878400
C	4.16753100	-1.27266500	-0.84358300
C	4.77752800	0.82208800	0.15318900
C	-2.30945400	4.18664900	0.78619700
C	-1.33210200	5.42422500	-1.01490300
C	5.32375000	-1.83042600	-0.31612200
C	5.93966100	0.25826600	0.65776500
C	-1.85036400	5.40620000	0.27928700
H	-2.73853900	4.11857600	1.77923400
C	6.23516900	-1.08635300	0.43156200
H	5.50084500	-2.87860600	-0.52814700
H	-1.57503300	1.99844000	-2.98212300
H	2.90613900	1.73131200	-1.37372900
H	0.24756500	-2.93374300	-0.01072500
H	-3.29511600	-4.77245000	2.27255700
H	6.60746400	0.88483800	1.23243300
H	-0.97247700	6.34477400	-1.45317800
O	3.38221600	-2.06254000	-1.63562800
H	2.44157100	-1.86945800	-1.41801200
O	-2.80797800	-2.08226500	-2.17958200
H	-2.27440000	-1.26408400	-2.03768200
O	-2.72481700	1.86678200	0.56747700
H	-2.80472300	1.19449500	-0.12954900
H	-0.83393300	4.27738600	-2.76904800
H	-1.15049300	-3.71842800	1.73267700
H	4.55764800	1.86818700	0.33387900
C	-1.94702200	6.65720800	1.15264100
C	-3.41779100	6.91815900	1.50551900
C	-1.15020400	6.43657700	2.44565000
C	-1.39216800	7.89446700	0.44765400
H	-4.01232500	7.07246100	0.60169600
H	-3.85141800	6.08510700	2.06188700
H	-3.49821500	7.81518400	2.12471000
H	-0.09812600	6.23994900	2.22496700
H	-1.20649300	7.32874100	3.07439100
H	-1.54190900	5.59502900	3.02003700
H	-1.47925800	8.75598900	1.11301200
H	-0.33698700	7.77406400	0.19181200
H	-1.94532800	8.12082500	-0.46668500
C	7.49711400	-1.76278900	0.96492600
C	7.09925900	-2.92932700	1.87944300
C	8.31922400	-2.30264100	-0.21315800
C	8.37078400	-0.79633000	1.76373000
H	6.50376600	-2.57498400	2.72439800
H	6.51736700	-3.68021400	1.34172700
H	7.99545900	-3.41555600	2.27289700
H	8.61049000	-1.49306500	-0.88684300
H	9.22829600	-2.78284300	0.15743000
H	7.75858600	-3.04167000	-0.78855900
H	9.25637400	-1.32535400	2.12190900
H	8.70718100	0.04374400	1.15187200
H	7.84248300	-0.39993800	2.63381300
C	-5.63076000	-4.72988100	0.85077100
C	-6.13024500	-4.17153800	2.19025700
C	-6.70166800	-4.47835300	-0.20978800
C	-5.42066800	-6.24458900	0.98167300
H	-5.42265700	-4.37061200	2.99716500
H	-6.28350300	-3.09140600	2.12786100
H	-7.08291100	-4.63735300	2.45460700
H	-6.40702000	-4.87742900	-1.18307100
H	-7.62722900	-4.97468100	0.08920000
H	-6.91500100	-3.41304400	-0.32371400
H	-6.36703700	-6.73060900	1.23177400
H	-5.05625900	-6.66871000	0.04282100
H	-4.70156400	-6.48377500	1.76721400

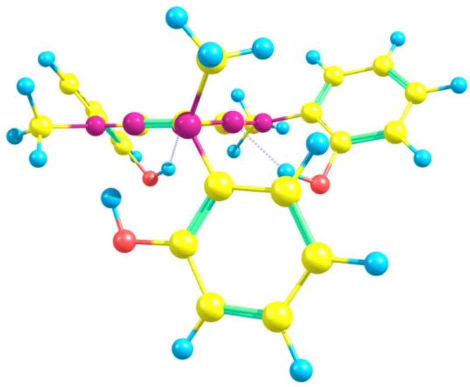

13-TS1 (syn)

C	-0.12941100	0.89320300	0.11252800
N	0.11697000	2.15045600	-0.30369400
N	0.89948200	0.16836800	0.59635400
C	1.35375100	2.81492500	-0.03103300
N	-1.37928100	0.42921900	0.00665400
C	0.59005000	-1.06241000	0.97330300
C	1.31572000	4.02777700	0.65387200
C	2.58345400	2.33091800	-0.49203100
C	-1.57809200	-0.81838500	0.44800900
N	1.65968400	-1.88851500	1.44714200
N	-0.60477400	-1.61363900	0.94992400
C	2.47153500	4.76165700	0.86841600
C	3.74129100	3.06722800	-0.26657000
N	-2.82124700	-1.33216700	0.38652100
C	2.58261100	-2.18835000	0.39421600
C	3.68873500	4.28130100	0.39923800
H	4.67813200	2.66765200	-0.63552000
C	-3.96931500	-0.52727600	0.10652400
C	2.18162200	-3.14332900	-0.55116300
C	3.83573500	-1.59832700	0.27068800
C	-4.11260400	0.17945200	-1.09242100
C	-5.01203000	-0.51531400	1.03081000
C	3.01856900	-3.48062300	-1.60503200
C	4.67939800	-1.94448400	-0.77838800
C	-5.28817600	0.87845500	-1.34423200
C	-6.18507600	0.17515600	0.77143100
C	4.26473700	-2.87893300	-1.71868500
H	2.68325100	-4.22262800	-2.31946000
C	-6.32463900	0.86965100	-0.42500600
H	-5.37089300	1.41586600	-2.28105200
H	4.59864900	4.84606300	0.55924700
H	2.41962700	5.70282400	1.40038900
H	-7.23721100	1.41147000	-0.63995100
H	-6.98468000	0.17026600	1.50091800
H	4.91411500	-3.14949800	-2.54201200
H	5.65268700	-1.47795200	-0.86141100
O	-3.14805700	0.17370500	-2.05979700
H	-2.28582600	0.30266200	-1.61501700
O	2.68994100	1.16543300	-1.19935600
H	2.18446100	0.48110100	-0.71254800
O	0.97160200	-3.76548700	-0.46237100
H	0.51019300	-3.48355200	0.34621900
H	4.15516400	-0.86235700	0.99800300
H	0.36194400	4.39527500	1.01303400
H	-4.89474200	-1.06253500	1.95848600
C	-0.99963300	2.96058500	-0.79996300
H	-1.52125100	2.42596000	-1.59144900
H	-1.70662200	3.19353300	-0.00200800
H	-0.59407400	3.88088400	-1.20968700
C	-3.05154000	-2.69715800	0.87257300
H	-2.31726300	-3.36866600	0.43351000
H	-2.97822900	-2.75120500	1.95967400
H	-4.04534000	-3.00268200	0.55811200
C	2.24586500	-1.35266200	2.67999000
H	1.46415200	-1.30285900	3.43794800
H	3.01972500	-2.03847500	3.02155200
H	2.67497900	-0.35475200	2.55245400

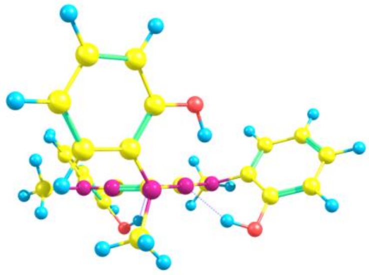

13-TS2 (anti)

C	-0.03464400	-1.12611700	0.29706500
N	0.12439900	-2.43753700	0.03266400
N	1.06668000	-0.39642200	0.58618600
C	1.40812600	-3.01758200	-0.21912800
N	-1.26976100	-0.62173400	0.26594500
C	0.83983400	0.88501400	0.82885400
C	1.60393000	-3.68854100	-1.42512000
C	2.44029500	-3.00099800	0.72586900
C	-1.37072600	0.69346900	0.50348400
N	1.99590400	1.65725900	1.17099300
N	-0.32572300	1.49583800	0.79223800
C	2.79230400	-4.35074400	-1.68797800
C	3.63377000	-3.66086500	0.45328100
N	-2.59358300	1.25794300	0.45396900
C	2.26744700	2.68571300	0.21753900
C	3.80933300	-4.34172800	-0.74039600
H	4.41396100	-3.63090300	1.20413600
C	-3.72623800	0.57472300	-0.08858100
C	2.80474900	2.28244000	-1.01329400
C	2.06334500	4.04010200	0.45434800
C	-4.21650500	-0.61445600	0.46158000
C	-4.40521500	1.15986100	-1.15540900
C	3.09900600	3.21921000	-1.99253300
C	2.37340300	4.98164400	-0.52077800
C	-5.37078100	-1.18999800	-0.05816600
C	-5.55959100	0.58702000	-1.66482000
C	2.88027000	4.56829900	-1.74592700

H	3.51319400	2.87728300	-2.93324700
C	-6.04678500	-0.59019100	-1.10844900
H	-5.72736600	-2.10998900	0.38877600
H	4.74210100	-4.85680800	-0.93314100
H	2.92213000	-4.86842400	-2.62960700
H	-6.94862800	-1.04669500	-1.49670900
H	-6.07359300	1.05816200	-2.49262900
H	3.11764000	5.29594200	-2.51199500
H	2.21142800	6.03337600	-0.32204000
O	-3.62327900	-1.21879400	1.53438700
H	-2.65796500	-1.21854400	1.37691100
O	2.30791000	-2.38617500	1.94000600
H	1.93507000	-1.49429300	1.77317800
O	3.05312500	0.96703600	-1.27868900
H	2.93123900	0.43783400	-0.47201200
H	1.65998600	4.36195800	1.40608500
H	0.80520800	-3.69290100	-2.15708100
H	-4.01933800	2.07860400	-1.58035300
C	1.95348300	2.07066600	2.57696700
H	2.87925500	2.59304000	2.81377000
H	1.10176800	2.71807200	2.80505500
H	1.89043900	1.17566500	3.19632400
C	-2.71888000	2.70131400	0.68229000
H	-2.19408200	2.97290700	1.59480900
H	-2.30540700	3.27432900	-0.14916500
H	-3.77400600	2.93321400	0.79655600
C	-1.06048500	-3.22956800	-0.31191200
H	-1.82576800	-3.09852000	0.45041100
H	-1.46915000	-2.93320300	-1.27948400
H	-0.77224600	-4.27620900	-0.34086300

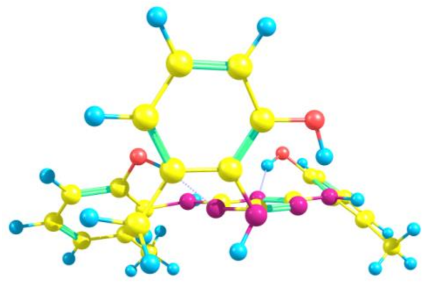

14-TS1 (syn)

C	-0.07810800	0.80956700	0.05962500
N	0.26742500	2.02880900	-0.38291400
N	0.86677900	0.03064600	0.60350800
C	1.50954500	2.68848400	-0.18191300
N	-1.35969000	0.44253500	-0.09351200
C	0.45481500	-1.17653000	0.98708600
C	1.48112400	3.99530100	0.32624600
C	2.72770200	2.10030600	-0.54238600
C	-1.65835100	-0.77743600	0.34653700
N	1.44077400	-2.06425900	1.51668900
N	-0.76761800	-1.64111900	0.89733200
C	2.67846900	4.68954300	0.46098400
C	3.91339000	2.80679900	-0.37859500
N	-2.91964500	-1.22616900	0.25045000
C	2.40731100	-2.45290300	0.52837100
C	3.88753700	4.10156100	0.11086900
H	4.84113900	2.32240500	-0.65759400
C	-4.08481100	-0.48997400	-0.08662500
C	2.02738500	-3.46898100	-0.35337900
C	3.68025400	-1.88056600	0.43966200
C	-4.16909900	0.27723600	-1.25332000
C	-5.20061600	-0.62266600	0.75359400
C	2.89325200	-3.90281400	-1.34714700
C	4.54371100	-2.33436600	-0.55635000
C	-5.35912800	0.92089800	-1.57294700
C	-6.38361200	0.01816300	0.40474000
C	4.15115200	-3.32787600	-1.44465100
H	2.57184000	-4.68793200	-2.02018200
C	-6.46529200	0.78296600	-0.75271000
H	-5.39482400	1.51191700	-2.47968000
H	1.89858300	-1.61498200	2.30608300
H	-3.06777600	-2.13444400	0.67512900
H	-0.50543000	2.59886500	-0.70810800
H	4.81302000	4.65160600	0.22792100
H	2.65765400	5.69957100	0.85213600
H	-7.39217400	1.28001100	-1.01087500
H	-7.24595200	-0.08324700	1.05257200
H	4.83230300	-3.66100600	-2.21804800
H	5.53084600	-1.89489600	-0.63559700
O	-3.12771500	0.37955900	-2.13295500
H	-2.30628700	0.52996900	-1.61410100
O	2.79745600	0.85601700	-1.10445200
H	2.22339100	0.25678200	-0.57843400
O	0.79868700	-4.05409100	-0.26063800
H	0.33802100	-3.72153300	0.52797800
C	4.11670400	-0.81174300	1.40385100
H	4.25463700	-1.22366300	2.40797300
H	5.06499600	-0.38103300	1.08426800
H	3.38131900	-0.00786300	1.47755900
C	0.17428300	4.63037500	0.70706600
H	-0.46247000	4.78855600	-0.16823400
H	-0.38181000	4.00183700	1.40595700
H	0.34707600	5.59898800	1.17337800
C	-5.11059500	-1.45261500	2.00232400
H	-4.94745800	-2.50892500	1.76935100
H	-4.28213200	-1.12634400	2.63491200
H	-6.03426600	-1.37599300	2.57353800

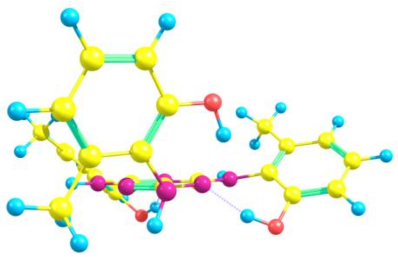

14-TS2 (anti)

C	-0.44222400	-1.10939600	-0.45018200
N	-0.99338200	-2.31396800	-0.23542600
N	-1.24370100	-0.05976500	-0.69685700
C	-2.36283300	-2.62000100	-0.01284800
N	0.89545700	-1.04052800	-0.41317300
C	-0.61357400	1.09401500	-0.90889600
C	-2.67472400	-3.42684700	1.09206000
C	-3.37107500	-2.21451300	-0.89477400
C	1.41066600	0.17050000	-0.61755000
N	-1.46753200	2.20630700	-1.18078300
N	0.68378400	1.28402300	-0.87080500
C	-3.99425900	-3.81910900	1.28610700
C	-4.68534600	-2.60755700	-0.66991900
N	2.74309200	0.33492900	-0.58521000
C	-1.35090500	3.27849700	-0.24122700
C	-4.99434600	-3.41612700	0.41001300
H	-5.44537600	-2.27516200	-1.36604600
C	3.72713400	-0.59270500	-0.15609100
C	-1.98738500	3.10238000	0.99066600
C	-0.68770100	4.47476600	-0.52622600
C	3.80642700	-1.88834200	-0.67806900
C	4.68942700	-0.14067100	0.75964300
C	-1.93386800	4.09079900	1.96262300
C	-0.65725300	5.46772200	0.45146800
C	4.83493900	-2.73345400	-0.27792100
C	5.71772200	-0.99876300	1.13141400
C	-1.26390800	5.27322500	1.68635600
H	-2.43035100	3.92534500	2.91072700
C	5.79386100	-2.28630000	0.61441900
H	4.86916700	-3.73254700	-0.69431900
H	-1.31212500	2.52577600	-2.13353700
H	3.04226300	1.29909500	-0.67376500
H	-0.32681700	-3.03807700	0.00844800
H	-6.01883400	-3.72570000	0.57533900
H	-4.23580700	-4.44240800	2.13854800
H	6.59828800	-2.94577600	0.91561100
H	6.46169400	-0.65064300	1.83775800
H	-1.22070800	6.05156700	2.43830000
H	-0.14502100	6.39856200	0.23912100
O	2.92949600	-2.34741800	-1.62149100
H	2.02346000	-2.08440800	-1.34698200
O	-3.10578000	-1.47456800	-2.01270700
H	-2.48710700	-0.75274200	-1.75490000
O	-2.67104000	1.95242400	1.26582600
H	-2.76328300	1.42658500	0.45362300
C	-0.03496600	4.68220800	-1.86340800
H	0.67702500	3.88421800	-2.08352000
H	-0.77613500	4.69162900	-2.66782600
H	0.49453700	5.63364800	-1.88426600
C	4.60750400	1.25175900	1.31723300
H	4.77033000	2.00492800	0.54063600
H	3.62710500	1.44418800	1.75835900
H	5.36688700	1.39530700	2.08415100
C	-1.59514500	-3.86615900	2.04011300
H	-0.89398700	-4.55297600	1.55687300
H	-1.01858200	-3.01411900	2.40649200
H	-2.03105400	-4.38233900	2.89391700

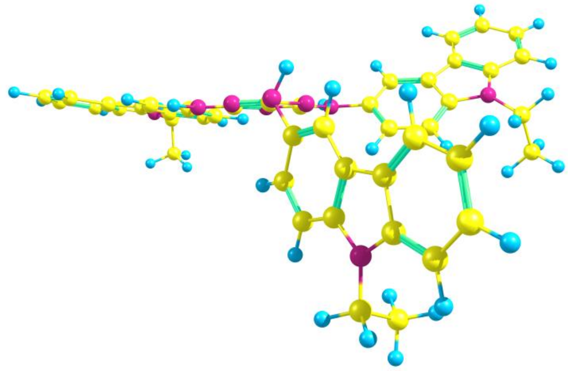

4-TS1 (syn)

C	0.05360000	-1.43656100	-1.34860400
N	-0.88363000	-2.35843300	-1.91616200
N	1.32183100	-1.81748800	-1.34645800
C	-1.88835400	-2.89552000	-1.06143000
N	-0.42779700	-0.27465800	-0.89147600
C	2.18894700	-0.89922000	-0.82024700
C	-3.18409000	-3.09127900	-1.54883800
C	-1.56263700	-3.30236700	0.25342700
C	0.50861200	0.55987000	-0.39497700
N	3.48464100	-1.30194300	-0.82719800
N	1.83181300	0.29212000	-0.33655200
C	-4.14185700	-3.70168000	-0.72647400
H	-3.43985000	-2.77346800	-2.55705900
C	-2.50740300	-3.89630200	1.08908300
H	-0.55321600	-3.15498700	0.62559600
N	0.14085300	1.77471300	0.08461700
C	4.68230900	-0.68191700	-0.39783400
C	-3.79897200	-4.10326200	0.59198000
C	-5.53571700	-4.04718200	-0.90912000
H	-2.23113300	-4.18981700	2.09677500
C	-1.15650800	2.35830800	0.12229600
C	4.72784800	0.51781500	0.34895000
C	5.87222000	-1.34406500	-0.73190300
N	-4.90676700	-4.68800200	1.20244500
C	-6.43782500	-3.90819700	-1.97391800
C	-5.96797100	-4.64398300	0.31139900
C	-2.27150200	1.64533000	0.61445600
C	-1.28731100	3.69160600	-0.27335800
C	5.94434300	1.06525100	0.75811300
H	3.80663700	1.01842700	0.60843200
C	7.09580100	-0.80700600	-0.32370300
C	-4.96735000	-5.15324000	2.58589200
C	-7.74940400	-4.35466400	-1.81440000
H	-6.11838000	-3.45687200	-2.90969800
C	-7.28790000	-5.08988200	0.47491700
C	-3.53021600	2.23713300	0.69005900
H	-2.14476100	0.62149600	0.94491600

C	-2.54139000	4.30792600	-0.18585600
C	7.12901300	0.40593800	0.41738900
H	5.95104000	1.98492500	1.33416500
C	8.47286000	-1.22275300	-0.49152900
C	-5.35215000	-4.05733600	3.58093300
H	-5.68462800	-5.97781000	2.62987700
H	-3.98939700	-5.57333200	2.83939700
C	-8.16471000	-4.93801600	-0.59959000
H	-8.45941100	-4.25168100	-2.63009200
H	-7.62426300	-5.53612800	1.40541400
C	-3.66331200	3.57099100	0.28308600
H	-4.37407700	1.67181700	1.07185000
C	-3.02426100	5.63567200	-0.50034100
N	8.44938600	0.74730900	0.68648900
C	9.27200800	-0.23781700	0.15710800
H	-6.34275000	-3.64857700	3.35495300
H	-5.37725500	-4.47142500	4.59525900
H	-4.62725000	-3.23659700	3.56580000
H	-9.19213300	-5.27621000	-0.49440700
N	-4.79195400	4.37937500	0.25074300
C	-4.41763000	5.63632200	-0.20592100
C	8.88503400	1.88462600	1.49387600
C	10.67001300	-0.34120000	0.18938500
C	-6.12221700	4.00569300	0.72697500
C	-5.20139200	6.78347700	-0.39284600
C	8.95701900	1.57609200	2.98995300
H	9.86304800	2.20236300	1.12152600
H	8.19233700	2.71019000	1.30616400
C	11.25583400	-1.43883600	-0.44210500
H	11.28222200	0.40260400	0.68936900
C	-6.33460000	4.28313800	2.21569500
H	-6.26607100	2.94313200	0.51102200
H	-6.85547700	4.55228900	0.12715500
C	-4.57249800	7.92651200	-0.88763300
H	-6.26162600	6.79045700	-0.16127700
H	7.97789200	1.27906200	3.38029500
H	9.28525500	2.46823000	3.53509700
H	9.67018200	0.77029600	3.19351700
C	10.47760100	-2.41857100	-1.09174700
H	12.33777200	-1.53964800	-0.42951700
H	-6.21855000	5.34874700	2.43999200
H	-7.34732500	3.98163500	2.50600000
H	-5.62241800	3.72041200	2.82843300
C	-3.19529200	7.93881500	-1.18913700
H	-5.15983800	8.82773700	-1.04154900
C	9.08693800	-2.31553300	-1.11945700
H	10.96751200	-3.26050300	-1.57244500
C	-2.41682700	6.79768900	-0.99742400
H	-2.73869300	8.84698500	-1.57217900
H	8.48694400	-3.07169000	-1.61892000
H	-1.35485000	6.80808400	-1.22818300
H	-0.42260900	4.23689400	-0.64219400
H	5.83813800	-2.26883900	-1.30288600
H	3.60835100	-2.21434600	-1.25170700
H	0.91770200	2.38646600	0.30837400
H	-1.29991300	-1.95217700	-2.75189100

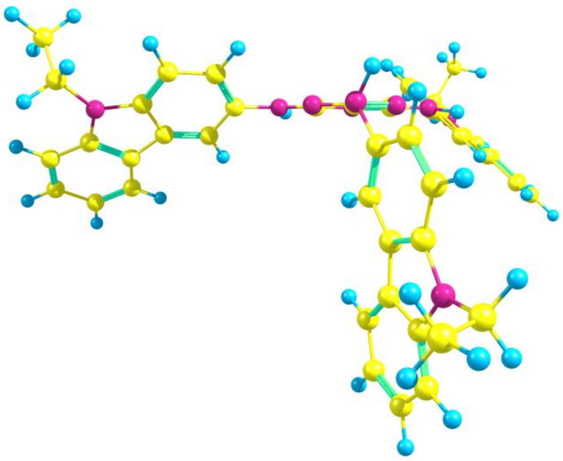

4-TS2 (anti)

C	-0.53773900	0.95643900	-2.15274000
N	-1.31903800	2.03326100	-2.68044300
N	0.78376600	1.09580300	-2.15659300
C	-1.27638700	3.28930500	-2.01505300
N	-1.22135300	-0.10263500	-1.71856000
C	1.46669700	0.03214800	-1.64869300
C	-1.20903200	3.37446600	-0.62090400
C	-1.37949900	4.46986400	-2.78755800
C	-0.44794300	-1.10435000	-1.23717100
N	2.81746300	0.16840000	-1.64593100
N	0.89814200	-1.08111400	-1.17574100
C	-1.23857000	4.63858700	-0.01321700
H	-1.14164500	2.47763900	-0.01194300
C	-1.42021300	5.73210500	-2.19859100
H	-1.43183200	4.38541600	-3.87033500
N	-1.03742600	-2.22922000	-0.76111500
C	3.80403100	-0.73570700	-1.16484400
C	-1.34283400	5.81365400	-0.80355300
C	-1.18929800	5.07739400	1.36560500
H	-1.50454800	6.61895300	-2.81849000
C	-2.42453400	-2.53957400	-0.68207700
C	3.75866900	-2.11319900	-1.47335500
C	4.87376400	-0.20999100	-0.43566500
N	-1.34031000	6.93354300	0.02586000
C	-1.08864500	4.40788400	2.59385000
C	-1.26629500	6.50078900	1.34048300
C	-3.27787200	-2.37668900	-1.79512200
C	-2.90900300	-3.08877800	0.50719700
C	4.75620300	-2.98333400	-1.03951900
H	2.93737500	-2.50016700	-2.06410500
C	5.89448800	-1.06763100	-0.00835600
C	-1.51749600	8.31557300	-0.41164100
C	-1.07152700	5.15255100	3.77303100
H	-1.02661100	3.32316800	2.62534500
C	-1.25409200	7.25129000	2.52550300

C	-4.62356900	-2.72972300	-1.73069700
H	-2.87651700	-1.97676700	-2.71873800
C	-4.25491900	-3.46756300	0.58334700
C	5.82387700	-2.45725800	-0.30071300
H	4.70175400	-4.03796800	-1.28895800
C	7.11563400	-0.86719100	0.74241000
C	-2.98343300	8.74066400	-0.51003000
H	-0.97914500	8.95913000	0.29013200
H	-1.02272200	8.42374900	-1.38134100
C	-1.15492100	6.55973200	3.73315200
H	-0.99541000	4.64688600	4.73145600
H	-1.32247400	8.33444900	2.51121400
C	-5.11147500	-3.27302400	-0.53493800
H	-5.26219800	-2.59765900	-2.59792600
C	-5.06842200	-4.05457900	1.62674400
N	6.92578600	-3.10056400	0.24846200
C	7.72439500	-2.14848600	0.86866600
H	-3.48357300	8.66368200	0.46123800
H	-3.04439400	9.78275800	-0.84360000
H	-3.52780500	8.12013400	-1.22972800
H	-1.14390800	7.12114900	4.66358900
N	-6.39424700	-3.69563500	-0.21201100
C	-6.37972300	-4.18620500	1.08722100
C	7.25254500	-4.51669300	0.09205700
C	8.94199800	-2.31287000	1.54393800
C	-7.53407400	-3.74009000	-1.12591400
C	-7.42680100	-4.73402400	1.84140100
C	8.06724700	-4.81631300	-1.16683100
H	7.79837600	-4.83230500	0.98559100
H	6.31289300	-5.07636700	0.08259400
C	9.53651700	-1.17852200	2.09763000
H	9.41315900	-3.28620900	1.63581200
C	-7.62402000	-5.04304000	-1.92117600
H	-7.45298800	-2.88385000	-1.80145800
H	-8.44067500	-3.59008300	-0.53290900
C	-7.14428400	-5.14052600	3.14589900
H	-8.42517700	-4.84328100	1.43006600
H	7.51908000	-4.52966900	-2.07051000
H	8.27855400	-5.89013200	-1.22221500
H	9.02242200	-4.28074600	-1.15650100
C	8.94297200	0.09531000	1.98452300
H	10.48044300	-1.28189000	2.62600600
H	-7.73131400	-5.90601000	-1.25558200
H	-8.49701700	-5.01170500	-2.58274800
H	-6.73260000	-5.19126900	-2.53982500
C	-5.85220100	-5.01151300	3.69516800
H	-7.94003000	-5.56818400	3.74984900
C	7.73367500	0.25683900	1.30895900
H	9.43460100	0.95686800	2.42723800
C	-4.81154600	-4.46949400	2.94137000
H	-5.66810700	-5.33919900	4.71435200
H	7.27705800	1.23911300	1.22063500
H	-3.81541800	-4.37130000	3.36475700
H	-2.24353400	-3.21976900	1.35625500
H	4.90827300	0.85254600	-0.20970600
H	3.13986800	1.09789100	-1.88983200
H	-0.40251100	-2.87126800	-0.30027500
H	-1.13524100	2.14002600	-3.67568200

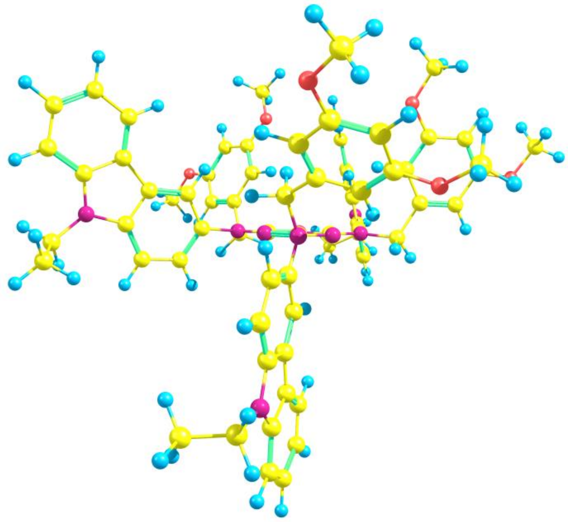

5-TS1 (syn)

C	1.31582900	-0.73860500	0.30329200
N	1.16588500	0.58287700	0.31640900
C	-0.04651900	1.00731700	0.76306300
N	0.41367400	-1.64699500	0.66010000
C	-0.77116300	-1.12600000	1.08246700
N	-1.03469300	0.18846700	1.15849800
N	2.57009700	-1.24096600	-0.17034900
N	-0.25907300	2.35210000	0.81735700
N	-1.75352200	-1.99719000	1.44755500
C	3.46893400	-1.11812700	2.10385800
C	6.10833200	-1.19200100	1.06966100
C	4.57183600	-1.08590000	2.96569300
C	3.67255700	-1.17903900	0.71463300
C	5.00144000	-1.21372800	0.21939900
C	5.89269500	-1.12948900	2.44815900
C	4.70050500	-1.01449200	4.40700400
C	5.63380900	-0.87994300	7.03618000
C	3.77795500	-0.94718700	5.46065100
C	6.09811900	-1.01919700	4.68911800
C	6.57352800	-0.94750400	6.00687100
C	4.24883500	-0.88039300	6.77213300
N	6.80684700	-1.10777100	3.50123100
C	-3.95571400	-1.13493100	0.78416200
C	-4.74065700	-1.08795800	3.52095900
C	-5.23768900	-0.70232500	1.14235100
C	-3.07181100	-1.52607800	1.78917900
C	-3.46523300	-1.50386000	3.14239800
C	-5.62666400	-0.68781000	2.51146800
C	-6.37548700	-0.22410200	0.38590300
C	-8.86010100	0.75131700	-0.42973400
C	-6.61304800	-0.01159600	-0.97965600

C	-7.39900500	0.05560500	1.33460000
C	-8.64849000	0.54919400	0.93468700
C	-7.85656500	0.47593000	-1.38083000
N	-6.93779700	-0.24449800	2.61083400
C	0.80777400	3.56859100	-1.03095700
C	2.51135000	4.87527800	0.84004400
C	1.75344800	4.50293000	-1.47126100
C	0.72578700	3.28514200	0.33144000
C	1.56831000	3.93720700	1.25470100
C	2.59771600	5.15674400	-0.53032100
C	2.11381200	5.01881700	-2.77482500
C	3.27970700	6.38391700	-4.91251900
C	1.65469600	4.77335500	-4.07681100
C	3.15957800	5.96019000	-2.55905400
C	3.75340000	6.64748300	-3.62678100
C	2.24161700	5.45783200	-5.14075500
N	3.42901000	6.04426400	-1.19822700
C	2.77775500	-1.08166500	-1.61300800
C	-1.47704900	2.92282900	1.41597600
C	-1.52897000	-3.45026500	1.51118900
C	4.48604100	6.84813300	-0.58657500
C	5.83083200	6.12452800	-0.50979600
C	-7.67333400	-0.02766300	3.85543500
C	-7.48688900	1.37530400	4.43414300
C	8.25950900	-1.05388300	3.36324200
C	8.80807900	0.36835500	3.23727400
H	2.46625900	-1.09763200	2.51806800
H	7.10899800	-1.21818100	0.64980900
H	5.18383600	-1.26307200	-0.84682000
H	5.98051800	-0.82322200	8.06458800
H	2.71018900	-0.94497800	5.25715700
H	7.63661500	-0.94034100	6.22597700
H	3.54527000	-0.82570900	7.59802300
H	-3.64507900	-1.15680600	-0.25548100
H	-5.02384400	-1.07033700	4.56828800
H	-2.75482300	-1.81280100	3.90375400
H	-9.82095200	1.13308100	-0.76421300
H	-5.83856200	-0.22062200	-1.71244800
H	-9.42860300	0.77114300	1.65577900
H	-8.05567100	0.64743000	-2.43470700
H	0.15405100	3.06206800	-1.73437300
H	3.15802100	5.36066700	1.56359900
H	1.48174100	3.69429600	2.30985000
H	3.72513700	6.90404700	-5.75623700
H	0.85531500	4.05845600	-4.25274800
H	4.55641800	7.35983200	-3.46715300
H	1.89850400	5.27699500	-6.15541200
H	3.45880000	-0.24650300	-1.82844300
H	1.81179200	-0.80442900	-2.04692700
H	-1.16853600	3.72921000	2.08703800
H	-1.94162800	2.14504600	2.02409100
H	-1.91717200	-3.80723400	2.46927200
H	-0.45131500	-3.61941000	1.50942700
H	4.14836100	7.13690900	0.41270900
H	4.57962500	7.77055600	-1.16665600
H	6.57597200	6.78024700	-0.04553700
H	6.19072300	5.85189000	-1.50747500
H	5.75578900	5.21309500	0.09256000
H	-7.34367400	-0.78478300	4.57236600
H	-8.73083100	-0.22208400	3.65485800
H	-8.06077700	1.46988100	5.36282100
H	-7.83946100	2.14149500	3.73571500
H	-6.43402900	1.57219600	4.66212700
H	8.69715000	-1.55670700	4.23058500
H	8.53198000	-1.64682400	2.48499100
H	9.89871700	0.33628700	3.13477300
H	8.56629100	0.96534300	4.12304800
H	8.39790500	0.87391500	2.35662500
C	-2.48480100	3.46353300	0.41397100
C	-2.99902200	4.75812900	0.60220800
C	-2.93100400	2.67776100	-0.64735700
C	-3.96718500	5.24386600	-0.28164900
H	-2.63769000	5.36068300	1.42695700
C	-3.90307500	3.18182400	-1.52636200
H	-2.54843500	1.67502800	-0.80604700
C	-4.42827900	4.46321200	-1.35347500
H	-5.17703100	4.87712700	-2.01776400
C	3.26858600	-2.33299400	-2.33007200
C	3.94512300	-2.18189100	-3.54536900
C	3.00926100	-3.61130300	-1.83177100
C	4.35012300	-3.31367800	-4.26166000
H	4.16384500	-1.19530900	-3.94443000
C	3.42688600	-4.73508700	-2.55564600
H	2.49248300	-3.74685100	-0.88801100
C	4.09983800	-4.60621000	-3.77821000
H	4.42127600	-5.47779800	-4.33010500
C	-2.17597100	-4.23909100	0.38413200
C	-2.87229400	-5.41196100	0.68908700
C	-2.04322800	-3.83494800	-0.94881200
C	-3.42594800	-6.18248600	-0.34190300
H	-2.99262500	-5.73838000	1.71800800
C	-2.60681000	-4.61013100	-1.96770900
H	-1.50919700	-2.92838000	-1.21315400
C	-3.30413300	-5.79344700	-1.68199400
H	-3.73887100	-6.38670600	-2.47375100
O	-4.08824200	-7.31705200	0.05594700
O	-2.42677900	-4.13339300	-3.24299000
O	3.13257500	-5.94901600	-1.98416000
O	5.00342200	-3.06565900	-5.44384700
O	-4.28681600	2.33329100	-2.53328700
O	-4.53248000	6.49169900	-0.18926600
C	-4.12574500	7.35202800	0.88343600
H	-3.05654100	7.58202100	0.82067700
H	-4.70379300	8.26875000	0.75806600
H	-4.35552400	6.90400600	1.85633200
C	-5.29085700	2.77304000	-3.45706500
H	-6.22925300	3.00221600	-2.94124500
H	-4.95020100	3.65057100	-4.01795800

H	-5.44397600	1.93927900	-4.14366800
C	3.51635900	-7.14847300	-2.66883200
H	3.17762100	-7.96874200	-2.03421600
H	3.02809400	-7.21757800	-3.64715400
H	4.60389200	-7.20433800	-2.78875400
C	5.45428500	-4.17410900	-6.23342500
H	6.18245700	-4.78035000	-5.68356100
H	4.61302700	-4.79854900	-6.55366800
H	5.93423600	-3.73346100	-7.10848800
C	-4.68170400	-8.15778100	-0.94238200
H	-5.13931500	-8.98318100	-0.39511000
H	-5.45300200	-7.61951100	-1.50403000
H	-3.92346900	-8.54995400	-1.62894200
C	-2.95141600	-4.88540000	-4.34485600
H	-2.67843500	-4.32296900	-5.23899000
H	-2.50313600	-5.88398300	-4.38996000
H	-4.04219600	-4.96698800	-4.28376000

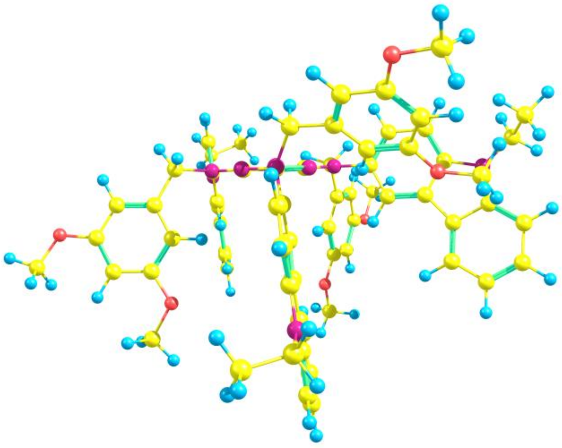

5-TS2 (anti)

C	0.69079600	1.15823000	-1.92501000
N	0.97907100	-0.09424900	-1.58065600

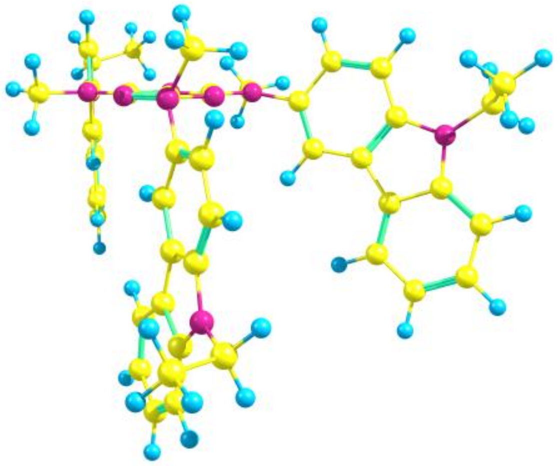

6-TS1 (syn)

C	0.71436900	1.15408300	-1.36724500
N	0.65117600	2.34106400	-0.70792300
N	-0.43076300	0.67830500	-1.92892600
C	1.85552900	2.90513000	-0.08688700
C	-0.61063100	2.96875800	-0.41138900
N	1.90522400	0.53580200	-1.43106200
C	-0.29141300	-0.47584000	-2.57184600
H	1.63241700	3.92694200	0.22126900
H	2.68484600	2.92237800	-0.79724400
H	2.16058100	2.32888200	0.79531900
C	-1.40876300	2.47613900	0.62055300
C	-0.99688100	4.11384900	-1.13601300
C	1.91787400	-0.62580500	-2.11091300
N	-1.46017900	-1.00971500	-3.20888900
N	0.82831800	-1.18250500	-2.70839200
C	-2.61226400	3.12803700	0.91484800
H	-1.10062800	1.59556500	1.17682900
C	-2.18602100	4.78466300	-0.85418700
H	-0.35199400	4.47458000	-1.93210500
N	3.08910500	-1.30183300	-2.21896200
C	-1.97726700	-0.14869400	-4.27867800
C	-2.39157200	-1.66996700	-2.36500100
C	-2.99218600	4.28503700	0.17743200
C	-3.66231600	2.89464900	1.88351100
H	-2.47029800	5.66175000	-1.42638600
C	3.15257500	-2.57242700	-2.95436300
C	4.29464800	-0.81660600	-1.59826500
H	-2.53028000	-0.74885500	-5.00616400
H	-1.13162400	0.30239000	-4.80189100
H	-2.63133700	0.65443400	-3.90994700
C	-3.76389800	-1.73644600	-2.71661800
C	-1.96236400	-2.30549800	-1.18724100
N	-4.20161400	4.76175500	0.66301200
C	-3.86663100	1.92942700	2.87990700
C	-4.62740500	3.92191200	1.68489900
H	4.19099900	-2.90314400	-2.96454600
H	2.54205300	-3.34348300	-2.47262600
H	2.81142800	-2.44980600	-3.98637300
C	5.18862600	-0.02841800	-2.35058000
C	4.59138700	-1.17574200	-0.28449000
C	-4.69632700	-2.41887800	-1.93250500
H	-4.11832900	-1.25297700	-3.61858000
C	-2.88937200	-2.98641700	-0.38933900
C	-4.95497900	5.88917100	0.11683400
C	-5.02229600	1.99504600	3.65778200
H	-3.13556000	1.14168400	3.04116200
C	-5.79186800	3.98810900	2.46254700
C	6.38961200	0.42421300	-1.80731800
H	4.92919100	0.23073900	-3.37290700
C	5.79122000	-0.72906000	0.28354400
C	-4.25857500	-3.03988200	-0.76085700
H	-5.73691100	-2.45022000	-2.23993900
C	-2.77460000	-3.72498100	0.85203700
C	-5.92021200	5.48982900	-0.99978000

H	-5.49683600	6.35880800	0.94270900
H	-4.23327800	6.62592700	-0.24657800
C	-5.97192500	3.01535400	3.44659900
H	-5.19537100	1.25368200	4.43261100
H	-6.53422800	4.76464200	2.30858600
C	6.68654500	0.06715800	-0.48517400
H	7.06474300	1.03489000	-2.39778800
C	6.40473400	-0.89758300	1.58386200
N	-4.97441000	-3.75389700	0.19939400
C	-4.08510500	-4.18792300	1.17006400
H	-6.66502600	4.77157600	-0.64122200
H	-6.44972100	6.37741300	-1.36388400
H	-5.38475900	5.04173800	-1.84343900
H	-6.86717600	3.04590000	4.06176000
N	7.80420600	0.36927500	0.27905200
C	7.64143700	-0.19390400	1.53868000
C	-6.39326300	-4.09079900	0.12476500
C	-4.32605300	-4.95338600	2.32064100
C	8.91508500	1.22314800	-0.13738200
C	8.49439400	-0.14593600	2.65002500
C	-6.67306100	-5.38806600	-0.63576500
H	-6.77428100	-4.15853300	1.14788900
H	-6.91060900	-3.25126400	-0.34910600
C	-3.24173400	-5.24271200	3.14992700
H	-5.32027900	-5.31574400	2.56241400
C	8.68819000	2.70477600	0.16456500
H	9.06801600	1.06996200	-1.20937400
H	9.81737700	0.86316600	0.36508100
C	8.09542300	-0.82054600	3.80437700
H	9.43361400	0.39713800	2.62104900
H	-6.31937000	-5.32457000	-1.67031000
H	-7.75191700	-5.57966100	-0.65547000
H	-6.18393800	-6.24163100	-0.15445600
C	-1.94133900	-4.78796200	2.84998300
H	-3.40619200	-5.83453500	4.04647000
H	8.55665500	2.87415300	1.23846000
H	9.55548200	3.28560800	-0.16886600
H	7.80289400	3.08356800	-0.35700500
C	6.87607100	-1.52571600	3.86219400
H	8.74034900	-0.79832100	4.67869400
C	-1.70309400	-4.02994200	1.70329100
H	-1.11997700	-5.03297600	3.51736700
C	6.02726400	-1.56767300	2.75643600
H	6.59706300	-2.03919400	4.77788200
H	-0.70007100	-3.68129400	1.47115900
H	5.08678000	-2.11034100	2.80180500
H	3.89693800	-1.78735500	0.28437600
H	-0.91831000	-2.28144700	-0.89263300

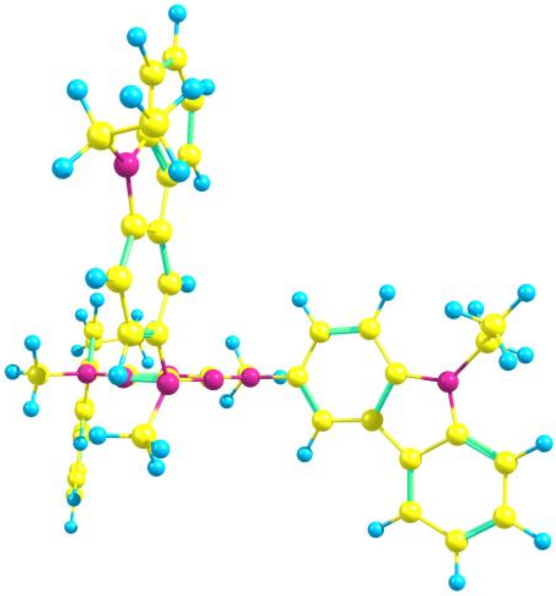

6-TS2 (anti)

C	0.47777600	-0.68592200	-0.23358600
N	0.51789200	-1.78660000	0.55954300
N	-0.67798600	-0.43801000	-0.91593100
C	1.72757700	-2.09261600	1.33325800
C	-0.63788000	-2.62490400	0.74950000
N	1.58039000	0.07722900	-0.29878100
C	-0.64073800	0.65074100	-1.67299900
H	1.57043800	-3.04133900	1.84627500
H	1.93252500	-1.31601400	2.07855000
H	2.59851000	-2.18471300	0.67794600
C	-0.81551700	-3.75184800	-0.05097800
C	-1.54363100	-2.32803200	1.78809200
C	1.49338300	1.15161900	-1.10656300
N	-1.82426200	0.93942900	-2.42981800
N	0.38743200	1.48627300	-1.82125800
C	-1.91794300	-4.58281900	0.18631300
H	-0.10893300	-3.97315300	-0.84572600
C	-2.64656300	-3.14076200	2.04305800
H	-1.37576300	-1.44386000	2.39602000
N	2.57348200	1.96508400	-1.22363600
C	-1.62402100	0.81645000	-3.87821100
C	-2.64198100	1.99354000	-1.94722800
C	-2.82624800	-4.27378500	1.23796800
C	-2.40386300	-5.79781000	-0.43234700
H	-3.33810300	-2.89356300	2.84177100
C	2.52763700	3.13888200	-2.10663900
C	3.79727900	1.69747900	-0.51339600
H	-2.57432700	0.59076400	-4.36951200
H	-0.95044000	-0.02222100	-4.06663000
H	-1.19493900	1.72093600	-4.33326700
C	-3.47695600	2.72286600	-2.83147600
C	-2.67414700	2.30072500	-0.57616000
N	-3.81555000	-5.24509700	1.29036900
C	-1.95581800	-6.59055100	-1.49862000
C	-3.58290300	-6.16544200	0.27568100

H	3.48456800	3.65469900	-2.02976800
H	2.36585000	2.84793300	-3.14953500
H	1.73140500	3.82755300	-1.80813400
C	3.95371100	2.16981400	0.80543400
C	4.83544400	1.02682800	-1.15804400
C	-4.33495400	3.72749500	-2.37919100
H	-3.46616200	2.50384500	-3.89202600
C	-3.52311200	3.31237400	-0.11224400
C	-4.96175200	-5.24746500	2.19778700
C	-2.68298300	-7.72824700	-1.84748300
H	-1.05605100	-6.32055300	-2.04512100
C	-4.31837600	-7.30606600	-0.07554500
C	5.13935900	1.97214400	1.51041600
H	3.12791300	2.69243900	1.27900000
C	6.03808000	0.82055500	-0.47018100
C	-4.35282700	4.02758300	-1.01551100
H	-4.96288000	4.25769400	-3.08832300
C	-3.78481300	3.86065600	1.20329500
C	-6.17212400	-4.49148300	1.64885300
H	-5.22147000	-6.29011700	2.40191100
H	-4.63613500	-4.81258300	3.14692400
C	-3.85159800	-8.07734900	-1.14045800
H	-2.34943700	-8.35152200	-2.67226200
H	-5.22253000	-7.58463200	0.45638100
C	6.18344000	1.29710500	0.86322300
H	5.24015300	2.33525500	2.52799900
C	7.28925800	0.18081600	-0.81686600
N	-5.08462400	4.98651600	-0.31610600
C	-4.76246100	4.88350400	1.02804600
H	-6.52330300	-4.93468400	0.71105000
H	-6.99241200	-4.53309900	2.37421000
H	-5.93141000	-3.43895100	1.46596200
H	-4.40510600	-8.96620600	-1.43101200
N	7.45559700	0.98954800	1.32443700
C	8.13101700	0.30016300	0.32489700
C	-6.10563400	5.85944300	-0.88809300
C	-5.24892000	5.61876400	2.11910300
C	7.96118200	1.24588600	2.67203200
C	9.43003500	-0.22649400	0.33146200
C	-7.49998300	5.23114900	-0.91488900
H	-6.11581600	6.78666500	-0.30774700
H	-5.78742600	6.12474900	-1.90069500
C	-4.73773400	5.32389800	3.38355700
H	-6.00026100	6.39171700	1.99177100
C	7.67206700	0.11036200	3.65435400
H	7.51361200	2.17966000	3.02358800
H	9.03794400	1.42154400	2.59498700
C	9.87690200	-0.86662800	-0.82504200
H	10.07144000	-0.14526400	1.20309500
H	-7.50982900	4.31558500	-1.51575900
H	-8.21465300	5.93570000	-1.35518000
H	-7.84225100	4.98543000	0.09601900
C	-3.76629900	4.31934400	3.57087400
H	-5.10031200	5.88175700	4.24286700
H	8.13694200	-0.82513700	3.32584600
H	8.07654100	0.36455800	4.64052100
H	6.59487200	-0.05631900	3.76001900
C	9.05648400	-0.98766200	-1.96510400
H	10.88065000	-1.28249900	-0.84402600
C	-3.28799300	3.58534600	2.48516100
H	-3.39083400	4.11582200	4.56971200
C	7.76283700	-0.46670700	-1.96695500
H	9.43731100	-1.49308700	-2.84798900
H	-2.54057000	2.80956400	2.63002900
H	7.12994600	-0.56137100	-2.84533700
H	4.70619400	0.67195400	-2.17663000
H	-2.05421900	1.75681700	0.12917200

Table S23. Theoretical thermodynamic parameters of restricted rotation around triazine-N bond

Comp.	Process	Via	Method	$\Delta H^{\neq}$ (298 K) kcal mol^{-1}	$\Delta S^{\neq}$ (298 K) cal K^{-1} mol^{-1}	$\Delta G^{\neq}$ (298 K) kcal mol^{-1}	$\Delta G^{\neq}_{eff}$ (298 K) kcal mol^{-1}
**11**	***GS1->GS2***	Anti-TS	SMD(DMF)//B3LYP/6-31+G(d,p)	15.5	-2.6	15.9	15.9
		Syn-TS	SMD(DMF)//B3LYP/6-31+G(d,p)	15.4	-3.0	16.3	
		Anti-TS	SMD(DMF)//M062X/TZVP	14.6	-1.4	15.0	14.4
		Syn-TS	SMD(DMF)//M062X/TZVP	13.4	-4.3	14.7	
	Exp. S to A			13.1 ± 1.3	2.2 ± 4.4		12.4 ± 0.1
**11**	***GS2->GS1***	Anti-TS	SMD(DMF)//B3LYP/6-31+G(d,p)	13.4	-4.5	14.8	14.4
		Syn-TS	SMD(DMF)//B3LYP/6-31+G(d,p)	13.3	-4.9	14.8	
		Anti-TS	SMD(DMF)//M062X/TZVP	14.4	-3.4	15.4	14.8
		Syn-TS	SMD(DMF)//M062X/TZVP	13.2	-6.2	15.1	
	Exp. A to S			12.9 ± 1.2	0.8 ± 4.2		12.7 ± 0.1
**12**	***GS1->GS2***	Anti-TS	SMD(DMF)//B3LYP/6-31+G(d,p)	14.3	-2.0	14.8	
		Syn-TS	SMD(DMF)//B3LYP/6-31+G(d,p)	14.7	-3.2	15.6	14.7
		Anti-TS	SMD(DMF)//M062X/TZVP	13.7	-5.5	15.3	
		Syn-TS	SMD(DMF)//M062X/TZVP	11.2	-10.5	14.3	14.2
	Exp. S to A			14.9 ± 0.8	6.4 ± 3.2		13.0 ± 0.1
**12**	***GS2->GS1***	Anti-TS	SMD(DMF)//B3LYP/6-31+G(d,p)	13.2	-6.4	15.1	
		Syn-TS	SMD(DMF)//B3LYP/6-31+G(d,p)	13.7	-7.6	15.9	15.0
		Anti-TS	SMD(DMF)//M062X/TZVP	15.1	-3.2	16.0	
		Syn-TS	SMD(DMF)//M062X/TZVP	12.5	-8.2	15.0	14.6
	Exp. A to S			15.3 ± 0.9	7.6 ± 3.6		13.0 ± 0.1
**13**	***GS1->GS2***	Anti-TS	SMD(DMF)//B3LYP/6-31+G(d,p)	12.2	-4.9	13.7	
		Syn-TS	SMD(DMF)//B3LYP/6-31+G(d,p)	12.1	-6.6	14.1	13.5
		Anti-TS	SMD(DMF)//M062X/TZVP	13.6	-7.4	15.8	
		Syn-TS	SMD(DMF)//M062X/TZVP	12.2	-6.9	14.3	14.2
	Exp. S to A			13.0 ± 1.5	-3.7 ± 4.8		14.1 ± 0.1
**13**	***GS2->GS1***	Anti-TS	SMD(DMF)//B3LYP/6-31+G(d,p)	12.8	-4.7	14.2	
		Syn-TS	SMD(DMF)//B3LYP/6-31+G(d,p)	12.7	-6.4	14.6	13.9
		Anti-TS	SMD(DMF)//M062X/TZVP	15.8	-1.8	16.3	
		Syn-TS	SMD(DMF)//M062X/TZVP	14.4	-1.4	14.8	14.7
	Exp. A to S			15.4 ± 1.1	5.3 ± 6.1		13.8 ± 0.1
**14**	***GS1->GS2***	Anti-TS	SMD(DMF)//B3LYP/6-31+G(d,p)	15.7	-5.7	17.4	
		Syn-TS	SMD(DMF)//B3LYP/6-31+G(d,p)	15.4	-6.4	17.3	16.9
		Anti-TS	SMD(DMF)//M062X/TZVP	14.4	-1.9	14.9	
		Syn-TS	SMD(DMF)//M062X/TZVP	12.1	-4.6	13.5	13.5
	Exp. S to A			15.5 ± 1.1	5.2 ± 5.3		13.9 ± 0.1
**14**	***GS2->GS1***	Anti-TS	SMD(DMF)//B3LYP/6-31+G(d,p)	14.2	-6.5	16.1	
		Syn-TS	SMD(DMF)//B3LYP/6-31+G(d,p)	13.9	-7.2	16.0	15.7
		Anti-TS	SMD(DMF)//M062X/TZVP	15.8	-0.3	15.9	
		Syn-TS	SMD(DMF)//M062X/TZVP	13.6	-3.0	14.5	14.4
	Exp. A to S			14.7 ± 1.2	1.7 ± 3.7		14.2 ± 0.1
**4**	***GS1->GS2***	Anti-TS	SMD(DMF)//B3LYP/6-31+G(d,p)	11.2	-7.5	13.5	
		Syn-TS	SMD(DMF)//B3LYP/6-31+G(d,p)	11.4	-7.6	13.7	13.2
		Anti-TS	SMD(DMF)//M062X/TZVP	11.0	-6.9	13.0	
		Syn-TS	SMD(DMF)//M062X/TZVP	11.0	-7.2	12.8	12.5
	Exp. S to A			14.4 ± 0.8	1.8 ± 3.0		13.9 ± 0.1
**4**	***GS2->GS1***	Anti-TS	SMD(DMF)//B3LYP/6-31+G(d,p)	10.8	-4.0	12.0	
		Syn-TS	SMD(DMF)//B3LYP/6-31+G(d,p)	11.0	-4.1	12.2	11.7
		Anti-TS	SMD(DMF)//M062X/TZVP	16.1	3.6	15.0	
		Syn-TS	SMD(DMF)//M062X/TZVP	15.8	3.2	14.9	14.5
	Exp. A to S			15.6 ± 0.7	6.6 ± 3.1		13.6 ± 0.1
**5**	***GS1->GS2***	Anti-TS	SMD(DMF)//B3LYP/6-31+G(d,p)	14.1	-11.2	17.5	14.1
		Syn-TS	SMD(DMF)//B3LYP/6-31+G(d,p)	11.6	-8.2	14.1	
		Anti-TS	SMD(DMF)//M062X/TZVP				
		Syn-TS	SMD(DMF)//M062X/TZVP				
	Exp. S to A			14.8 ± 1.0	1.7 ± 3.7		14.3 ± 0.1
**5**	***GS2->GS1***	Anti-TS	SMD(DMF)//B3LYP/6-31+G(d,p)	12.9	-11.2	16.3	12.9
		Syn-TS	SMD(DMF)//B3LYP/6-31+G(d,p)	10.4	-8.2	12.9	
		Anti-TS	SMD(DMF)//M062X/TZVP				
		Syn-TS	SMD(DMF)//M062X/TZVP				
	Exp. A to S			16.5 ± 1.0	6.1 ± 4.4		14.7 ± 0.1
**6**	***GS1->GS2***	Anti-TS	SMD(DMF)//B3LYP/6-31+G(d,p)	10.5	-3.9	11.7	
		Syn-TS	SMD(DMF)//B3LYP/6-31+G(d,p)	10.5	-3.2	11.4	11.1
		Anti-TS	SMD(DMF)//M062X/TZVP				
		Syn-TS	SMD(DMF)//M062X/TZVP				
	Exp. S to A			14.6 ± 0.9	9.8 ± 4.2		11.7 ± 0.1
**6**	***GS2->GS1***	Anti-TS	SMD(DMF)//B3LYP/6-31+G(d,p)	9.9	-6.0	11.1	
		Syn-TS	SMD(DMF)//B3LYP/6-31+G(d,p)	9.9	-5.2	11.4	11.1
		Anti-TS	SMD(DMF)//M062X/TZVP				
		Syn-TS	SMD(DMF)//M062X/TZVP				
	Exp. A to S			15.3 ± 1.0	9.6 ± 4.1		12.5 ± 0.1

Table S24. Theoretical thermodynamic parameters of restricted rotation around N-Ph bond

Comp.	Process	Via	Method	$\Delta H^{\neq}$ (298 K) kcal mol^{-1}	$\Delta S^{\neq}$ (298 K) cal K^{-1} mol^{-1}	$\Delta G^{\neq}$ (298 K) kcal mol^{-1}	$\Delta G^{\neq}_{eff}$ (298 K) kcal mol^{-1}
**11**	***GS1->GS3***	Anti-TS	B3LYP/6-31+G(d,p)	3.1	4.6	1.8	1.8
		Syn-TS	B3LYP/6-31+G(d,p)	4.5	-2.3	5.2	
		Anti-TS	SMD(DMF)//B3LYP/6-31+G(d,p)	1.6	-2.3	2.3	2.0
		Syn-TS	SMD(DMF)//B3LYP/6-31+G(d,p)	1.9	-1.8	2.4	
**11**	***GS3->GS1***	Anti-TS	B3LYP/6-31+G(d,p)	1.2	1.8	0.7	0.7
		Syn-TS	B3LYP/6-31+G(d,p)	2.6	-5.1	4.1	
		Anti-TS	SMD(DMF)//B3LYP/6-31+G(d,p)	0.1	-4.9	1.4	1.1
		Syn-TS	SMD(DMF)//B3LYP/6-31+G(d,p)	0.3	-4.4	1.6	

References:
1. Frisch, M.J.; Trucks, G.W.; Schlegel, H.B.; Scuseria, G.E.; Robb, M.A.; Cheeseman, J.R.; Scalmani, G.; Barone, V.; Mennucci, B.; Petersson, G.A., et al. Gaussian 09, Rev. D.01. In *Gaussian, Inc., Wallingford CT*, 2013.
2. Becke, A.D. Density-functional thermochemistry. III. The role of exact exchange. *The Journal of Chemical Physics* **1993**, *98*, 5648-5652.
3. Hariharan, P.C.; Pople, J.A. The influence of polarization functions on molecular orbital hydrogenation energies. *Theoretica Chimica Acta* **1973**, *28*, 213-222, doi:10.1007/BF00533485.
4. Zhao, Y.; Truhlar, D.G. Density Functionals with Broad Applicability in Chemistry. *Accounts of Chemical Research* **2008**, *41*, 157-167, doi:10.1021/ar700111a.
5. Zhao, Y.; Truhlar, D.G. The M06 suite of density functionals for main group thermochemistry, thermochemical kinetics, noncovalent interactions, excited states, and transition elements: two new functionals and systematic testing of four M06-class functionals and 12 other functionals. *Theoretical Chemistry Accounts* **2008**, *120*, 215-241, doi:10.1007/s00214-007-0310-x.
6. Schäfer, A.; Huber, C.; Ahlrichs, R. Fully optimized contracted Gaussian basis sets of triple zeta valence quality for atoms Li to Kr. *The Journal of Chemical Physics* **1994**, *100*, 5829-5835, doi:10.1063/1.467146.
7. Marenich, A.V.; Cramer, C.J.; Truhlar, D.G. Universal solvation model based on solute electron density and on a continuum model of the solvent defined by the bulk dielectric constant and atomic surface tensions. *Journal of Physical Chemistry B* **2009**, *113*, 6378-6396, doi:10.1021/jp810292n.
